# Ants of the Hengduan Mountains: a new altitudinal survey and updated checklist for Yunnan Province highlight an understudied insect biodiversity hotspot

**DOI:** 10.3897/zookeys.978.55767

**Published:** 2020-10-26

**Authors:** Cong Liu, Georg Fischer, Francisco Hita Garcia, Seiki Yamane, Qing Liu, Yan Qiong Peng, Evan P. Economo, Benoit Guénard, Naomi E. Pierce

**Affiliations:** 1 Department of Organismic and Evolutional Biology, Museum of Comparative Zoology, Harvard University, 26 Oxford Street, Cambridge, MA 02138, USA Harvard University Cambridge United States of America; 2 Biodiversity and Biocomplexity Unit, Okinawa Institute of Science and Technology Graduate University, Onna, Okinawa, Japan Okinawa Institute of Science and Technology Graduate University Onna Japan; 3 Kagoshima University Museum, Korimoto 1-21-30, Kagoshima-shi, Japan Kagoshima University Korimoto Japan; 4 School of Resources and Environment, Baoshan University, Baoshan city, Yunnan Province, China Baoshan University Baoshan China; 5 CAS Key Laboratory of Tropical Forest Ecology, Xishuangbanna Tropical Botanical Garden, Chinese Academy of Sciences, Mengla, Yunnan Province, China Xishuangbanna Tropical Botanical Garden, Chinese Academy of Sciences Kunming China; 6 School of Biological Sciences, The University of Hong Kong, Hong Kong SAR, China The University of Hong Kong Hong Kong China

**Keywords:** biodiversity hotspot, checklist, China, Formicidae, Hengduan Mountains, new records, species

## Abstract

China’s Hengduan Mountain region has been considered one of the most diverse regions in the northern hemisphere. Its stunning topography with many deep valleys and impassable mountain barriers has promoted an astonishing diversification in many groups of organisms including plants, birds, mammals, and amphibians. However, the insect biodiversity in this region is still poorly known. Here, the first checklist of ant species from the Southern Hengduan Mountain region is presented, generated by sampling ant diversity using a wide array of collection methods, including Winkler leaf litter extraction, vegetation beating, and hand collection. 130 species/morphospecies from nine subfamilies and 49 genera were identified. Among them, 17 species from 13 genera represent new records for Yunnan province, and eight species are newly recorded for China. Moreover, we believe 41 novel morphospecies (31% of the total collected taxa) will prove to be new to science. These results highlight the rich ant fauna of this region and strongly support its status as a biodiversity hotspot. The current ant species checklist for the whole of Yunnan Province was updated by recording 550 named species from 99 genera. Taken together, our results suggest that the Yunnan ant fauna still remains under-sampled, and future sampling will likely yield many more species, among them many undescribed ones.

## Introduction

The Hengduan Mountain region, located in the southeastern part of the Qinghai-Tibet Plateau, is one of the 35 recognized biodiversity hotspots in the world (Myers et al. 2000). The unique landscape, geomorphology, microhabitat differentiation and geographic isolation created by tectonic uplift during the last eight million years has promoted an astonishing diversification in many groups of organisms, making this region one of the most diverse temperate regions in the northern hemisphere (Boufford 2014; Price et al 2014; Xing and Ree 2017). For example, it harbors nearly 40 percent of China’s vascular plant diversity (ca. 12,000 species), including more than 3,000 endemic species (Boufford 2014). However, aside from the well-documented plants and some vertebrates, the diversity of other groups, especially invertebrates in this region remains largely unknown. Insect taxonomic groups in particular have received limited attention, and our understanding of their diversity in the Hengduan Mountains is extremely fragmented.

Ants are an ecologically dominant component of many ecosystems in terms of their abundance, richness, and ecosystem function (Hölldobler and Wilson 1990). Globally, about 15,600 ant species and subspecies have been described (Bolton 2020), making them the most diverse group of social insects and one of the most diverse families of insects. Despite the fact that ant diversity is mainly concentrated within tropical regions (Dunn et al. 2009; Guénard et al. 2012; Economo et al. 2018), the ant fauna of many other regions is still poorly known, especially in Asia (Guénard et al. 2010). Compiling and curating comprehensive and accurate ant species checklists for these regions is essential not only for insights into ant taxonomy and systematics, but also for long-term monitoring and conservation of these ecosystems (Guénard et al. 2017). The goal of this study is to provide a better understanding of the poorly known ant biodiversity in China’s Hengduan Mountains. The ultra-variable topography of this region, ideal for creating numerous vicariance events, combined with its wide range of climatic zones has contributed to the exceptional richness of endemic species inhabiting this area. Nevertheless, the rough topography has also made access and exploration rather challenging in the past. Against the background of extraordinary levels of plant diversity harbored by the Hengduan Mountains, it remains unclear whether or not ants and other insects display similar patterns of high diversity and endemism in this region.

To address this gap, we here present the results of an ant biodiversity survey conducted in the Gaoligong Shan mountains (part of the Hengduan Mountains), Yunnan Province, southwest China undertaken in 2019. Our goal is to present a complete species checklist of ants from the Gaoligong Mountains, including new records, as well as to update the current ant species checklist for the whole of Yunnan Province.

The Gaoligong Shan mountains (lat. 24°560'–28°220'N, long. 98°080'–98°500'E) comprise the western-most part of the Hengduan Mountain Range, and are among the most biodiversity-rich areas in Yunnan (Li et al. 2008; Dumbecher et al. 2011; Lo and Bi 2019). The ant fauna in the Gaoligong Shan mountains remains poorly understood, despite several studies focusing on ant diversity patterns that have recorded 62 ant species from 31 genera (Xu 2001a, b), but lack a comprehensive list of species collected.

Yunnan province is the richest province of China in terms of ant diversity (Guénard and Dunn 2012). The latest ant checklist of Yunnan was compiled almost 10 years ago and consisted of 462 ant species. Since then, new ant inventories have been conducted (e.g. Liu et al. 2015a), as well as new species descriptions (e.g., Guénard et al. 2013; Xu et al. 2014a, b; Liu et al. 2015b; Staab et al. 2018), and the identification of previously dubious records have sensibly modified our understanding of Yunnan’s ant diversity and species composition. Therefore, in this study, we also provide an update to the ant species checklist of Yunnan province and discuss future trends.

## Materials and methods

Ant specimens were collected from natural forests along an elevational gradient on both the eastern and western slopes of the Gaoligong Mountains in July 2019. We sampled leaf litter ants from 16 sites at roughly 150 m elevational intervals from 600 m to 3000 m, following the standardized sampling protocol developed in Liu et al. 2016. At each site, we established a 400 m^2^ quadrat (20 m × 20 m) and collected leaf litter samples at the four corners of the quadrat (1 m^2^). We also collected leaf litter within the quadrat to cover a variety of microhabitats. Finally, ants on the ground, lower vegetation, and tree branches were collected both by hand and using a beating sheet. Leaf litter samples were extracted using mini Winkler extractors for 72 hours using the shuffling method described in Guénard and Lucky (2011).

Ant specimens were first placed in 99% ethanol and later sorted into morphospecies and point mounted. Each mounted specimen was assigned a unique Museum of Comparative Zoology, Harvard University (**MCZ**) specimen code and collection labels. Extended depth of field specimen images were taken with a Leica DFC400 digital camera mounted on a Leica M205C stereomicroscope through the Leica Application Suite V4 software in the Ant Room at the MCZ. Specimens were identified to species / morphospecies using available keys, the digital resources on Antwiki (http://www.antwiki.org) and AntWeb (http://www.antweb.org), as well as reference museum material. All mounted and alcohol-preserved ant specimens are currently deposited in the Ant Room of the MCZ.

Distribution maps of species were generated from records included within the Global Ant Biodiversity Informatics (**GABI**) database and available at https://antmaps.org (Janicki et al, 2016; Guénard et al. 2017). These maps are based on records reported at the country level, or at the first administrative division for the larger countries (China, India, Japan). For larger islands that form their own natural biogeographic units like Borneo, Sumatra, New Guinea, the distribution maps used the island boundary instead of political boundaries (see also Guénard et al. 2012).

## Results

### Ants of the Hengduan Mountain region

More than 3000 specimens were collected during this survey, and 130 species and morphospecies in 49 genera and nine subfamilies were identified. After identification of 88 valid species from the 130 total collected species, a total of 17 new species records are presented for Yunnan province and eight represent new records for China (see Table 1). The newly recorded species belong to 13 genera from four subfamilies. Moreover, the 41 morphospecies that could not be identified are likely to represent new species.

**Table 1.** List of ant species (Formicidae) in the Gaoligong Shan mountains, Yunnan with their respective illustrations. * New to Yunnan province; **New to China.

Species	Figure
**Dorylinae**	
*Aenictus artipus* Wilson, 1964	Fig. 1
** *Aenictus brevinodus* Jaitrong & Yamane, 2011	Fig. 2
*Aenictus hodgsoni* Forel, 1901	Fig. 3
*Aenictus paradentatus* Jaitrong, Yamane & Tasen, 2012	Fig. 4
* *Aenictus watanasiti* Jaitrong & Yamane, 2013	Fig. 5
*Cerapachys sulcinodis* Emery, 1889	Fig. 6
*Cerapachys* sp. clm01	Fig. 7
*Chrysapace costatus* (Bharti & Wachkoo, 2013)	Fig. 8
*Dorylus orientalis* Westwood, 1835	Figs 9, 10
*Ooceraea biroi* (Forel, 1907)	Fig. 11
**Amblyoponinae**	
*Stigmatomma octodentatum* (Xu, 2006)	Fig. 12
**Dolichoderinae**	
*Dolichoderus feae* Emery, 1889	Fig. 13
*Dolichoderus squamanodus* Xu, 2001	Fig. 14
*Dolichoderus taprobanae* (Smith, 1858)	Fig. 15
*Ochetellus glaber* (Mayr, 1862)	Fig. 16
*Tapinoma melanocephalum* (Fabricius, 1793)	Fig. 17
**Ectatomminae**	
*Gnamptogenys quadrutinodules* Chen, Lattke & Zhou, 2017	Fig. 18
**Formicinae**	
*Anoplolepis gracilipes* (Smith, 1857)	Fig. 19
** *Camponotus bellus leucodiscus* Wheeler, 1919	Fig. 20
** *Camponotus keihitoi* Forel, 1913	Fig. 21
*Camponotus lasiselene* Wang & Wu, 1994	Figs 22, 23
*Camponotus mitis* (Smith, 1858)	Fig. 24
*Camponotus nicobarensis* Mayr, 1865	Fig. 25
*Camponotus* sp. clm01	Fig. 26
*Camponotus* sp. clm02	Fig. 27
*Camponotus* sp. clm03	Fig. 28
*Camponotus* sp. clm04	Fig. 29
*Camponotus* sp. clm05	Fig. 30
*Formica cunicularia* Latreille, 1798	Fig. 31
*Formica japonica* Motschoulsky, 1866	Fig. 32
* *Lasius obscuratus* Stitz, 1930	Fig. 33
* *Lasius himalayanus* Bingham, 1903	Fig. 34
*Nylanderia bourbonica* (Forel, 1886)	Fig. 35
*Nylanderia* sp. clm01	Fig. 36
*Nylanderia* sp. clm02	Fig. 37
*Oecophylla smaragdina* (Fabricius, 1775)	Fig. 38
*Paraparatrechina sakurae* (Ito, 1914)	Fig. 39
*Paraparatrechina* sp. clm01	Fig. 40
*Paraparatrechina* sp. clm02	Fig. 41
*Polyrhachis armata* (Le Guillou, 1842)	Fig. 42
*Polyrhachis bihamata* (Drury, 1773)	Fig. 43
*Polyrhachis dives* Smith, 1857	Fig. 44
*Polyrhachis furcata* Smith, 1858	Fig. 45
*Polyrhachis halidayi* Emery, 1889	Fig. 46
*Polyrhachis illaudata* Walker, 1859	Fig. 47
*Polyrhachis laevigata* Smith, 1857	Fig. 48
*Polyrhachis tibialis* Smith, 1858	Fig. 49
* *Prenolepis angularis* Zhou, 2001	Fig. 50
* *Prenolepis fustinoda* Williams & LaPolla, 2016	Fig. 51
*Prenolepis* sp. clm01	Fig. 52
*Prenolepis* sp. clm02	Fig. 53
*Pseudolasius emeryi* Forel, 1915	Fig. 54
*Pseudolasius silvestrii* Wheeler, 1927	Fig. 55
**Myrmicinae**	
*Aphaenogaster feae* Emery, 1889	Fig. 56
*Aphaenogaster* sp. clm01	Fig. 57
*Aphaenogaster* sp. clm02	Fig. 58
*Aphaenogaster* sp. clm03	Fig. 59
*Aphaenogaster* sp. clm04	Fig. 60
*Aphaenogaster* sp. clm05	Fig. 61
* *Cardiocondyla itsukii* Seifert, Okita & Heinze, 2017	Fig. 62
*Cardiocondyla* sp. clm01	Fig. 63
*Carebara acutispina* (Xu, 2003)	Fig. 64
*Carebara affinis* (Jerdon, 1851)	Fig. 65
*Carebara altinoda* (Xu, 2003)	Fig. 66
*Carebara bihornata* (Xu, 2003)	Fig. 67
*Carebara* sp. clm01	Fig. 68
* *Cataulacus marginatus* Bolton, 1974	Fig. 69
*Crematogaster quadriruga* Forel, 1911	Fig. 70
*Crematogaster* sp. clm01	Fig. 71
*Crematogaster* sp. clm02	Fig. 72
** *Dilobocondyla eguchii* Bharti & Kumar, 2013	Fig. 73
*Gaoligongidris planodorsa* Xu, 2012	Fig. 74
*Gauromyrmex* sp. clm01	Fig. 75
*Lordomyrma* sp. clm01	Fig. 76
*Monomorium pharaonis* (Linnaeus, 1758)	Fig. 77
*Monomorium* sp. clm01	Fig. 78
*Myrmica draco* Radchenko, Zhou & Elmes, 2001	Fig. 79
*Myrmica pleiorhytida* Radchenko & Elmes, 2009	Fig. 80
*Myrmica* sp. clm01	Fig. 81
*Myrmecina* sp. clm01	Fig. 82
*Myrmecina* sp. clm02	Fig. 83
*Myrmecina* sp. clm03	Fig. 84
*Pheidole allani* Bingham, 1903	Figs 85, 86
*Pheidole fervens* Smith, 1858	Fig. 87
*Pheidole fervida* Smith, 1874	Fig. 88, 89
*Pheidole gatesi* (Wheeler, 1927)	Fig. 90
*Pheidole indica* Mayr, 1879	Fig. 91
*Pheidole magna* Eguchi, 2006	Figs 92, 93
* *Pheidole nodifera* (Smith 1858)	Fig. 94
*Pheidole zoceana* Santschi, 1925	Figs 95, 96
*Pristomyrmex brevispinosus* Emery, 1887	Fig. 97
*Pristomyrmex hamatus* Xu & Zhang, 2002	Fig. 98
*Stenamma wumengense* Liu & Xu, 2011	Fig. 99
*Strumigenys assamensis* De Andrade, 1994	Fig. 100
*Strumigenys strygax* Bolton, 2000	Fig. 101
** *Strumigenys taphra* (Bolton, 2000)	Fig. 102
*Strumigenys* sp. clm01	Fig. 103
*Strumigenys* sp. clm02	Fig. 104
*Strumigenys* sp. clm03	Fig. 105
* *Temnothorax striatus* Zhou, Huang, Yu & Liu, 2010	Fig. 106
*Temnothorax* sp. clm01	Fig. 107
*Temnothorax* sp. clm03	Fig. 108
*Tetramorium tonganum* Mayr, 1870	Fig. 109
*Tetramorium* sp. clm01	Fig. 110
*Tetramorium* sp. clm02	Fig. 111
*Tetramorium* sp. clm03	Fig. 112
*Tetramorium* sp. clm04	Fig. 113
*Vollenhovia pyrrhoria* Wu & Xiao, 1989	Fig. 114
*Vollenhovia* sp. clm03	Fig. 115
**Ponerinae**	
*Brachyponera luteipes* (Mayr, 1862)	Fig. 116
*Ectomomyrmex lobocarenus* (Xu, 1995)	Fig. 117
** *Ectomomyrmex obtusus* Emery, 1900	Fig. 118
*Hypoponera* sp. clm01	Fig. 119
*Hypoponera* sp. clm02	Fig. 120
*Hypoponera* sp. clm03	Fig. 121
*Leptogenys birmana* Forel, 1900	Fig. 122
*Leptogenys kitteli* (Mayr, 1870)	Fig. 123
*Odontomachus circulus* Wang, 1993	Fig. 124
* *Odontomachus fulgidus* Wang, 1993	Fig. 125
*Platythyrea parallela* (Smith, 1859)	Fig. 126
*Ponera bawana* Xu, 2001	Fig. 127
*Ponera xantha* Xu, 2001	Fig. 128
**Proceratinae**	
*Discothyrea banna* Xu, Burwell & Nakamura, 2014	Fig. 129
*Discothyrea diana* Xu, Burwell & Nakamura, 2014	Fig. 130
*Proceratium longigaster* Karavaiev, 1935	Fig. 131
*Proceratium longmenense* Xu, 2006	Fig. 132
*Proceratium zhaoi* Xu, 2000	Fig. 133
**Pseudomyrmecinae**	
*Tetraponera allaborans* (Walker, 1859)	Fig. 134
*Tetraponera attenuata* Smith, 1877	Fig. 135
*Tetraponera protensa* Xu & Chai, 2004	Fig. 136

Within the recent collection, the most speciose ant genus is *Pheidole* with eleven species (8.5% of the total species collected in the survey), followed by *Camponotus* (ten species, 7.7%), and *Polyrhachis* (seven species, 5.4%). Other diverse genera include *Aphaenogaster* (6 species, 4.6%), *Strumigenys* (six species, 4.6%), *Tetramorium* (six species, 4.6%), *Aenictus* (five species, 3.8%), and *Carebara* (five species, 3.8%). More details are presented in Table 2.

Here, we present the list of ant species that were collected in the Gaoligong Shan mountains (Table 1), as well as images for each species (Figs 1–136).

**Table 2.** Number of ant species of per genus collected in this survey as well the total number of each species per genus in Yunnan province.

Genus	Gaoligongshan Mt.	Yunnan	Genus	Gaoligongshan Mt.	Yunnan
* Camponotus *	10	30	* Leptogenys *	2	17
* Pheidole *	8	42	* Monomorium *	2	6
* Polyrhachis *	8	32	* Odontomachus *	2	6
* Aphaenogaster *	6	10	* Ponera *	2	14
* Strumigenys *	6	24	* Pristomyrmex *	2	4
* Tetramorium *	5	29	* Pseudolasius *	2	6
* Aenictus *	5	19	* Vollenhovia *	2	3
* Carebara *	5	19	* Anoplolepis *	1	1
* Prenolepis *	4	7	* Brachyponera *	1	3
* Crematogaster *	3	25	* Cataulacus *	1	4
* Dolichoderus *	3	9	* Chrysapace *	1	1
* Hypoponera *	3	7	* Dilobocondyla *	1	3
* Lasius *	2	6	* Dorylus *	1	3
* Myrmica *	3	12	* Gaoligongidris *	1	1
* Myrmecina *	3	7	* Gauromyrmex *	1	1
* Nylanderia *	3	10	* Gnamptogenys *	1	7
* Paraparatrechina *	3	2	* Lordomyrma *	1	1
* Proceratium *	3	4	* Ochetellus *	1	1
* Temnothorax *	3	7	* Oecophylla *	1	1
* Tetraponera *	3	12	* Ooceraea *	1	1
* Cardiocondyla *	2	4	* Platythyrea *	1	2
* Cerapachys *	2	1	* Stenamma *	1	4
* Discothyrea *	2	3	* Stigmatoma *	1	11
* Ectomomyrmex *	2	8	* Tapinoma *	1	4
* Formica *	2	7	**Total**	**130**	**550**

### Updated ant checklist in Yunnan

The ant species list of Yunnan Province was generated using records from GABI available at https://antmaps.org (Janicki et al. 2016; Guénard et al. 2017). In total, the Yunnan ant fauna is composed of 99 genera and 550 named species and subspecies. Among them, the ant genera *Lasiomyrma*, *Lordomyrma*, and *Prionopelta* are only known from unidentified morphospecies. Through our collection and the records from GABI, we have added 125 species and subspecies to the list of ants of Yunnan since the last ant checklist (Guénard et al. 2012). We also excluded 26 species records from the previous list and explained our rationale in each case (Table 3).

**Table 3.** Ant species records that have been excluded from Yunnan when compared to the previous list. The explanation “Needs verification” usually signifies that the species has never been recorded before in this region and/or is easily mistaken for another species and likely to have been misidentified. “Dubious” means that the record occurrence is highly unlikely given the known species distribution. Notes provide additional references regarding records and/or further information.

Excluded species records	Explanations	Notes
*Camponotus aethiops*	Needs verification	A Palearctic species with distribution in Asia needs confirmation
*Camponotus spenceri*	Dubious	An Australian species misreported previously
*Cardiocondyla nuda*	Dubious	Could be *C. kagutsuchi*, see Seifert 2003
*Discothyrea clavicornis*	Dubious	A misidentification of *D. diana*
*Discothyrea kamiteta*	Dubious	A misidentification of *D. banna*
*Formica fusca*	Needs verification	A Palearctic species with distribution in Asia needs confirmation
*Hypoponera exoecata*	Needs verification	Species with distribution limited to East Asia
*Lasius alienus*	Dubious	See Seifert 2020
*Lasius emarginatus*	Dubious	A West Palearctic species with distribution in Asia doubtful
*Lasius fuliginosus*	Dubious	See Espadaler et al. 2001
*Lasius niger*	Dubious	See Seifert 1992
*Lasius productus*	Needs verification	Species with distribution limited to Japan and the Korean Peninsula
*Lasius spathepus*	Needs verification	Species with distribution limited to Japan, the Korean Peninsula and Eastern Russia
*Leptogenys yerburyi*	Dubious	See Xu and He 2015
*Myrmica inezae*	Needs verification	See Chen et al. 2016.
*Odontoponera transversa*	Dubious	See Yamane 2009
*Proceratium deelemani*	Dubious	Record represented a new species subsequently described in Staab et al. 2018.
*Proceratium japonicum*	Dubious	A misidentification of *P. longigaster*
*Temnothorax melleus*	Needs verification	A central Asian species which presence in Yunnan requires confirmation
*Tetramorium inglebyi*	Dubious	An Indian species that is restricted to the Southwest.
*Tetramorium globulinode*	Dubious	An Afrotropical species incorrectly reported in Asia
*Tetramorium khnum*	Dubious	An endemic species in the Philippines
*Tetramorium melleum*	Dubious	A misidentification of *T. wroughtonii*
*Tetraponera aitkenii*	Dubious	Phil Ward (Personal communication, 18 August 2015)
*Tetraponera nigra*	Dubious	Phil Ward (Personal communication, 18 August 2015)
*Vollenhovia emeryi*	Dubious	See Wetterer et al. 2015

In Yunnan, the most diverse ant genus is *Pheidole* with 42 named species, followed by *Polyrhachis* (33 species), *Camponotus* (30 species), and *Tetramorium* (29 species). Other diverse genera include *Crematogaster* (25 species), and *Strumigenys* (25 species). Although 15 ant genera contain more than ten named species in Yunnan, the majority of ant genera occurring in Yunnan seem to be not particularly diverse. For example, 35 genera are represented by only one species in Yunnan (Table 4).

**Table 4.** Number of ant species (both native and exotic species) in Yunnan Province. * Ant genus only known from morphospecies records.

Genus	Native	Exotic	Genus	Native	Exotic
* Pheidole *	42	0	* Solenopsis *	2	1
* Polyrhachis *	32	0	* Acanthomyrmex *	2	0
* Camponotus *	30	0	* Acropyga *	2	0
* Tetramorium *	28	1	* Echinopla *	2	0
* Crematogaster *	25	0	* Meranoplus *	2	0
* Strumigenys *	24	1	* Myrmoteras *	2	0
* Aenictus *	19	0	* Paraparatrechina *	2	0
* Carebara *	19	0	* Perissomyrmex *	2	0
* Leptogenys *	17	0	* Platythyrea *	2	0
* Ponera *	14	0	* Pseudoneoponera *	2	0
* Tetraponera *	12	0	* Rhopalomastix *	2	0
* Myrmica *	12	0	* Trichomyrmex *	0	2
* Stigmatomma *	11	0	* Vollenhovia *	2	0
* Technomyrmex *	11	0	* Anoplolepis *	1	0
* Aphaenogaster *	10	0	* Buniapone *	1	0
* Nylanderia *	9	1	* Centromyrmex *	1	0
* Dolichoderus *	9	0	* Cerapachys *	1	0
* Ectomomyrmex *	8	0	* Chrysapace *	1	0
* Lepisiota *	8	0	* Diacamma *	1	0
* Colobopsis *	7	0	* Emeryopone *	1	0
* Hypoponera *	5	2	* Erromyrma *	1	0
* Prenolepis *	7	0	* Euponera *	1	0
* Temnothorax *	7	0	* Gaoligongidris *	1	0
* Formica *	7	0	* Gauromyrmex *	1	0
* Gnamptogenys *	7	0	* Gesomyrmex *	1	0
* Myrmecina *	7	0	* Harpegnathos *	1	0
* Anochetus *	6	0	* Iridomyrmex *	1	0
* Lasius *	6	0	Lasiomyrma*	1	0
* Odontomachus *	6	0	* Liometopum *	1	0
* Pseudolasius *	6	0	* Lioponera *	1	0
* Cryptopone *	5	0	Lordomyrma*	1	0
* Monomorium *	5	0	* Mesoponera *	1	0
* Proceratium *	4	0	* Messor *	1	0
* Cataulacus *	4	0	* Myrmicaria *	1	0
* Plagiolepis *	3	1	* Mystrium *	1	0
* Pristomyrmex *	4	0	* Ochetellus *	1	0
* Protanilla *	4	0	* Odontoponera *	1	0
* Stenamma *	4	0	* Oecophylla *	1	0
* Tapinoma *	4	0	* Ooceraea *	1	0
* Brachyponera *	3	0	* Parasyscia *	1	0
* Cardiocondyla *	2	1	* Paratrechina *	0	1
* Chronoxenus *	3	0	* Philidris *	1	0
* Dilobocondyla *	3	0	Prionopelta*	1	0
* Discothyrea *	3	0	* Probolomyrmex *	1	0
* Dorylus *	3	0	* Rotastruma *	1	0
* Kartidris *	3	0	* Simopone *	1	0
* Leptanilla *	3	0	* Syscia *	1	0
* Lophomyrmex *	3	0	* Vombisidris *	1	0
* Myopias *	3	0	* Yunodorylus *	1	0
* Recurvidris *	3	0			

**Figure 1.**
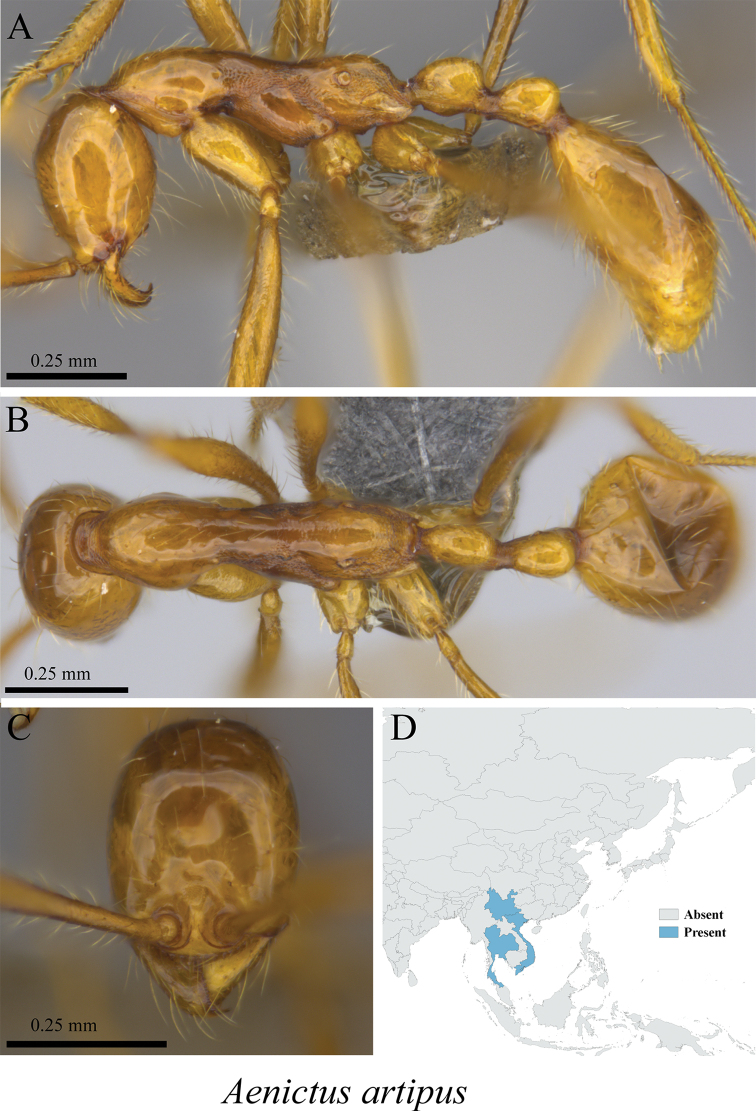
*Aenictus artipus* worker (MCZ-ENT00763651) **A** mesosoma in profile view **B** mesosoma in dorsal view **C** head in front view **D** global distribution map.

**Figure 2.**
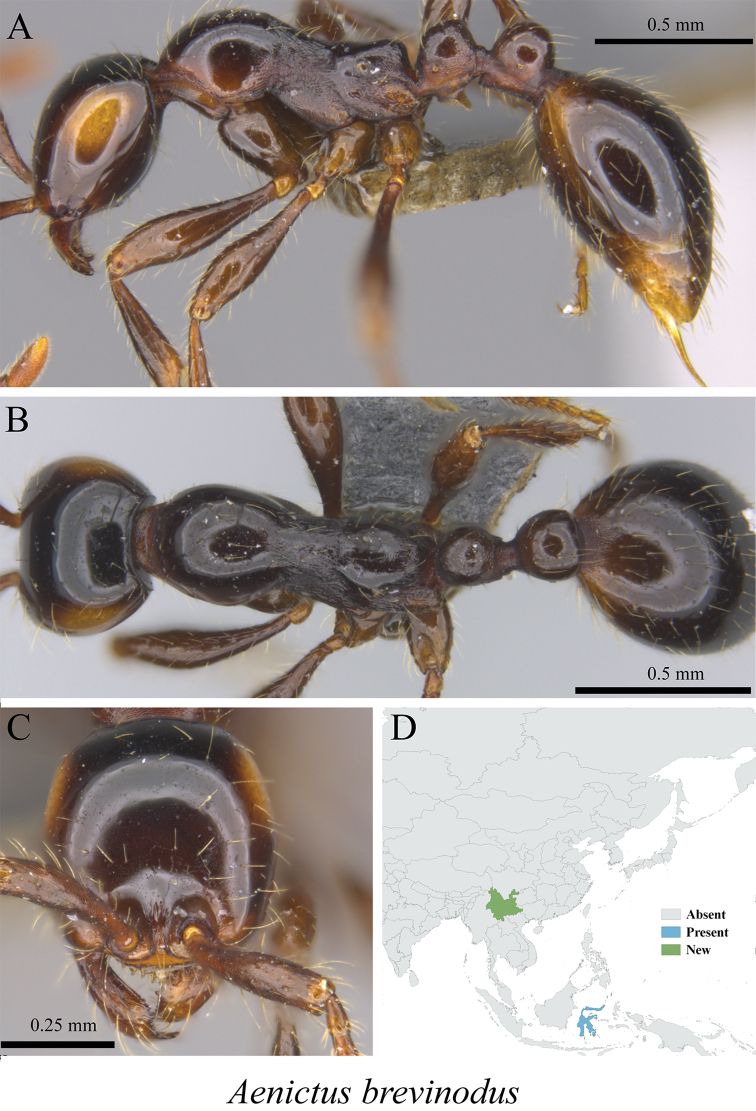
*Aenictus brevinodus* worker (MCZ-ENT00763491, new to China) **A** mesosoma in profile view **B** mesosoma in dorsal view **C** head in front view **D** global distribution map.

**Figure 3.**
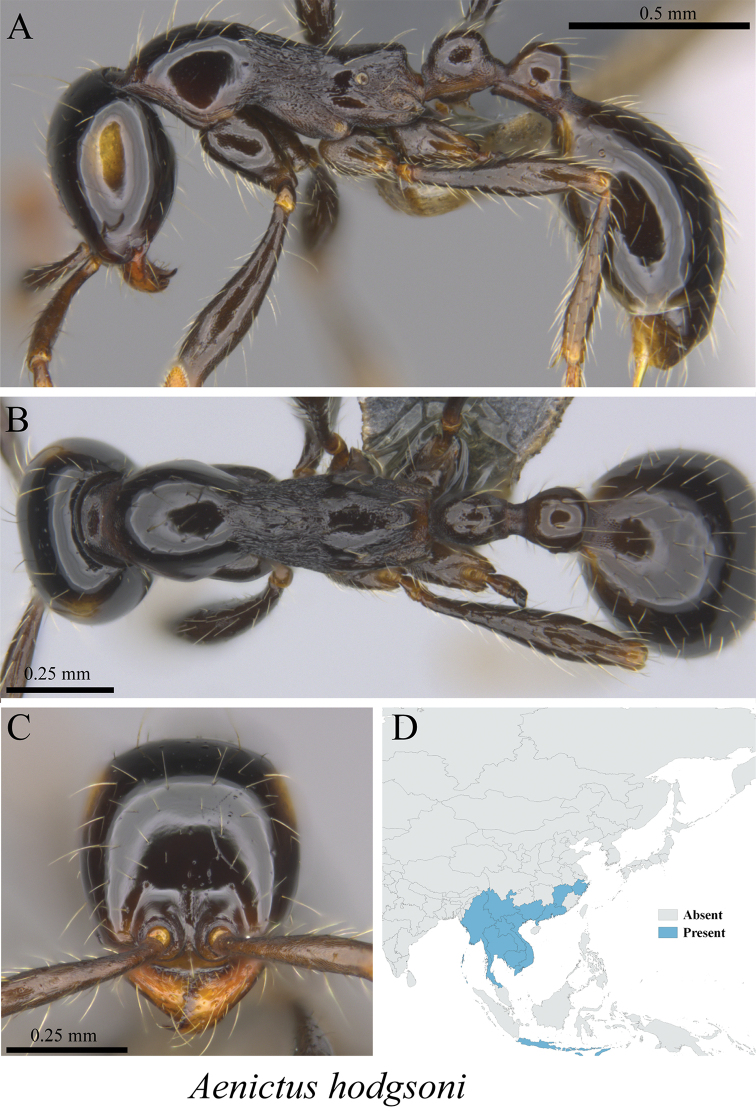
*Aenictus hodgsoni* worker (MCZ-ENT00763191) **A** mesosoma in profile view **B** mesosoma in dorsal view **C** head in front view **D** global distribution map.

**Figure 4.**
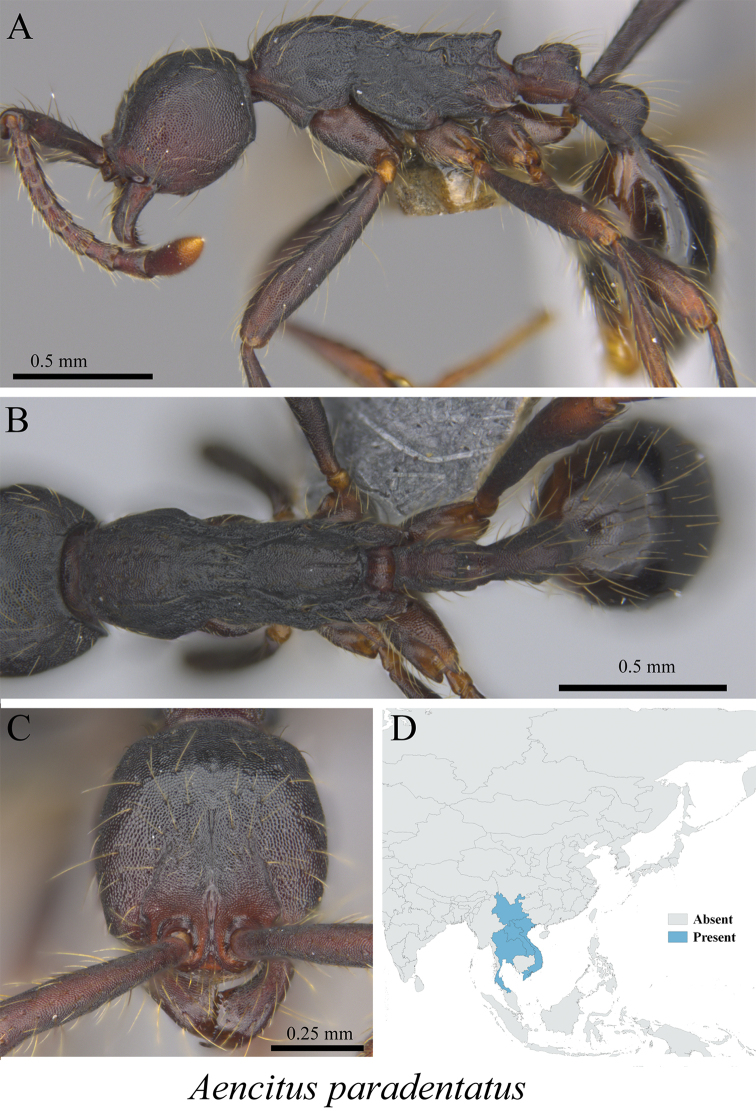
*Aenictus paradentatus* worker (MCZ-ENT00763384) **A** mesosoma in profile view **B** mesosoma in dorsal view **C** head in front view **D** global distribution map.

**Figure 5.**
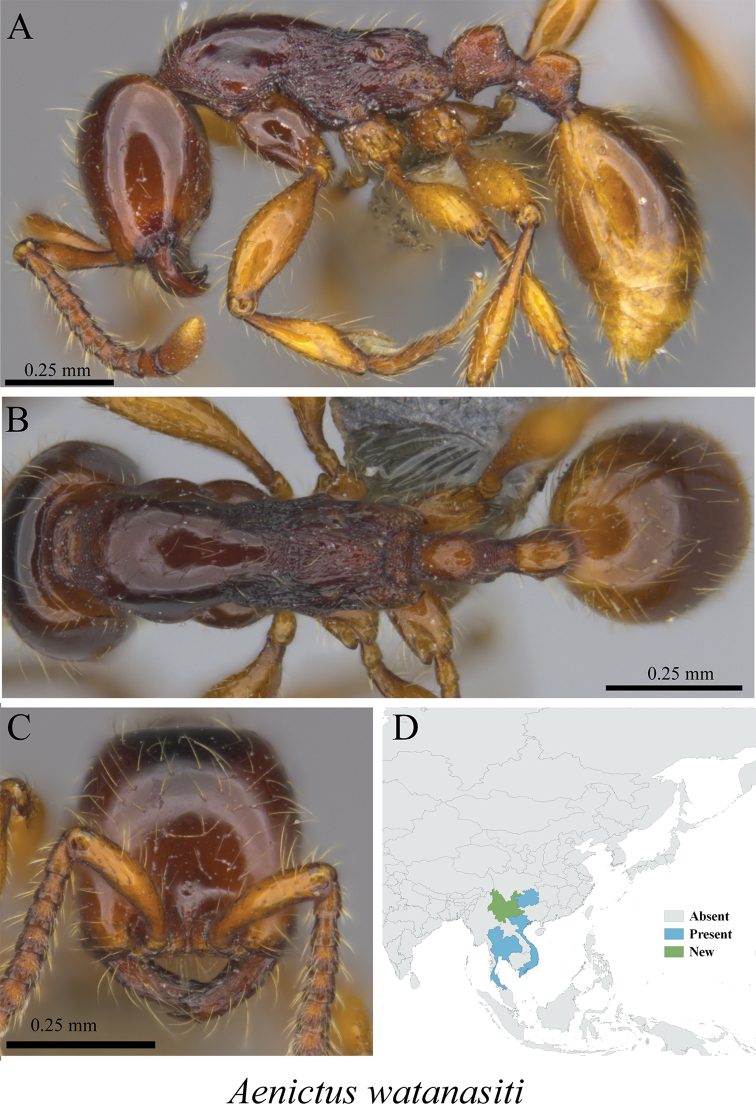
*Aenictus watanasiti* worker (MCZ-ENT00764608, new to Yunnan) **A** mesosoma in profile view **B** mesosoma in dorsal view **C** head in front view **D** global distribution map.

**Figure 6.**
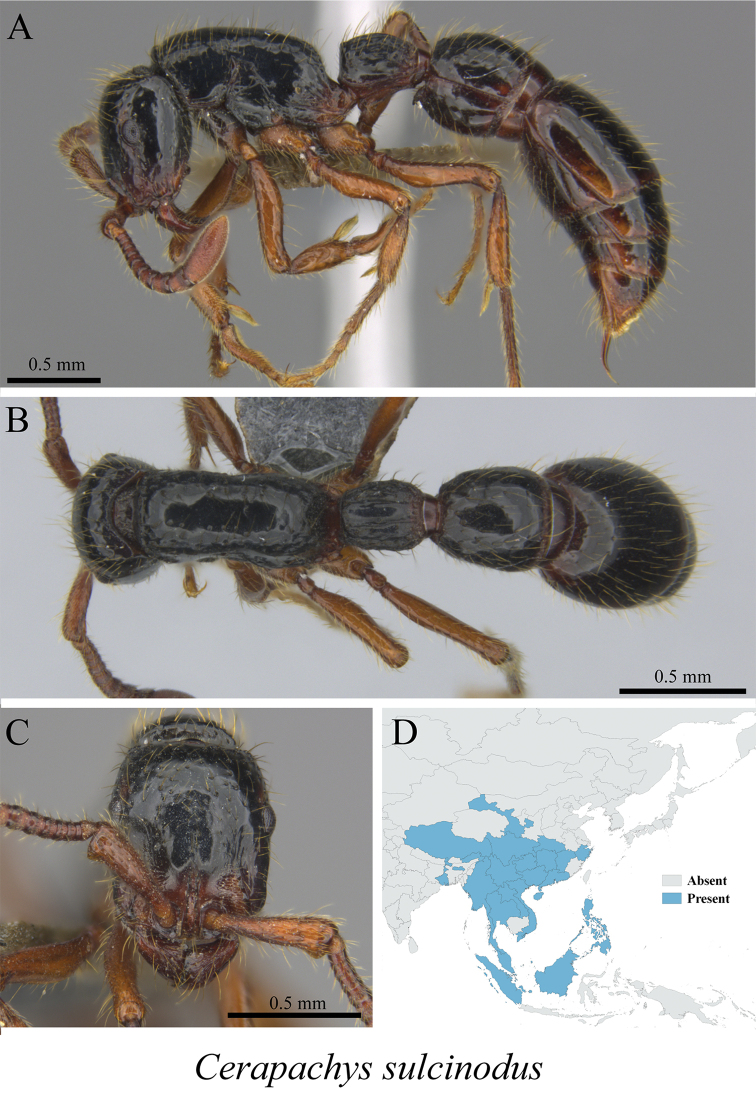
*Cerapachys sulcinodis* worker (MCZ-ENT00759751) **A** mesosoma in profile view **B** mesosoma in dorsal view **C** head in front view **D** global distribution map.

**Figure 7.**
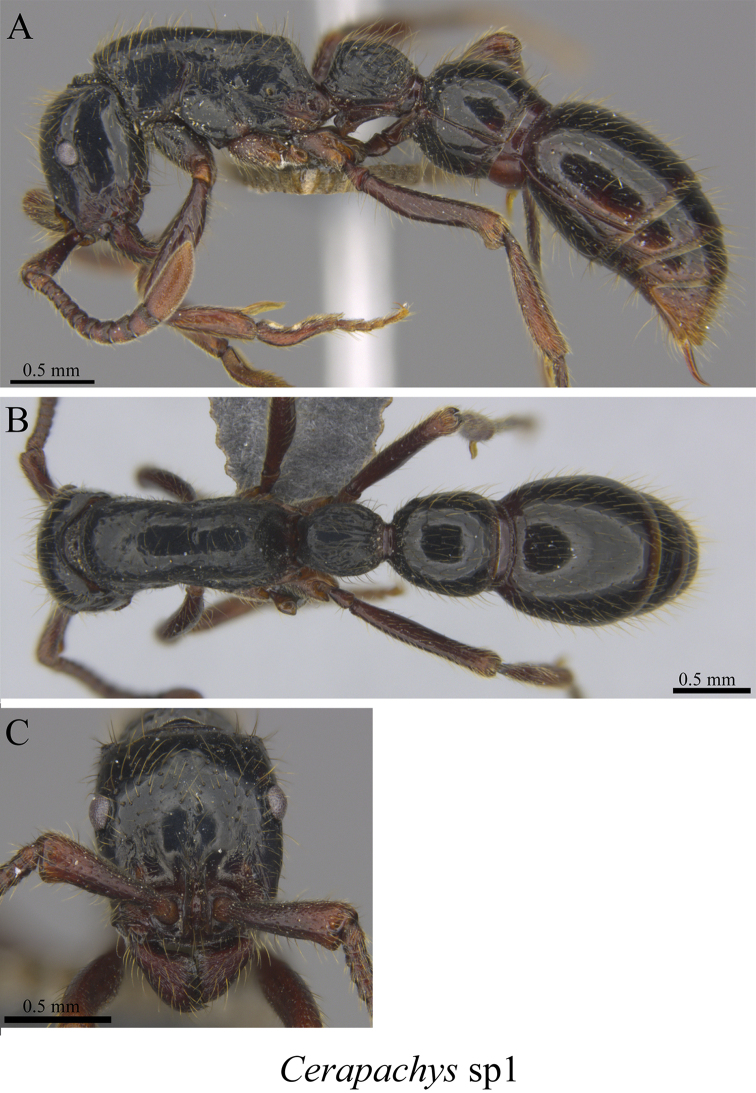
*Cerapachys* sp. clm01worker (MCZ-ENT00763371) **A** mesosoma in profile view **B** mesosoma in dorsal view **C** head in front view.

**Figure 8.**
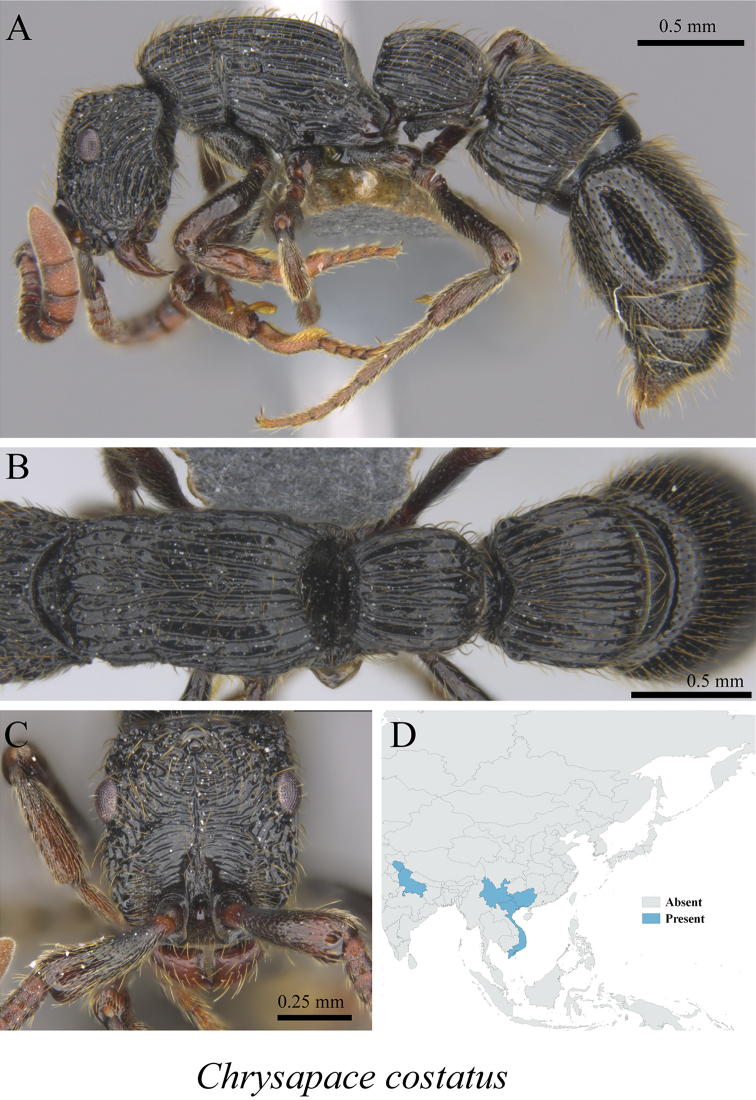
*Chrysapace costatus* worker (MCZ-ENT00763341) **A** mesosoma in profile view **B** mesosoma in dorsal view **C** head in front view **D** global distribution map.

**Figure 9.**
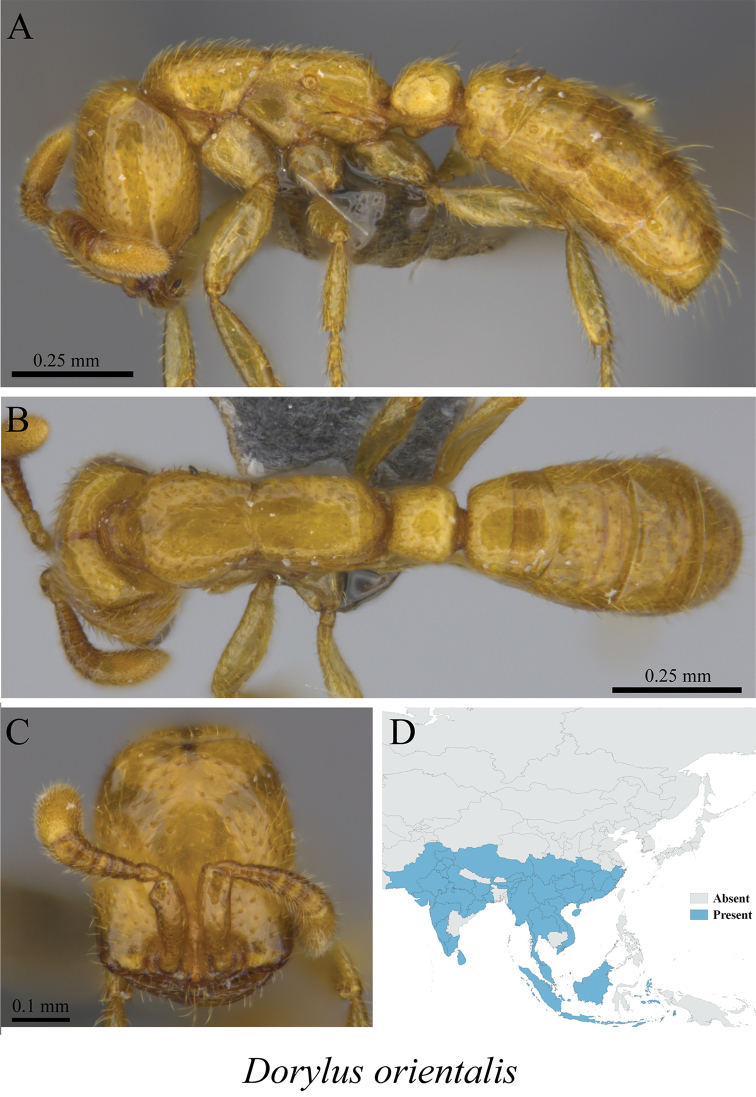
*Dorylus orientalis* minor worker (MCZ-ENT00760027) **A** mesosoma in profile view **B** mesosoma in dorsal view **C** head in front view **D** global distribution map.

**Figure 10.**
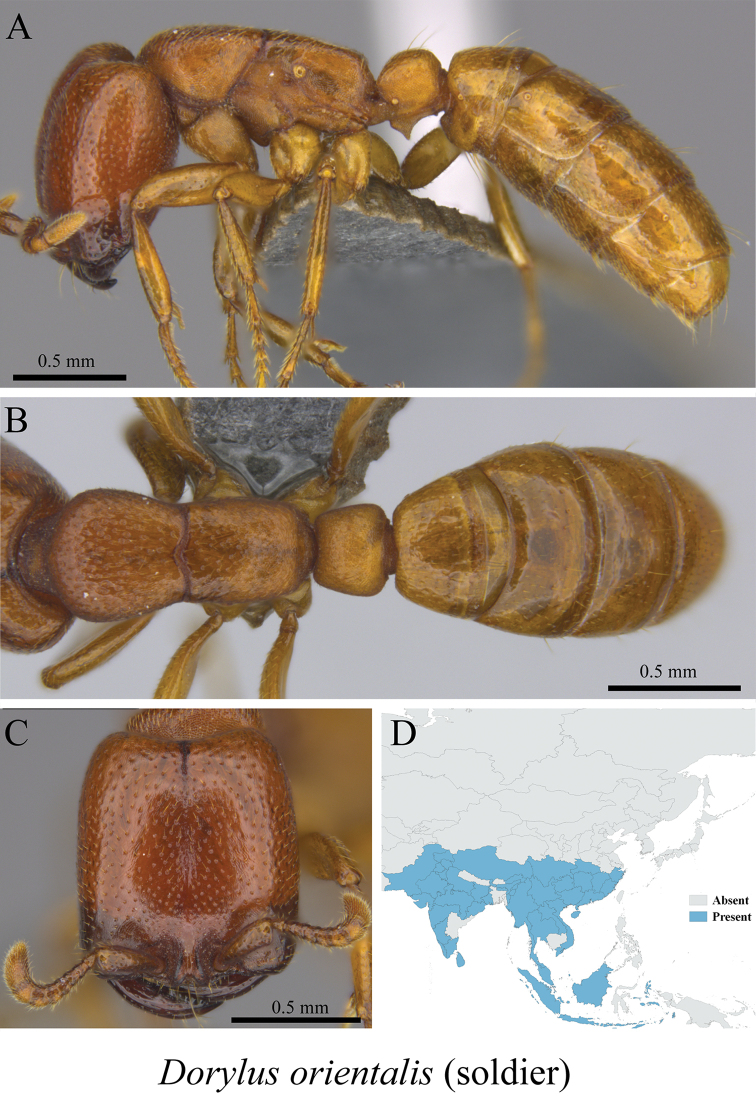
*Dorylus orientalis* major worker (MCZ-ENT00760028) **A** mesosoma in profile view **B** mesosoma in dorsal view **C** head in front view **D** global distribution map.

**Figure 11.**
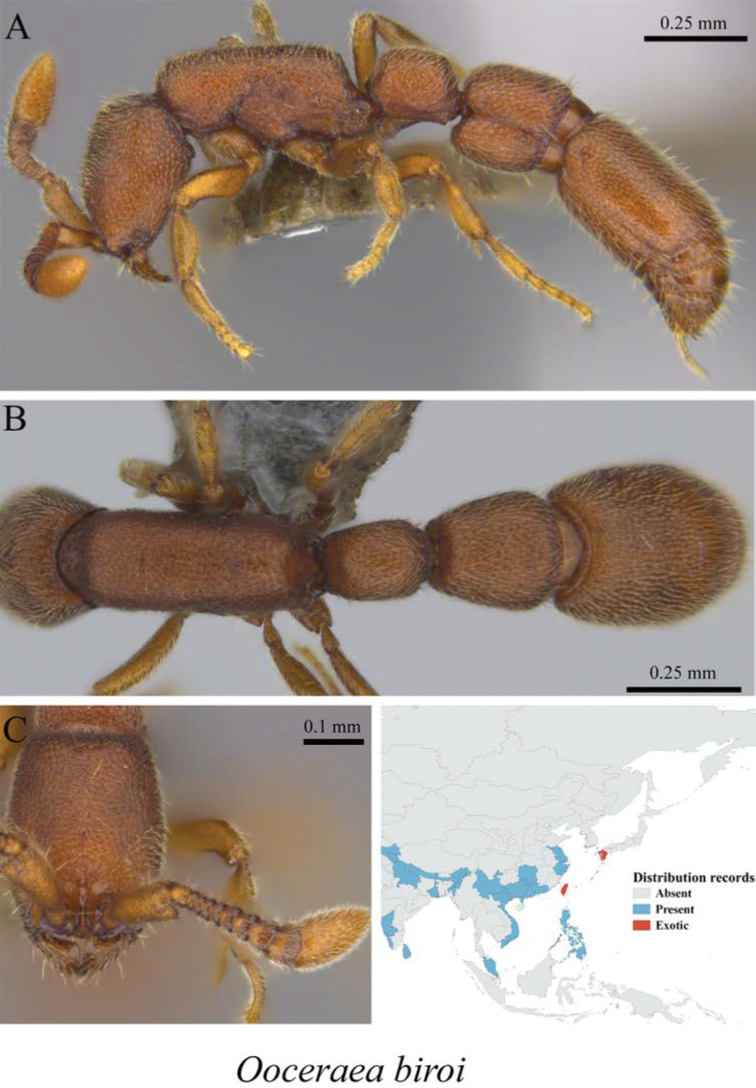
*Ooceraea biroi* worker (MCZ-ENT00759984) **A** mesosoma in profile view **B** mesosoma in dorsal view **C** head in front view **D** global distribution map.

**Figure 12.**
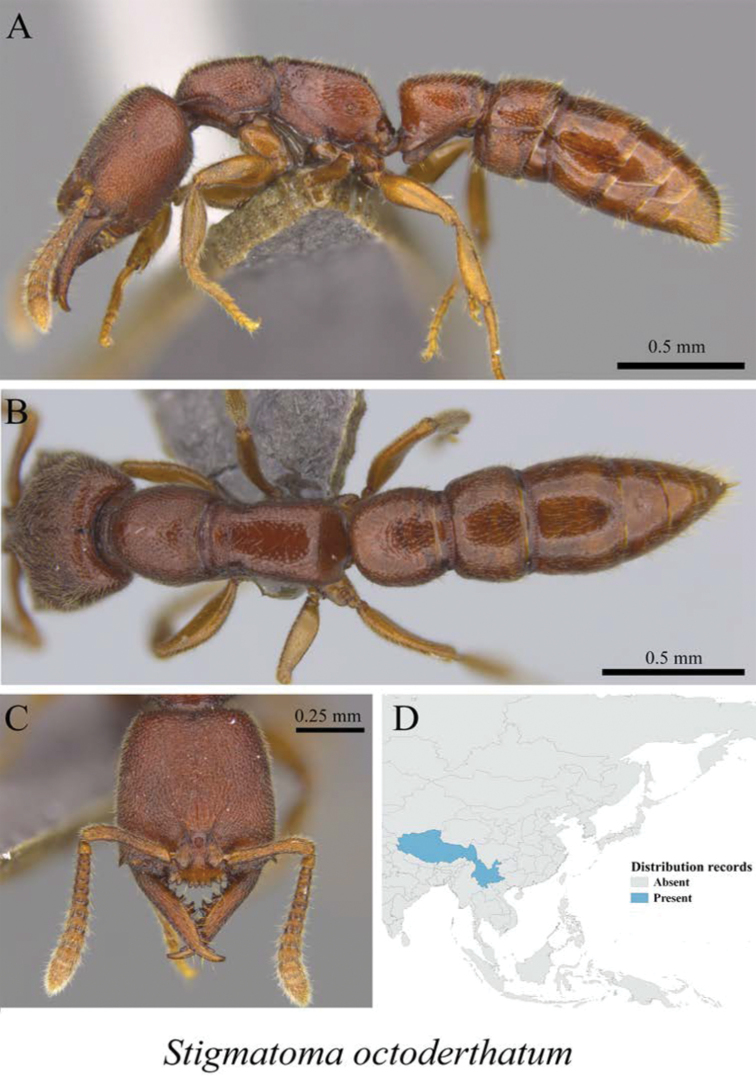
*Stigmatomma octodentatum* worker (MCZ-ENT00759880) **A** mesosoma in profile view **B** mesosoma in dorsal view **C** head in front view **D** global distribution map.

**Figure 13.**
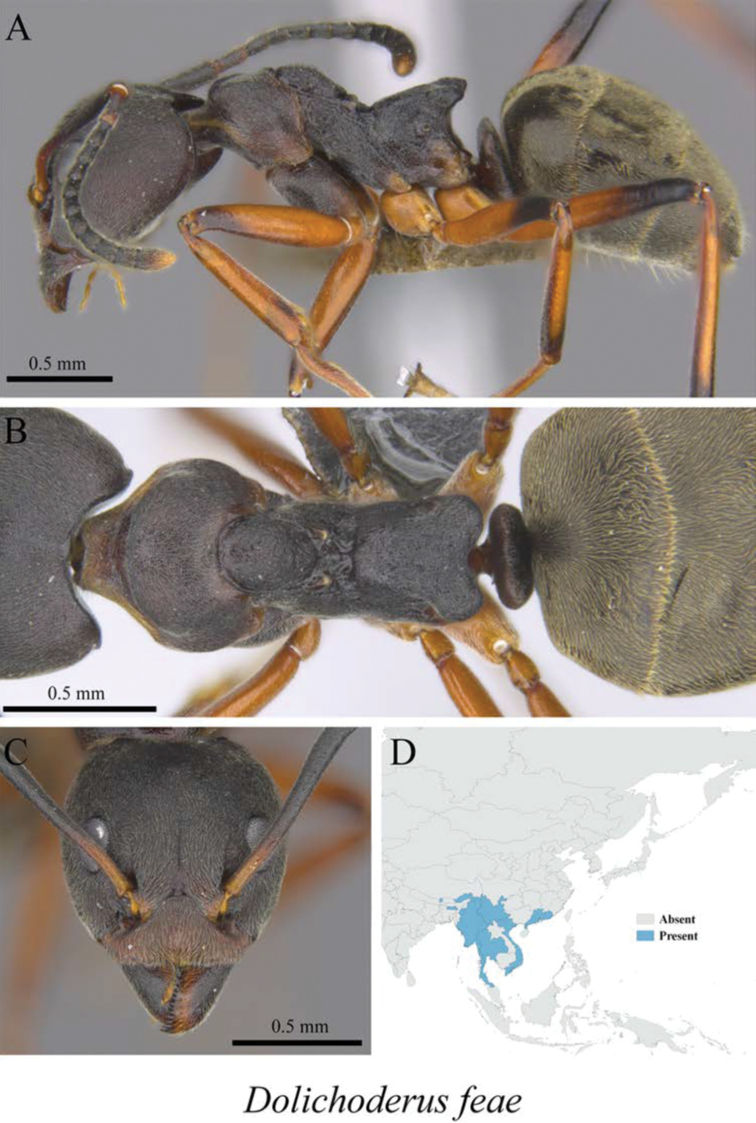
*Dolichoderus feae* worker (MCZ-ENT00763272) **A** mesosoma in profile view **B** mesosoma in dorsal view **C** head in front view **D** global distribution map.

**Figure 14.**
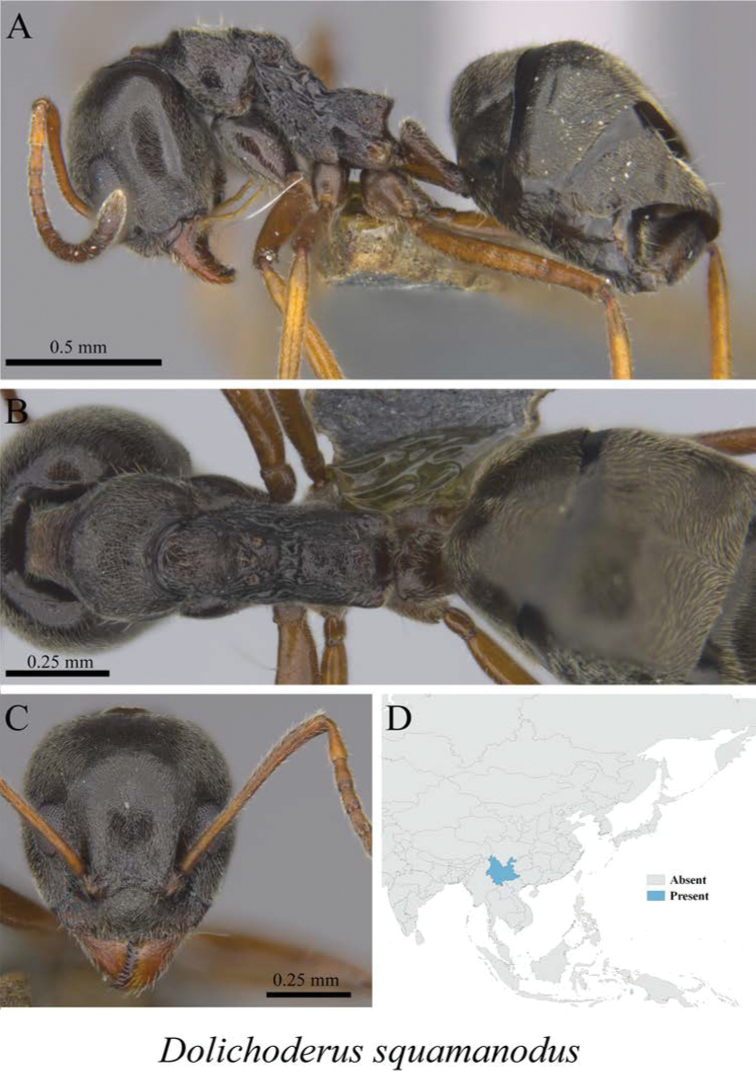
*Dolichoderus squamanodus* worker (MCZ-ENT00762839) **A** mesosoma in profile view **B** mesosoma in dorsal view **C** head in front view **D** global distribution map.

**Figure 15.**
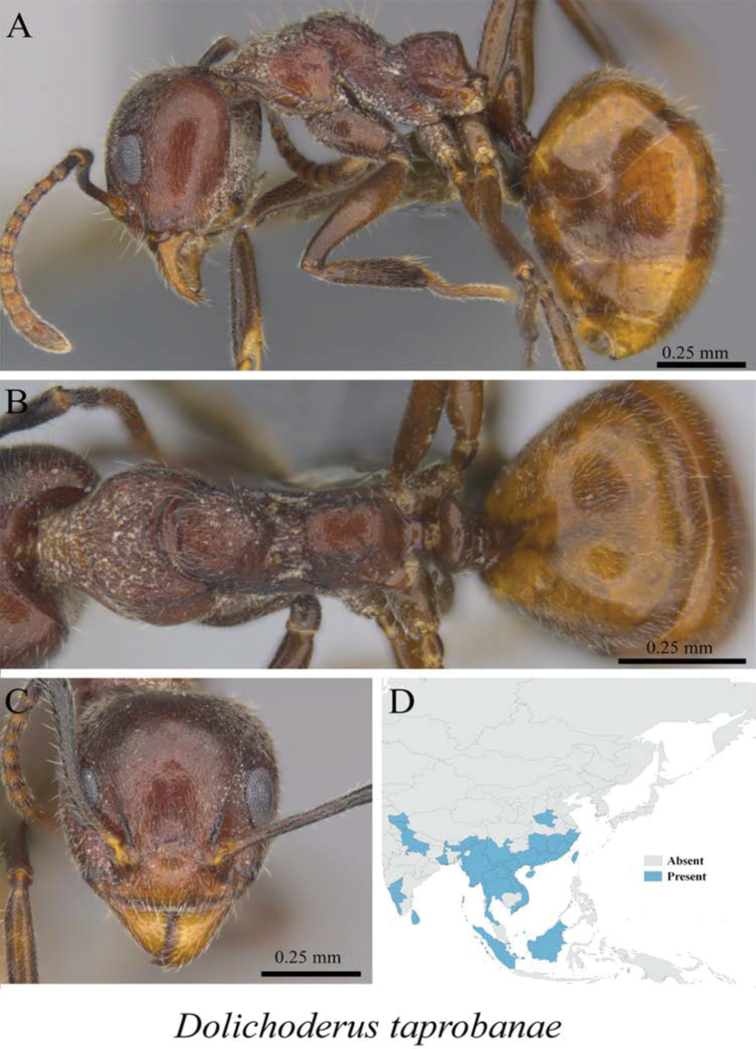
*Dolichoderus taprobanae* worker (MCZ-ENT00763246) **A** mesosoma in profile view **B** mesosoma in dorsal view **C** head in front view **D** global distribution map.

**Figure 16.**
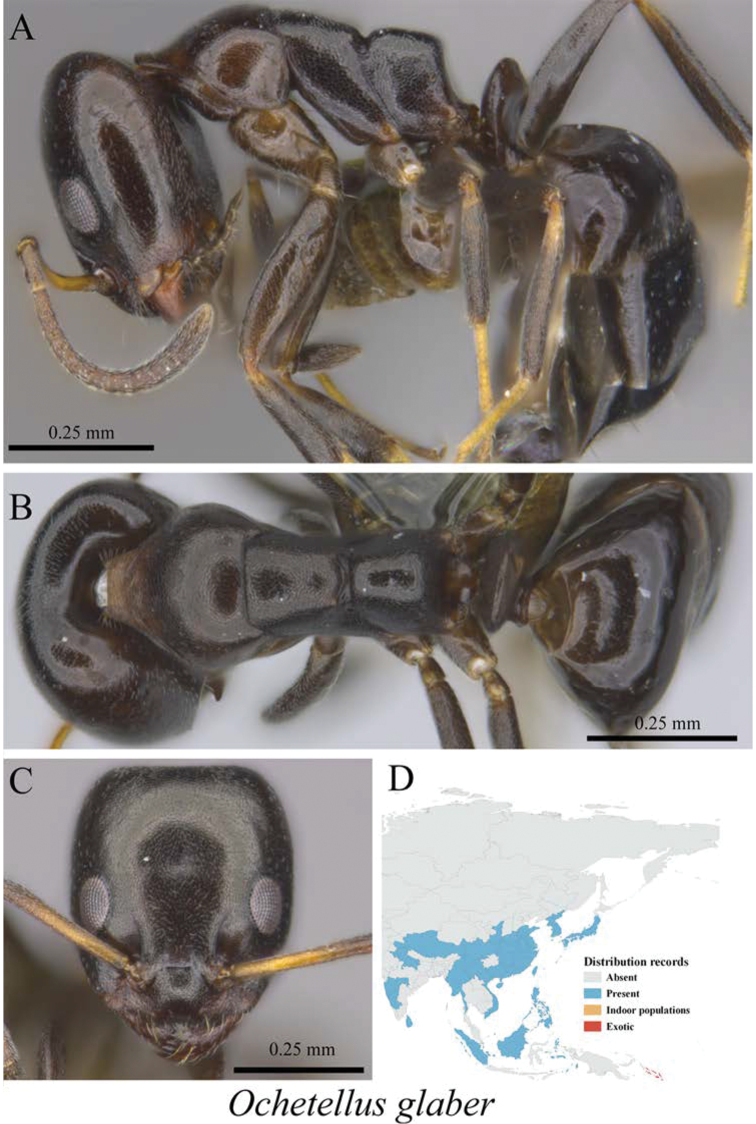
*Ochetellus glaber* worker (MCZ-ENT00763401) **A** mesosoma in profile view **B** mesosoma in dorsal view **C** head in front view **D** global distribution map.

**Figure 17.**
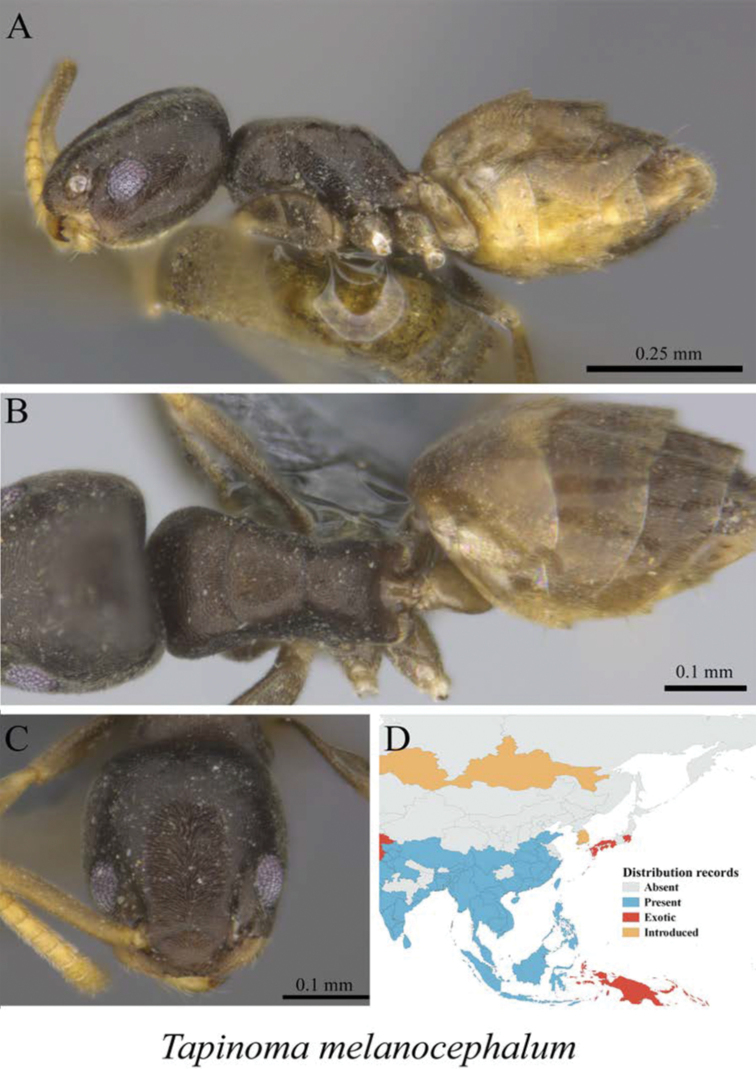
*Tapinoma melanocephalum* worker (MCZ-ENT00760062) **A** mesosoma in profile view **B** mesosoma in dorsal view **C** head in front view **D** global distribution map.

**Figure 18.**
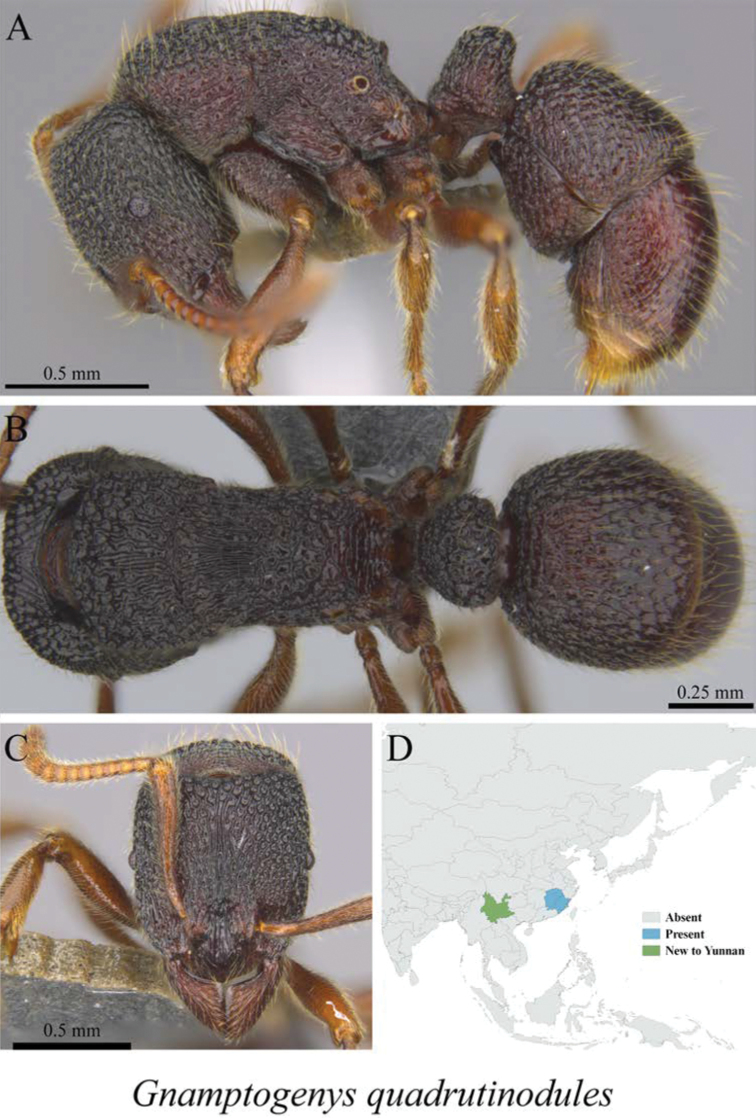
*Gnamptogenys quadrutinodules* worker (MCZ-ENT00759741) **A** mesosoma in profile view **B** mesosoma in dorsal view **C** head in front view **D** global distribution map.

**Figure 19.**
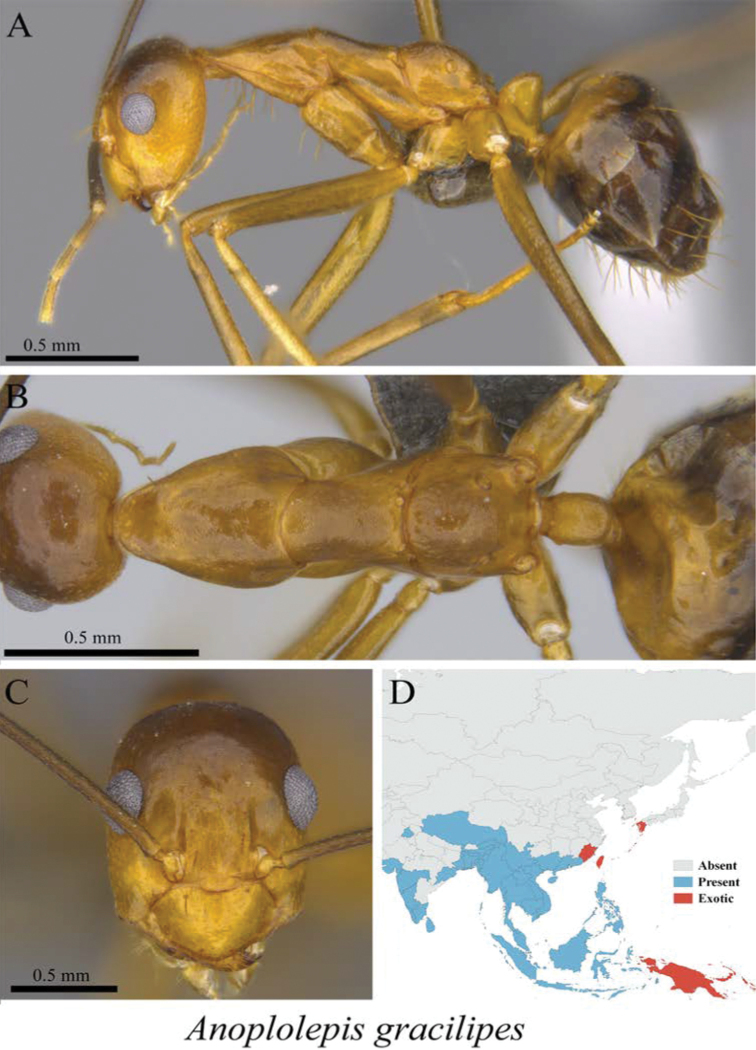
*Anoplolepis gracilipes* worker (MCZ-ENT00760060) **A** mesosoma in profile view **B** mesosoma in dorsal view **C** head in front view **D** global distribution map.

**Figure 20.**
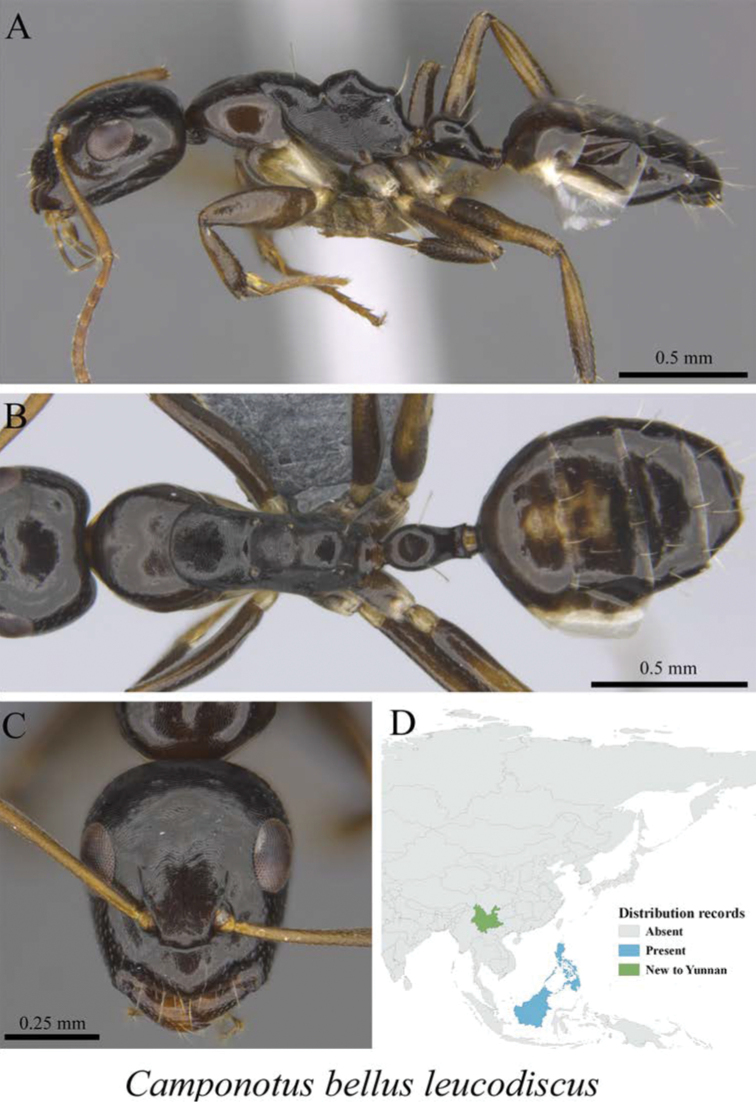
*Camponotus bellus leucodiscus* worker (MCZ-ENT00760068, new to China) **A** mesosoma in profile view **B** mesosoma in dorsal view **C** head in front view **D** global distribution map.

**Figure 21.**
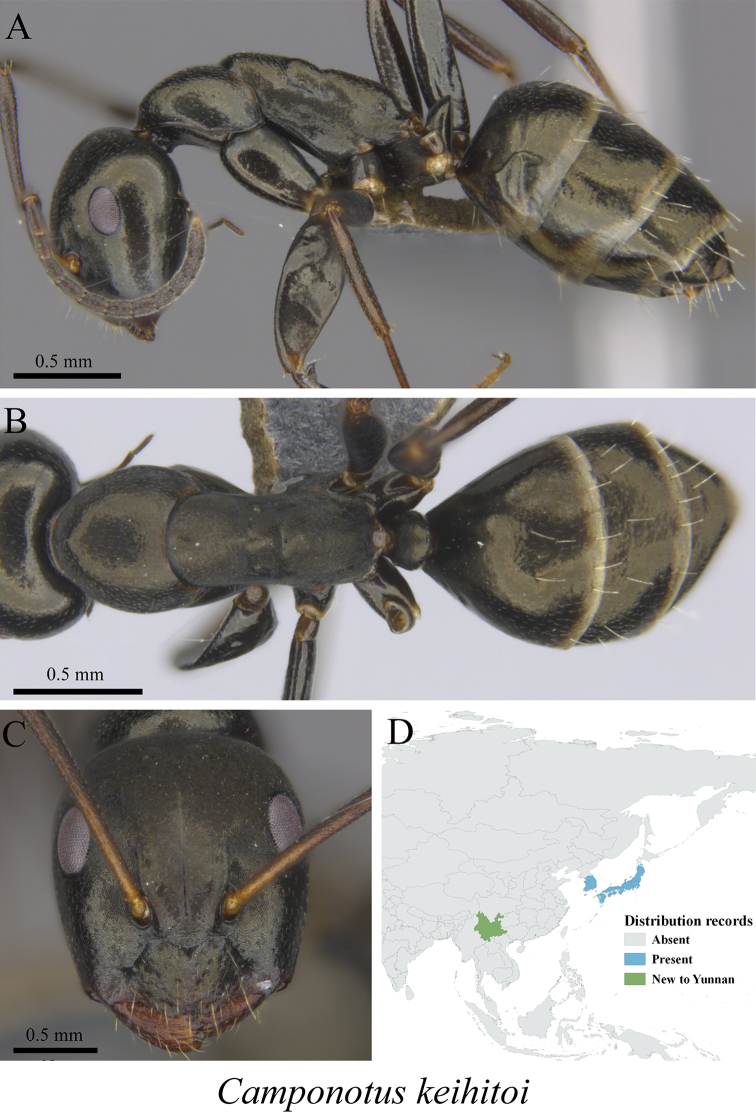
*Camponotus keihitoi* worker (MCZ-ENT00763692, new to China) **A** mesosoma in profile view **B** mesosoma in dorsal view **C** head in front view **D** global distribution map.

**Figure 22.**
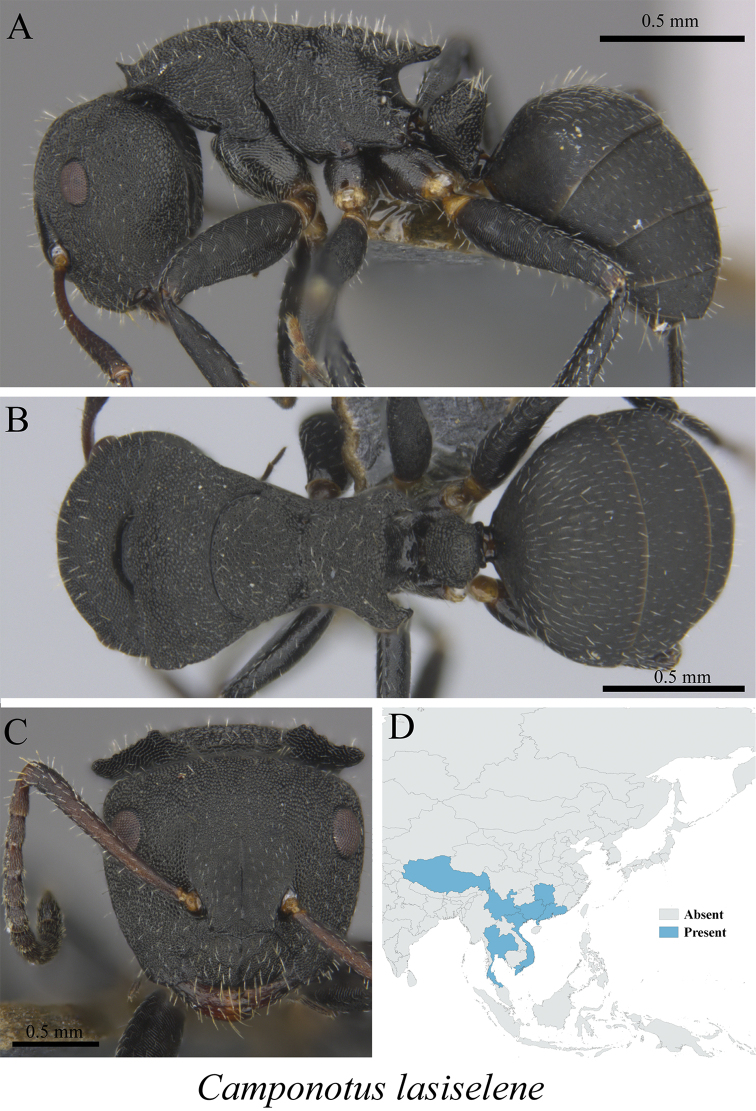
*Camponotus lasiselene* minor worker (MCZ-ENT00763190) **A** mesosoma in profile view **B** mesosoma in dorsal view **C** head in front view **D** global distribution map.

**Figure 23.**
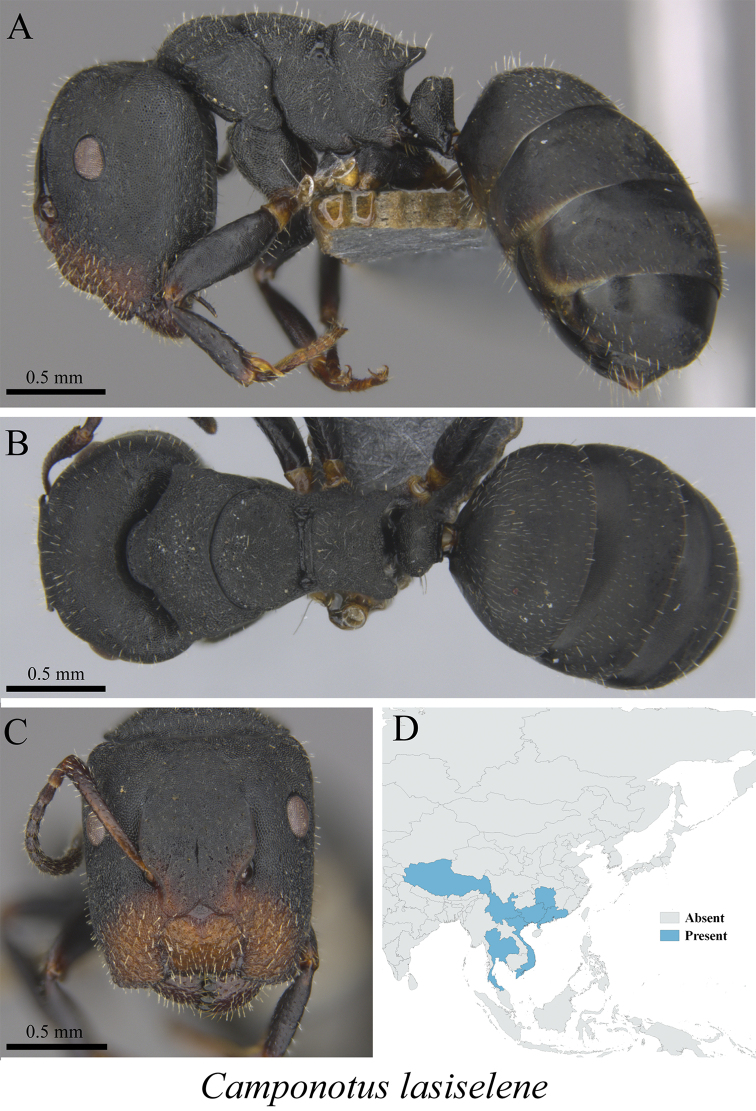
*Camponotus lasiselene* major worker (MCZ-ENT00763247) **A** mesosoma in profile view **B** mesosoma in dorsal view **C** head in front view **D** global distribution map.

**Figure 24.**
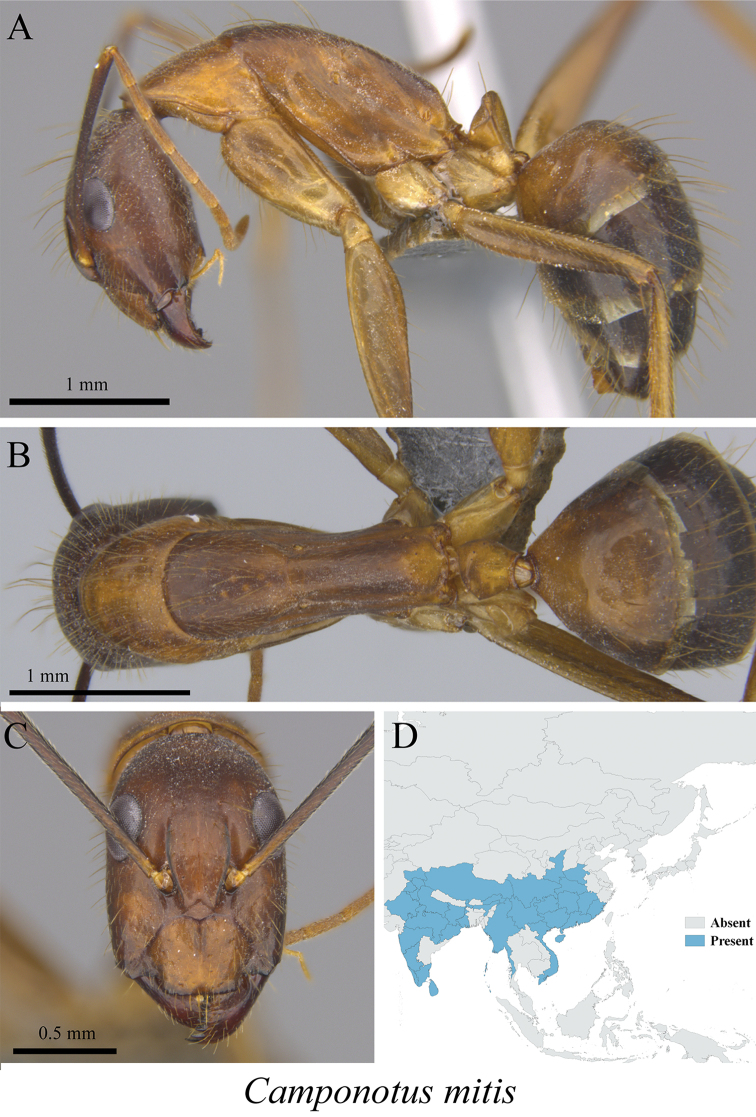
*Camponotus mitis* worker (MCZ-ENT00763213) **A** mesosoma in profile view **B** mesosoma in dorsal view **C** head in front view **D** global distribution map.

**Figure 25.**
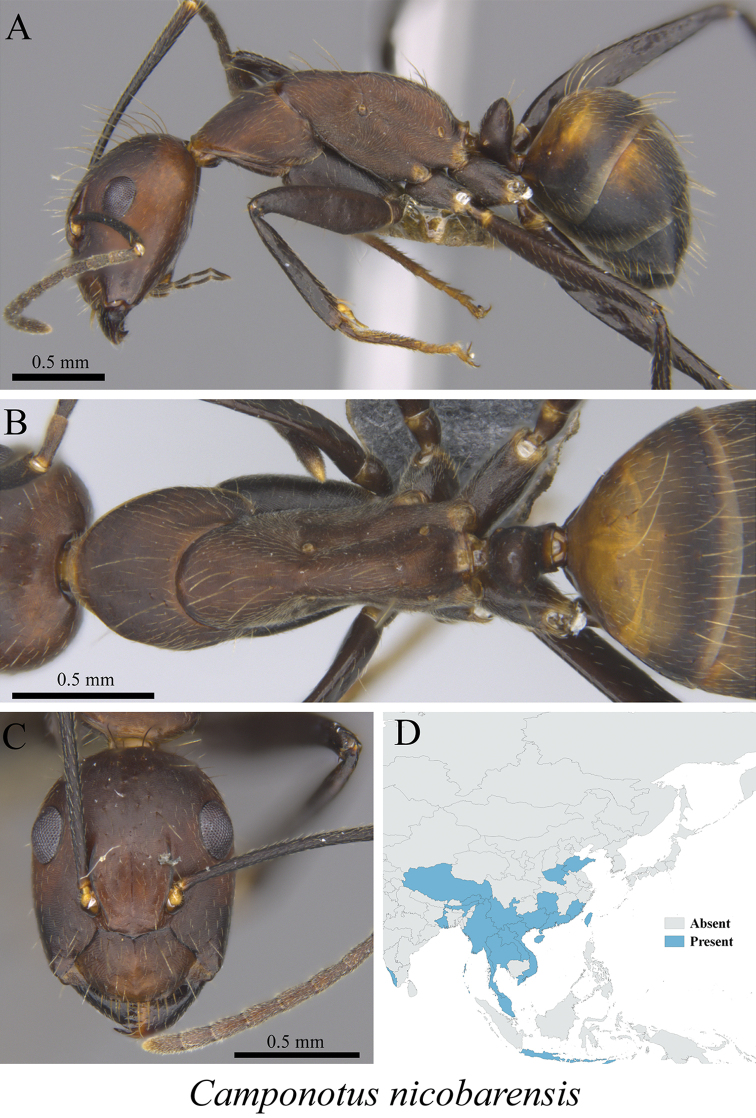
*Camponotus nicobarensis* worker (MCZ-ENT00763198) **A** mesosoma in profile view **B** mesosoma in dorsal view **C** head in front view **D** global distribution map.

**Figure 26.**
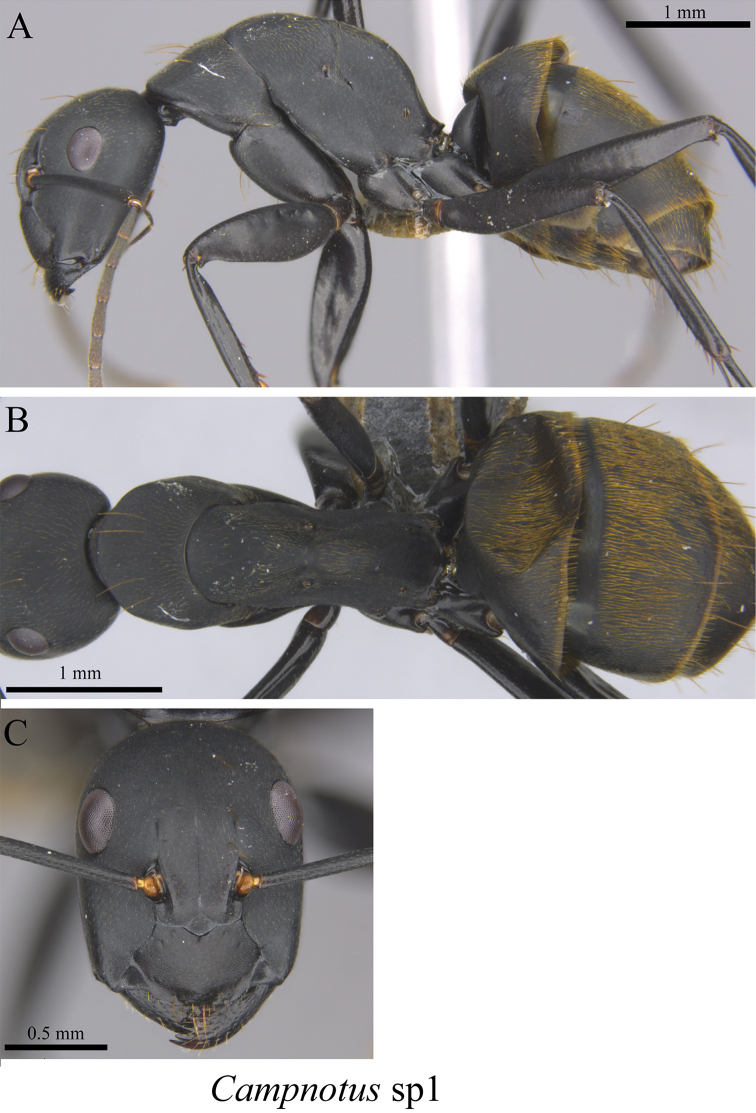
*Camponotus* sp. clm01 worker (MCZ-ENT00762843) **A** mesosoma in profile view **B** mesosoma in dorsal view **C** head in front view.

**Figure 27.**
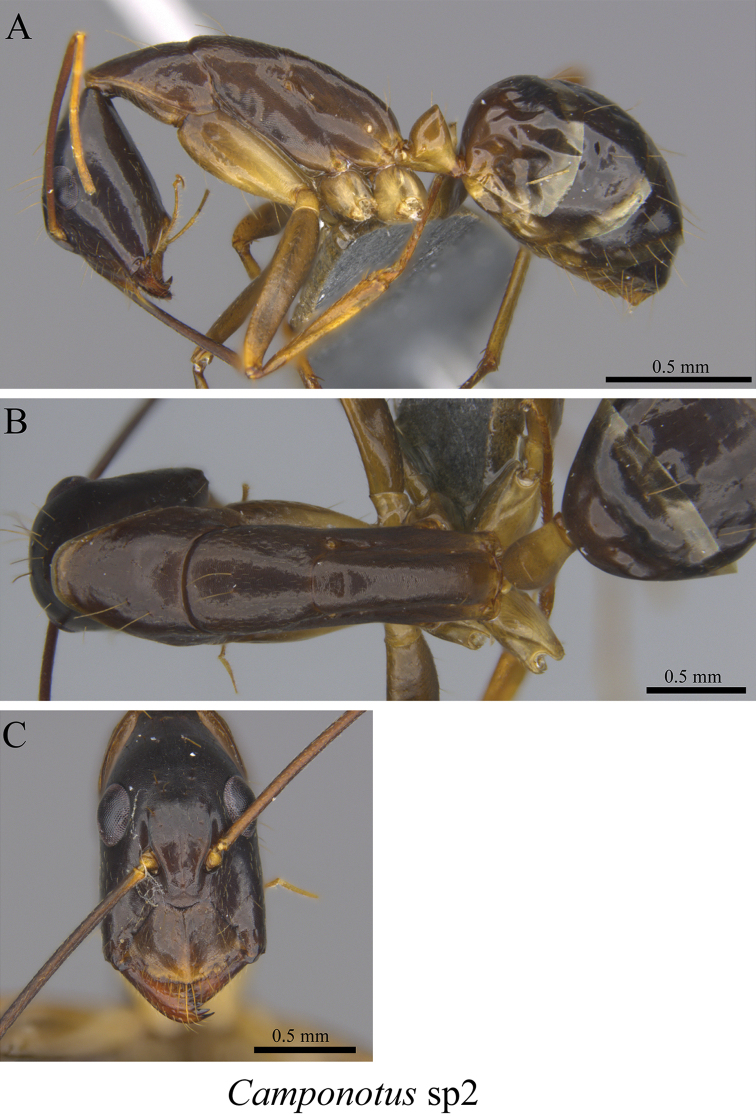
*Camponotus* sp. clm02 worker (MCZ-ENT00759861) **A** mesosoma in profile view **B** mesosoma in dorsal view **C** head in front view.

**Figure 28.**
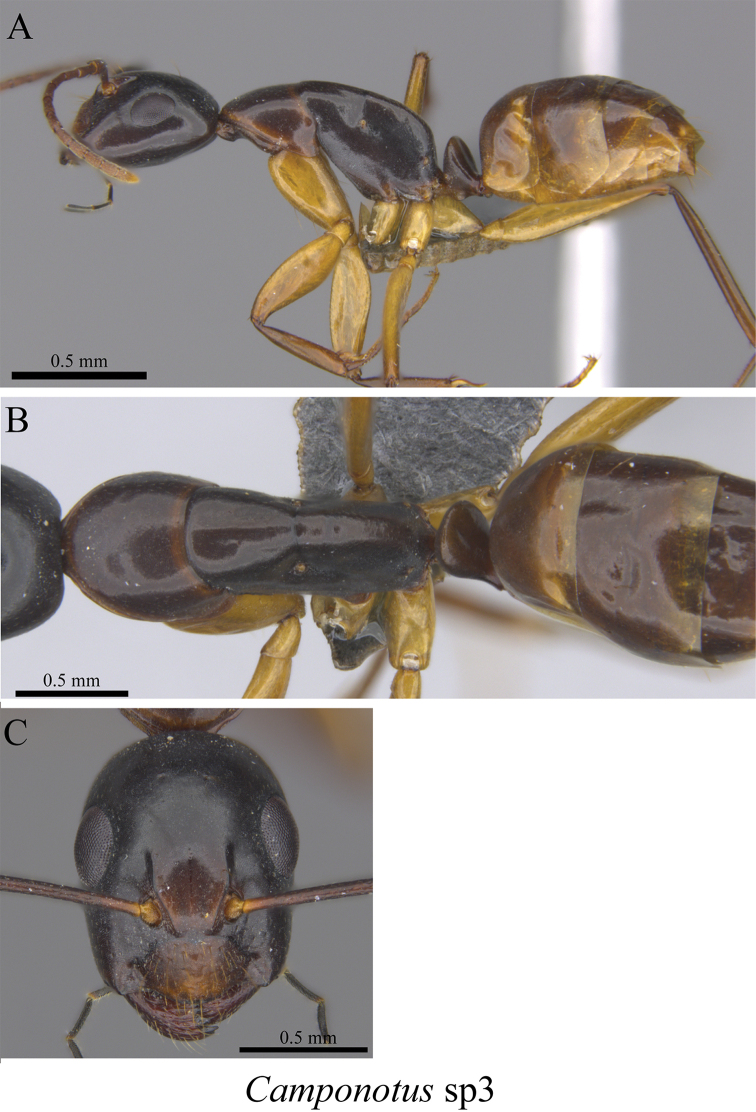
*Camponotus* sp. clm03 worker (MCZ-ENT00762821) **A** mesosoma in profile view **B** mesosoma in dorsal view **C** head in front view.

**Figure 29.**
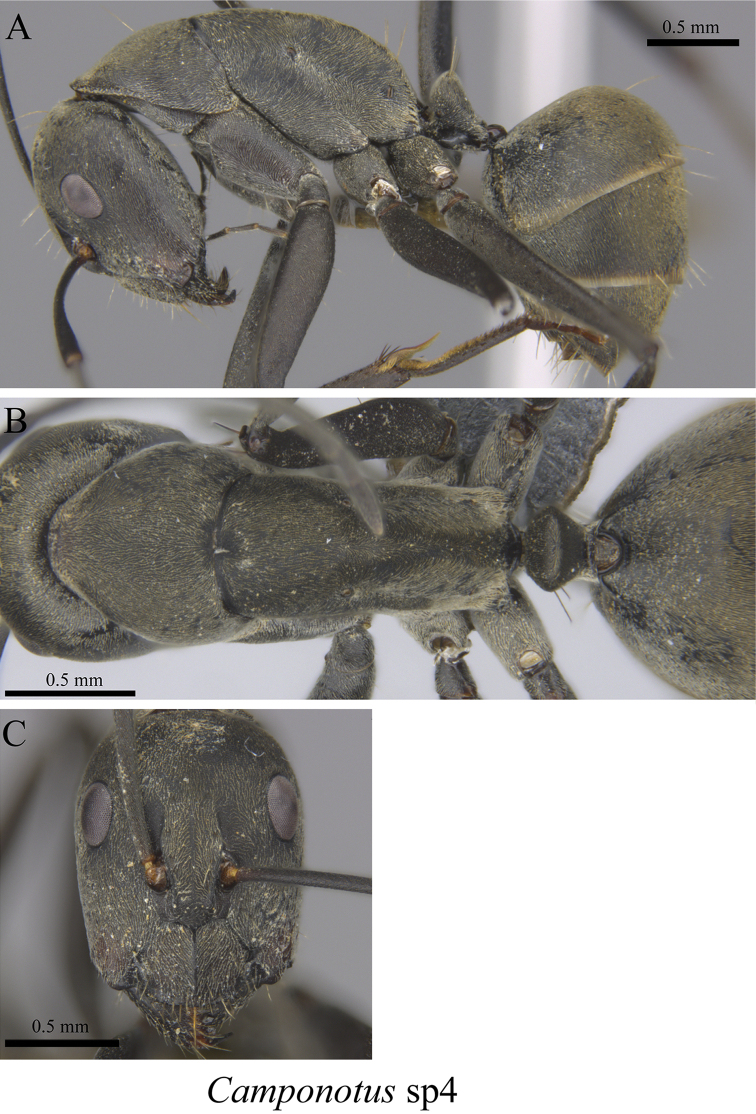
*Camponotus* sp. clm04 worker (MCZ-ENT00762978) **A** mesosoma in profile view **B** mesosoma in dorsal view **C** head in front view.

**Figure 30.**
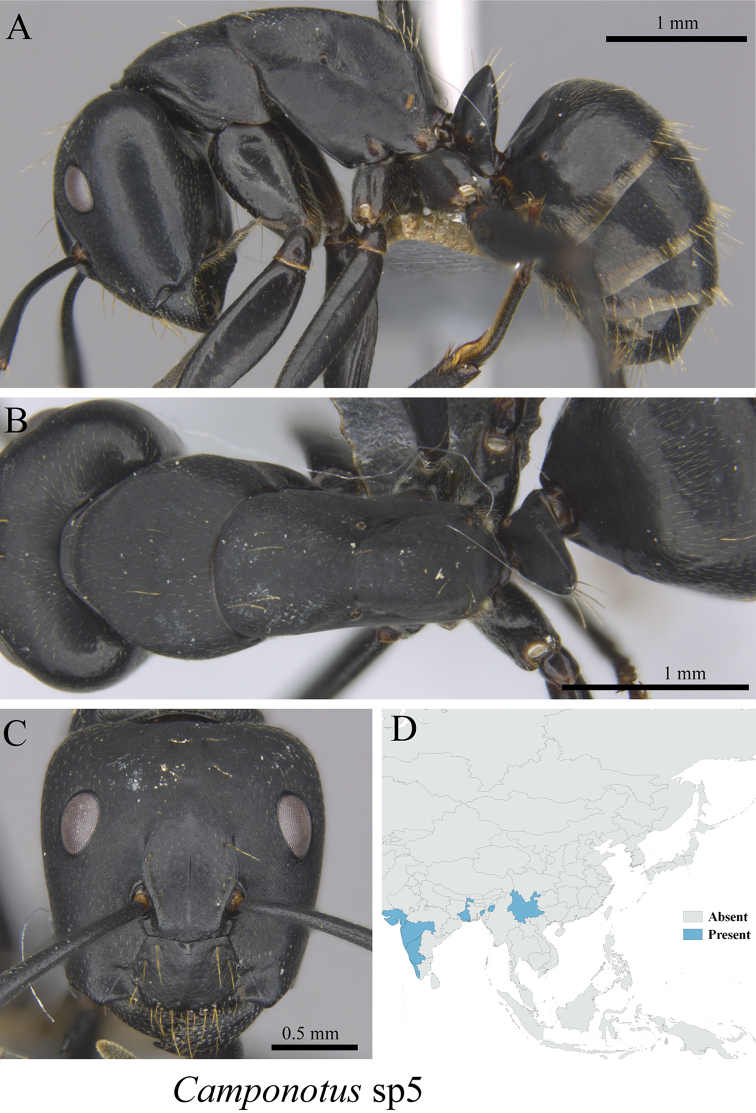
*Camponotus* sp. clm05 worker (MCZ-ENT00763312) **A** mesosoma in profile view **B** mesosoma in dorsal view **C** head in front view.

**Figure 31.**
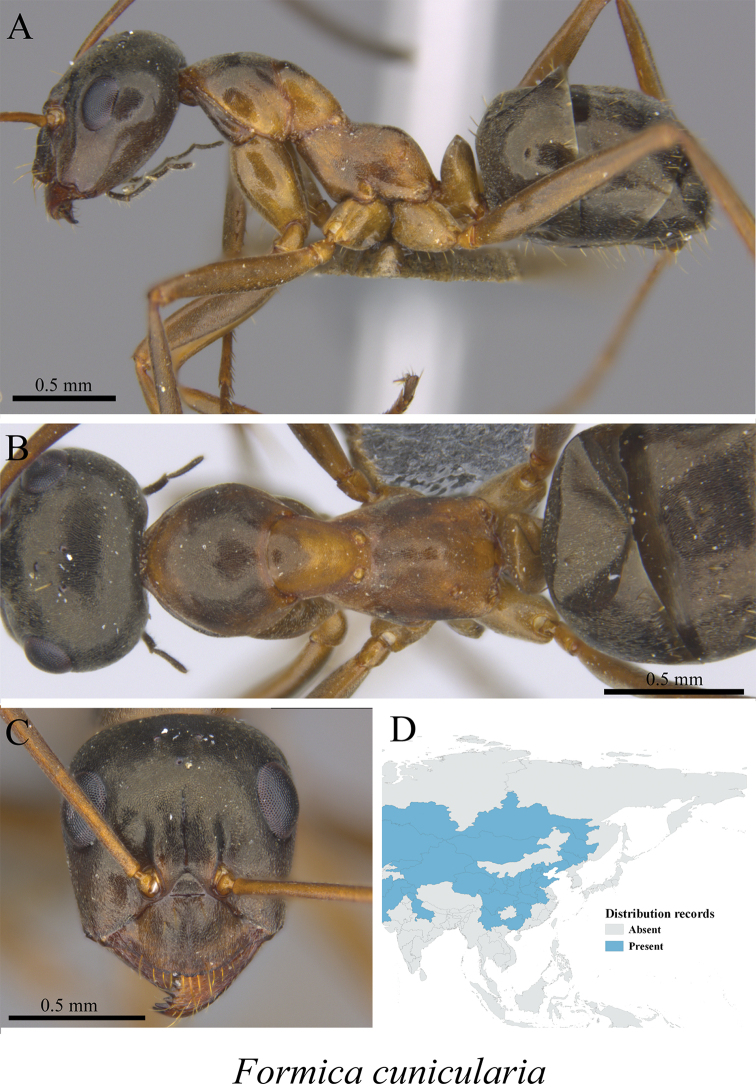
*Formica cunicularia* worker (MCZ-ENT00759967) **A** mesosoma in profile view **B** mesosoma in dorsal view **C** head in front view **D** global distribution map.

**Figure 32.**
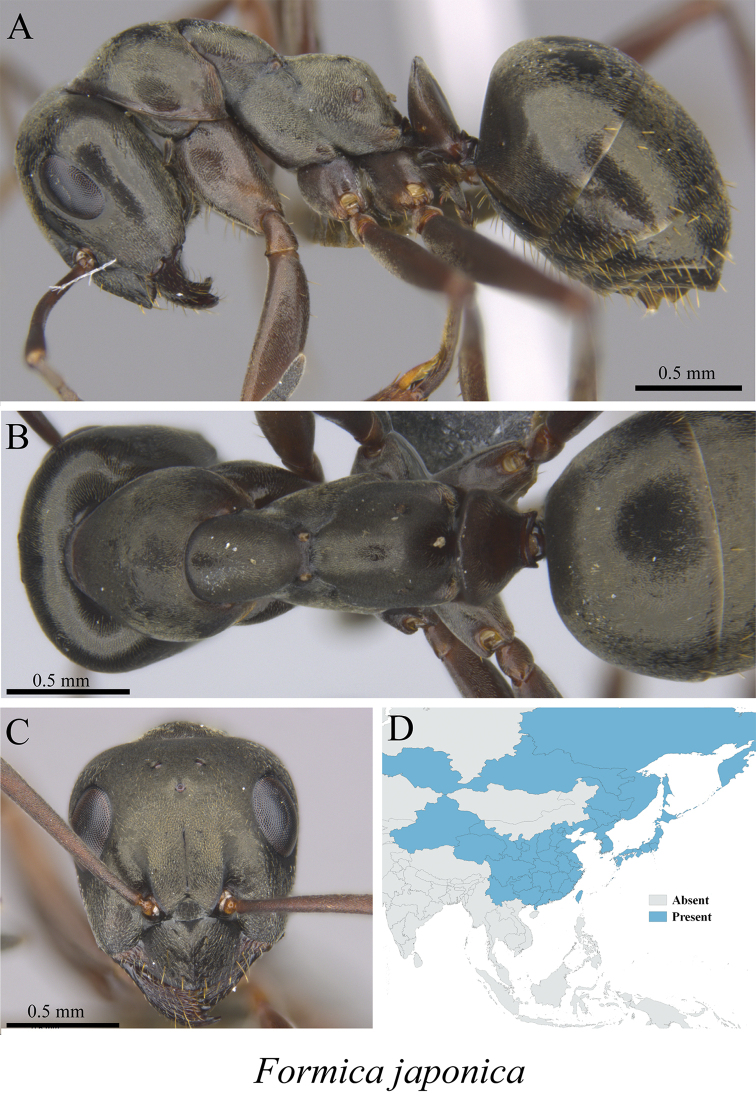
*Formica japonica* worker (MCZ-ENT00760066) **A** mesosoma in profile view **B** mesosoma in dorsal view **C** head in front view **D** global distribution map.

**Figure 33.**
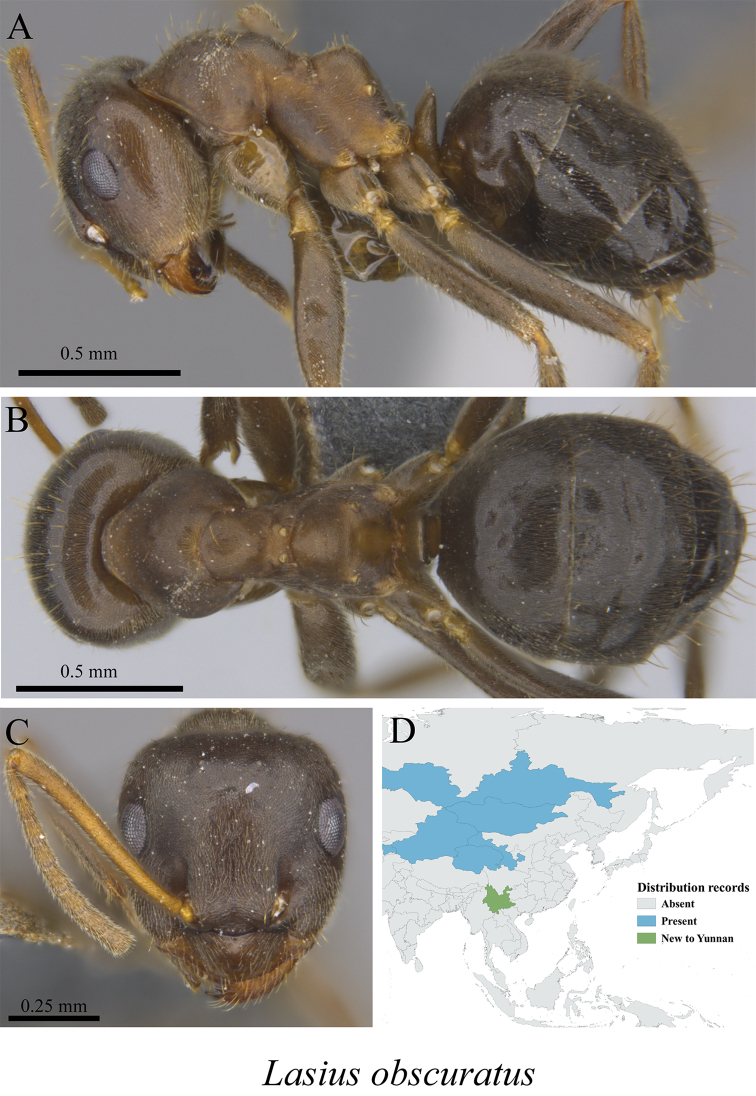
*Lasius obscuratus* worker (MCZ-ENT00760025, new to Yunnan) **A** mesosoma in profile view **B** mesosoma in dorsal view **C** head in front view **D** global distribution map.

**Figure 34.**
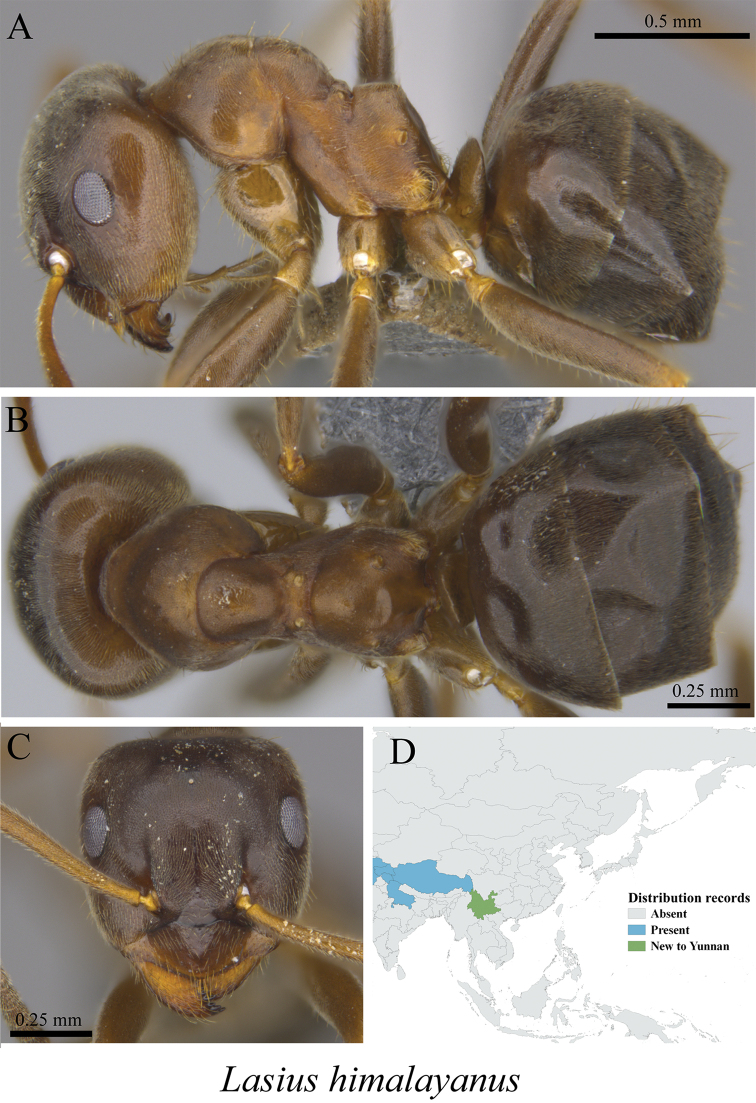
*Lasius himalayanus* worker (MCZ-ENT00763360, new to Yunnan) **A** mesosoma in profile view **B** mesosoma in dorsal view **C** head in front view **D** global distribution map.

**Figure 35.**
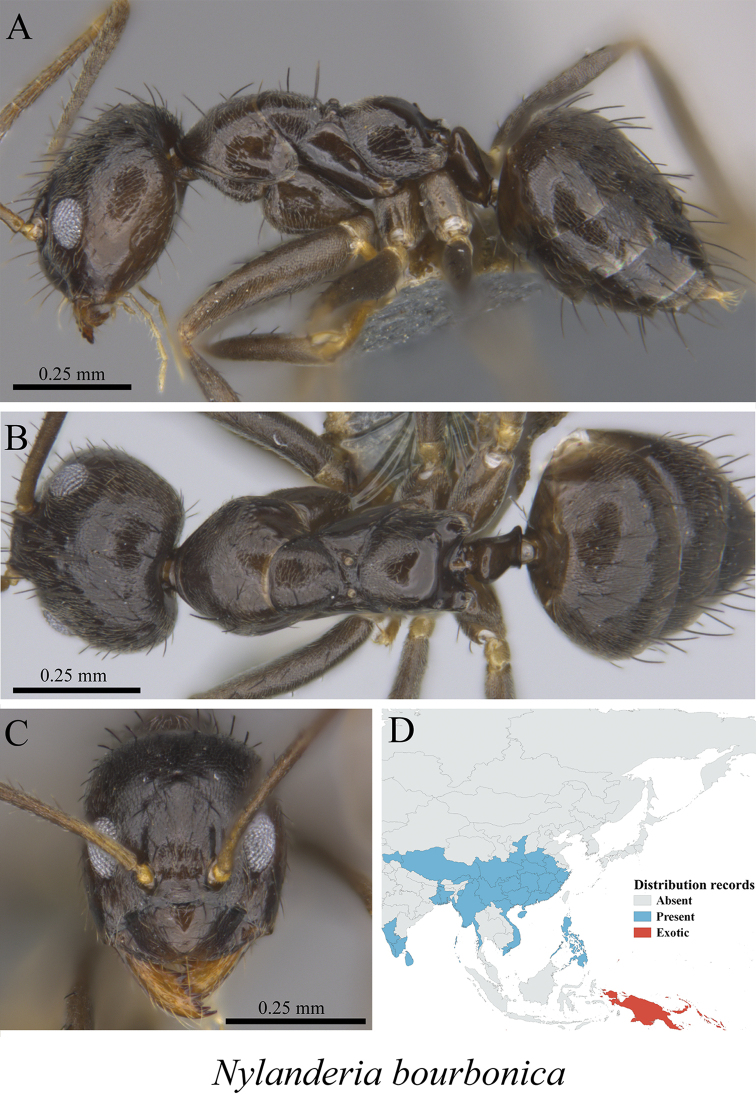
*Nylanderia bourbonica* worker (MCZ-ENT00760019) **A** mesosoma in profile view **B** mesosoma in dorsal view **C** head in front view **D** global distribution map.

**Figure 36.**
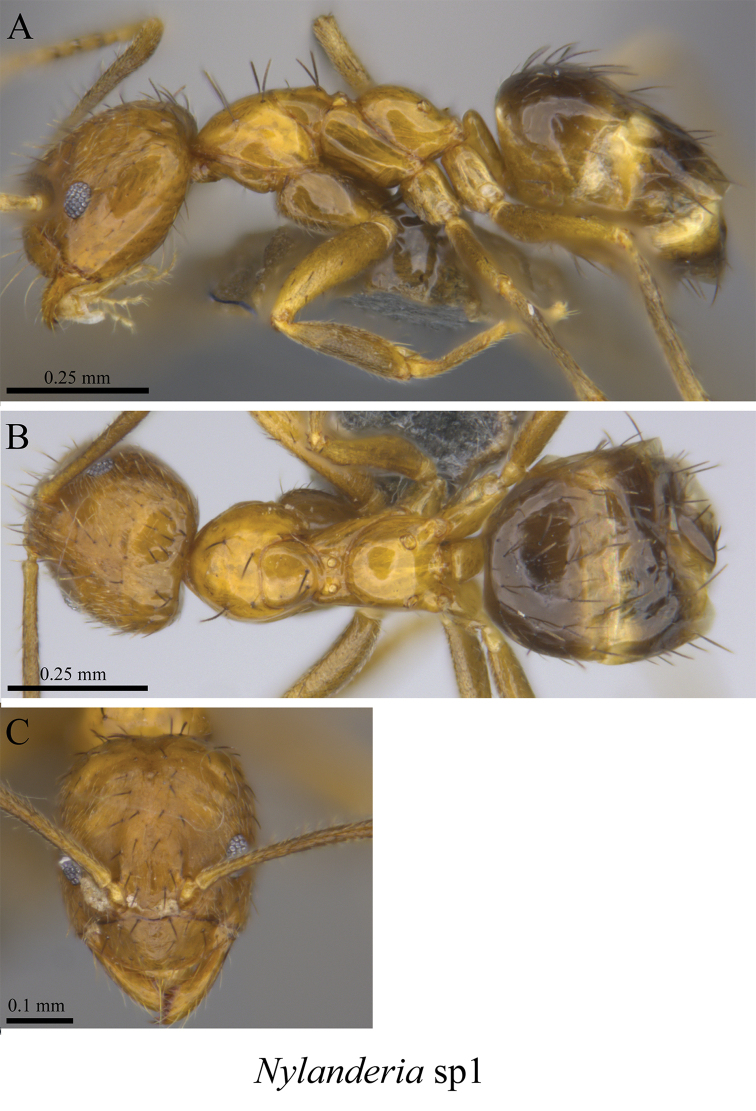
*Nylanderia* sp. clm01 worker (MCZ-ENT00759776) **A** mesosoma in profile view **B** mesosoma in dorsal view **C** head in front view **D** global distribution map.

**Figure 37.**
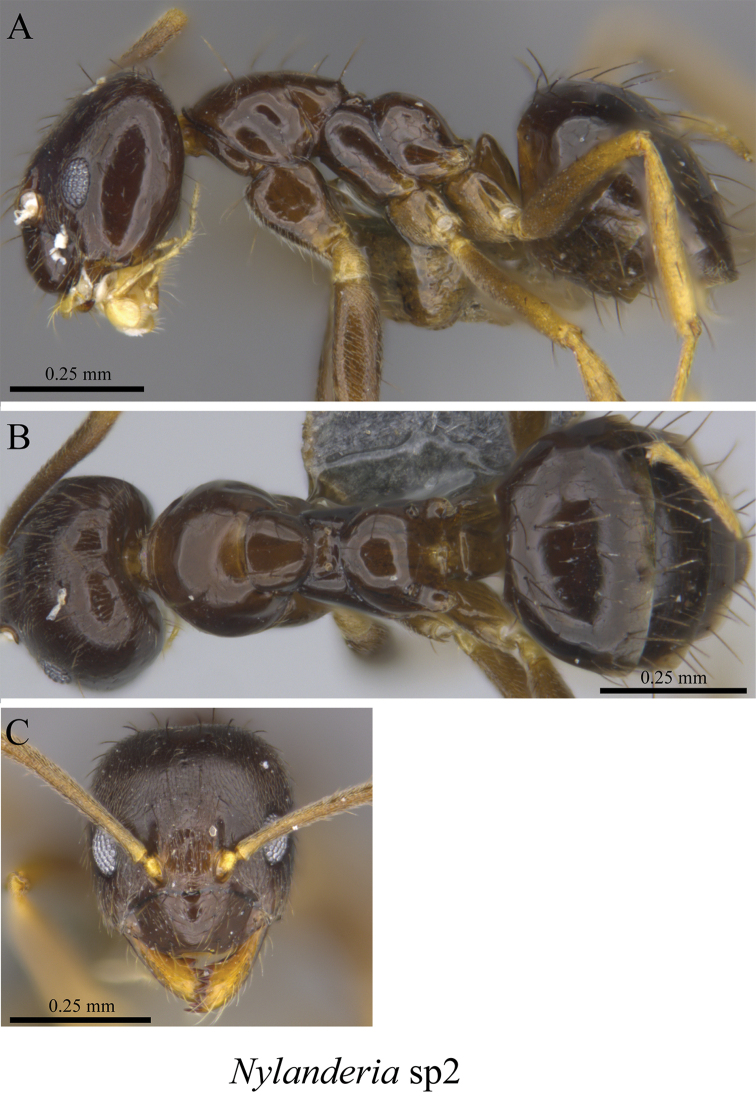
*Nylanderia* sp. clm02 worker (MCZ-ENT00759968) **A** mesosoma in profile view **B** mesosoma in dorsal view **C** head in front view **D** global distribution map.

**Figure 38.**
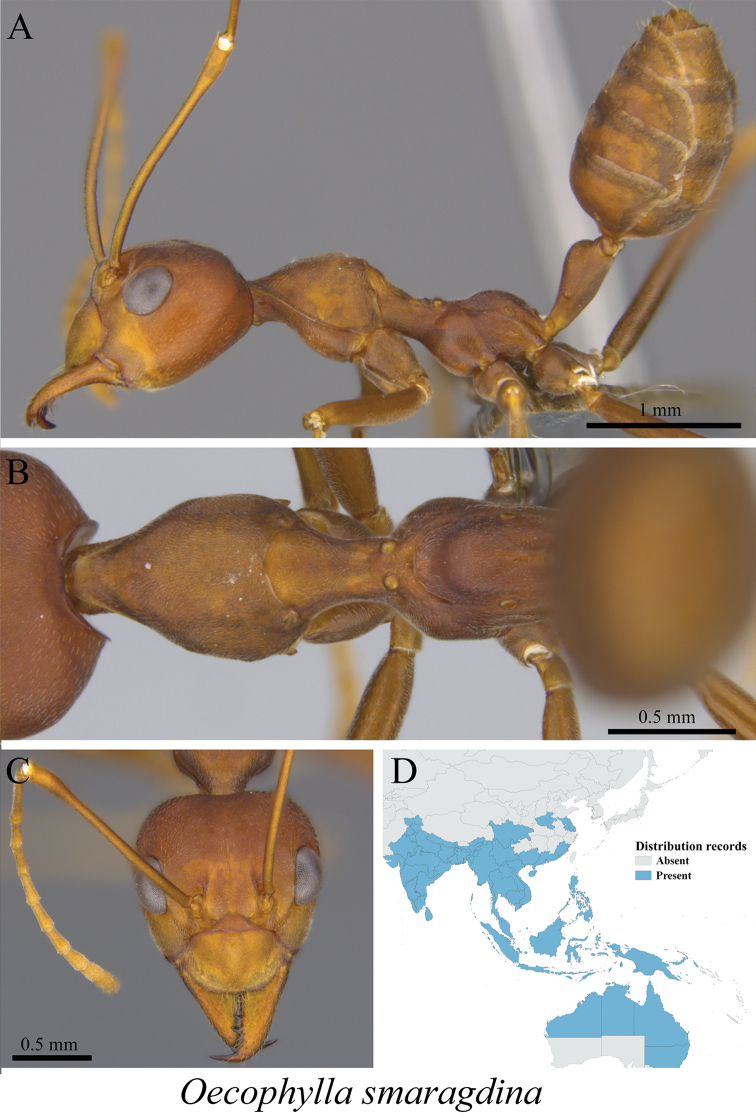
*Oecophylla smaragdina* worker (MCZ-ENT00763551) **A** mesosoma in profile view **B** mesosoma in dorsal view **C** head in front view **D** global distribution map.

**Figure 39.**
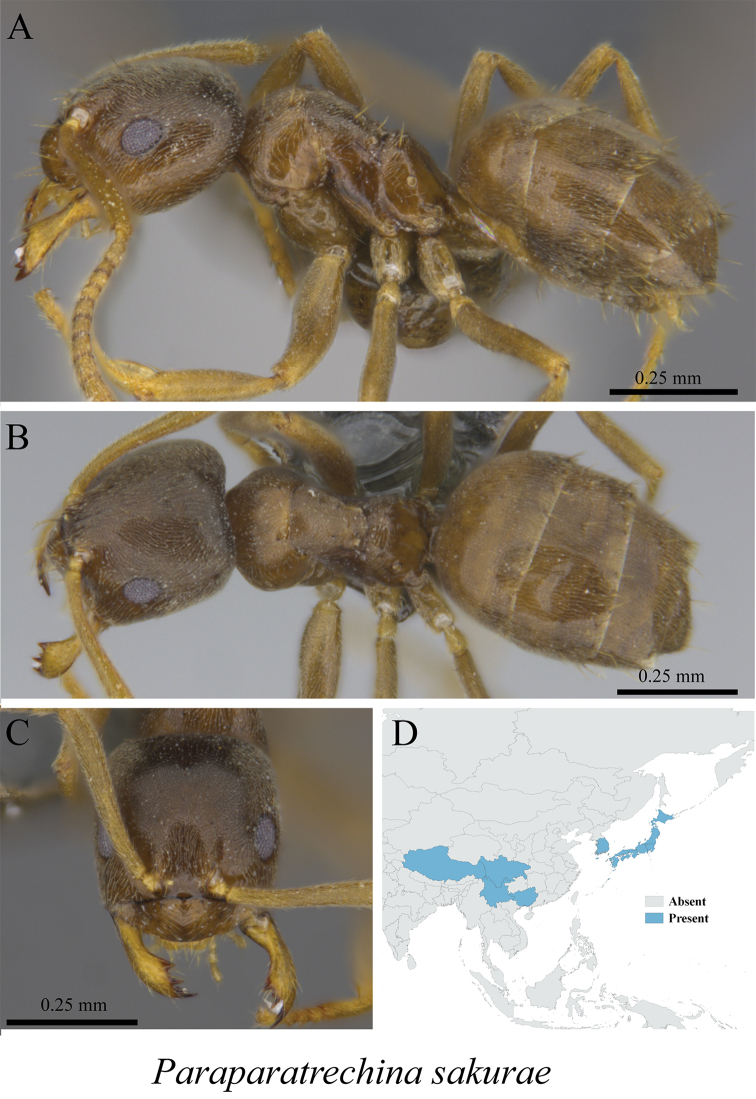
*Paraparatrechina sakurae* worker (MCZ-ENT00759953) **A** mesosoma in profile view **B** mesosoma in dorsal view **C** head in front view **D** global distribution map.

**Figure 40.**
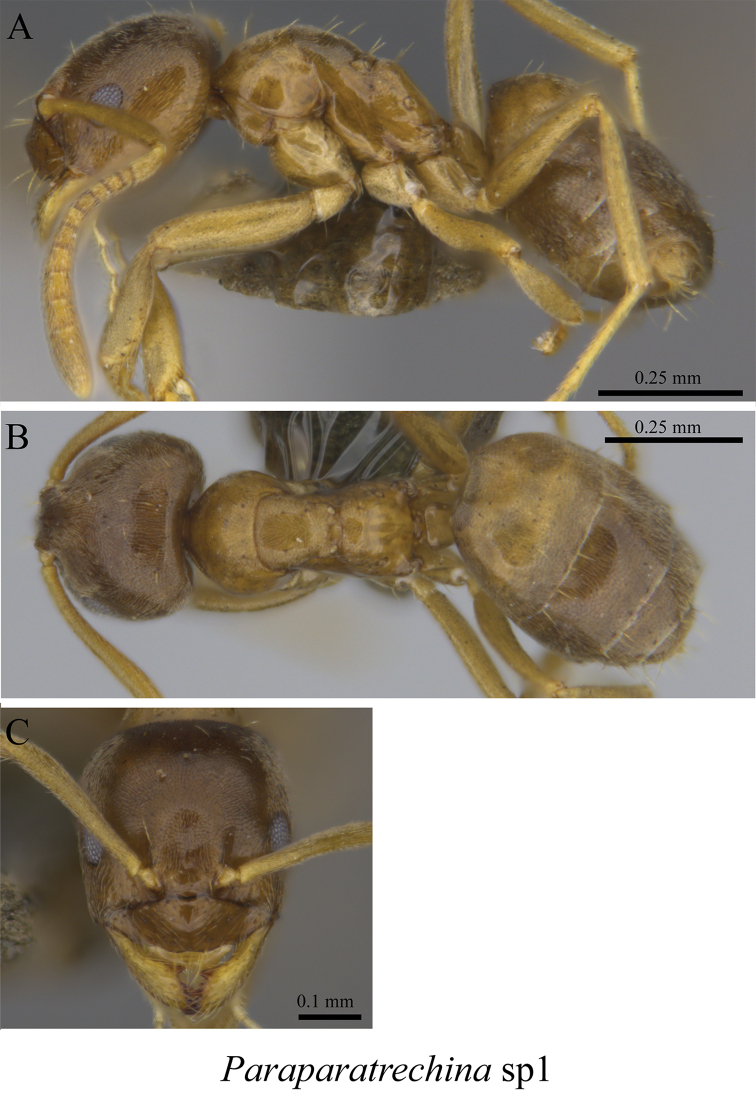
*Paraparatrechina* sp. clm01 worker (MCZ-ENT00763500) **A** mesosoma in profile view **B** mesosoma in dorsal view **C** head in front view **D** global distribution map.

**Figure 41.**
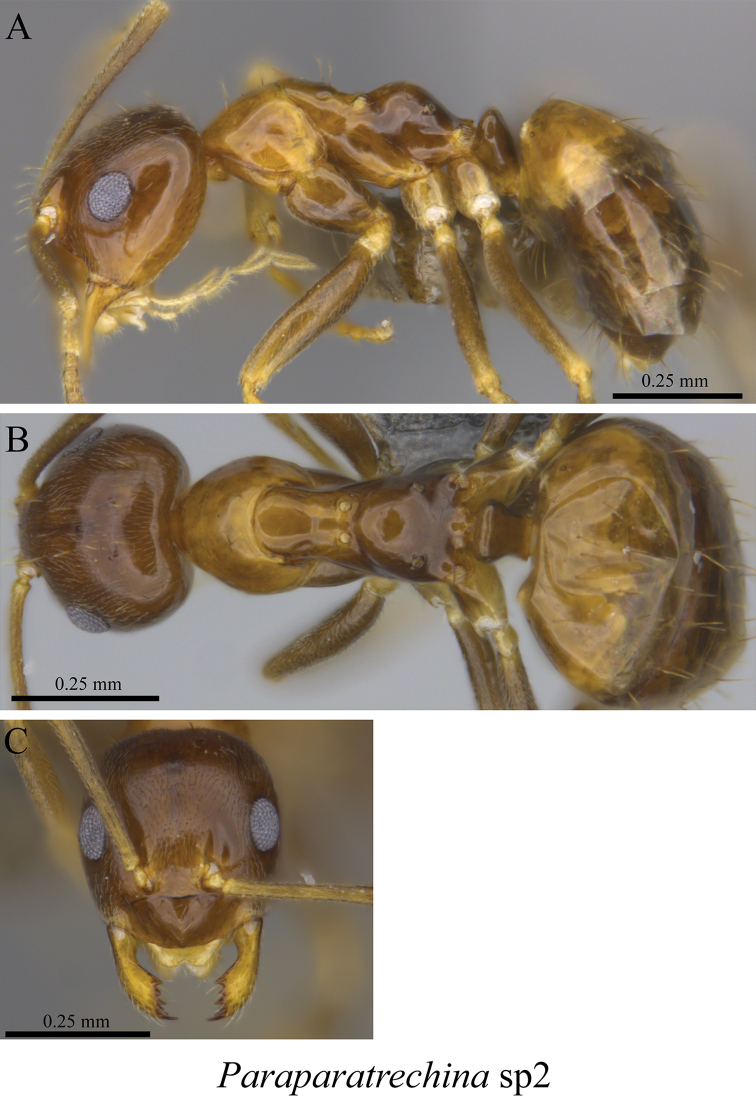
*Paraparatrechina* sp. clm02 worker (MCZ-ENT00763427) **A** mesosoma in profile view **B** mesosoma in dorsal view **C** head in front view **D** global distribution map.

**Figure 42.**
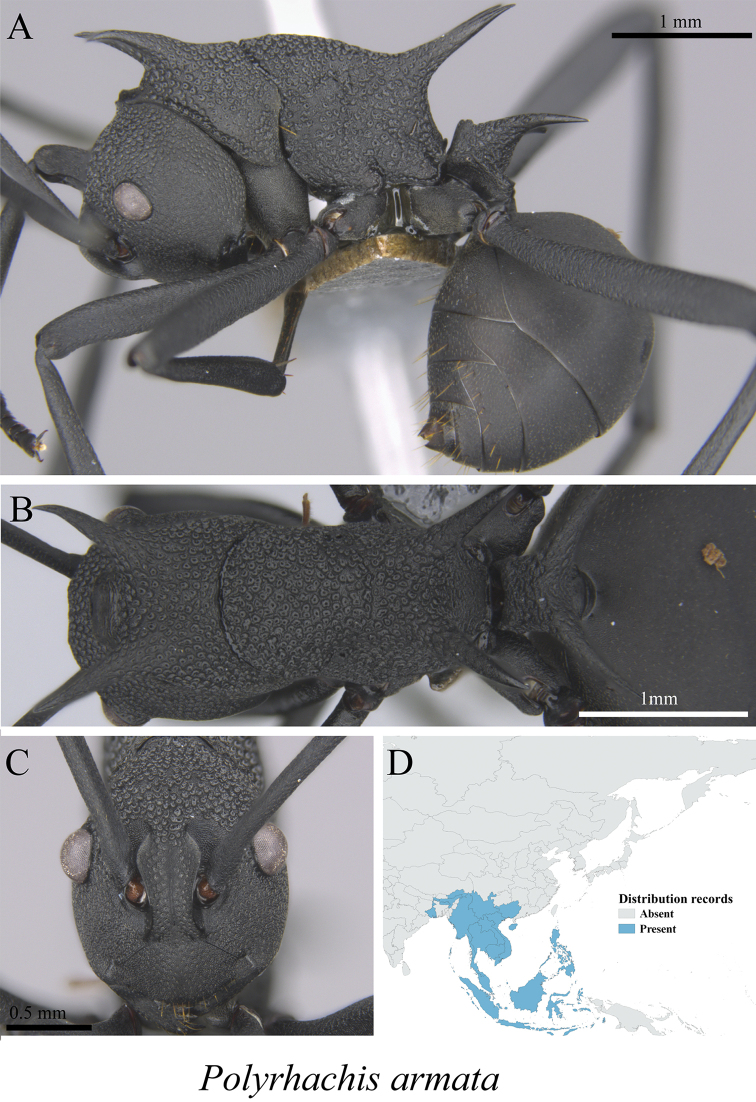
*Polyrhachis armata* worker (MCZ-ENT00763282) **A** mesosoma in profile view **B** mesosoma in dorsal view **C** head in front view **D** global distribution map.

**Figure 43.**
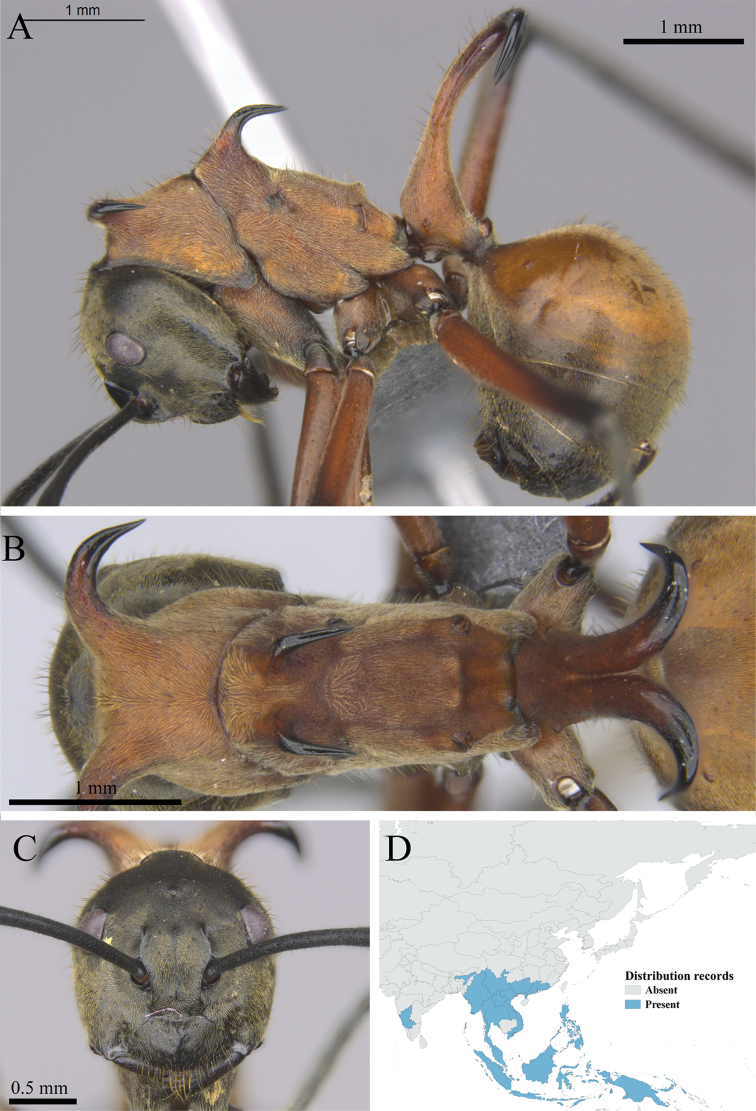
*Polyrhachis bihamata* worker (MCZ-ENT00763176). **A** mesosoma in profile view **B** mesosoma in dorsal view **C** head in front view **D** global distribution map.

**Figure 44.**
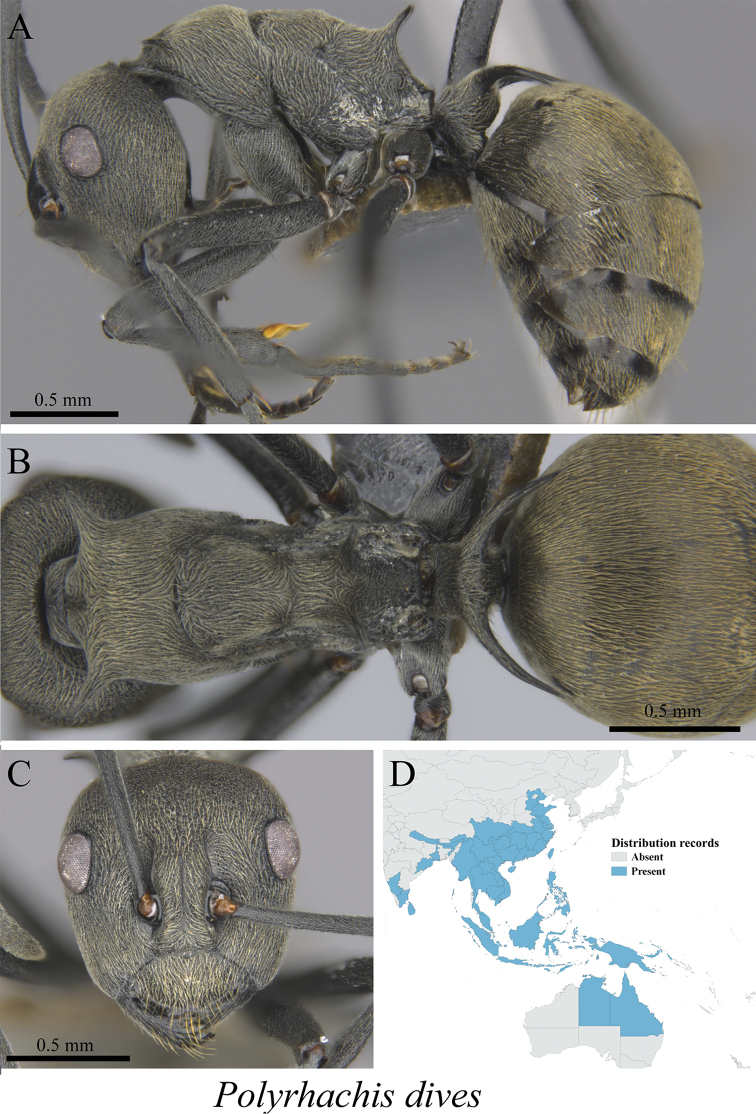
*Polyrhachis dives* worker (MCZ-ENT00760042). **A** mesosoma in profile view **B** mesosoma in dorsal view **C** head in front view **D** global distribution map.

**Figure 45.**
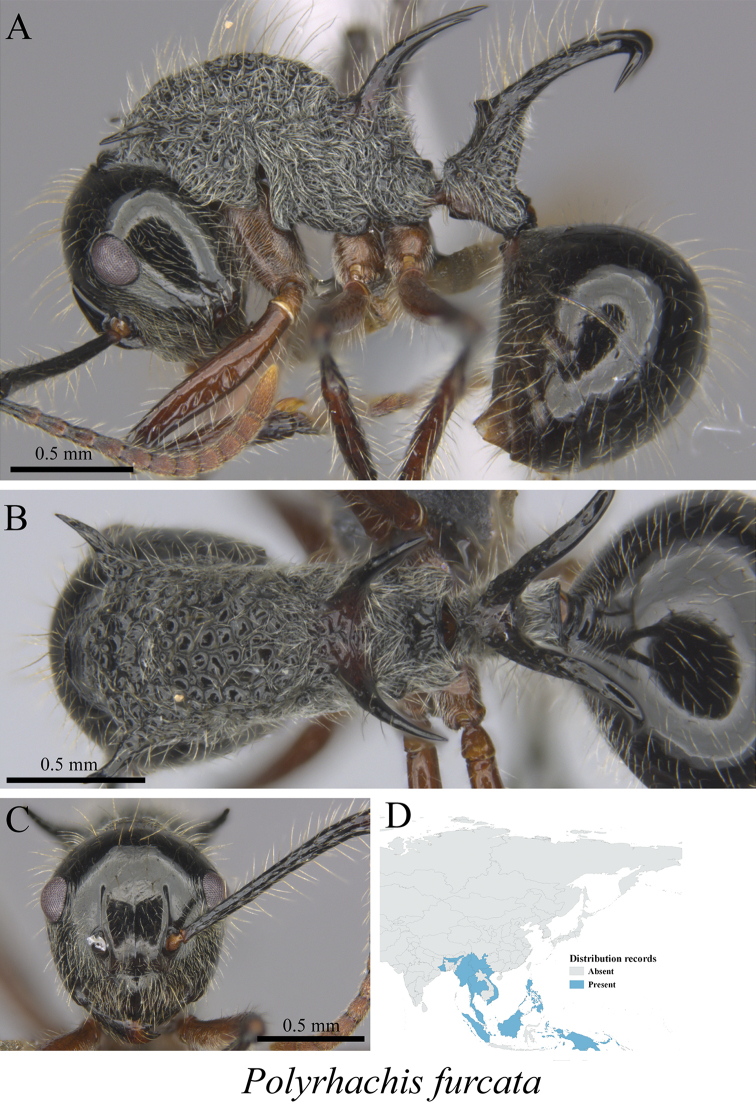
*Polyrhachis furcata* worker (MCZ-ENT00763549) **A** mesosoma in profile view **B** mesosoma in dorsal view **C** head in front view **D** global distribution map.

**Figure 46.**
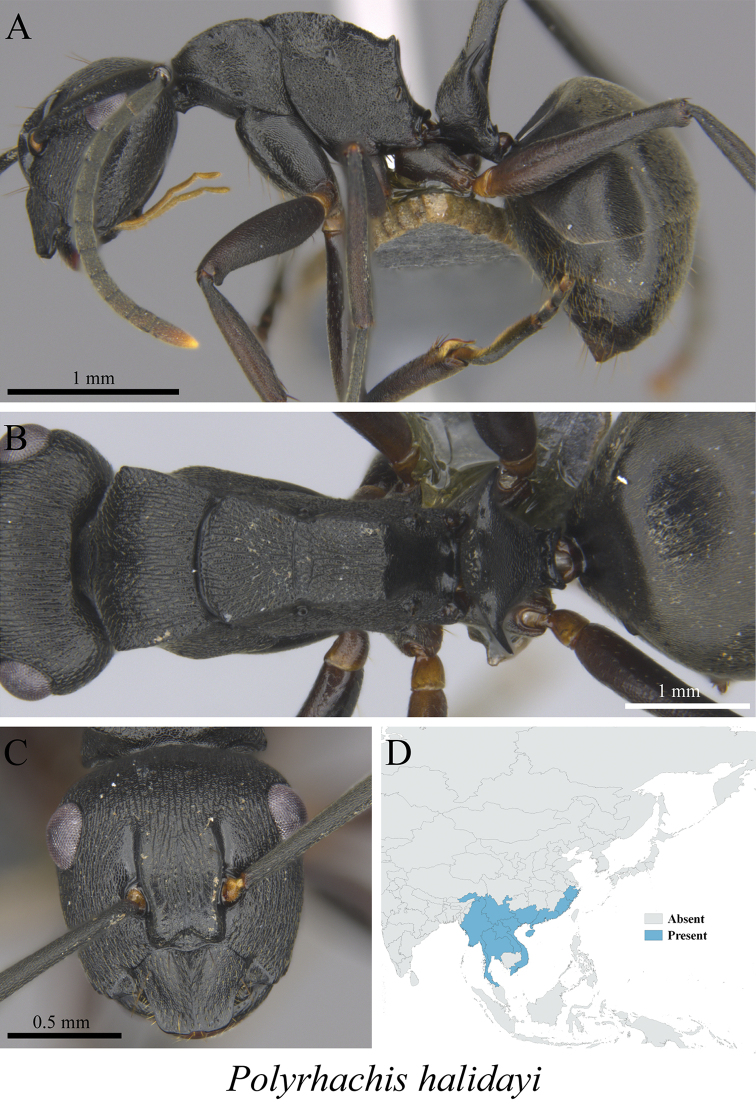
*Polyrhachis halidayi* worker (MCZ-ENT00763195) **A** mesosoma in profile view **B** mesosoma in dorsal view **C** head in front view **D** global distribution map.

**Figure 47.**
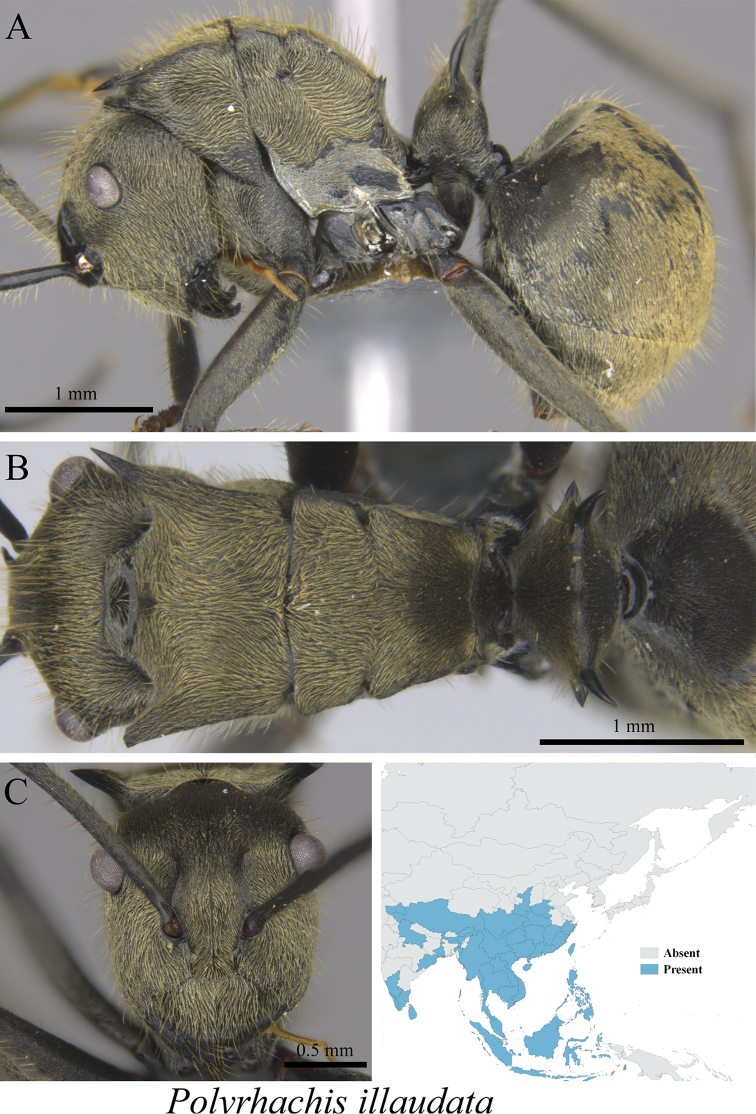
*Polyrhachis illaudata* worker (MCZ-ENT00760071) **A** mesosoma in profile view **B** mesosoma in dorsal view **C** head in front view **D** global distribution map.

**Figure 48.**
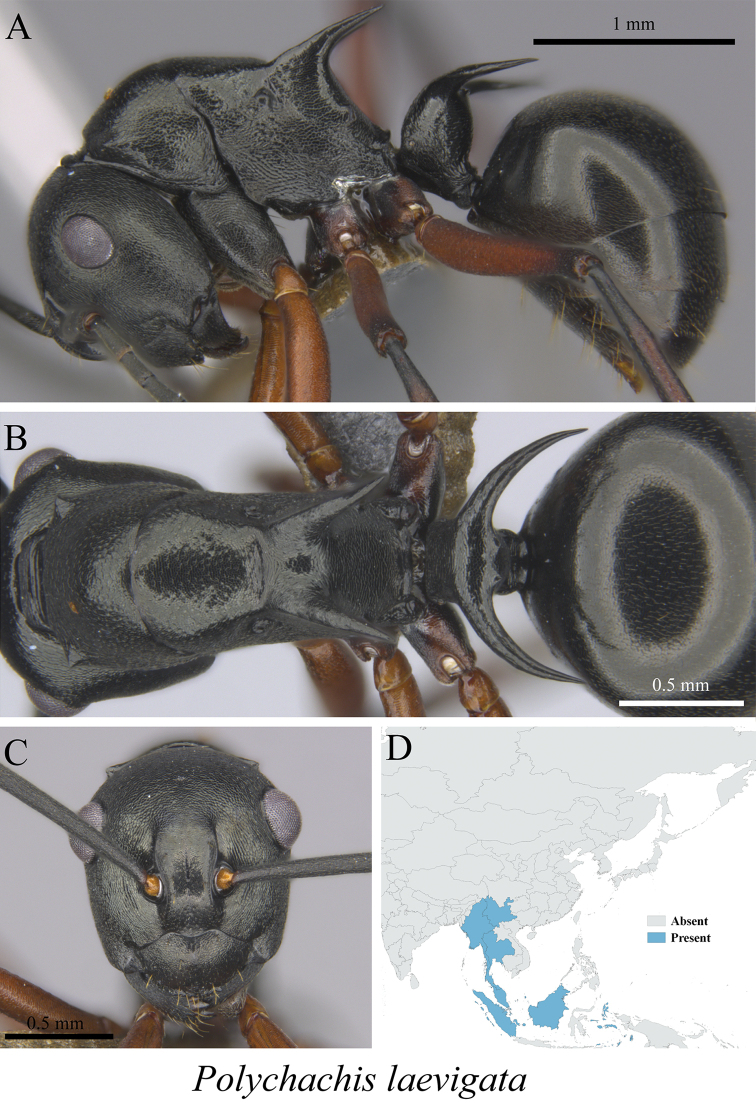
*Polyrhachis laevigata* worker (MCZ-ENT00763568) **A** mesosoma in profile view **B** mesosoma in dorsal view **C** head in front view **D** global distribution map.

**Figure 49.**
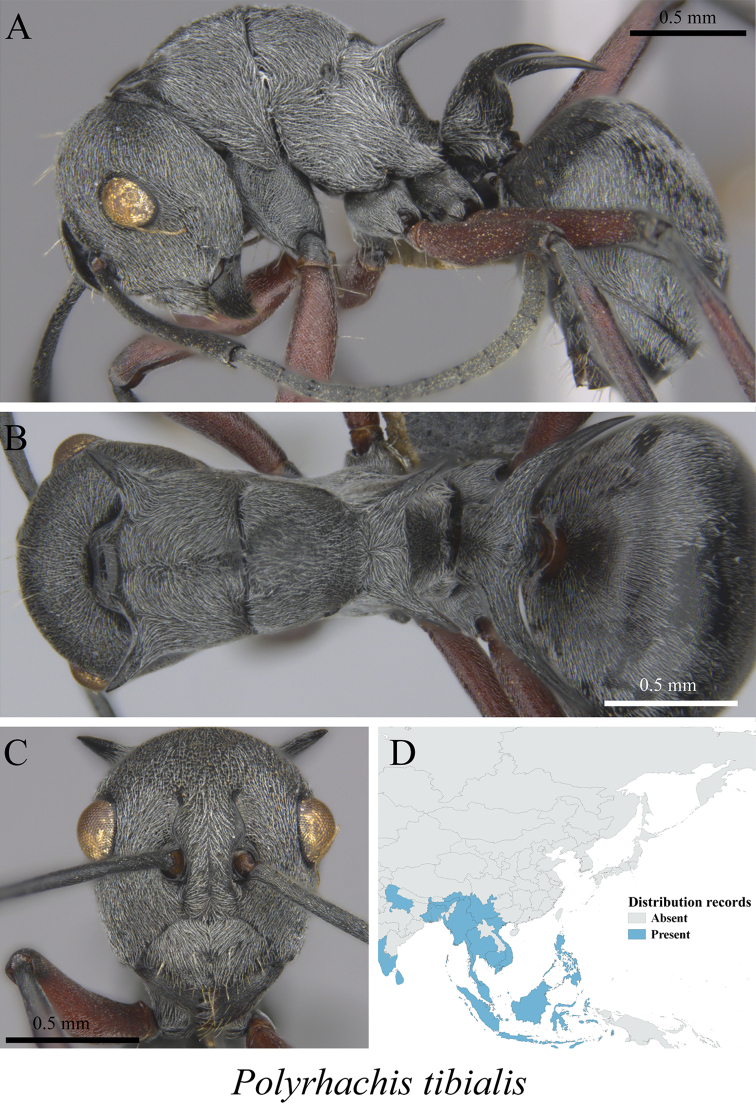
*Polyrhachis tibialis* worker (MCZ-ENT00763284). **A** mesosoma in profile view **B** mesosoma in dorsal view **C** head in front view **D** global distribution map.

**Figure 50.**
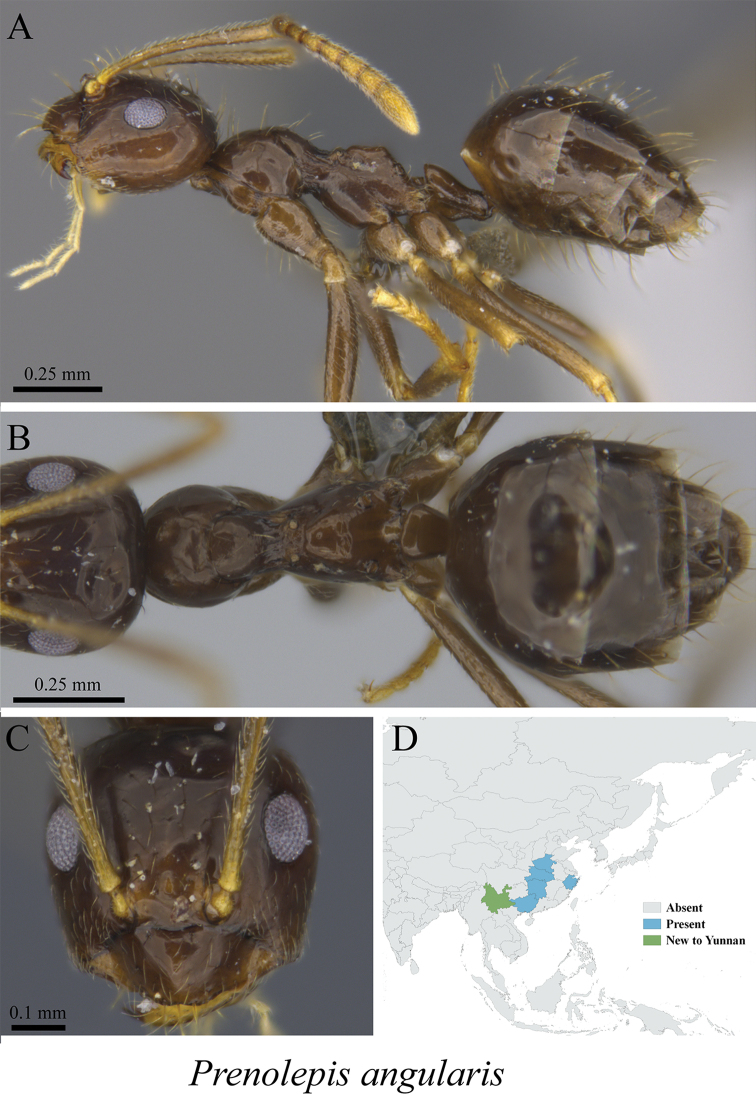
*Prenolepis angularis* worker (MCZ-ENT00763328, new to Yunnan). **A** mesosoma in profile view **B** mesosoma in dorsal view **C** head in front view **D** global distribution map.

**Figure 51.**
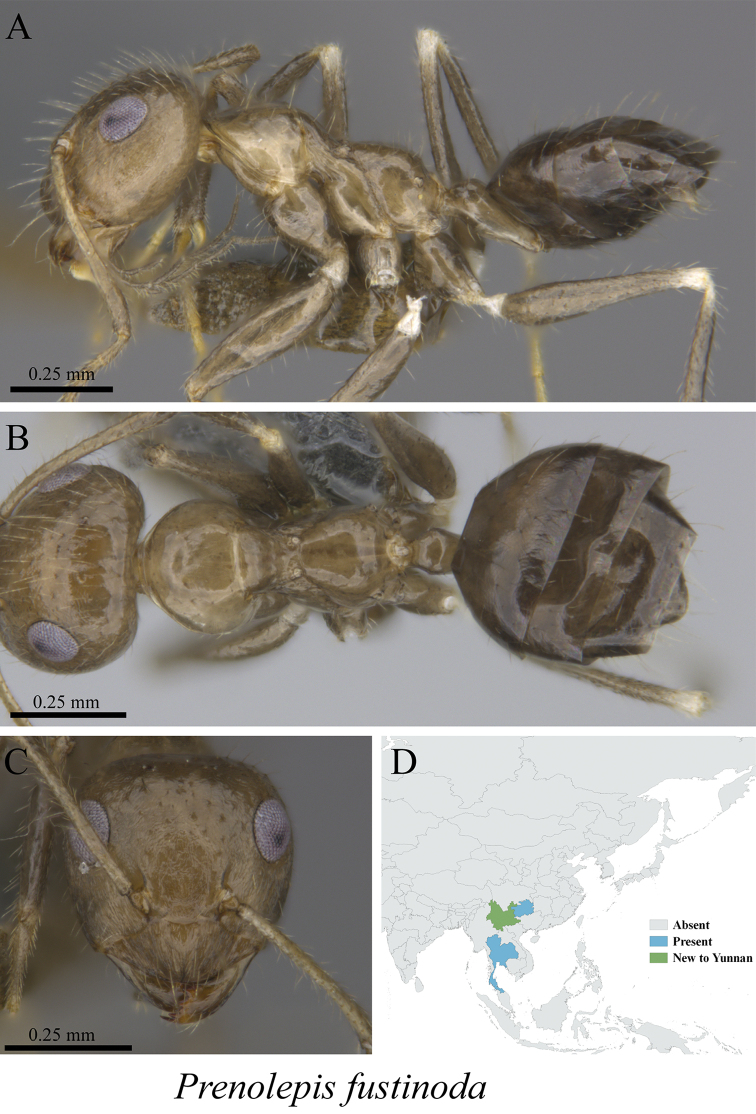
*Prenolepis fustinoda* worker (MCZ-ENT00763200, new to Yunnan) **A** mesosoma in profile view **B** mesosoma in dorsal view **C** head in front view **D** global distribution map.

**Figure 52.**
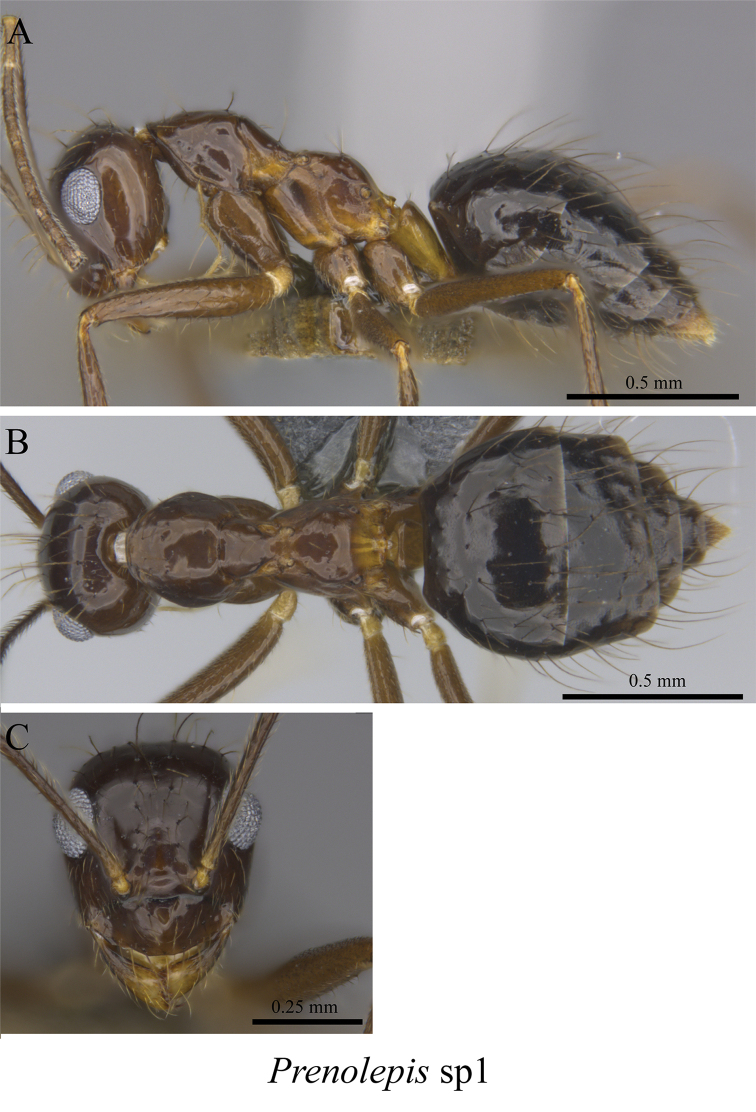
*Prenolepis* sp. clm01 worker (MCZ-ENT00763220) **A** mesosoma in profile view **B** mesosoma in dorsal view **C** head in front view.

**Figure 53.**
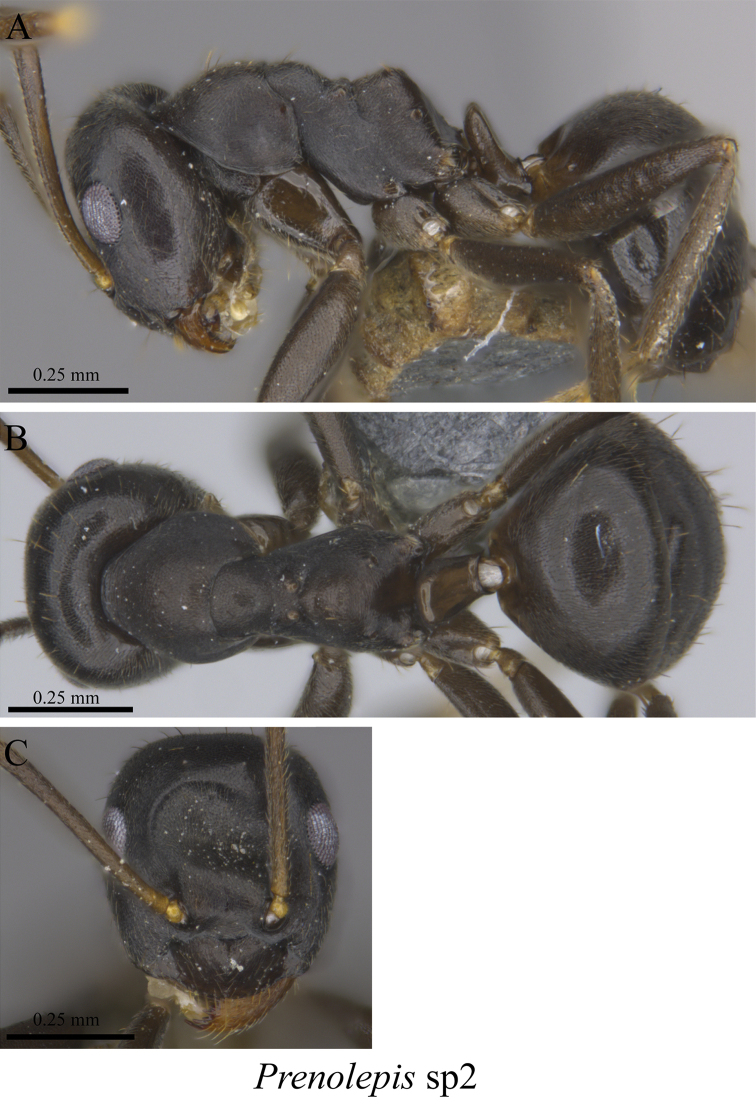
*Prenolepis* sp. clm02 worker (MCZ-ENT00763467) **A** mesosoma in profile view **B** mesosoma in dorsal view **C** head in front view.

**Figure 54.**
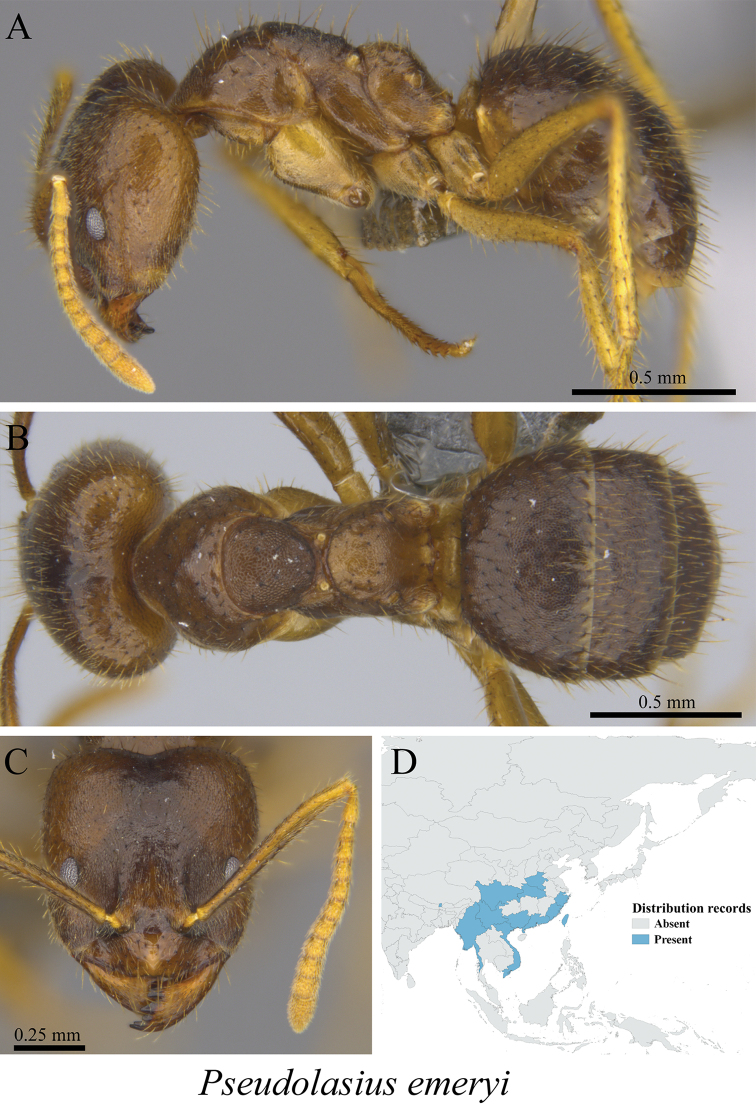
*Pseudolasius emeryi* worker (MCZ-ENT00762951) **A** mesosoma in profile view **B** mesosoma in dorsal view **C** head in front view **D** global distribution map.

**Figure 55.**
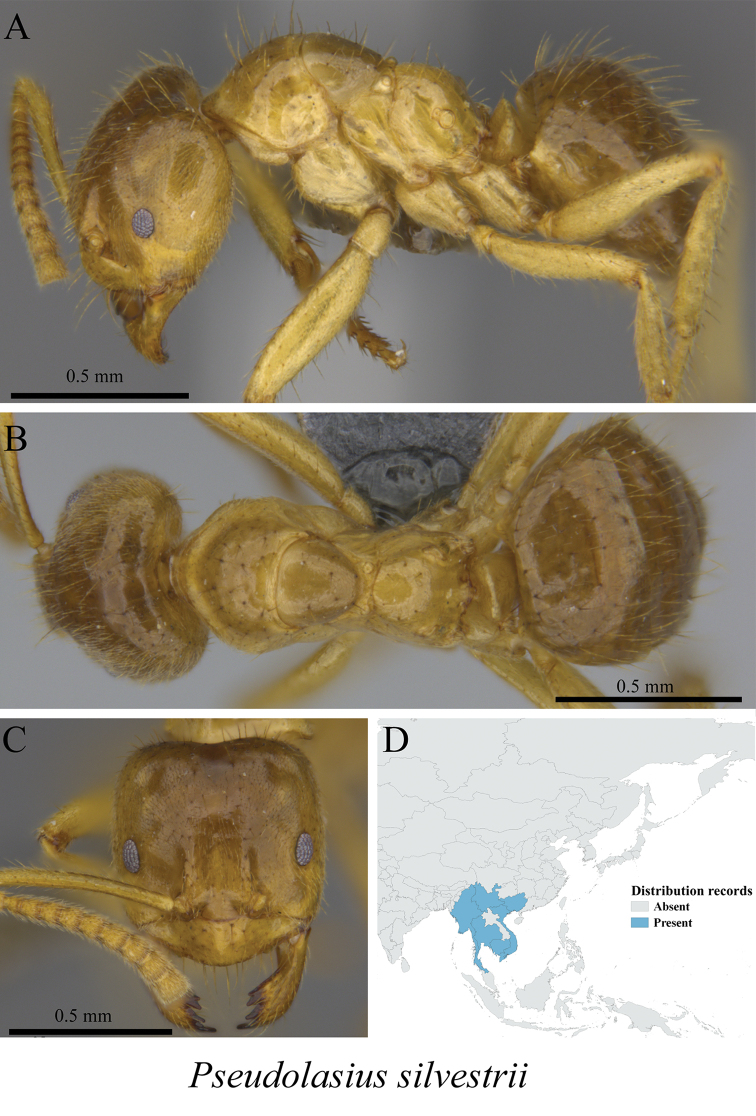
*Pseudolasius silvestrii* worker (MCZ-ENT00762838) **A** mesosoma in profile view **B** mesosoma in dorsal view **C** head in front view **D** global distribution map.

**Figure 56.**
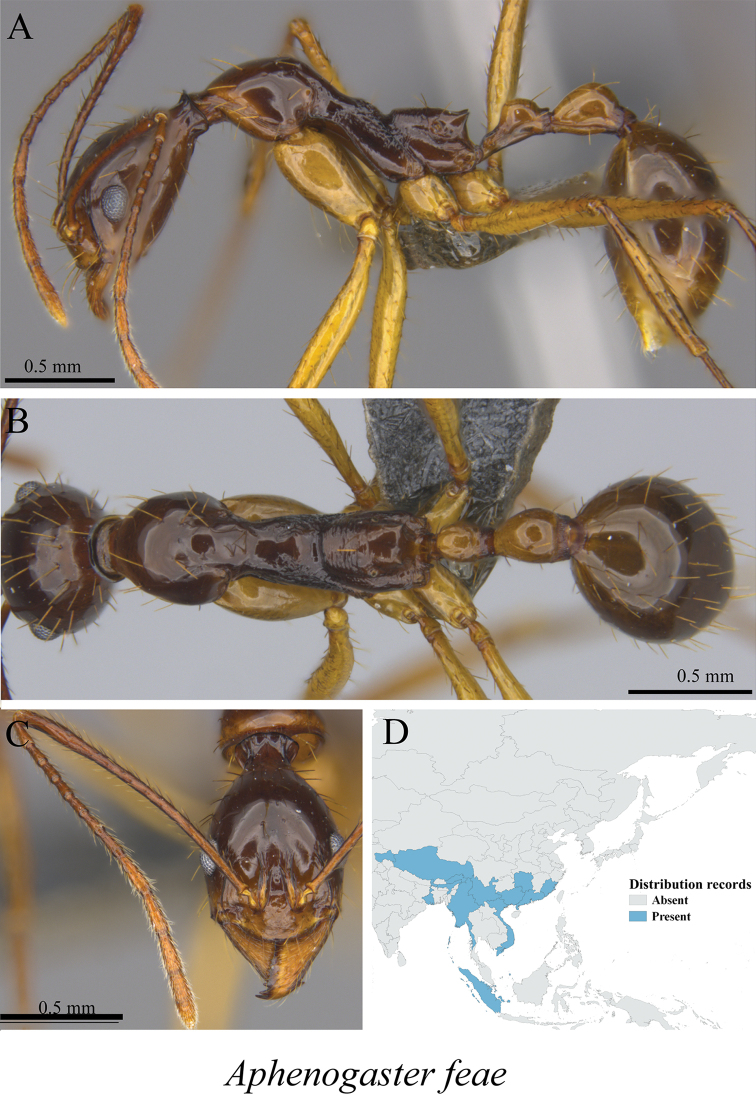
*Aphaenogaster feae* worker (MCZ-ENT00763554) **A** mesosoma in profile view **B** mesosoma in dorsal view **C** head in front view **D** global distribution map..

**Figure 57.**
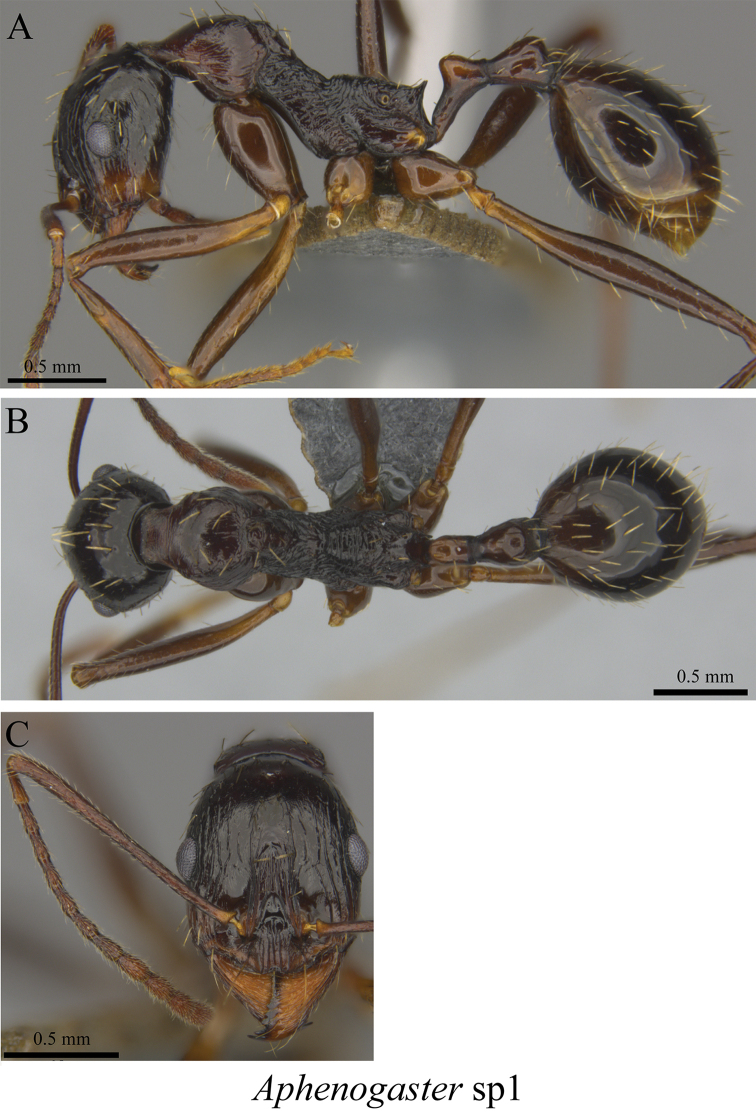
*Aphaenogaster* sp. clm01 worker (MCZ-ENT00762870) **A** mesosoma in profile view **B** mesosoma in dorsal view **C** head in front view.

**Figure 58.**
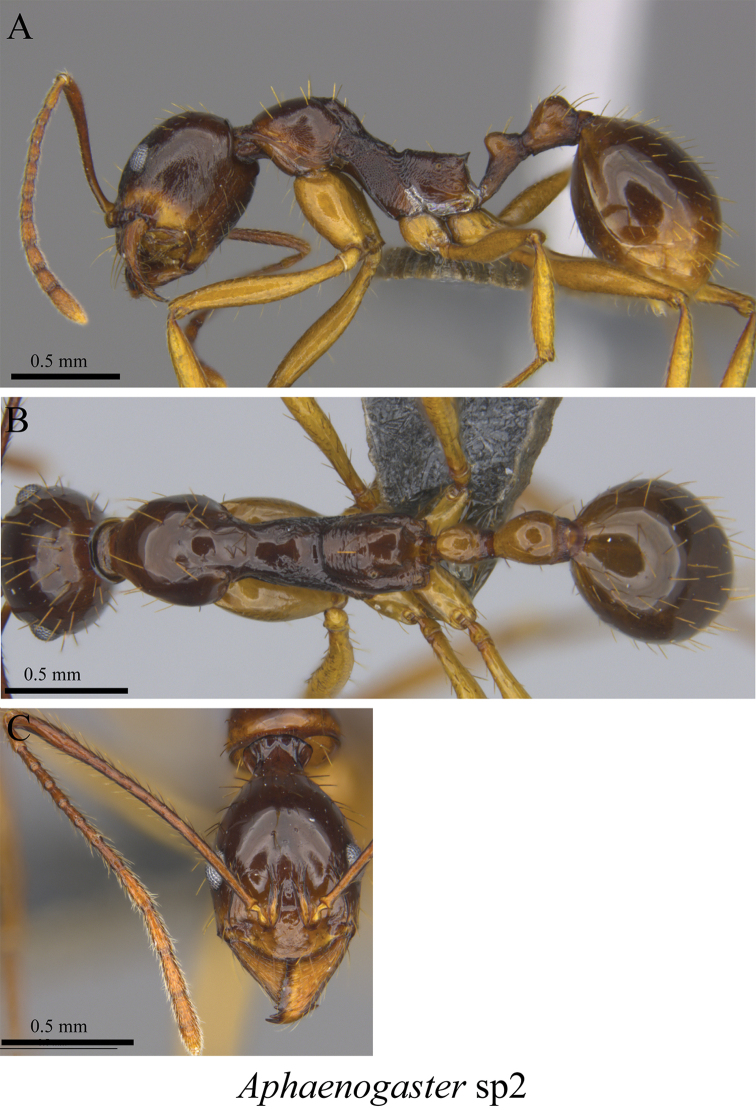
*Aphaenogaster* sp. clm02 worker (MCZ-ENT00763366) **A** mesosoma in profile view **B** mesosoma in dorsal view **C** head in front view.

**Figure 59.**
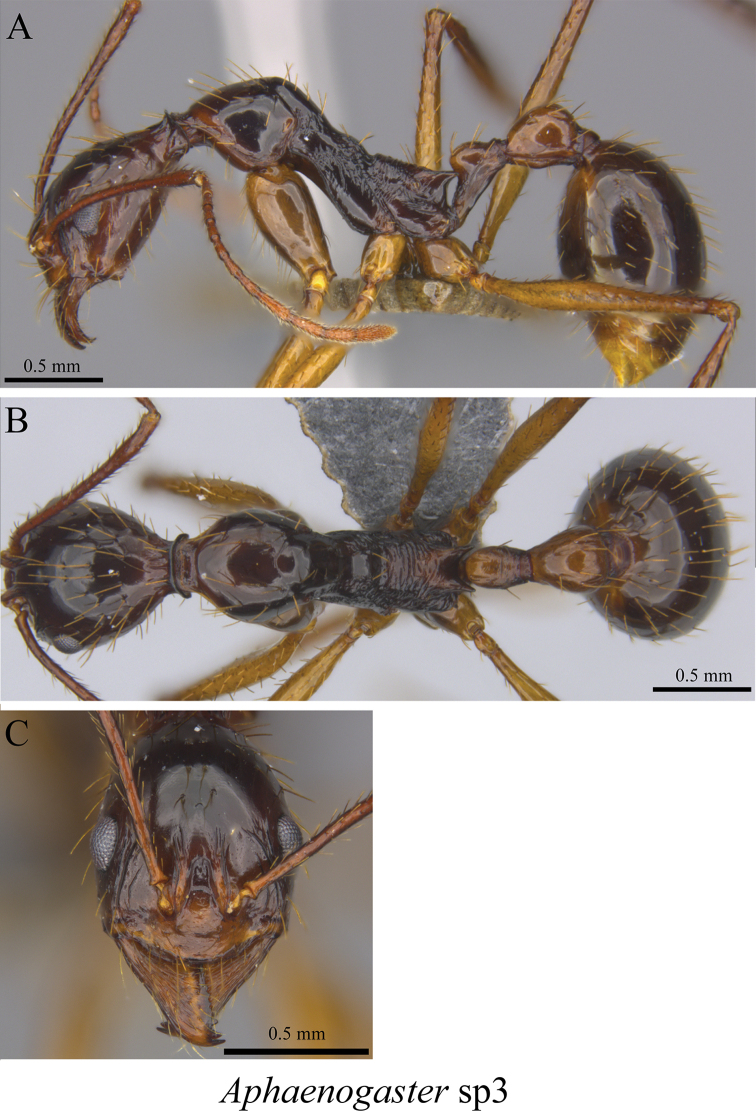
*Aphaenogaster* sp. clm03 worker (MCZ-ENT00763603) **A** mesosoma in profile view **B** mesosoma in dorsal view **C** head in front view.

**Figure 60.**
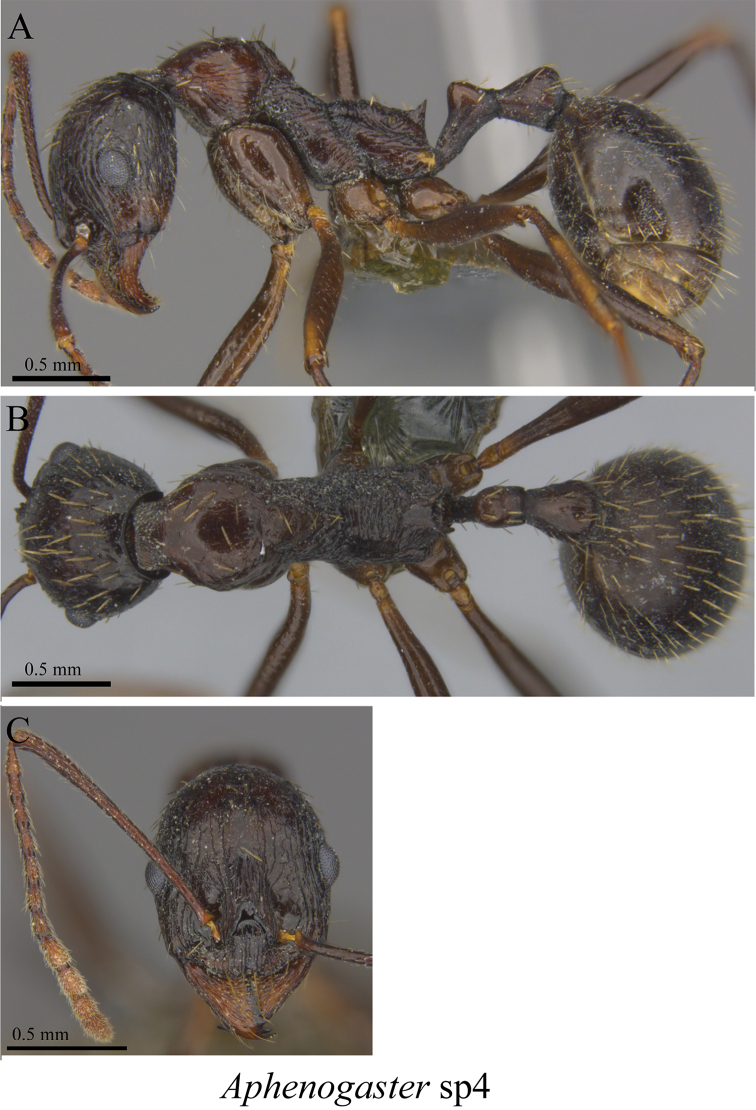
*Aphaenogaster* sp. clm04 worker (MCZ-ENT00764622) **A** mesosoma in profile view **B** mesosoma in dorsal view **C** head in front view.

**Figure 61.**
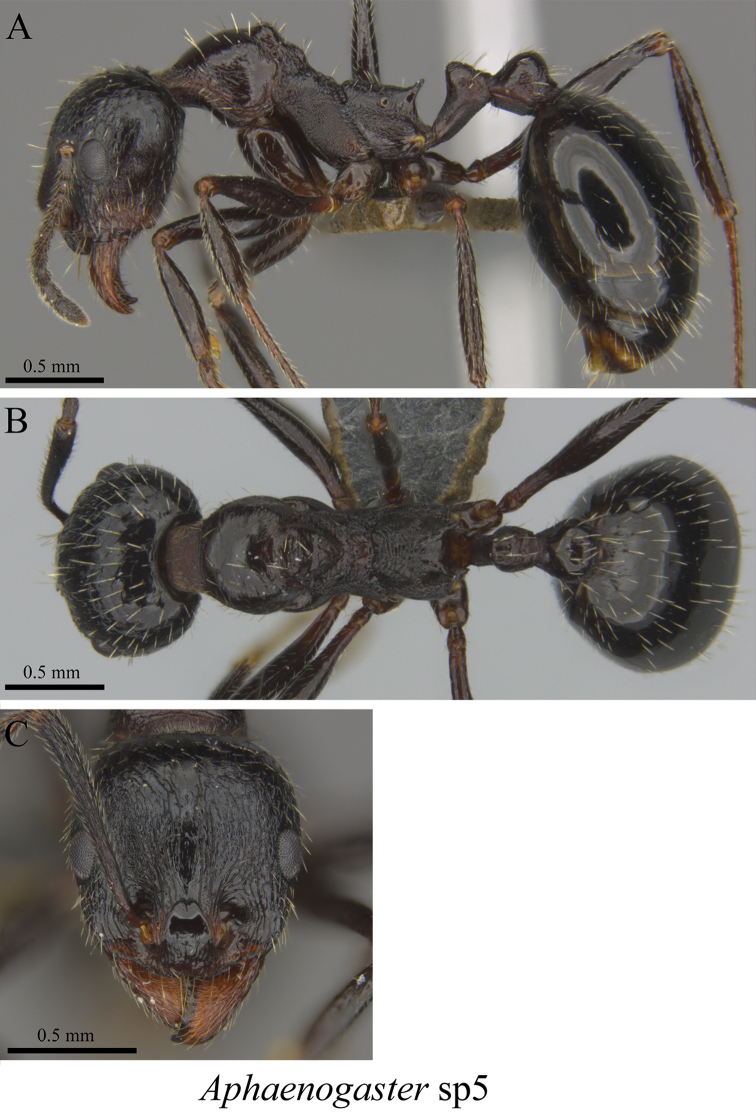
*Aphaenogaster* sp. clm05 worker (MCZ-ENT00762809) **A** mesosoma in profile view **B** mesosoma in dorsal view **C** head in front view.

**Figure 62.**
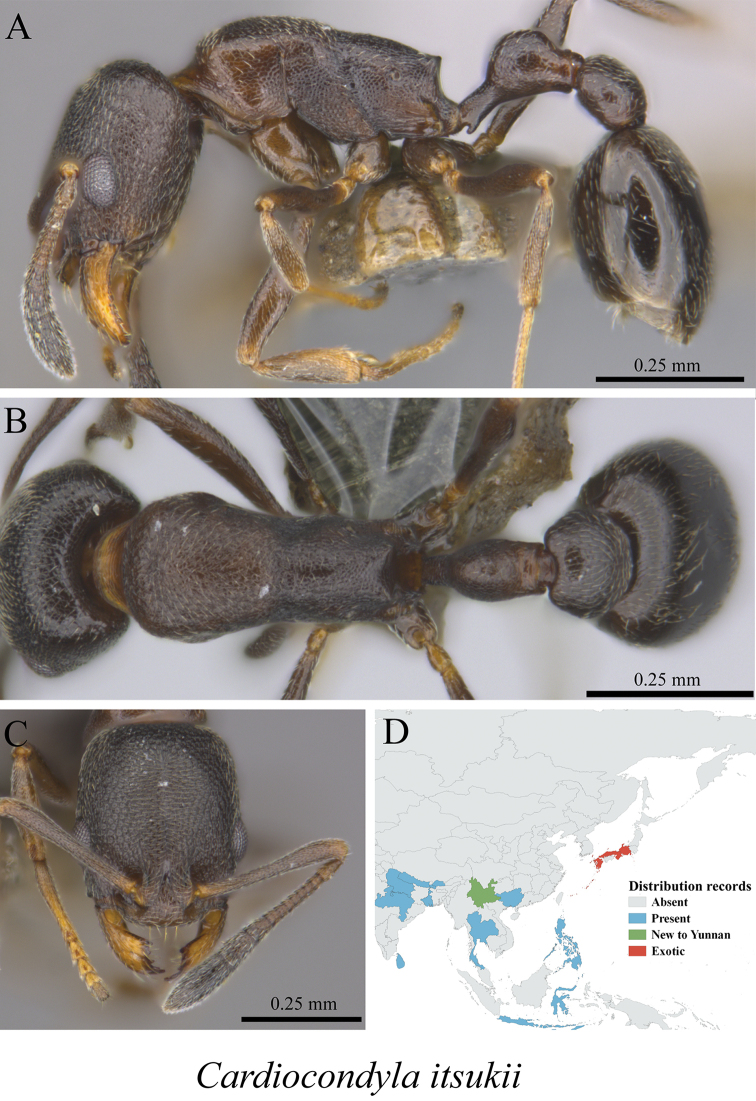
*Cardiocondyla itsukii* worker (MCZ-ENT00762820, new to Yunnan) **A** mesosoma in profile view **B** mesosoma in dorsal view **C** head in front view **D** global distribution map.

**Figure 63.**
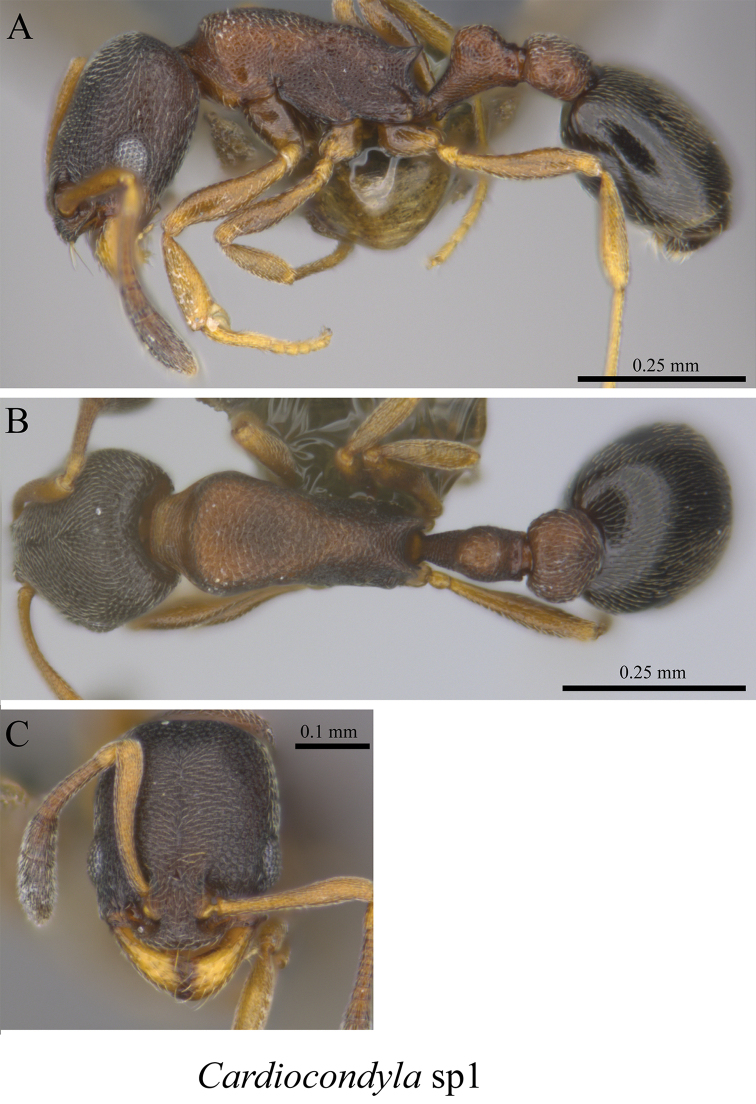
*Cardiocondyla* sp. clm01worker (MCZ-ENT00763607) **A** mesosoma in profile view **B** mesosoma in dorsal view **C** head in front view.

**Figure 64.**
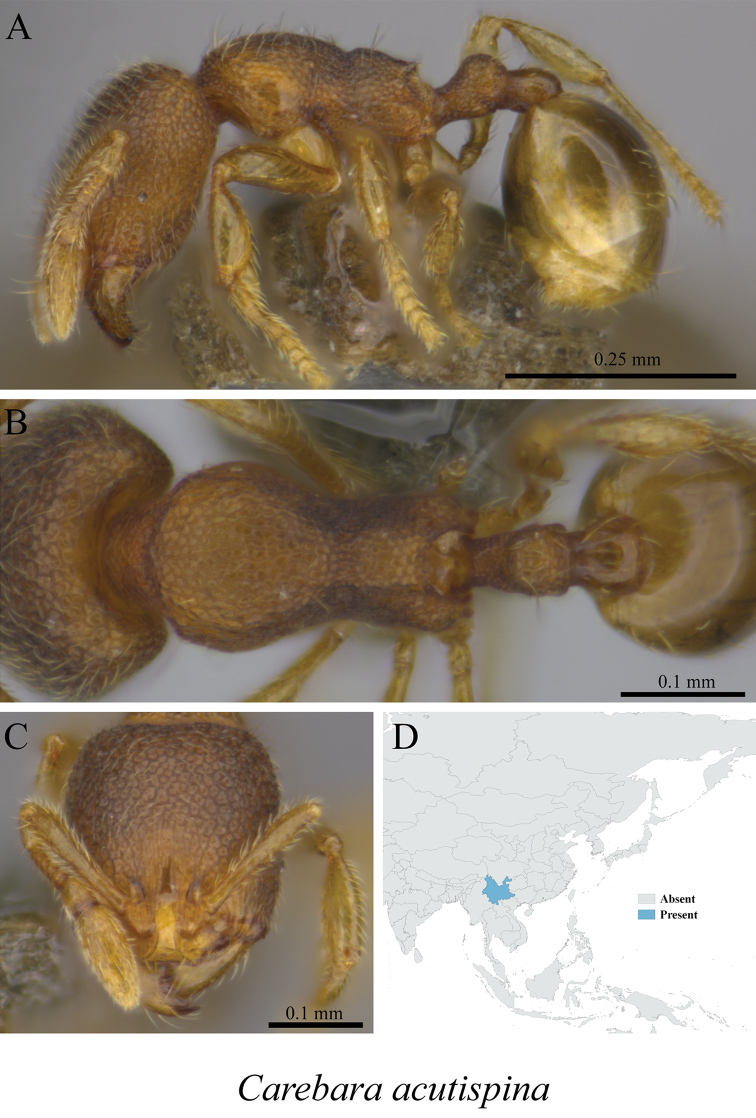
*Carebara affinis* worker (MCZ-ENT00759841) **A** mesosoma in profile view **B** mesosoma in dorsal view **C** head in front view **D** global distribution map.

**Figure 65.**
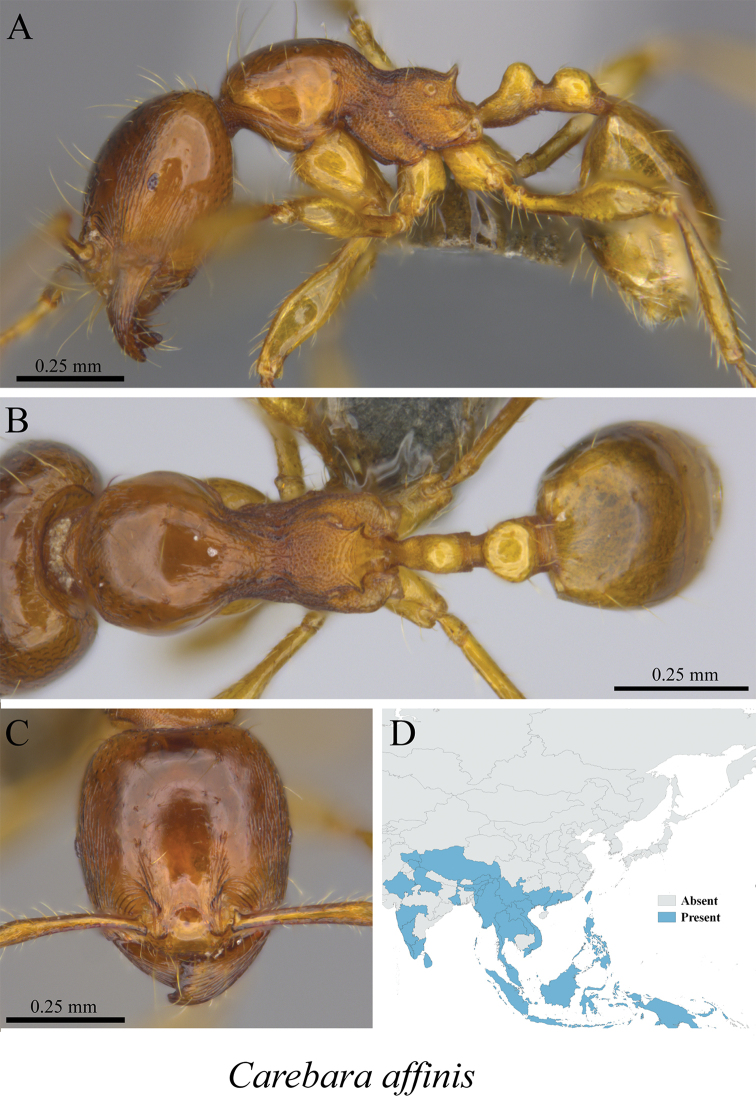
*Carebara acutispina* worker (MCZ-ENT00759773) **A** mesosoma in profile view **B** mesosoma in dorsal view **C** head in front view **D** global distribution map.

**Figure 66.**
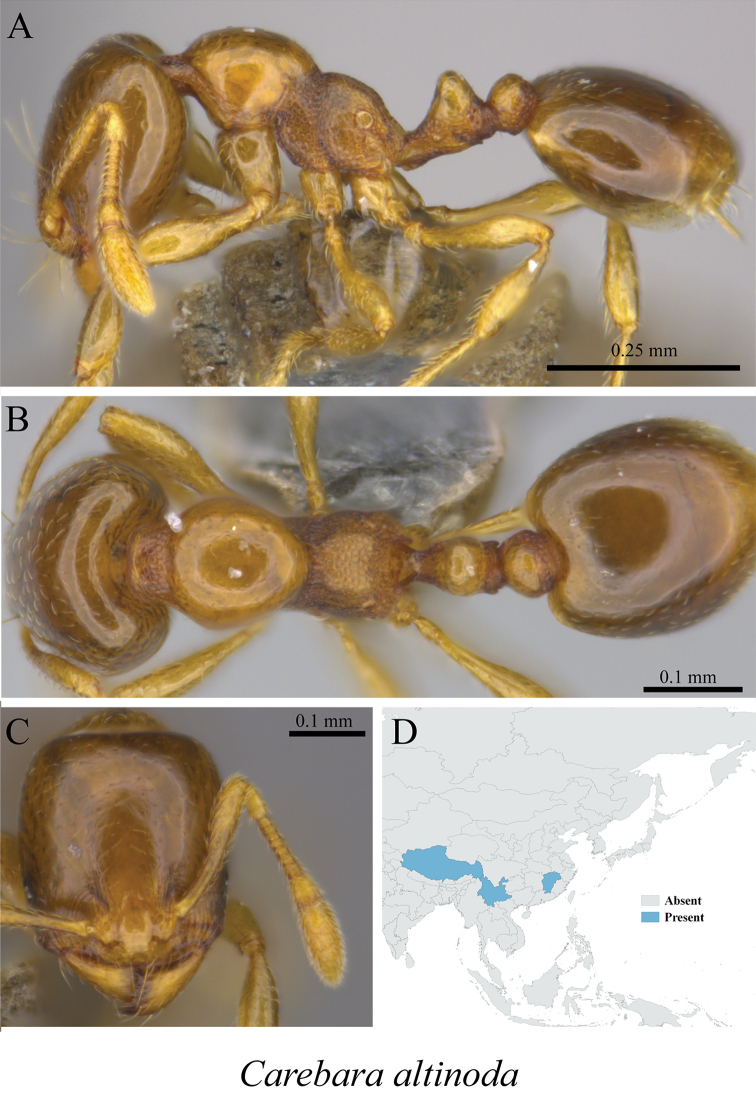
*Carebara altinoda* worker (MCZ-ENT00759928) **A** mesosoma in profile view **B** mesosoma in dorsal view **C** head in front view **D** global distribution map.

**Figure 67.**
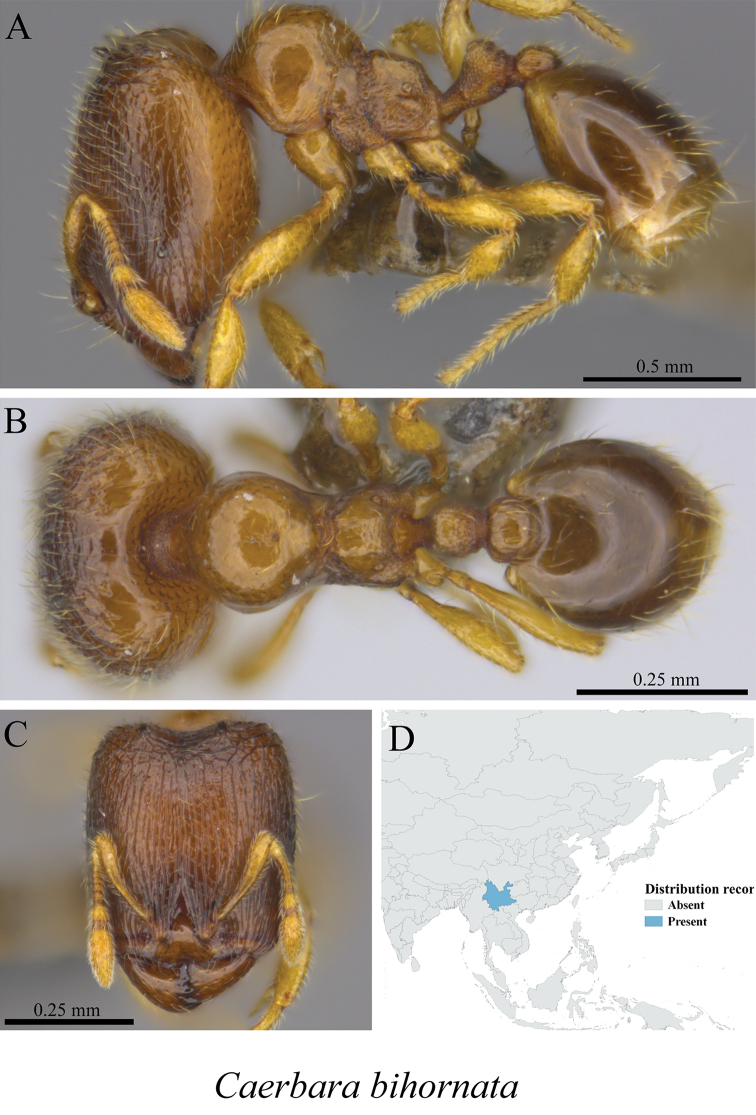
*Carebara bihornata* worker (MCZ-ENT00759796) **A** mesosoma in profile view **B** mesosoma in dorsal view **C** head in front view **D** global distribution map.

**Figure 68.**
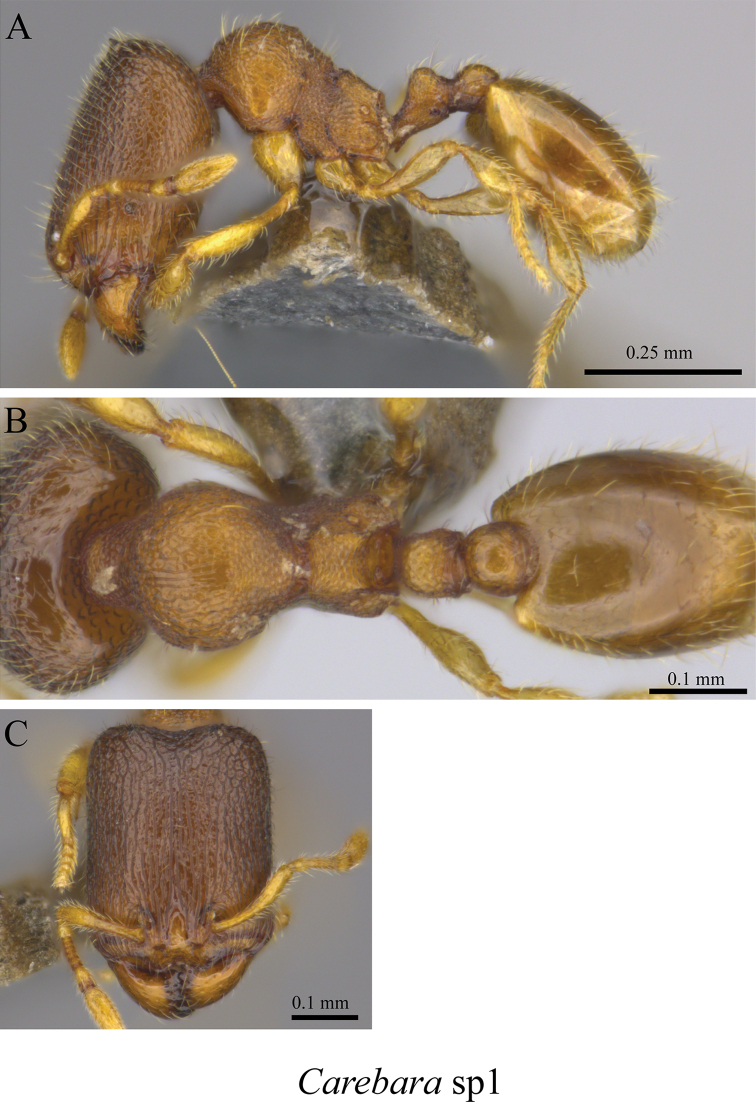
*Carebara* sp. clm01 worker (MCZ-ENT00759855) **A** mesosoma in profile view **B** mesosoma in dorsal view **C** head in front view.

**Figure 69.**
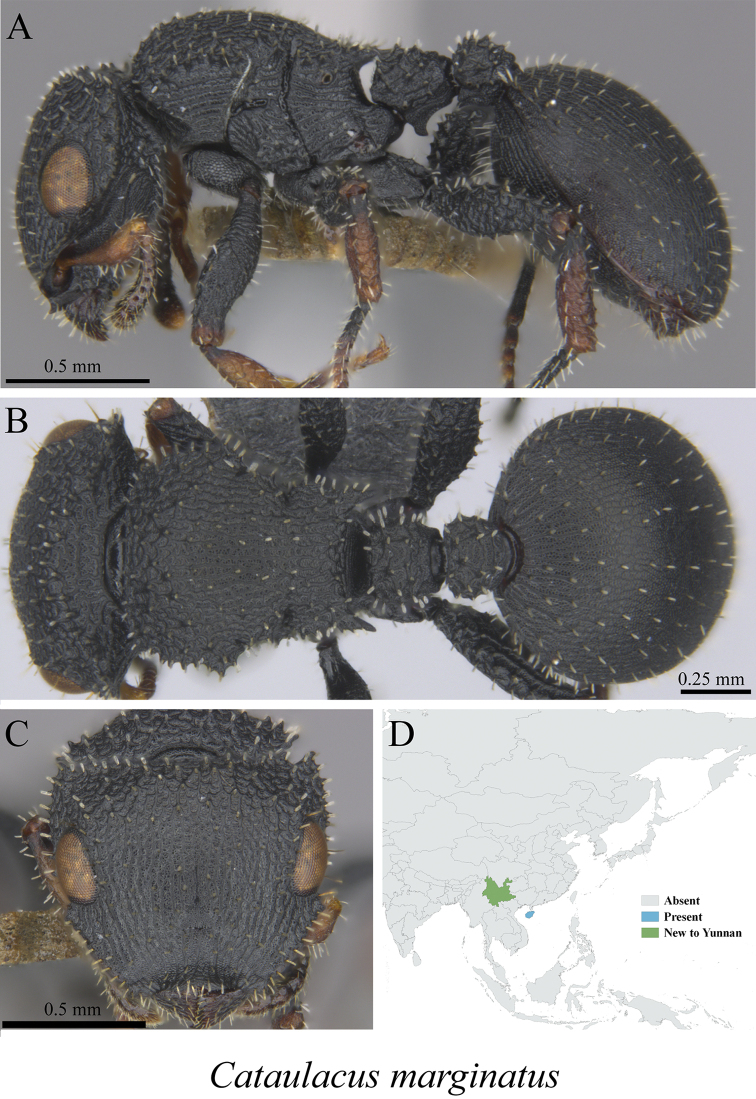
*Cataulacus marginatus* worker (MCZ-ENT00760045, new to Yunnan) **A** mesosoma in profile view **B** mesosoma in dorsal view **C** head in front view **D** global distribution map.

**Figure 70.**
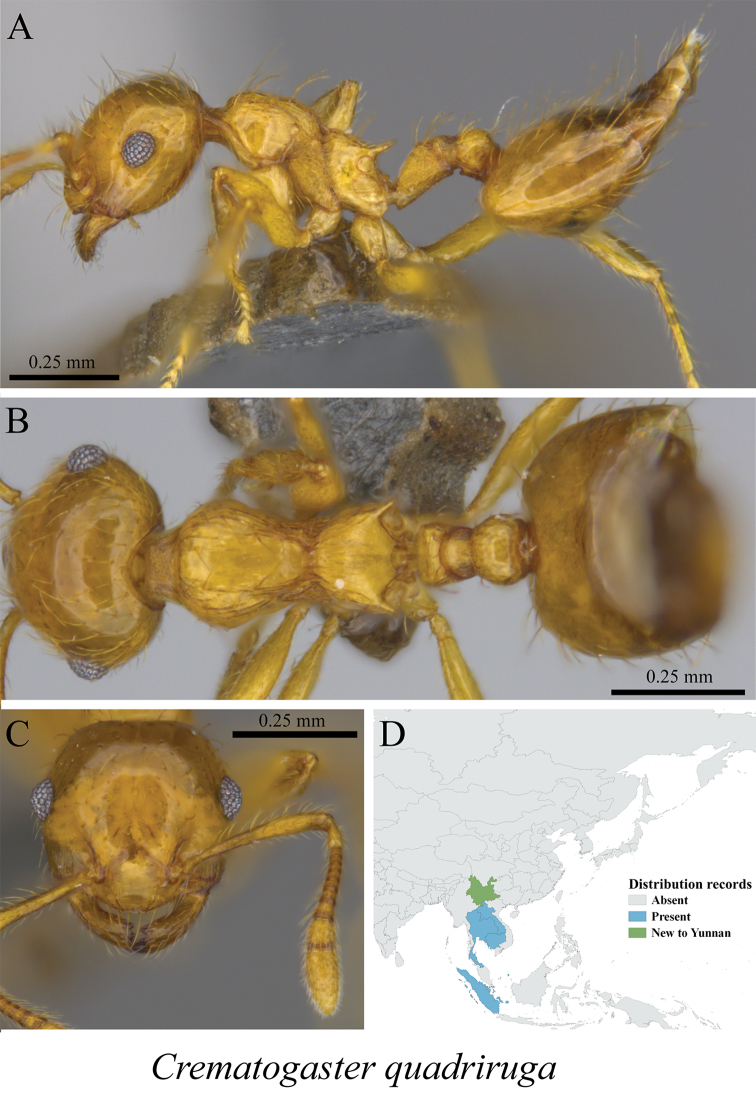
*Crematogaster quadriruga* worker (MCZ-ENT00759778) **A** mesosoma in profile view **B** mesosoma in dorsal view **C** head in front view **D** global distribution map.

**Figure 71.**
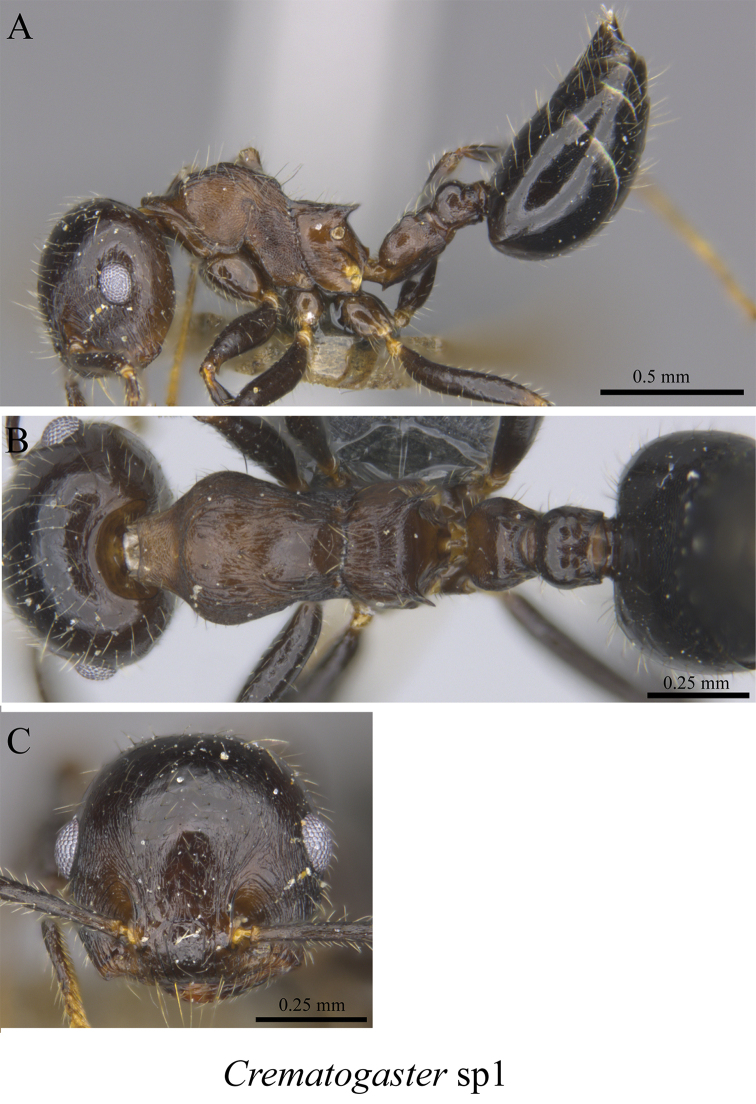
*Crematogaster* sp. clm01 worker (MCZ-ENT00762837) **A** mesosoma in profile view **B** mesosoma in dorsal view **C** head in front view.

**Figure 72.**
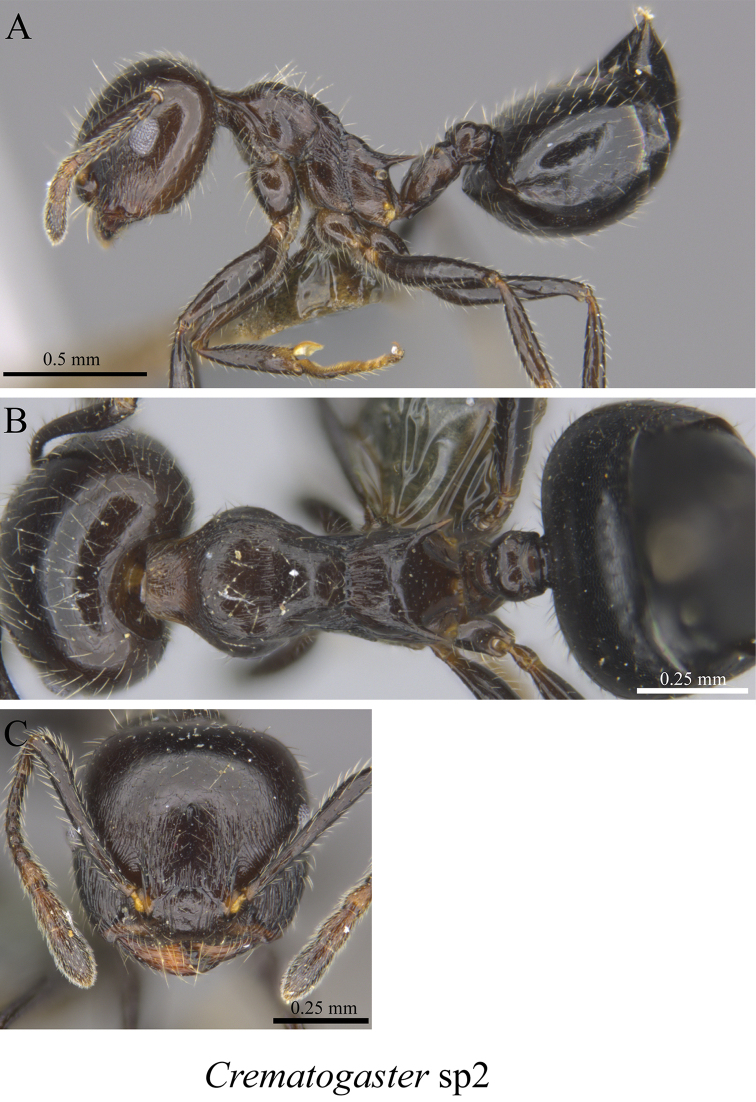
*Crematogaster* sp. clm02 worker (MCZ-ENT00762875) **A** mesosoma in profile view **B** mesosoma in dorsal view **C** head in front view.

**Figure 73.**
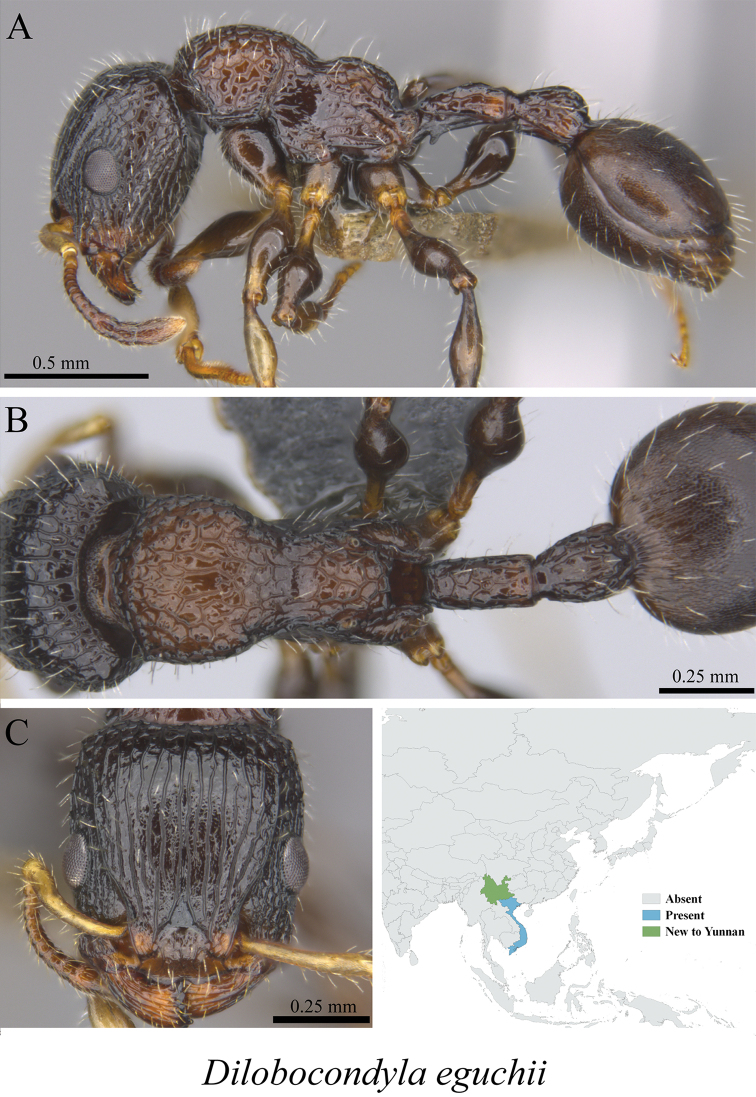
*Dilobocondyla eguchii* worker (MCZ-ENT00763656, new to China) **A** mesosoma in profile view **B** mesosoma in dorsal view **C** head in front view **D** global distribution map.

**Figure 74.**
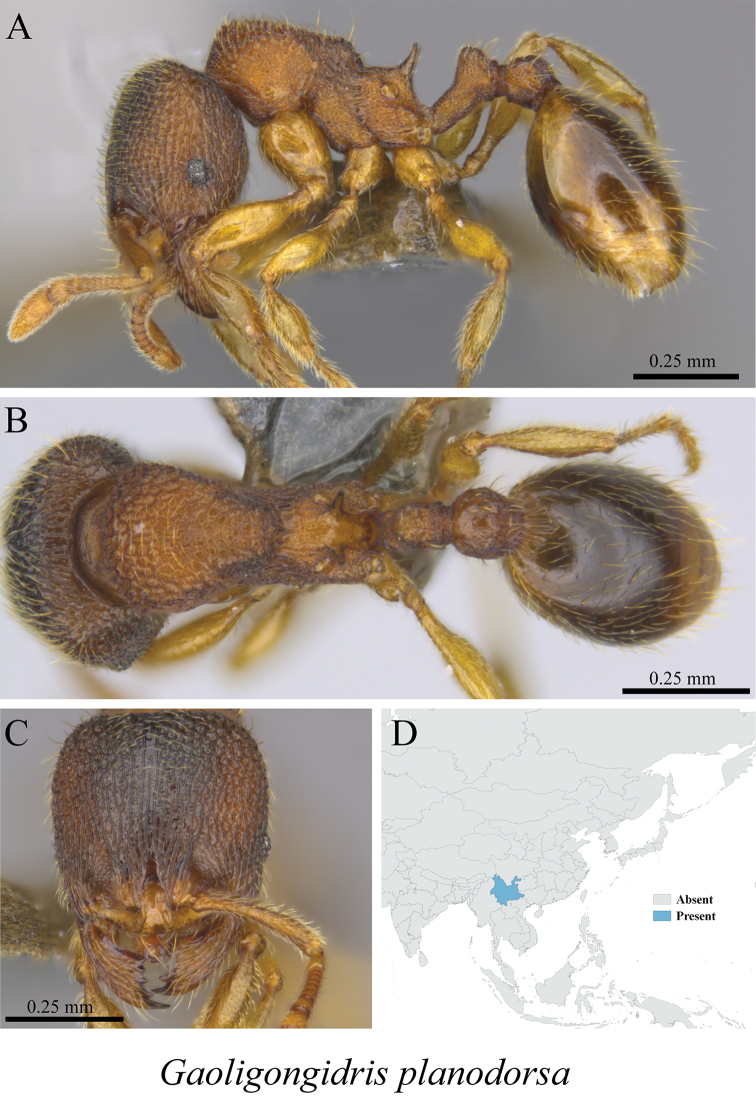
*Gaoligongidris planodorsa* worker (MCZ-ENT00759792) **A** mesosoma in profile view **B** mesosoma in dorsal view **C** head in front view **D** global distribution map.

**Figure 75.**
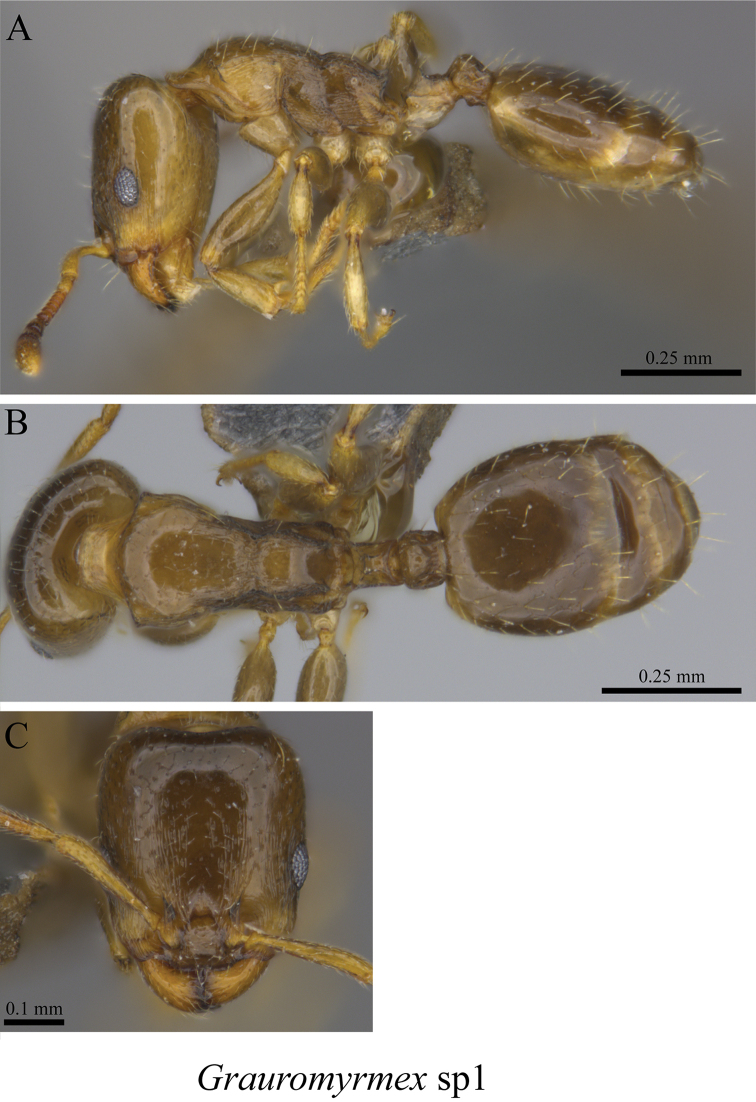
*Gauromyrmex* sp. clm01 worker (MCZ-ENT00764656) **A** mesosoma in profile view **B** mesosoma in dorsal view **C** head in front view **D** global distribution map.

**Figure 76.**
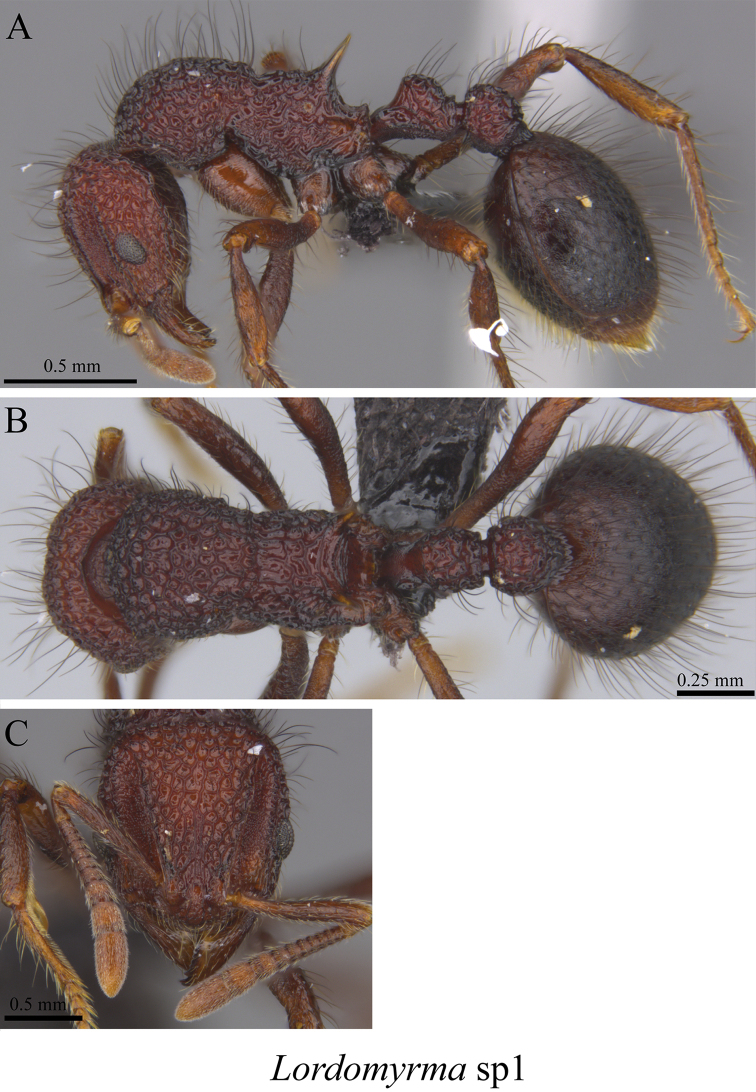
*Lordomyrma* sp. clm01 worker (MCZ-ENT00763514) **A** mesosoma in profile view **B** mesosoma in dorsal view **C** head in front view **D** global distribution map.

**Figure 77.**
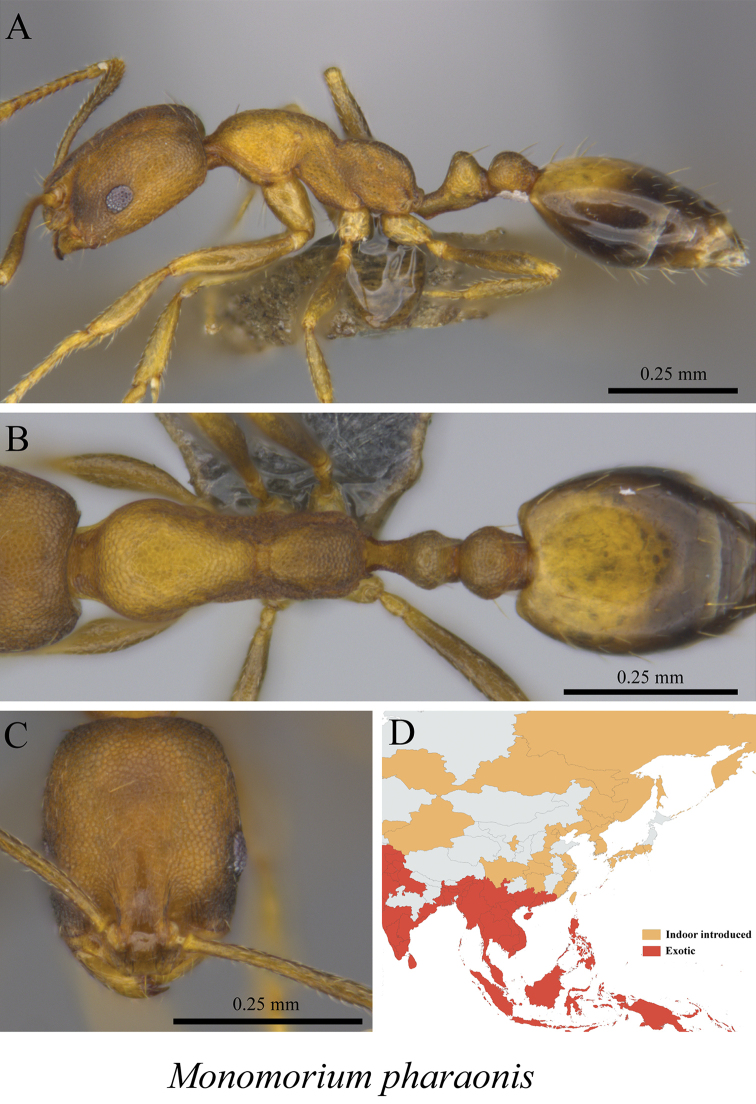
*Monomorium pharaonis* worker (MCZ-ENT00760064) **A** mesosoma in profile view **B** mesosoma in dorsal view **C** head in front view **D** global distribution map.

**Figure 78.**
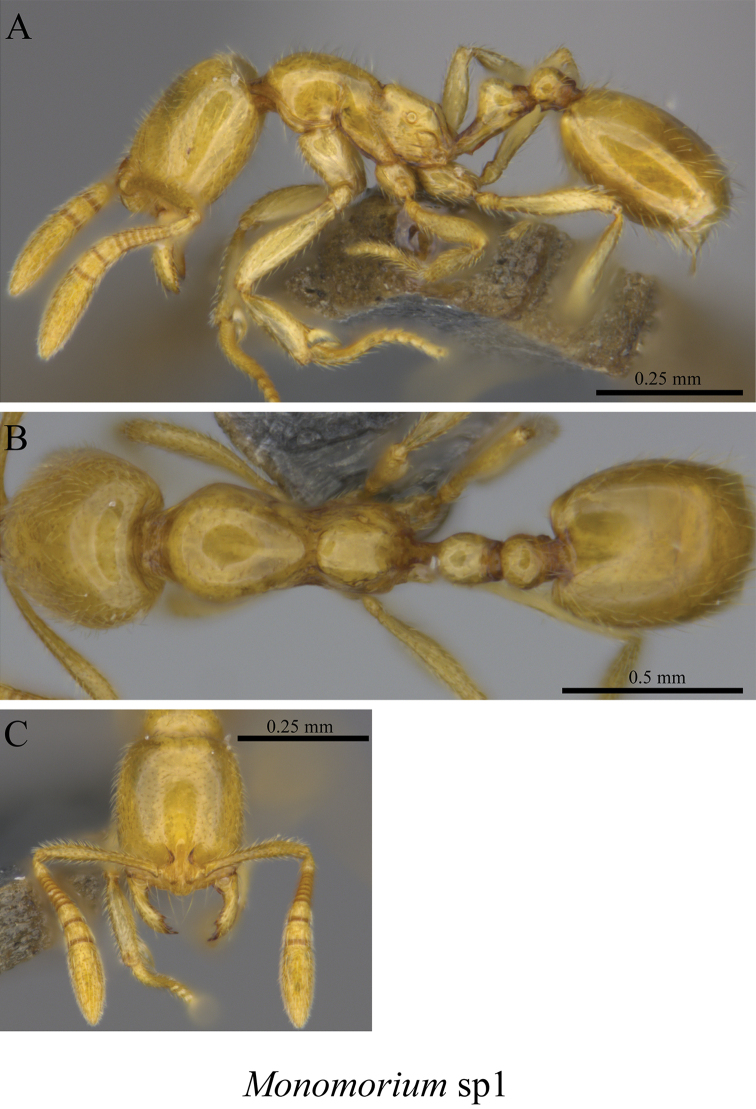
*Monomorium* sp. clm01worker (MCZ-ENT00759771) **A** mesosoma in profile view **B** mesosoma in dorsal view **C** head in front view.

**Figure 79.**
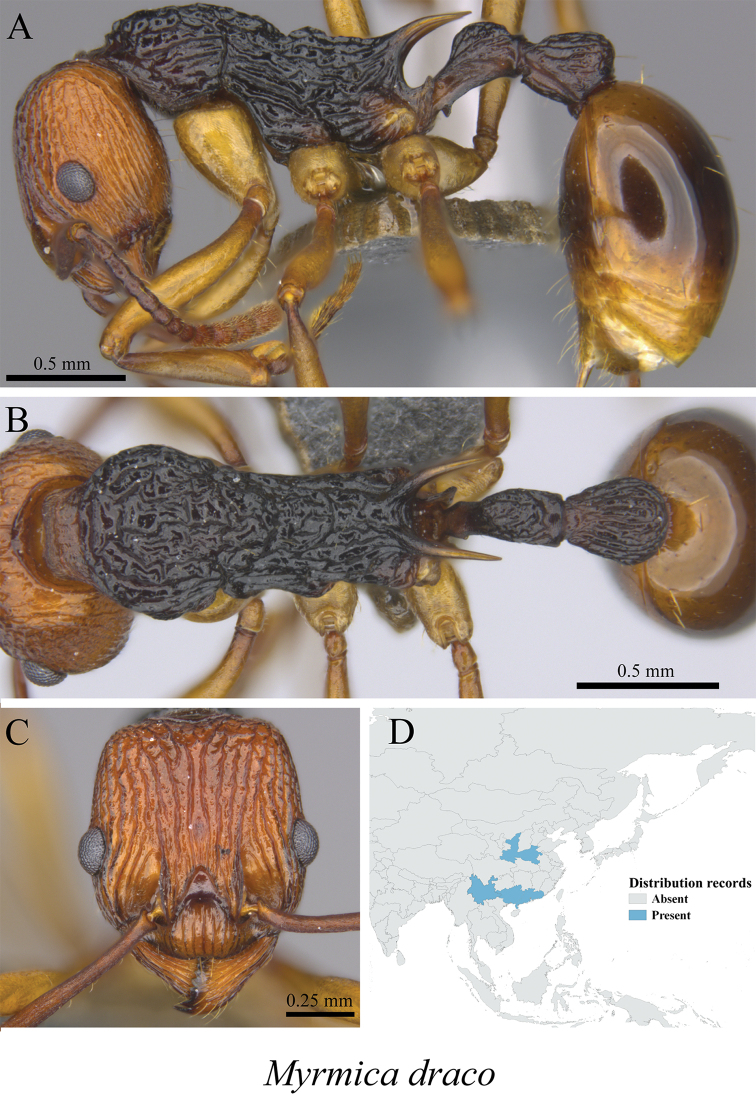
*Myrmica draco* worker (MCZ-ENT00759985) **A** mesosoma in profile view **B** mesosoma in dorsal view **C** head in front view **D** global distribution map.

**Figure 80.**
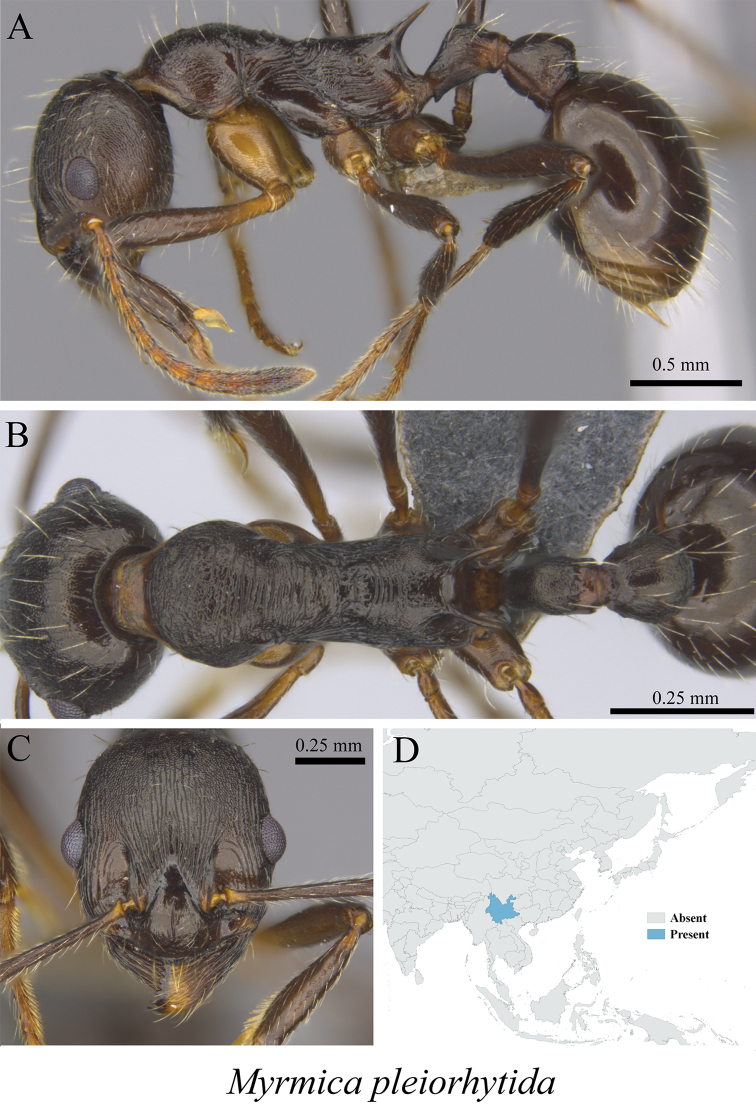
*Myrmica pleiorhytida* worker (MCZ-ENT00759935) **A** mesosoma in profile view **B** mesosoma in dorsal view **C** head in front view **D** global distribution map.

**Figure 81.**
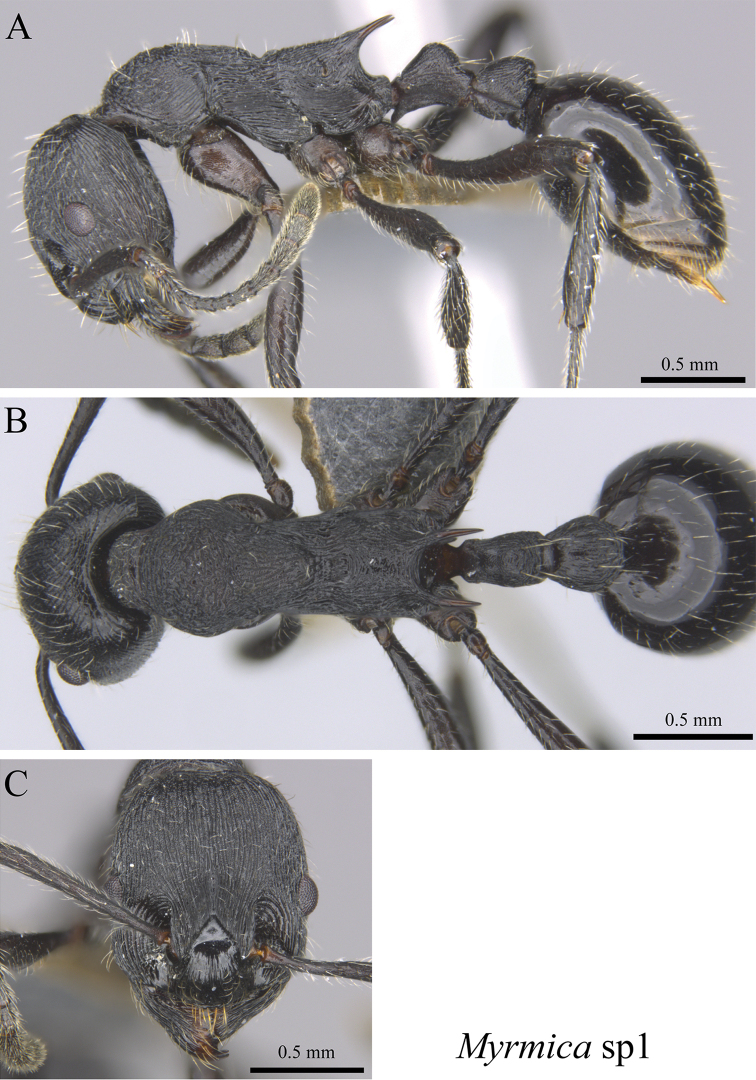
*Myrmica* sp. clm01 worker (MCZ-ENT00763256) **A** mesosoma in profile view **B** mesosoma in dorsal view **C** head in front view.

**Figure 82.**
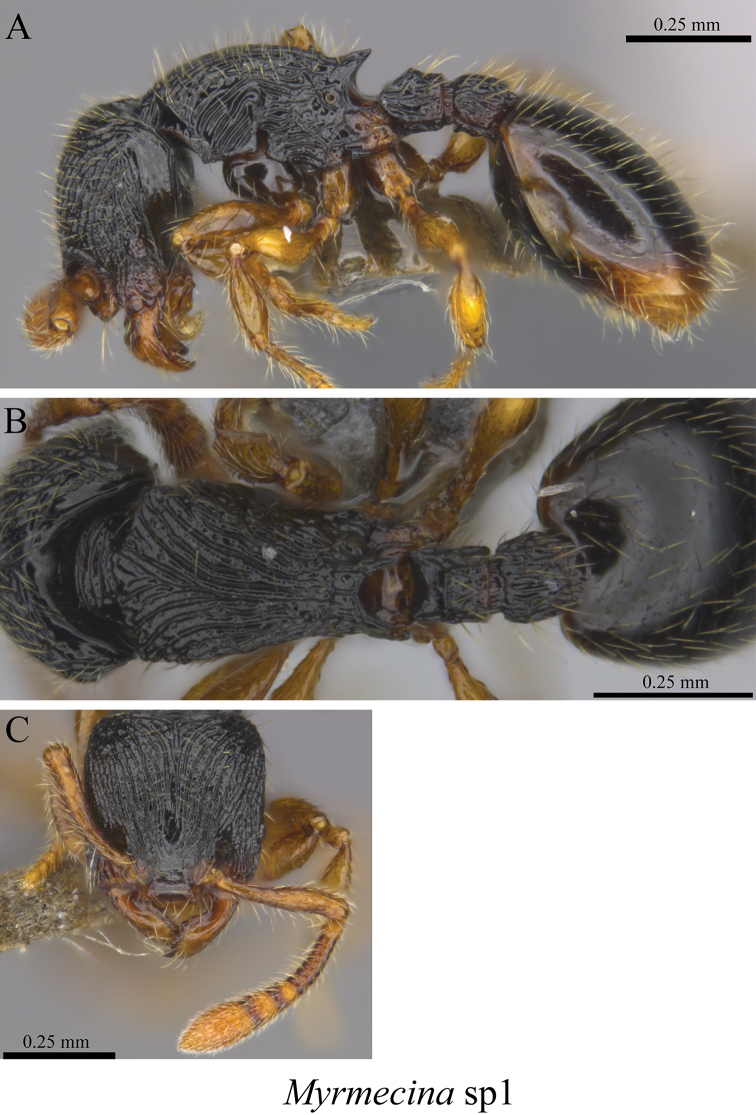
*Myrmecina* sp. clm01 worker (MCZ-ENT00759959) **A** mesosoma in profile view **B** mesosoma in dorsal view **C** head in front view.

**Figure 83.**
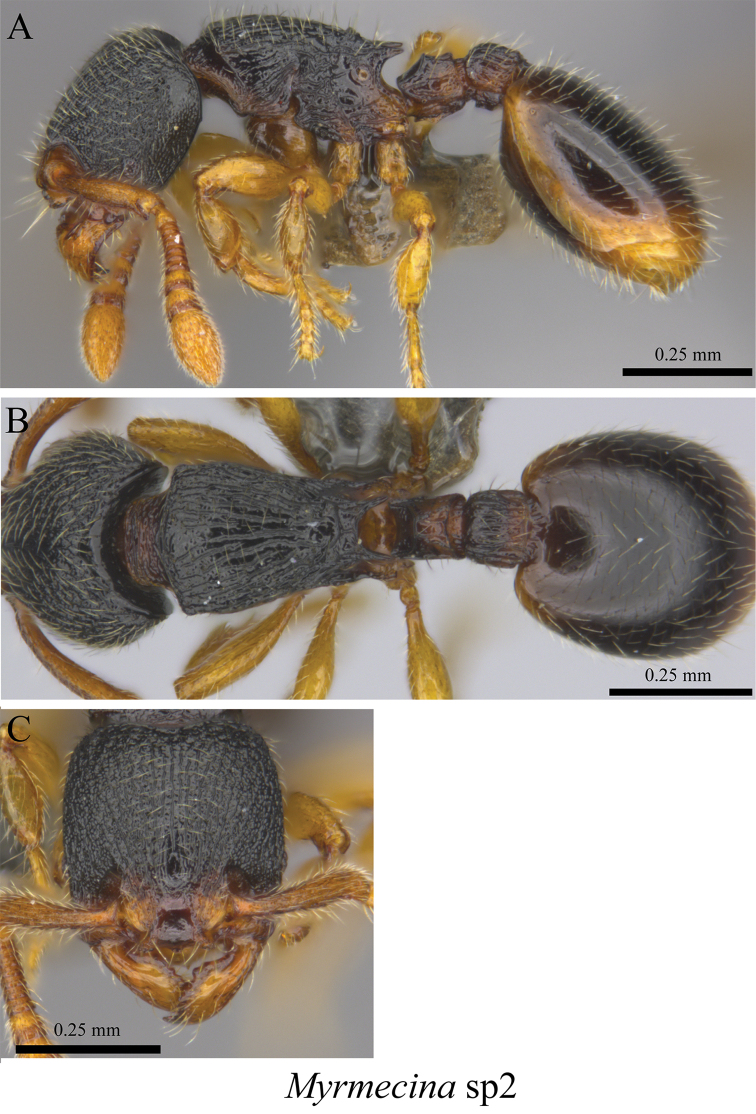
*Myrmecina* sp. clm02 worker (MCZ-ENT00759803). **A** mesosoma in profile view **B** mesosoma in dorsal view **C** head in front view.

**Figure 84.**
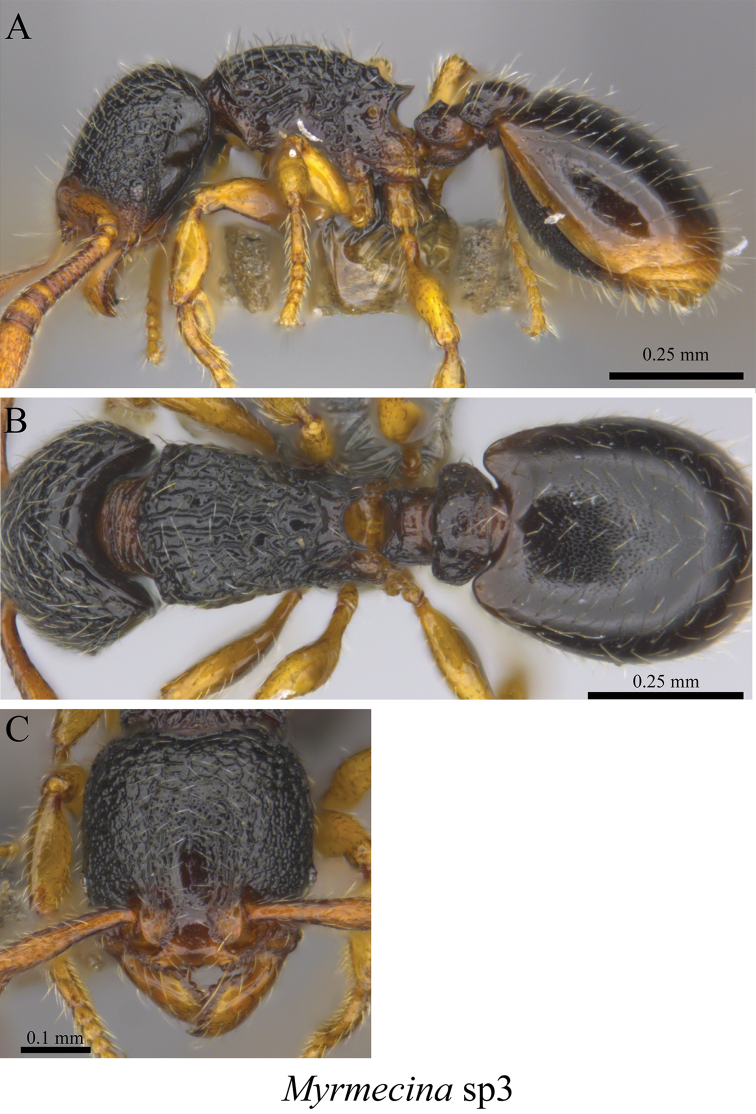
*Myrmecina* sp. clm03 worker (MCZ-ENT00763515). **A** mesosoma in profile view **B** mesosoma in dorsal view **C** head in front view.

**Figure 85.**
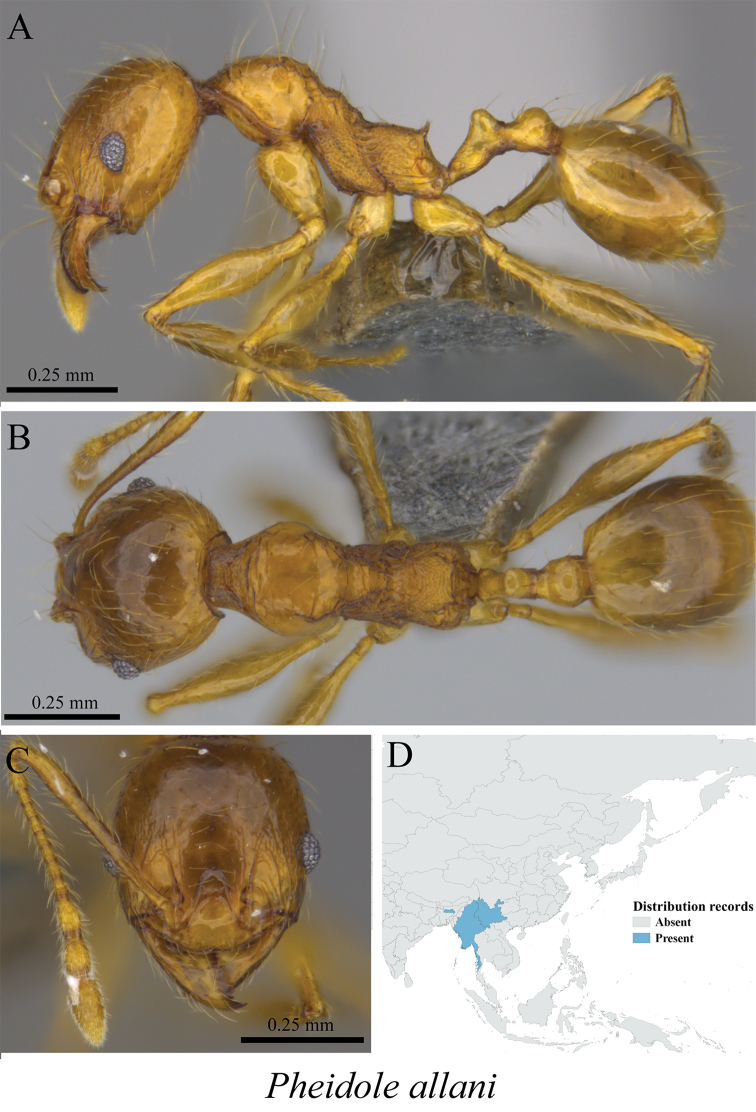
*Pheidole allani* minor worker (MCZ-ENT00759865) **A** mesosoma in profile view **B** mesosoma in dorsal view **C** head in front view **D** global distribution map.

**Figure 86.**
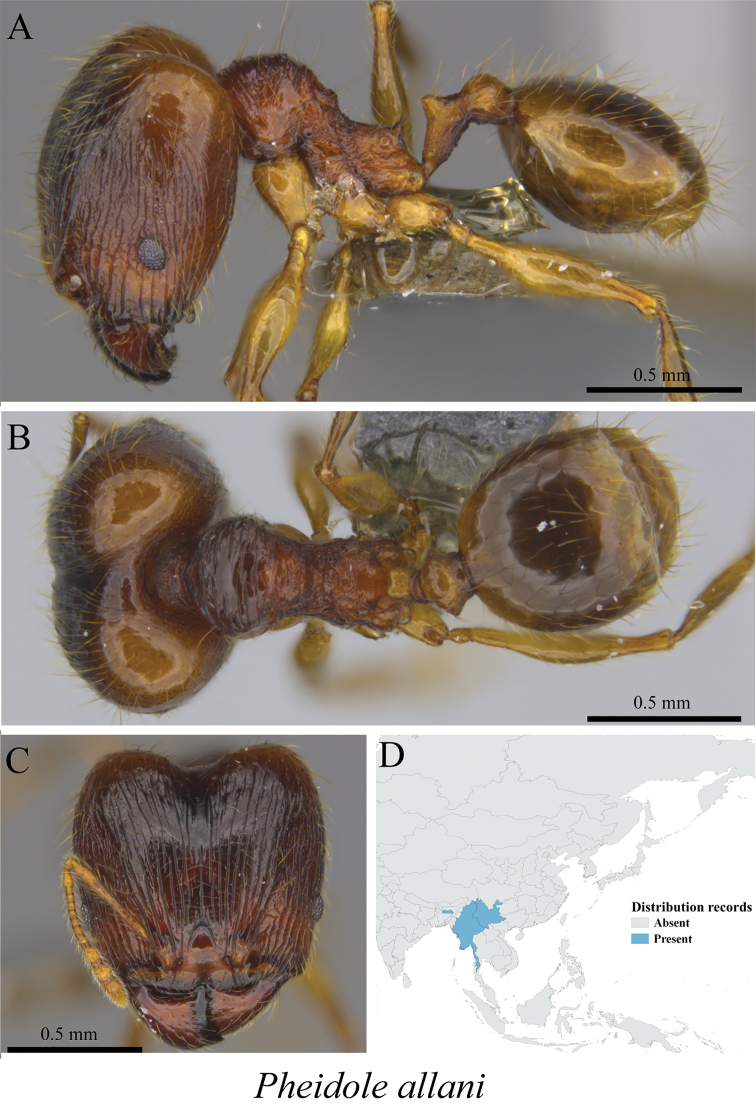
*Pheidole allani* major worker (MCZ-ENT00759866) **A** mesosoma in profile view **B** mesosoma in dorsal view **C** head in front view **D** global distribution map.

**Figure 87.**
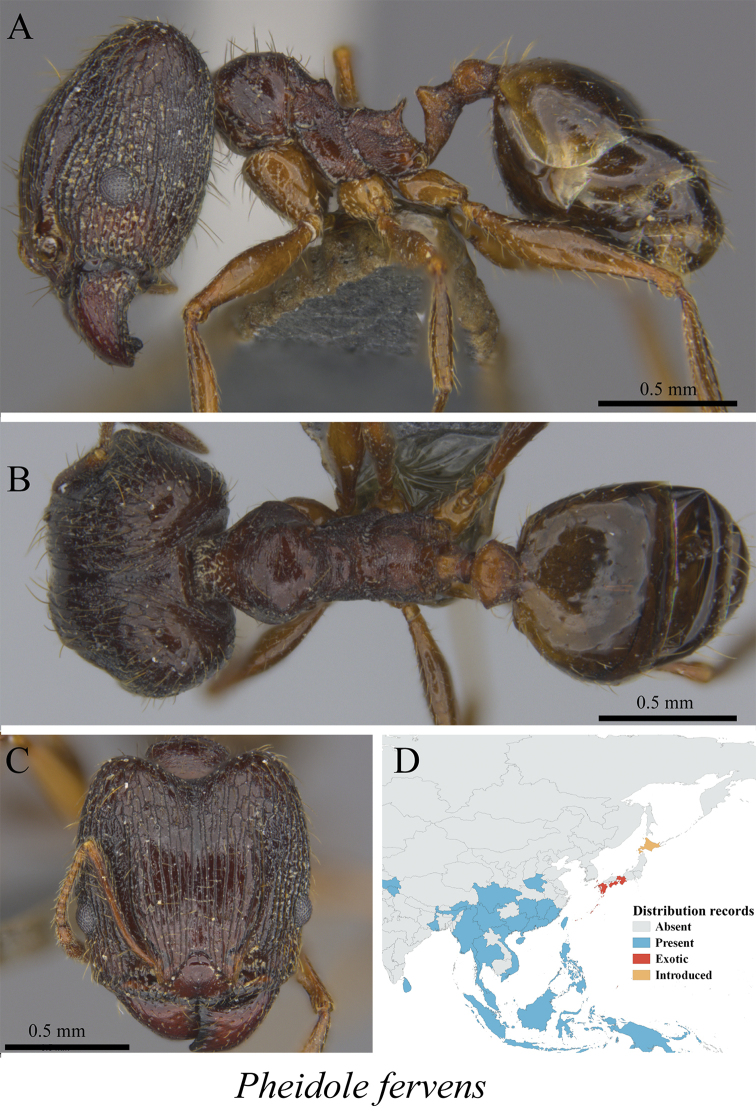
*Pheidole fervens* worker (MCZ-ENT00764619) **A** mesosoma in profile view **B** mesosoma in dorsal view **C** head in front view **D** global distribution map.

**Figure 88.**
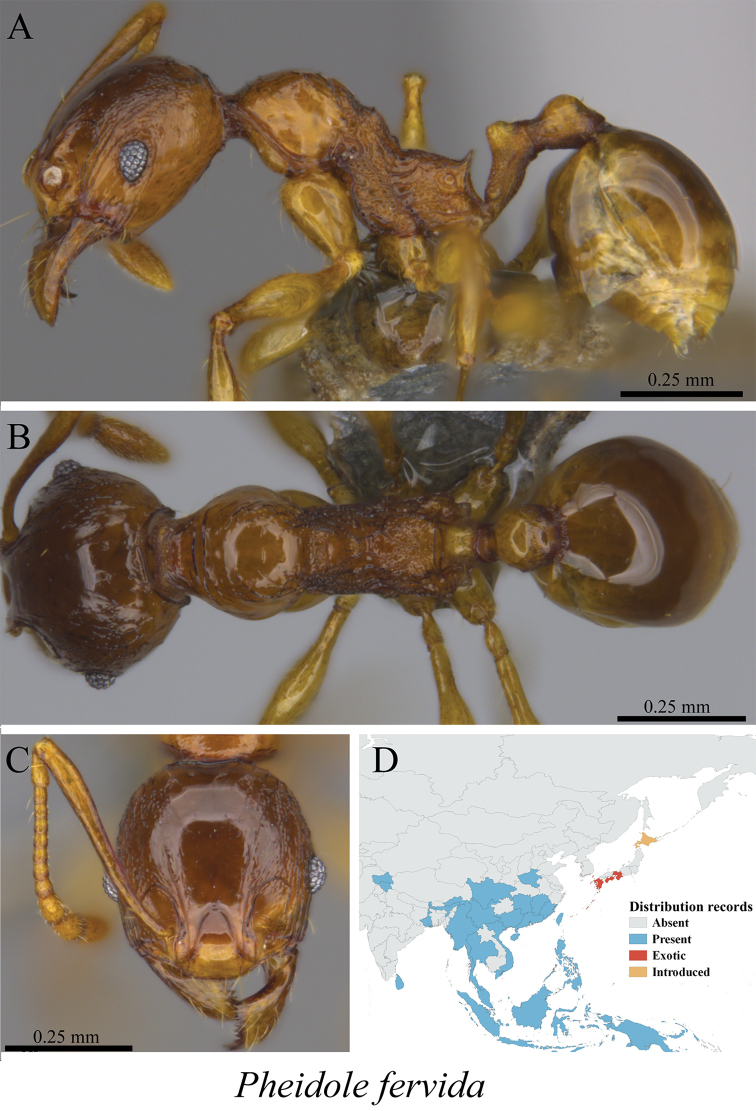
*Pheidole fervida* minor worker (MCZ-ENT00759918) **A** mesosoma in profile view **B** mesosoma in dorsal view **C** head in front view **D** global distribution map.

**Figure 89.**
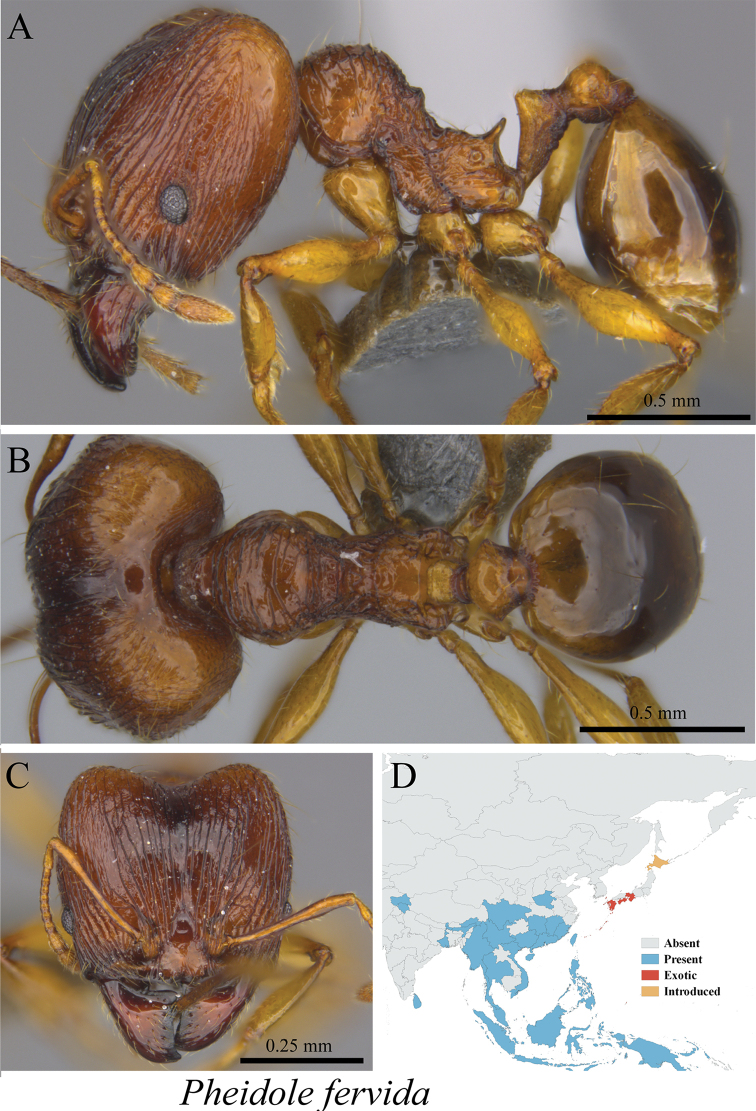
*Pheidole fervida* major worker (MCZ-ENT00760026) **A** mesosoma in profile view **B** mesosoma in dorsal view **C** head in front view **D** global distribution map.

**Figure 90.**
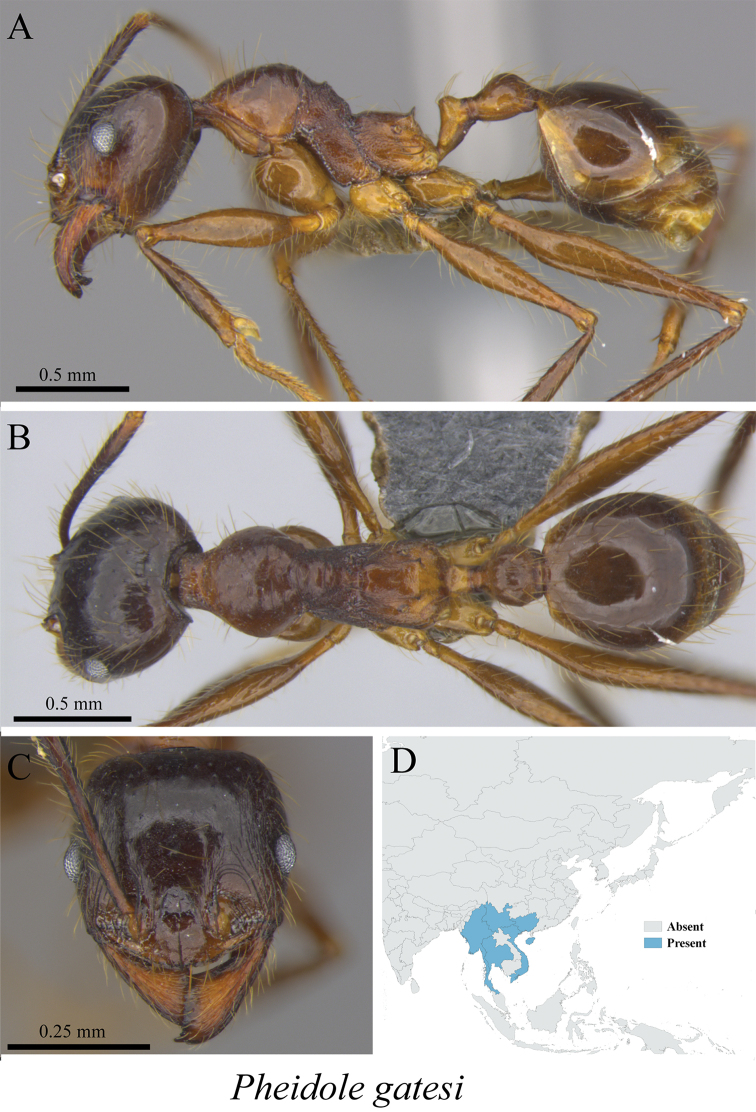
*Pheidole gatesi* worker (MCZ-ENT00763577) **A** mesosoma in profile view **B** mesosoma in dorsal view **C** head in front view **D** global distribution map.

**Figure 91.**
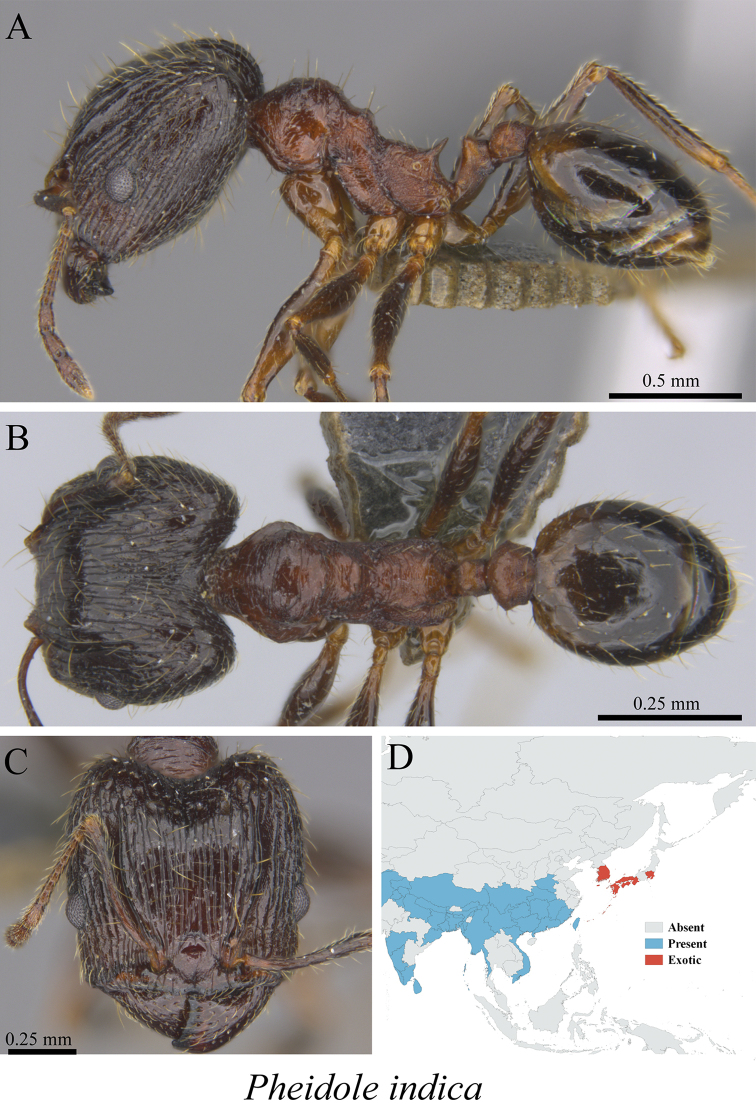
*Pheidole indica* worker (MCZ-ENT00762822) **A** mesosoma in profile view **B** mesosoma in dorsal view **C** head in front view **D** global distribution map.

**Figure 92.**
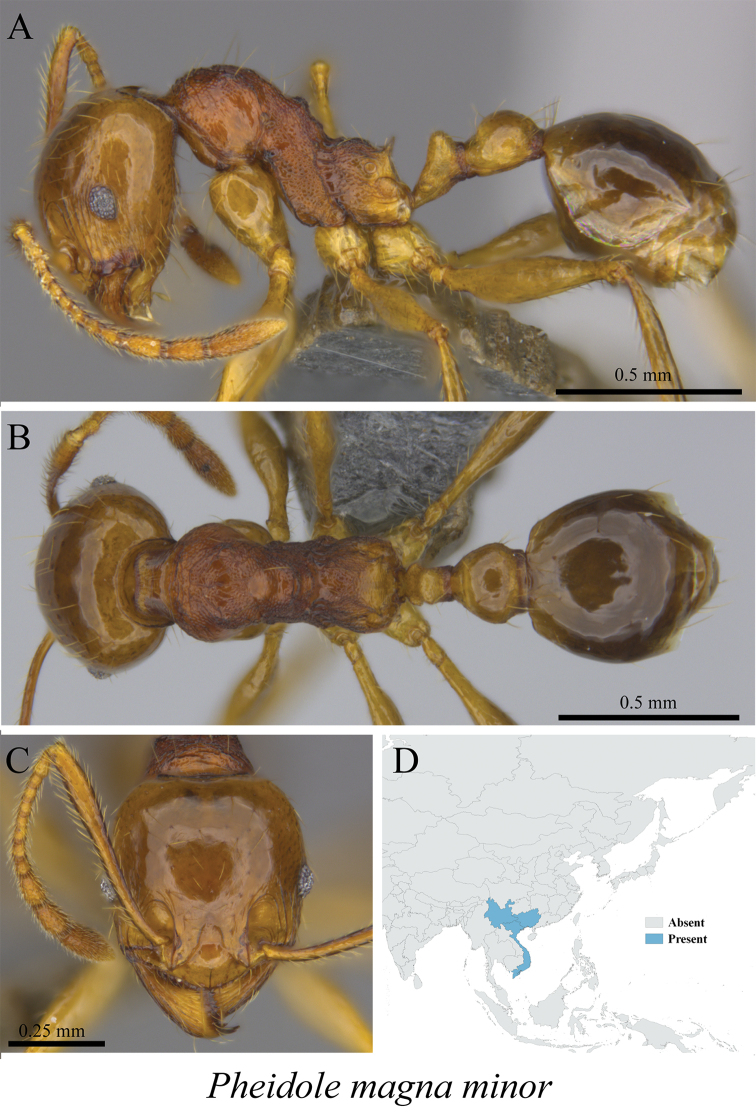
*Pheidole magna* minor worker (MCZ-ENT00759762) **A** mesosoma in profile view **B** mesosoma in dorsal view **C** head in front view **D** global distribution map.

**Figure 93.**
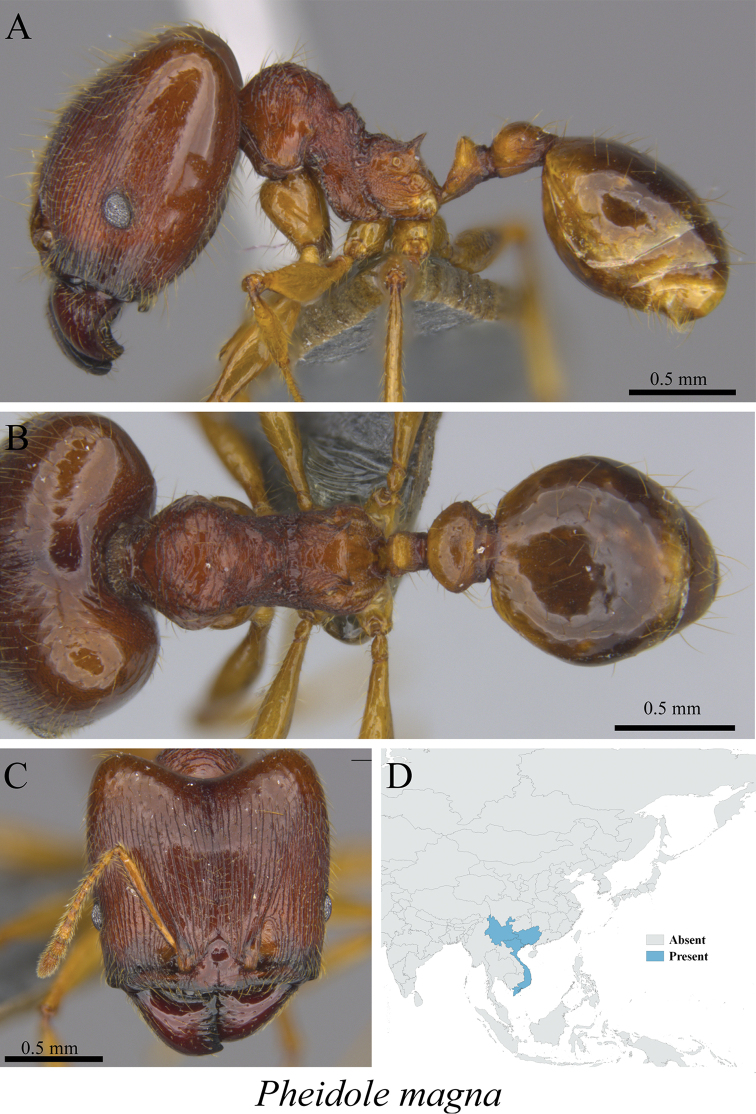
*Pheidole magna* major worker (MCZ-ENT00759980) **A** mesosoma in profile view **B** mesosoma in dorsal view **C** head in front view **D** global distribution map.

**Figure 94.**
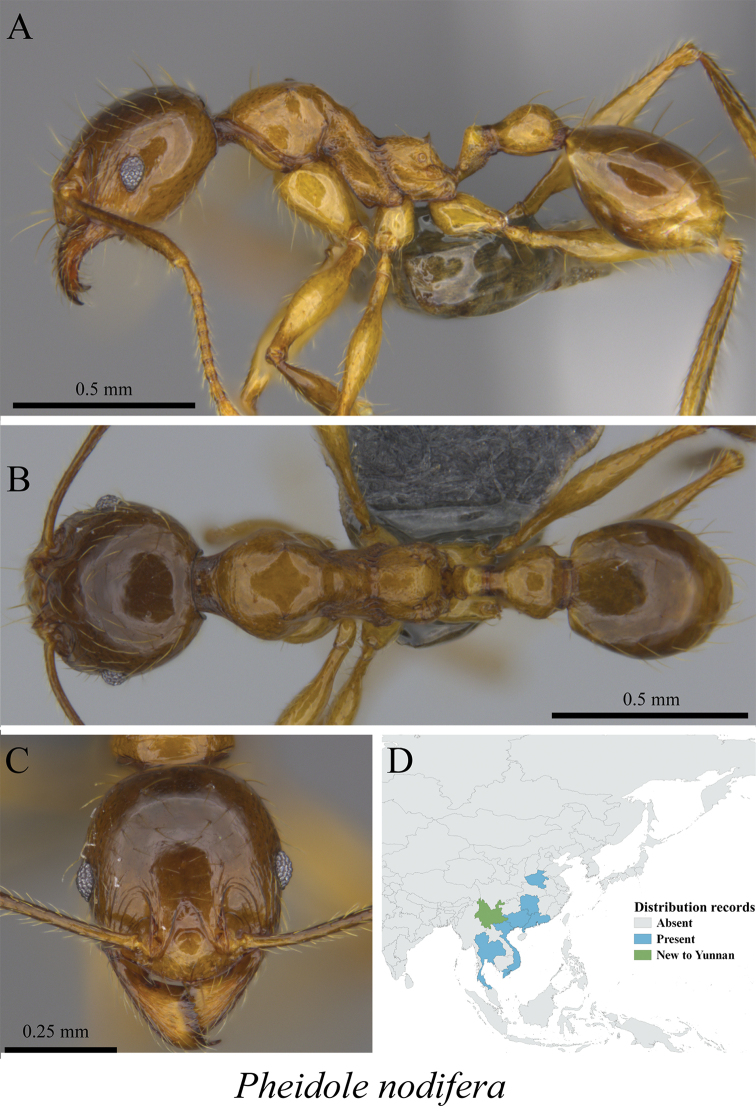
*Pheidole nodifera* worker (MCZ-ENT00759837, new to Yunnan) **A** mesosoma in profile view **B** mesosoma in dorsal view **C** head in front view **D** global distribution map.

**Figure 95.**
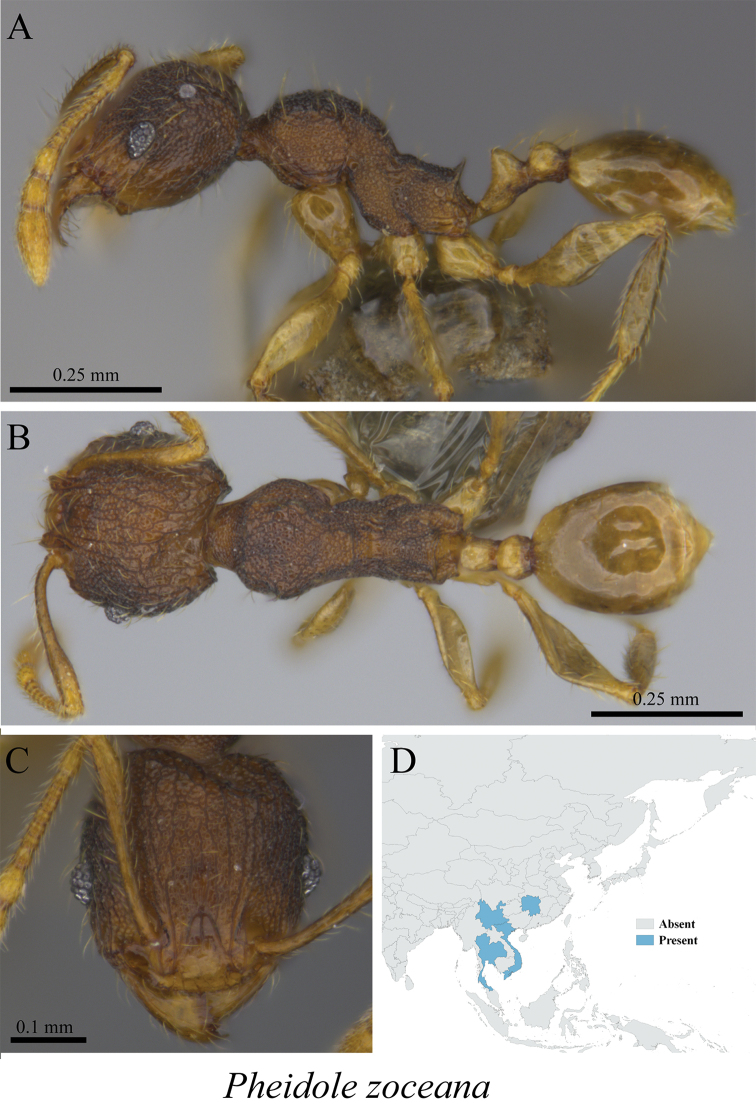
*Pheidole zoceana* minor worker (MCZ-ENT00760015) **A** mesosoma in profile view **B** mesosoma in dorsal view **C** head in front view **D** global distribution map.

**Figure 96.**
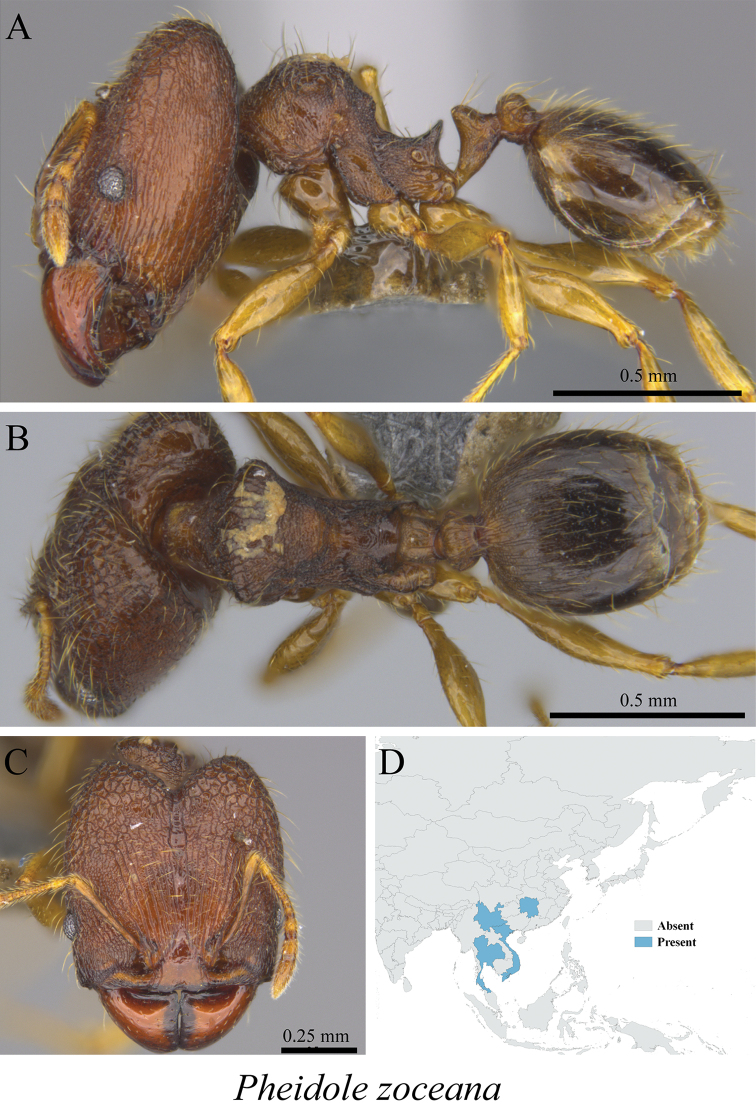
*Pheidole zoceana* major worker (MCZ-ENT00760016) **A** mesosoma in profile view **B** mesosoma in dorsal view **C** head in front view **D** global distribution map.

**Figure 97.**
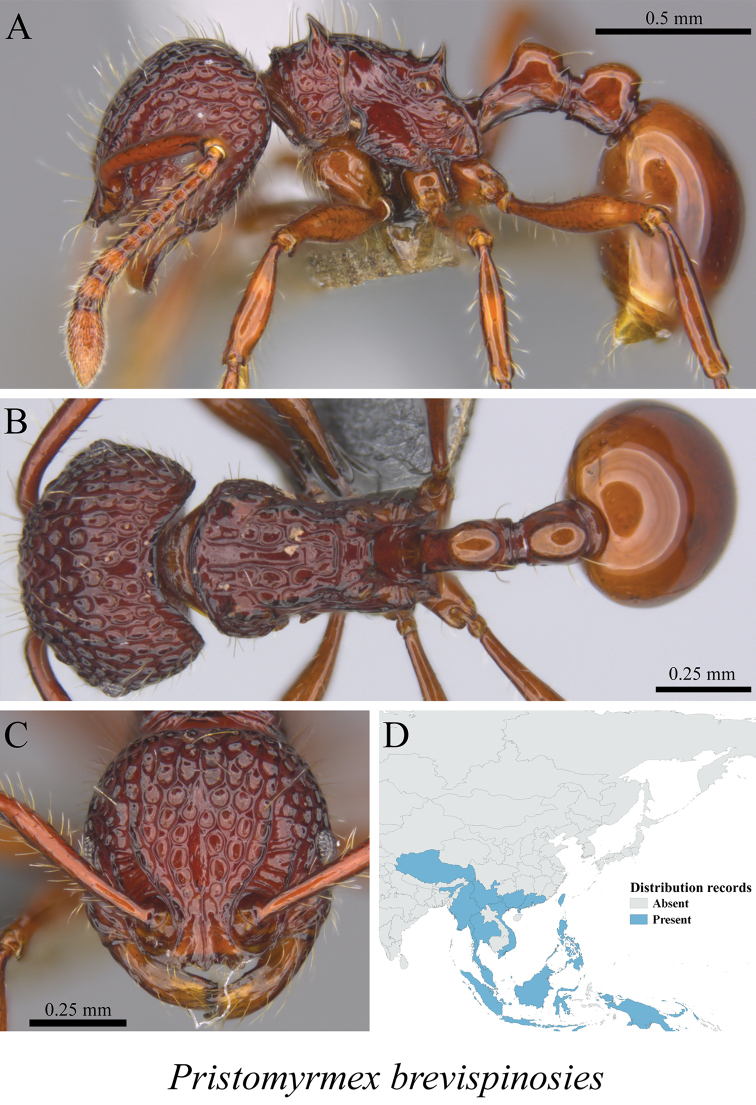
*Pristomyrmex brevispinosus* worker (MCZ-ENT00763505) **A** mesosoma in profile view **B** mesosoma in dorsal view **C** head in front view **D** global distribution map.

**Figure 98.**
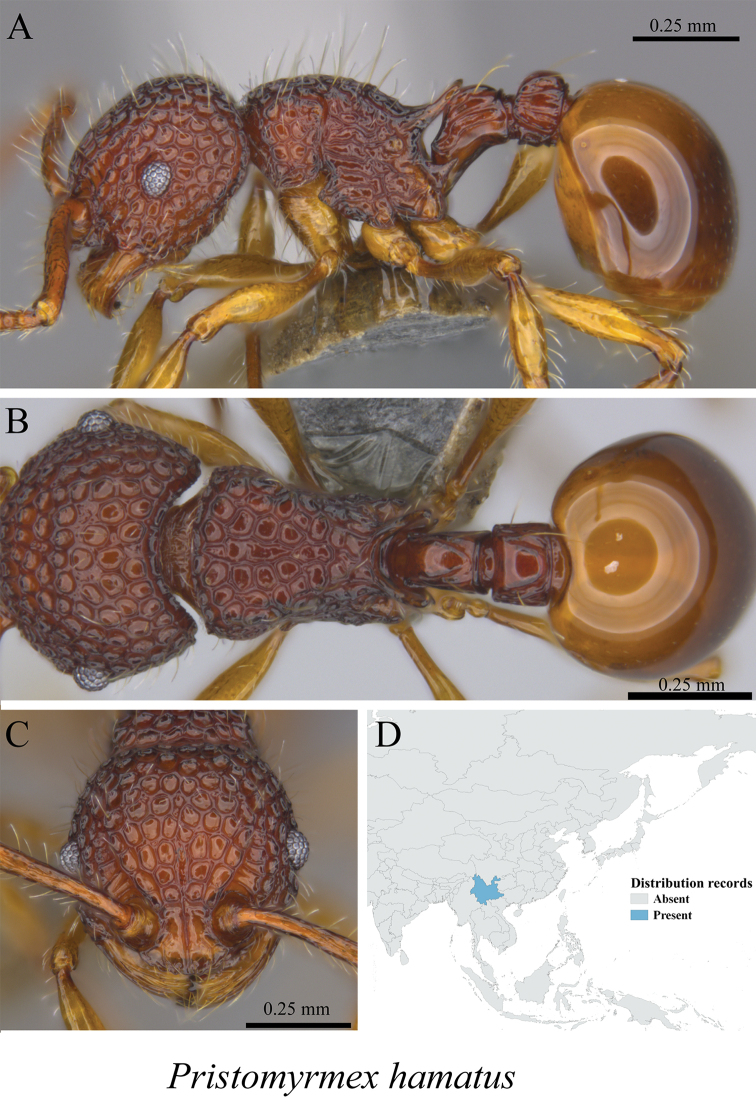
*Pristomyrmex hamatus* worker (MCZ-ENT00763502) **A** mesosoma in profile view **B** mesosoma in dorsal view **C** head in front view **D** global distribution map.

**Figure 99.**
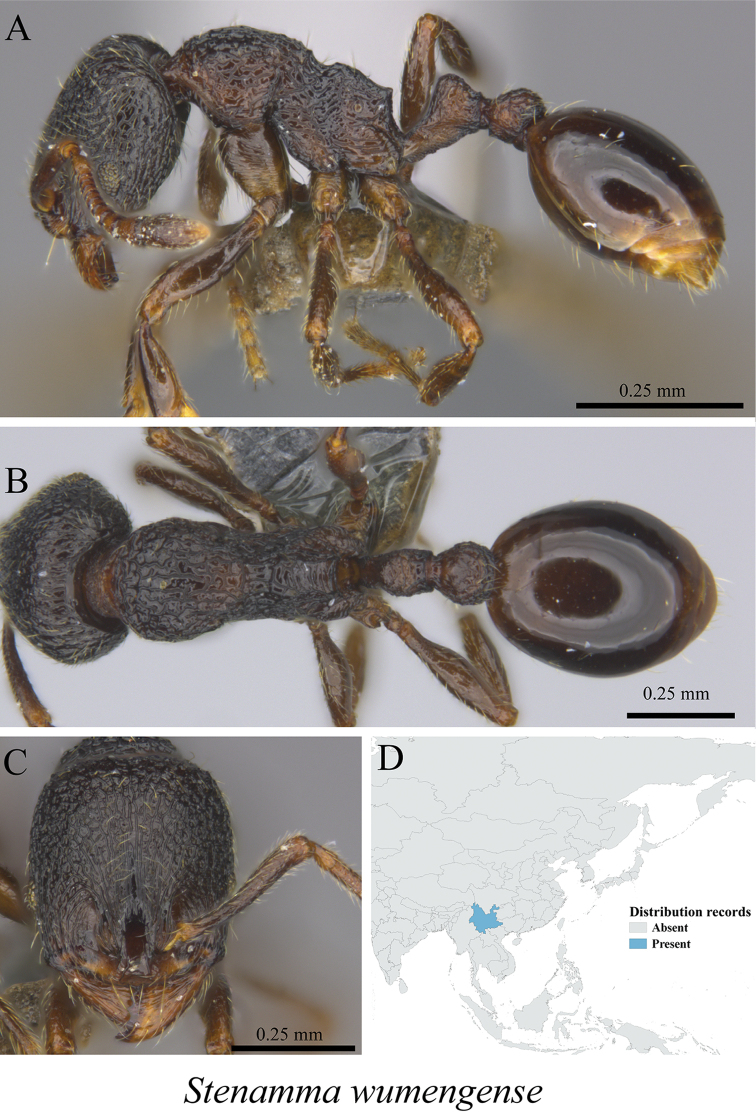
*Stenamma wumengense* worker (MCZ-ENT00762907) **A** mesosoma in profile view **B** mesosoma in dorsal view **C** head in front view **D** global distribution map.

**Figure 100.**
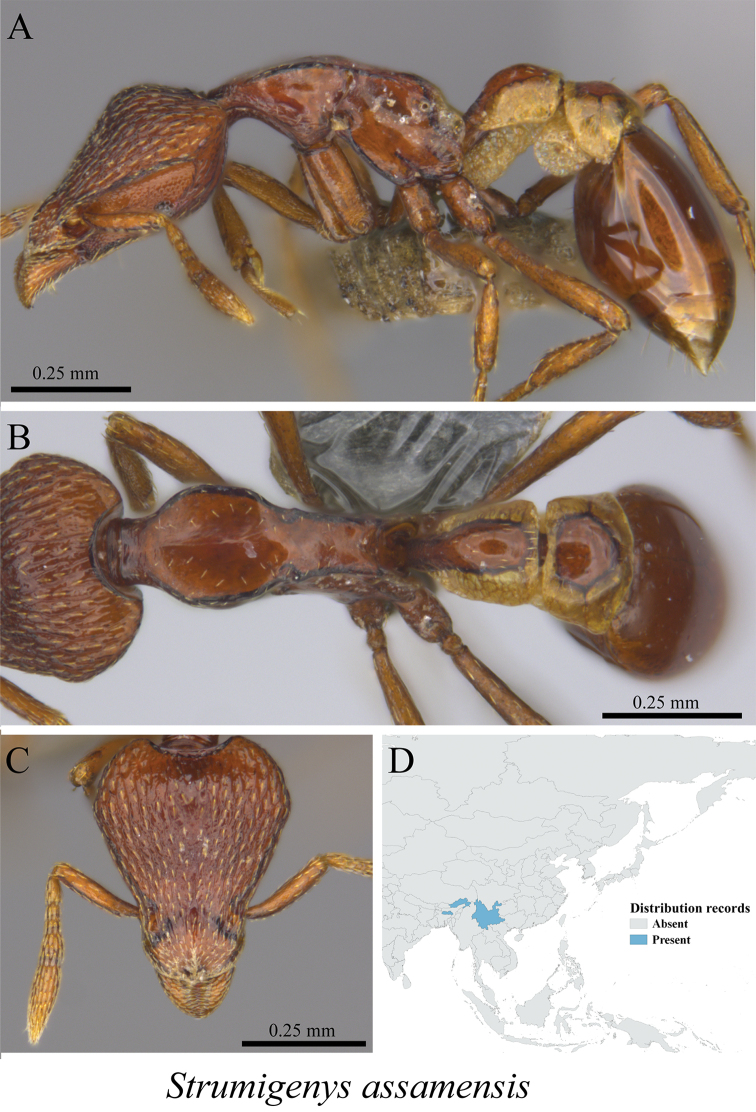
*Strumigenys assamensis* worker (MCZ-ENT00759885) **A** mesosoma in profile view **B** mesosoma in dorsal view **C** head in front view **D** global distribution map.

**Figure 101.**
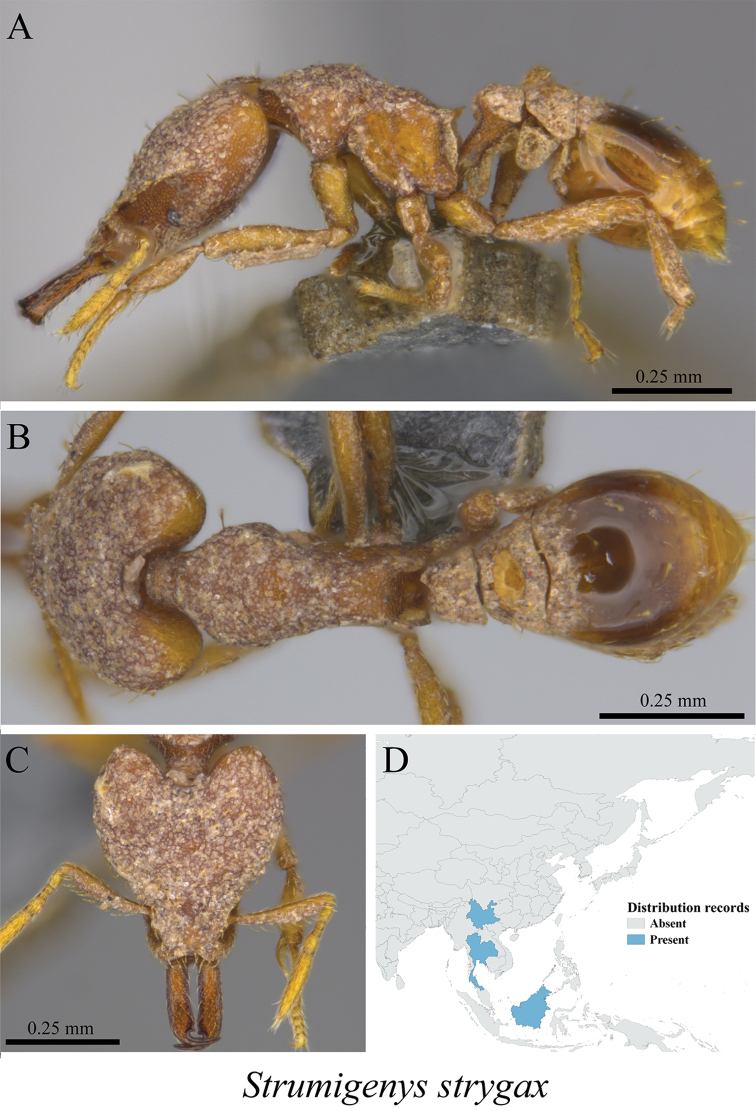
*Strumigenys strygax* worker (MCZ-ENT00763507) **A** mesosoma in profile view **B** mesosoma in dorsal view **C** head in front view **D** global distribution map.

**Figure 102.**
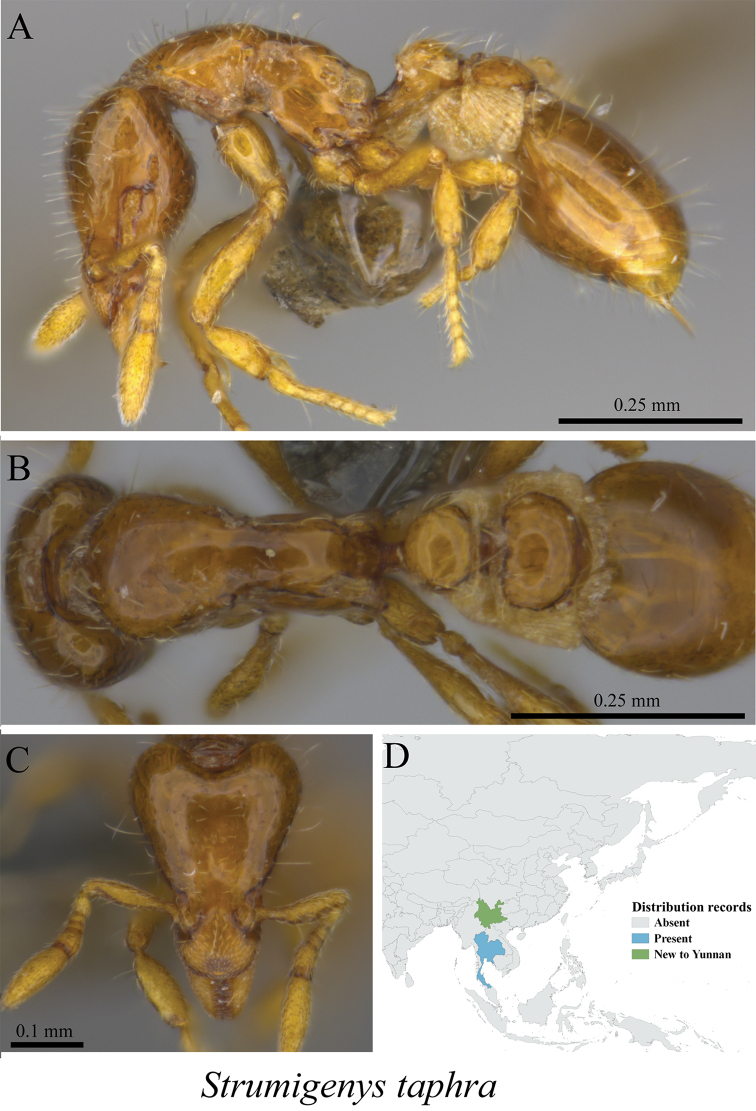
*Strumigenys taphra* worker (MCZ-ENT00759758, new to China) **A** mesosoma in profile view **B** mesosoma in dorsal view **C** head in front view **D** global distribution map.

**Figure 103.**
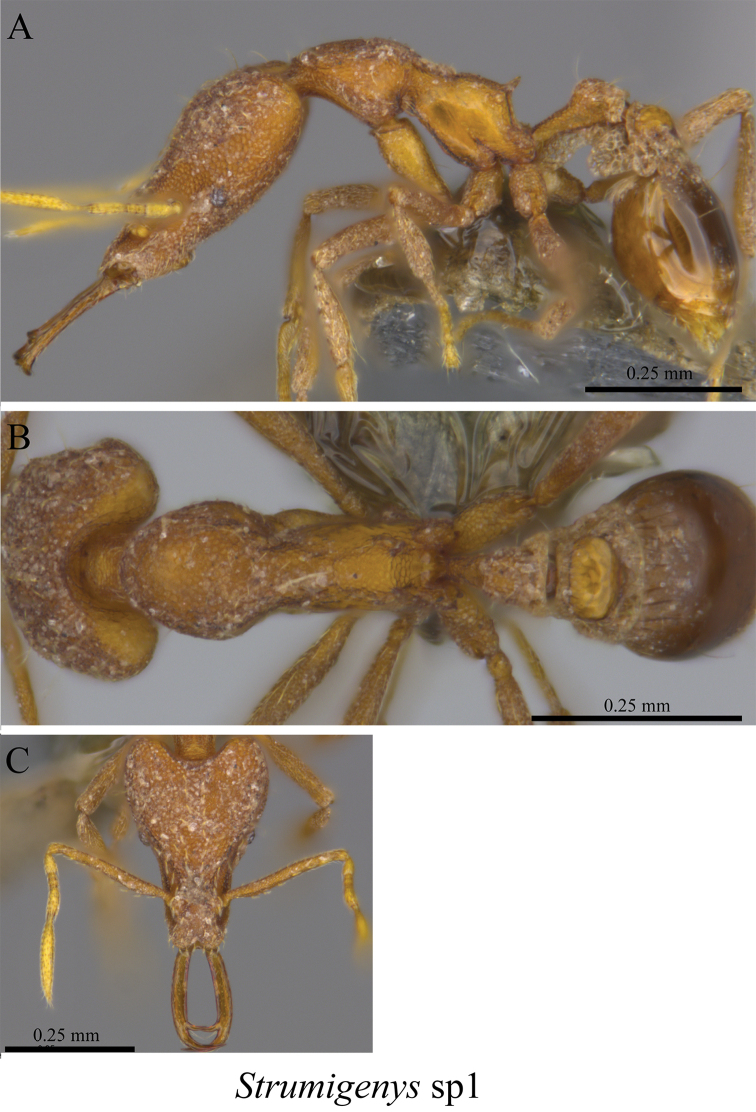
*Strumigenys* sp. clm01 worker (MCZ-ENT00763511) **A** mesosoma in profile view **B** mesosoma in dorsal view **C** head in front view.

**Figure 104.**
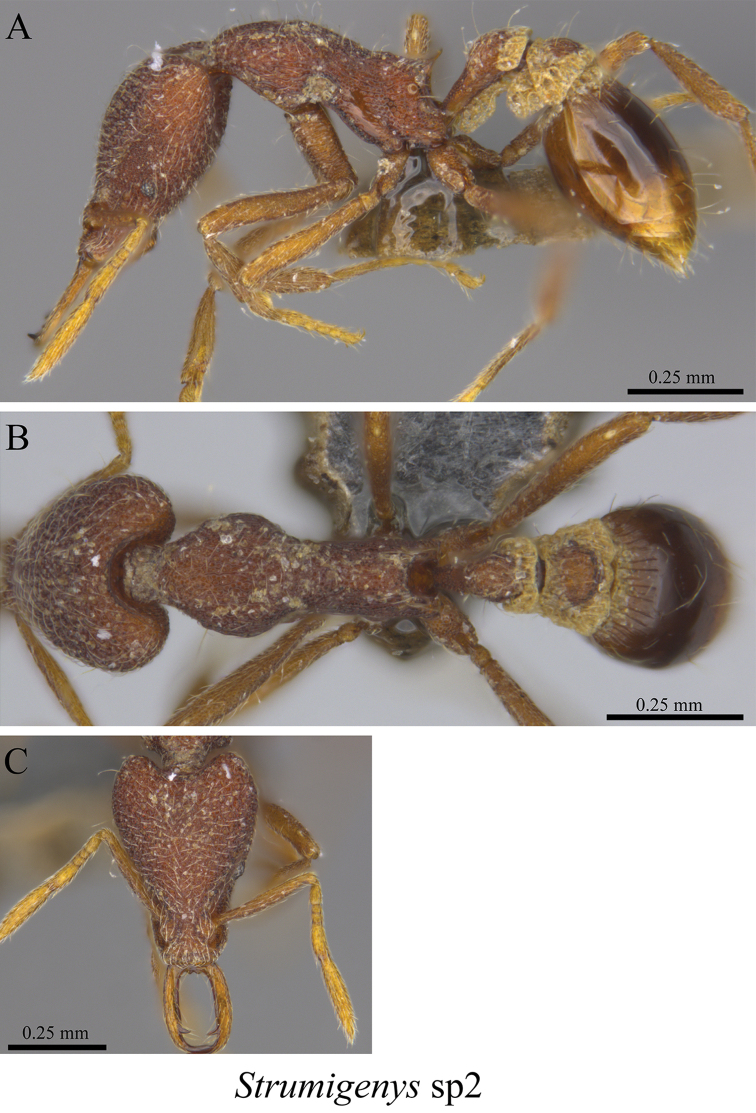
*Strumigenys* sp. clm02 worker (MCZ-ENT00759897) **A** mesosoma in profile view **B** mesosoma in dorsal view **C** head in front view.

**Figure 105.**
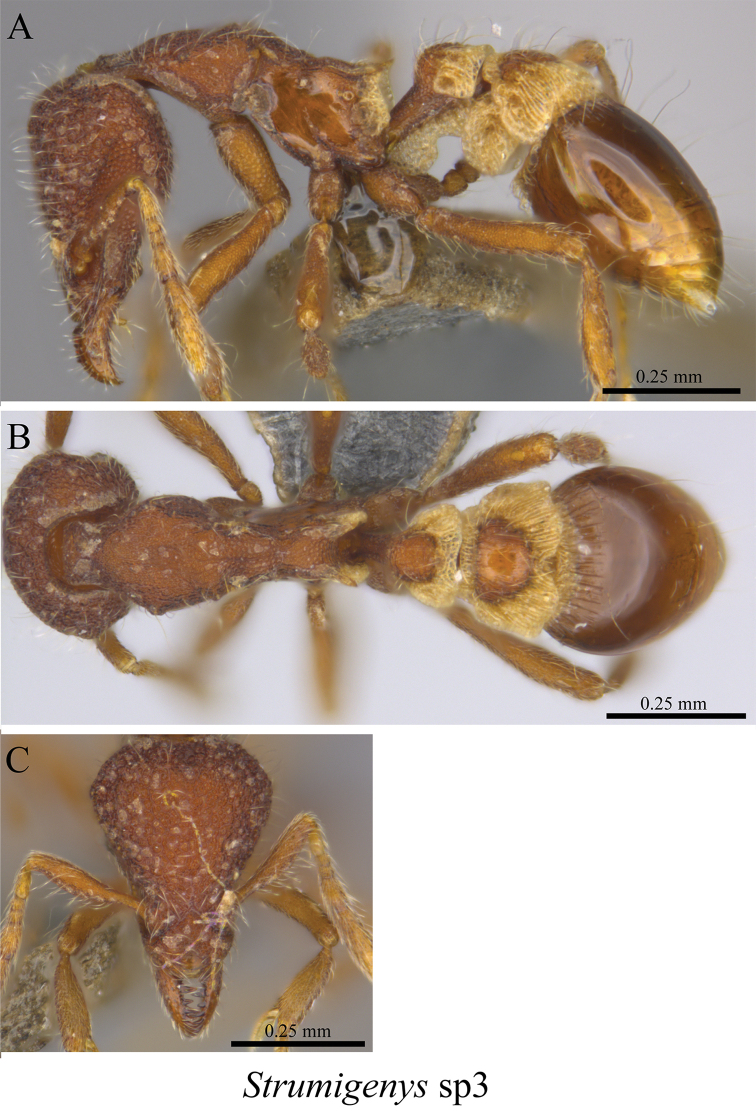
*Strumigenys* sp. clm03 worker (MCZ-ENT00759991) **A** mesosoma in profile view **B** mesosoma in dorsal view **C** head in front view.

**Figure 106.**
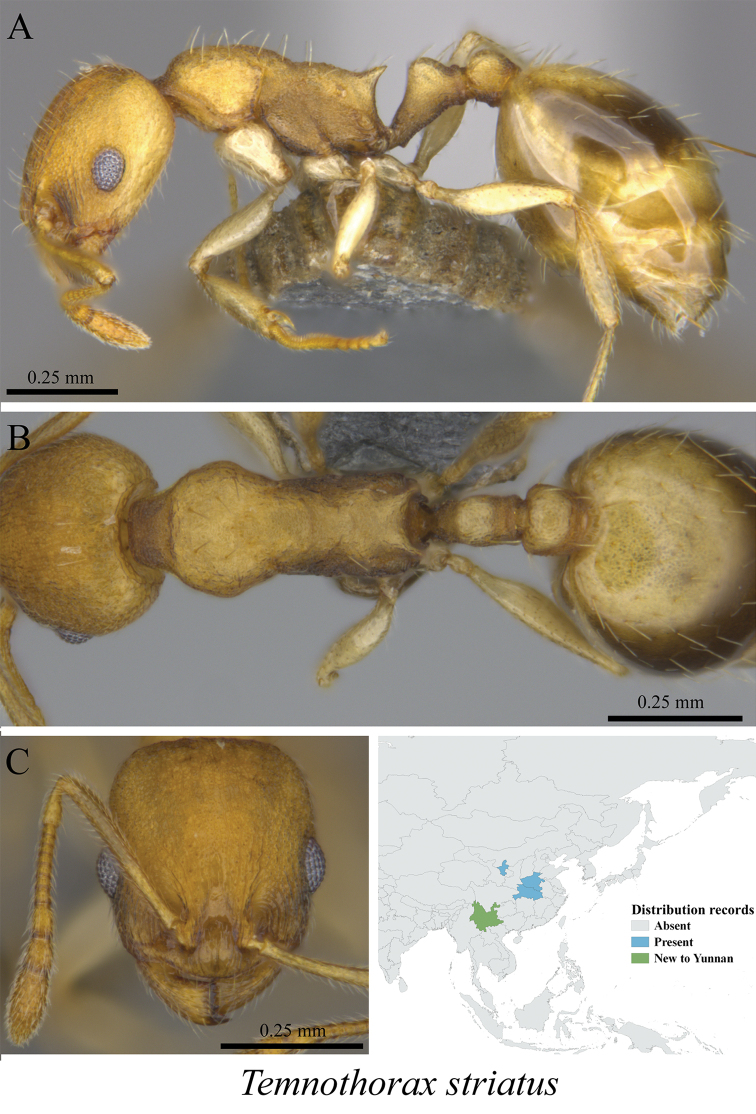
*Temnothorax striatus* worker (MCZ-ENT00759763, new to Yunnan) **A** mesosoma in profile view **B** mesosoma in dorsal view **C** head in front view **D** global distribution map.

**Figure 107.**
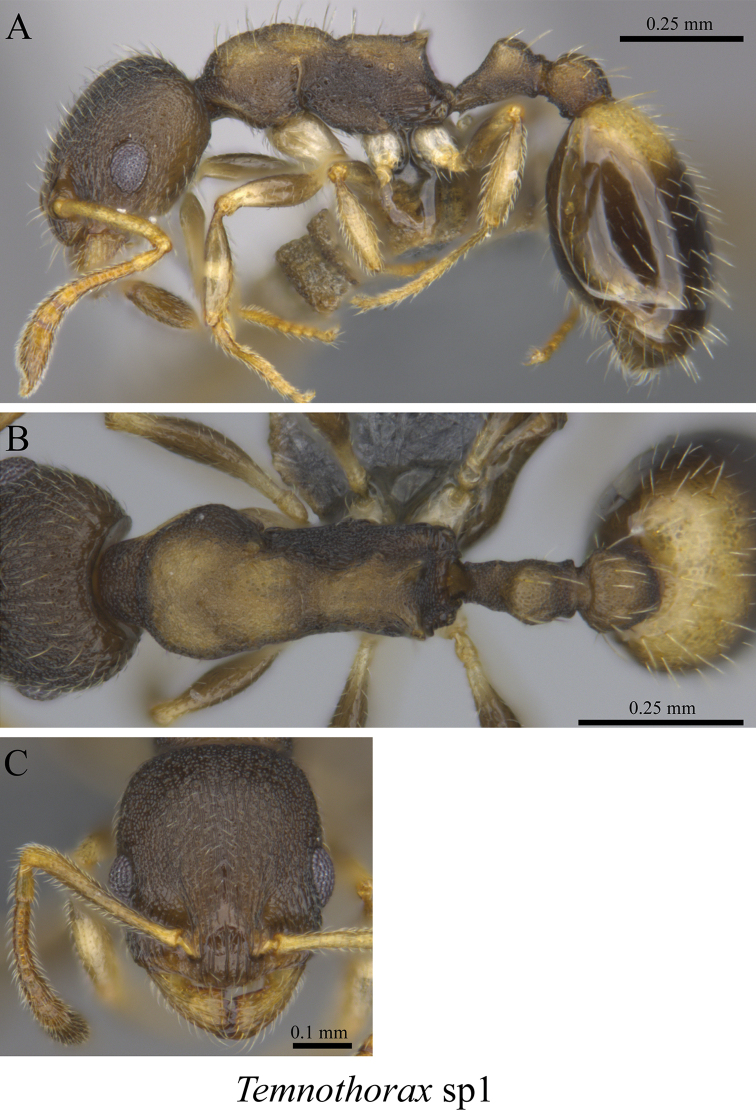
*Temnothorax* sp. clm01 worker (MCZ-ENT00759977) **A** mesosoma in profile view **B** mesosoma in dorsal view **C** head in front view.

**Figure 108.**
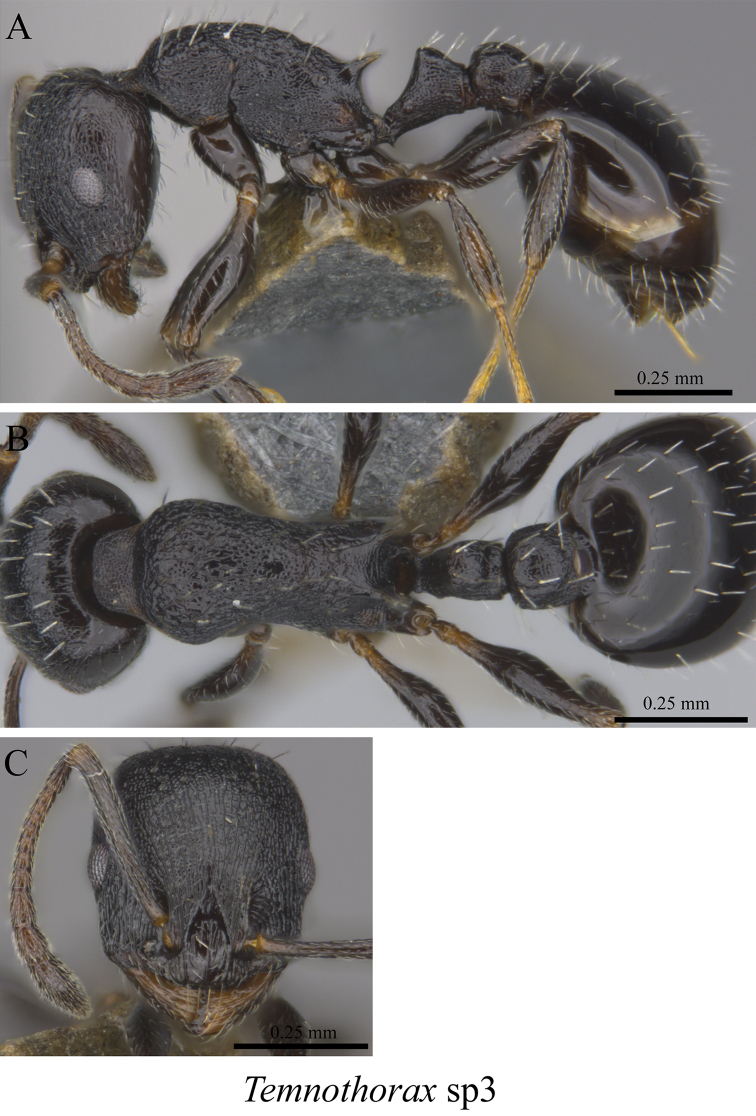
*Temnothorax* sp. clm03 worker (MCZ-ENT00763303) **A** mesosoma in profile view **B** mesosoma in dorsal view **C** head in front view.

**Figure 109.**
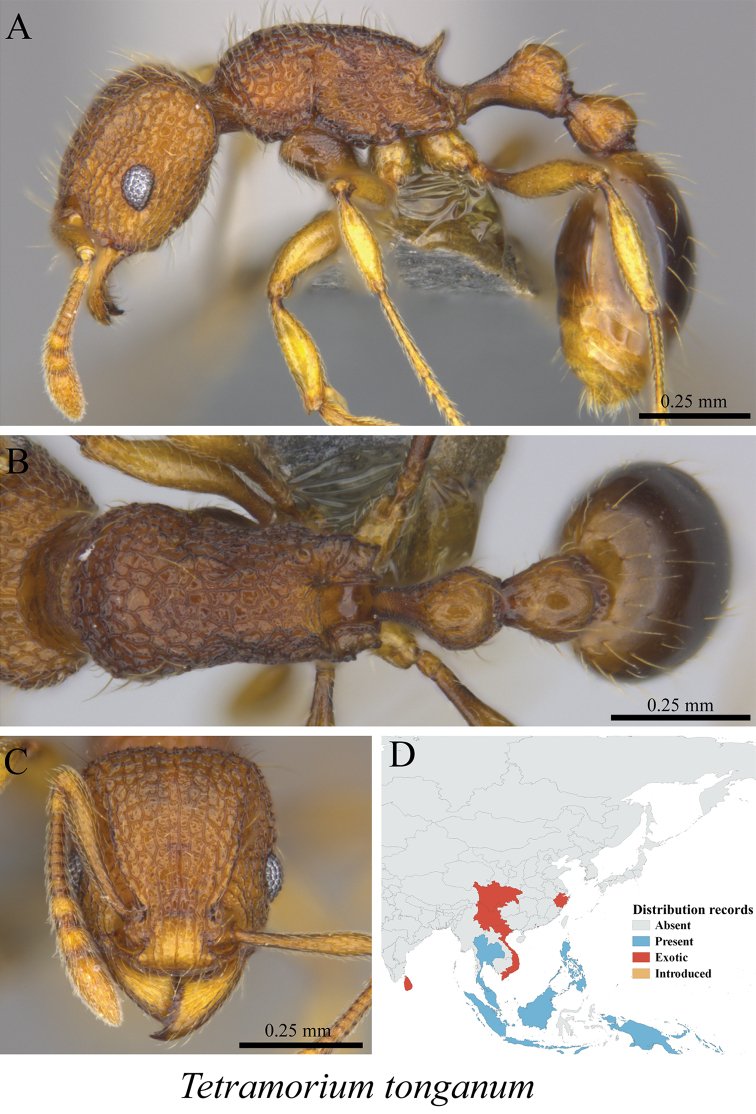
*Tetramorium tonganum* worker (MCZ-ENT00764651) **A** mesosoma in profile view **B** mesosoma in dorsal view **C** head in front view **D** global distribution map.

**Figure 110.**
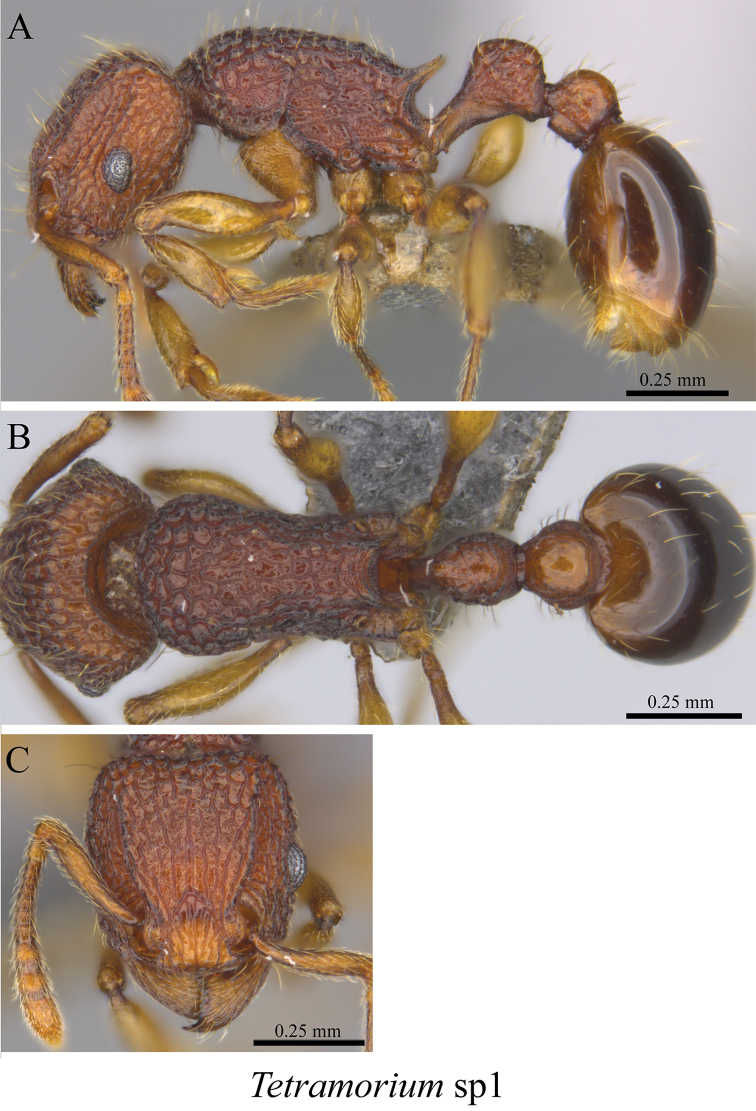
*Tetramorium* sp. clm01 worker (MCZ-ENT00759754) **A** mesosoma in profile view **B** mesosoma in dorsal view **C** head in front view.

**Figure 111.**
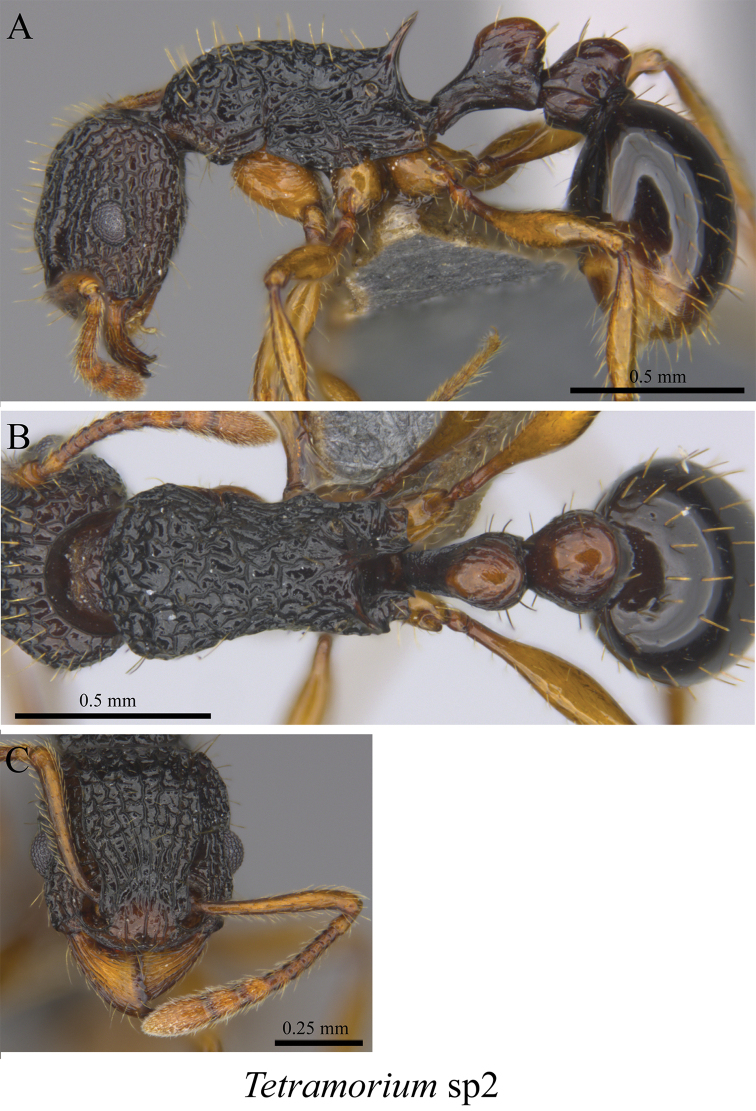
*Tetramorium* sp. clm02 worker (MCZ-ENT00763454) **A** mesosoma in profile view **B** mesosoma in dorsal view **C** head in front view.

**Figure 112.**
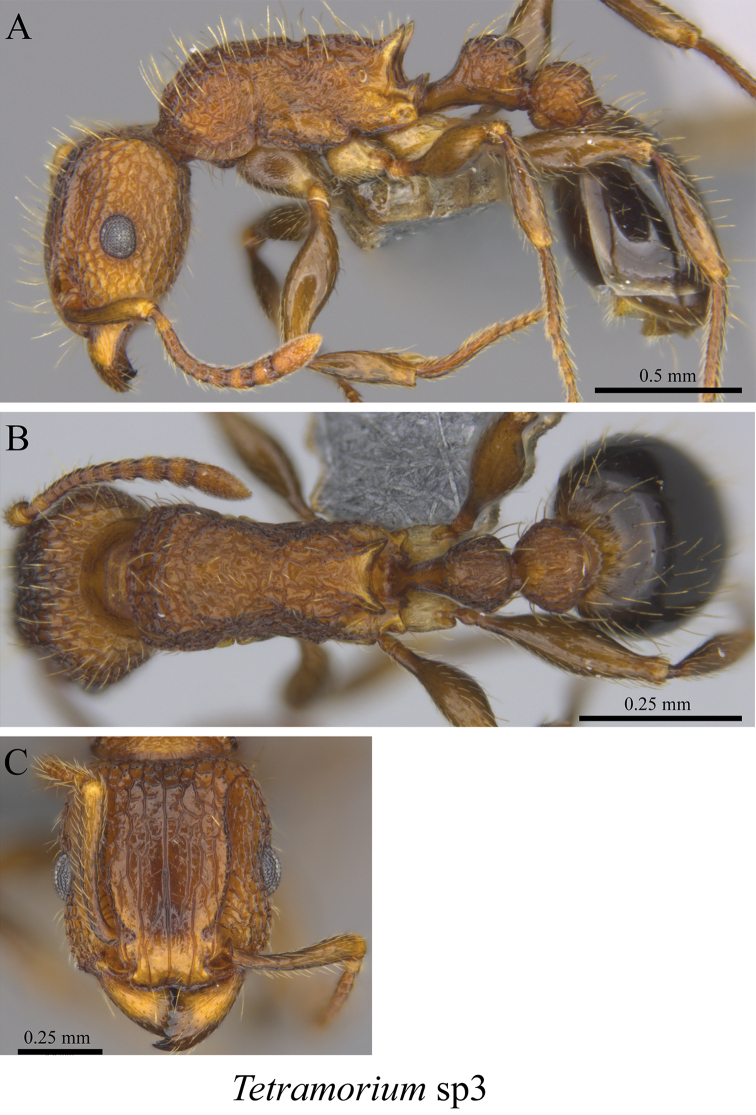
*Tetramorium* sp. clm03 worker (MCZ-ENT00760040) **A** mesosoma in profile view **B** mesosoma in dorsal view **C** head in front view.

**Figure 113.**
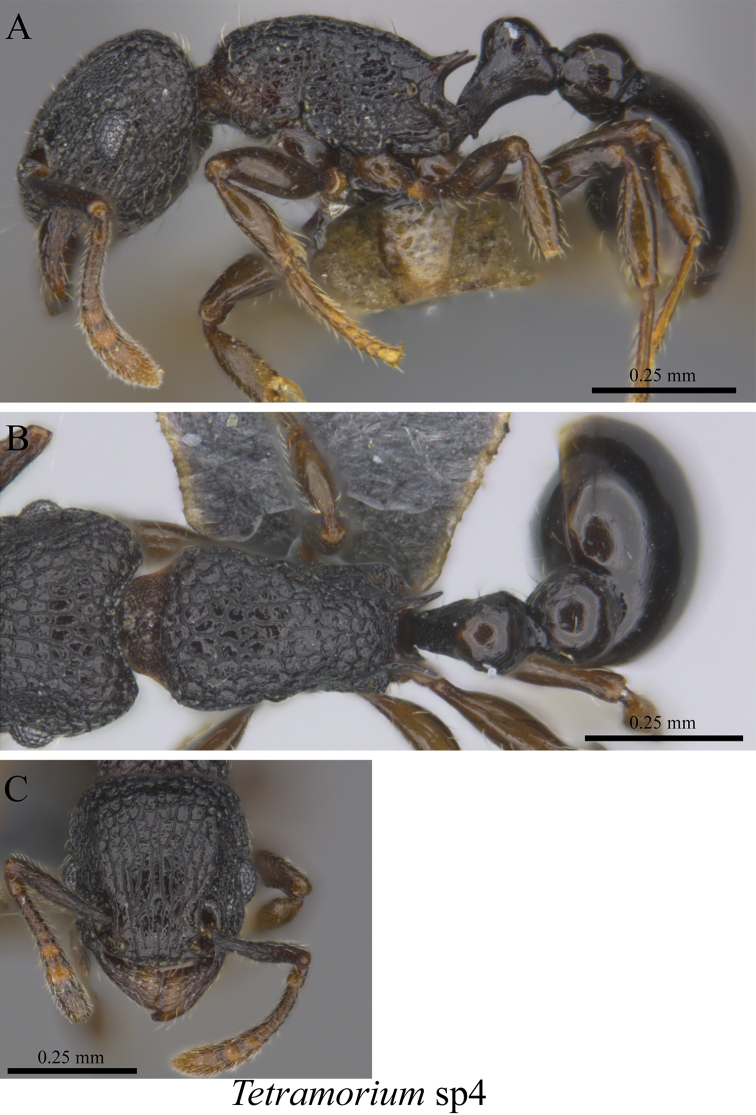
*Tetramorium* sp. clm04 worker (MCZ-ENT00759856) **A** mesosoma in profile view **B** mesosoma in dorsal view **C** head in front view.

**Figure 114.**
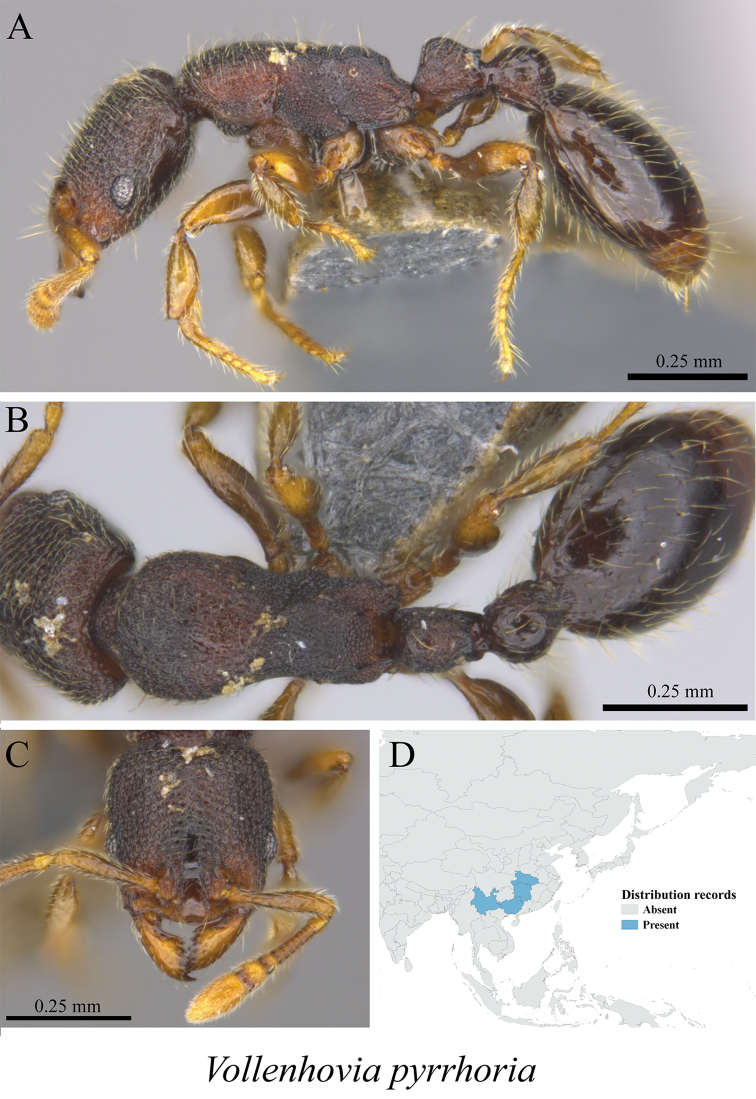
*Vollenhovia pyrrhoria* worker (MCZ-ENT00759854) **A** mesosoma in profile view **B** mesosoma in dorsal view **C** head in front view **D** global distribution map.

**Figure 115.**
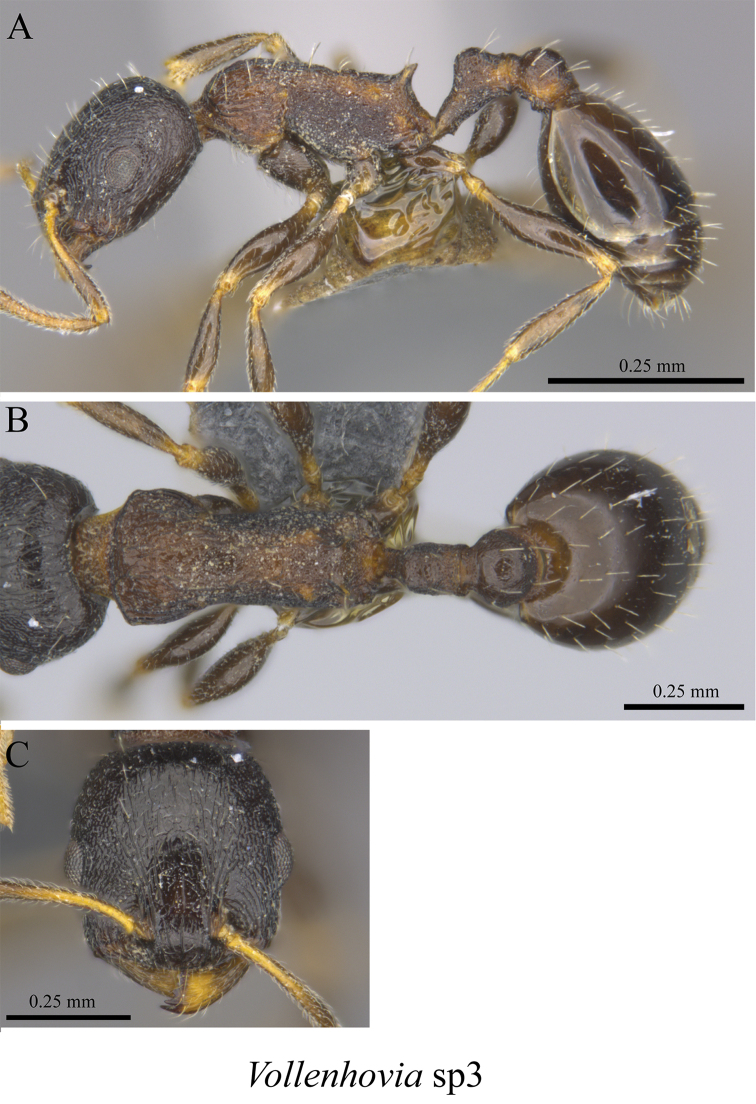
*Vollenhovia* sp. clm03 worker (MCZ-ENT00764617) **A** mesosoma in profile view **B** mesosoma in dorsal view **C** head in front view.

**Figure 116.**
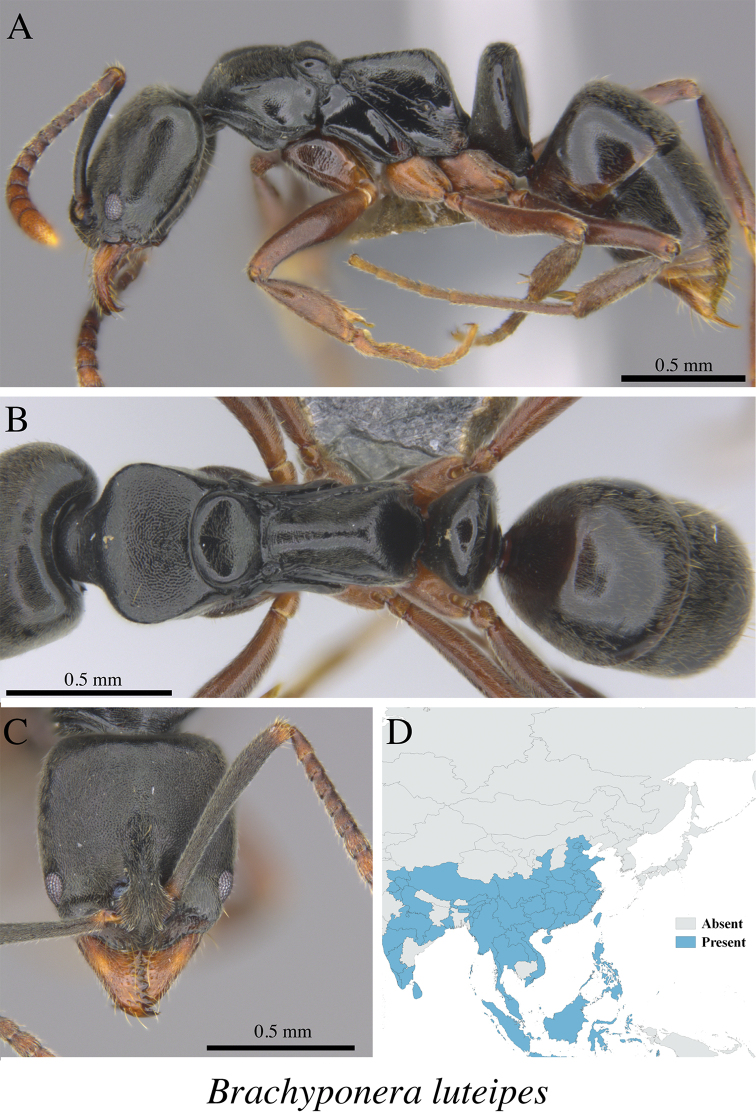
*Brachyponera luteipes* worker (MCZ-ENT00759752) **A** mesosoma in profile view **B** mesosoma in dorsal view **C** head in front view **D** global distribution map.

**Figure 117.**
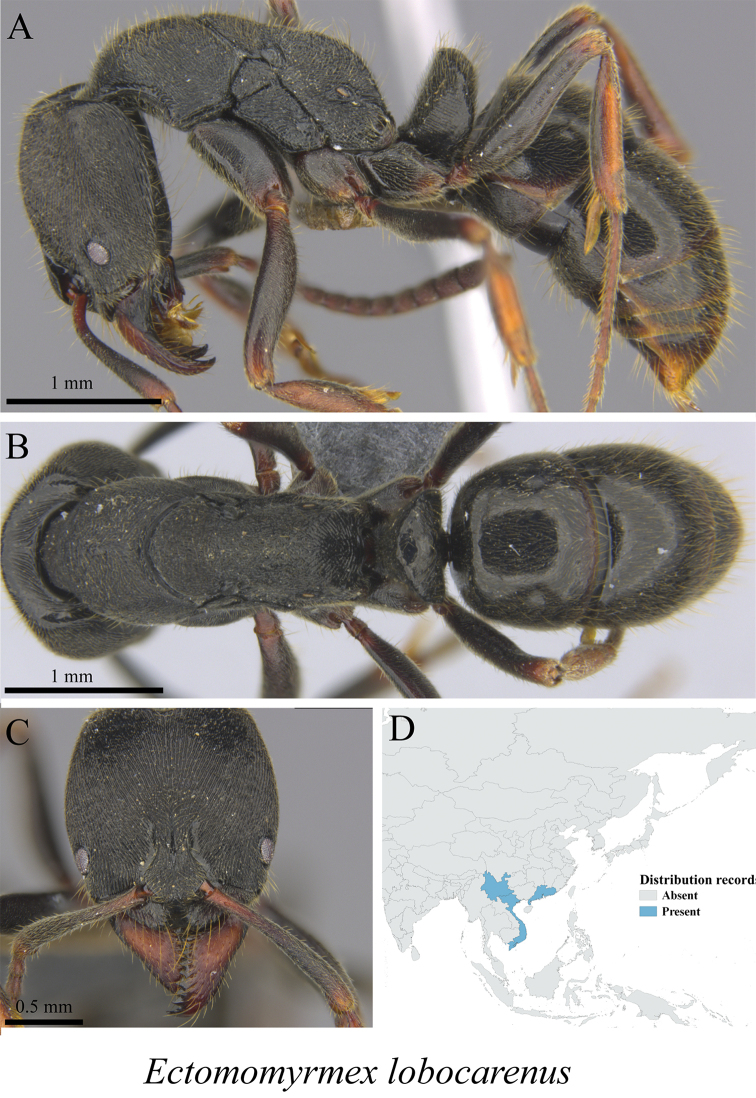
*Ectomomyrmex lobocarenus* worker (MCZ-ENT00759748) **A** mesosoma in profile view **B** mesosoma in dorsal view **C** head in front view **D** global distribution map.

**Figure 118.**
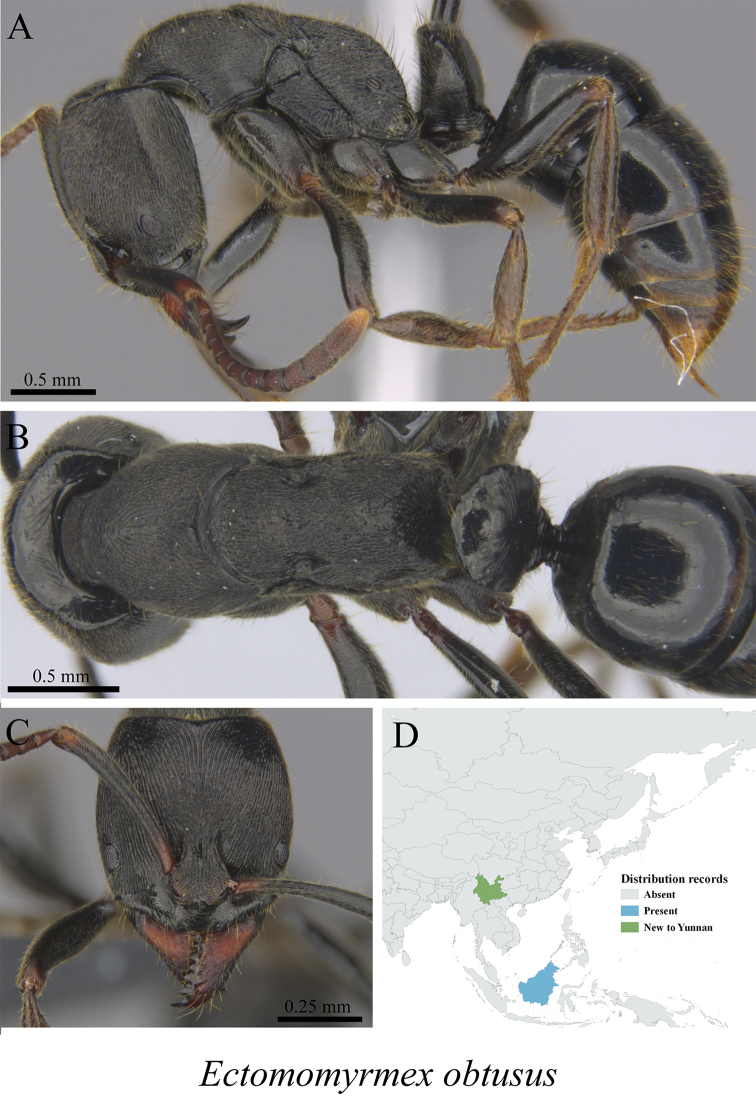
*Ectomomyrmex obtusus* worker (MCZ-ENT00759859, new to China) **A** mesosoma in profile view **B** mesosoma in dorsal view **C** head in front view **D** global distribution map.

**Figure 119.**
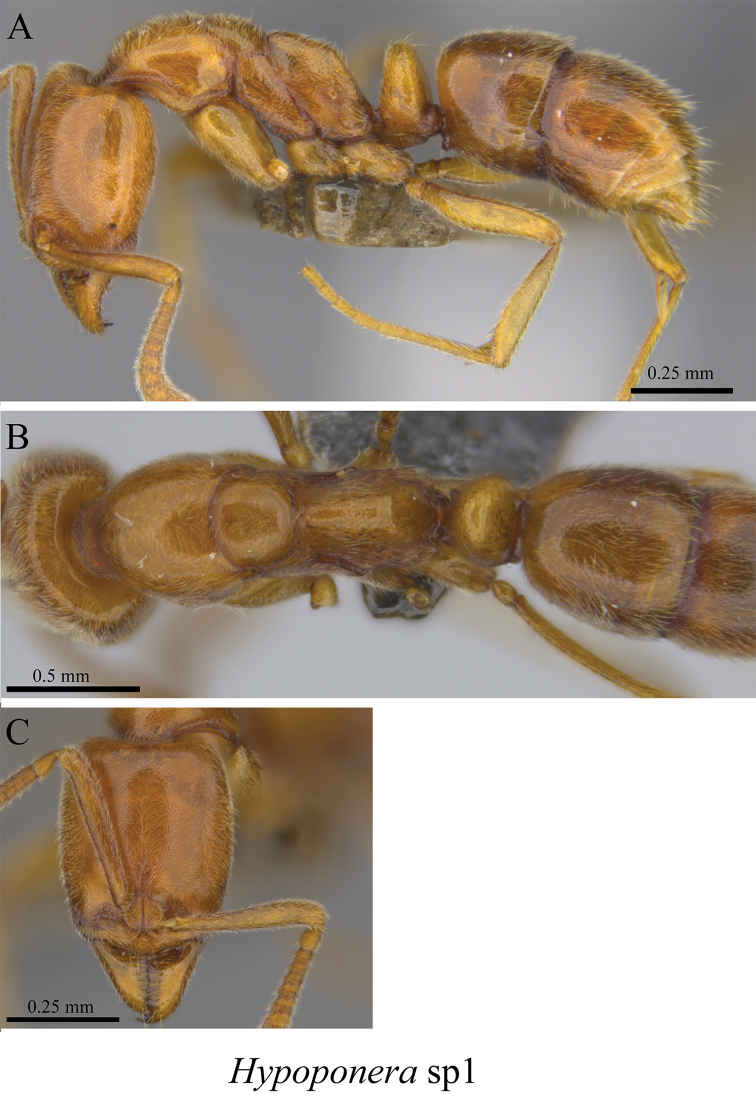
*Hypoponera* sp. clm01 worker (MCZ-ENT00759780) **A** mesosoma in profile view **B** mesosoma in dorsal view **C** head in front view.

**Figure 120.**
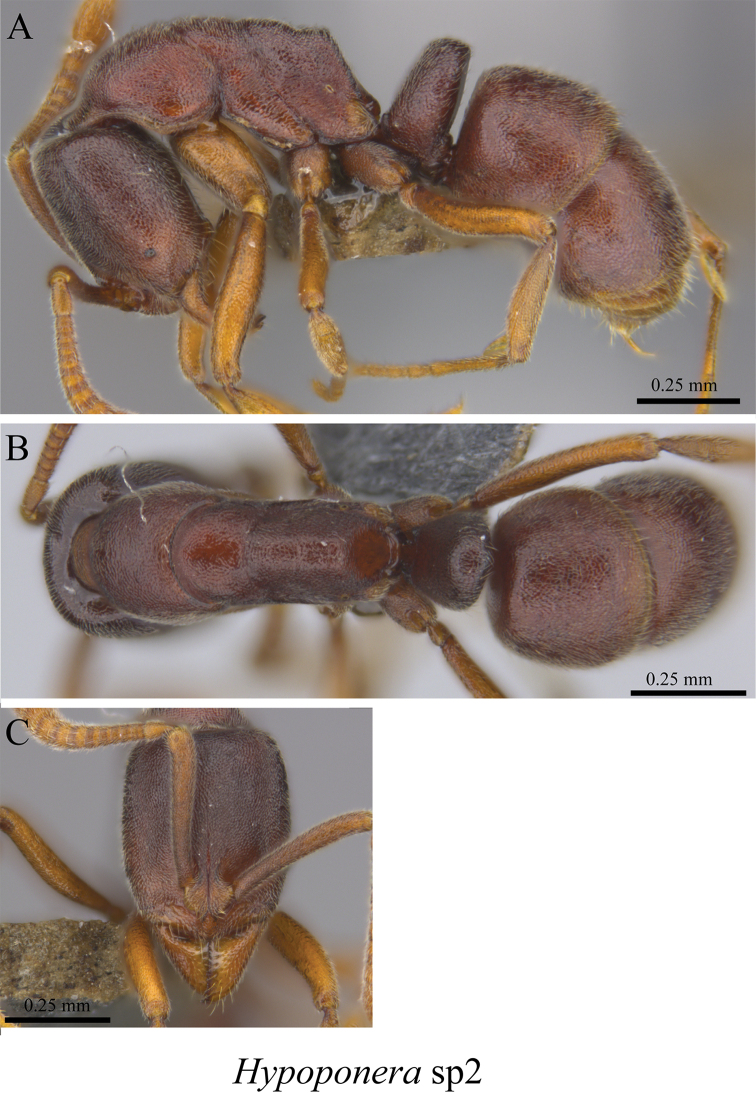
*Hypoponera* sp. clm02 worker (MCZ-ENT00759849) **A** mesosoma in profile view **B** mesosoma in dorsal view **C** head in front view.

**Figure 121.**
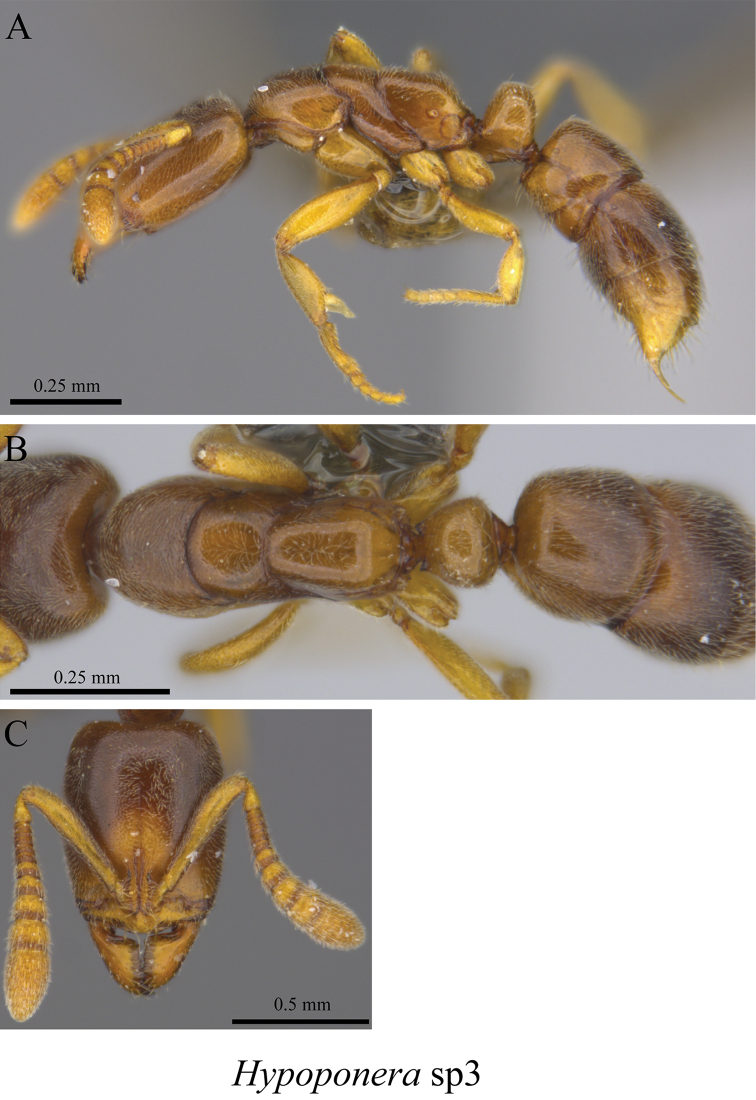
*Hypoponera* sp. clm03 worker (MCZ-ENT00759808) **A** mesosoma in profile view **B** mesosoma in dorsal view **C** head in front view.

**Figure 122.**
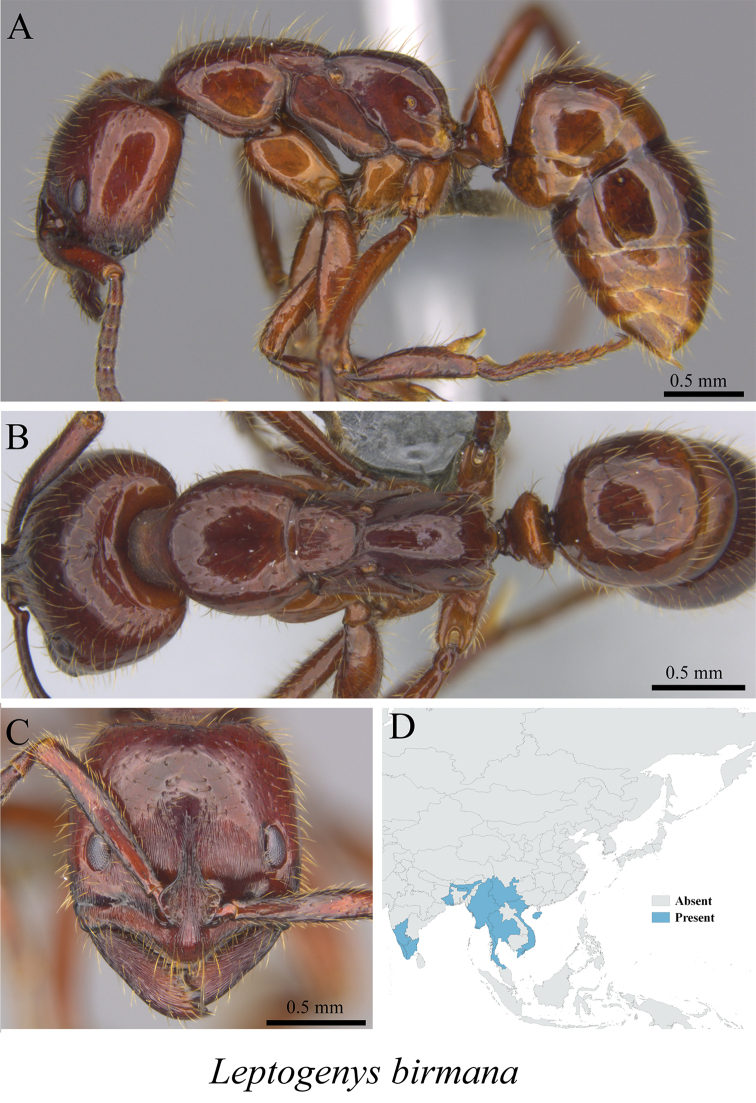
*Leptogenys birmana* worker (MCZ-ENT00763178) **A** mesosoma in profile view **B** mesosoma in dorsal view **C** head in front view **D** global distribution map.

**Figure 123.**
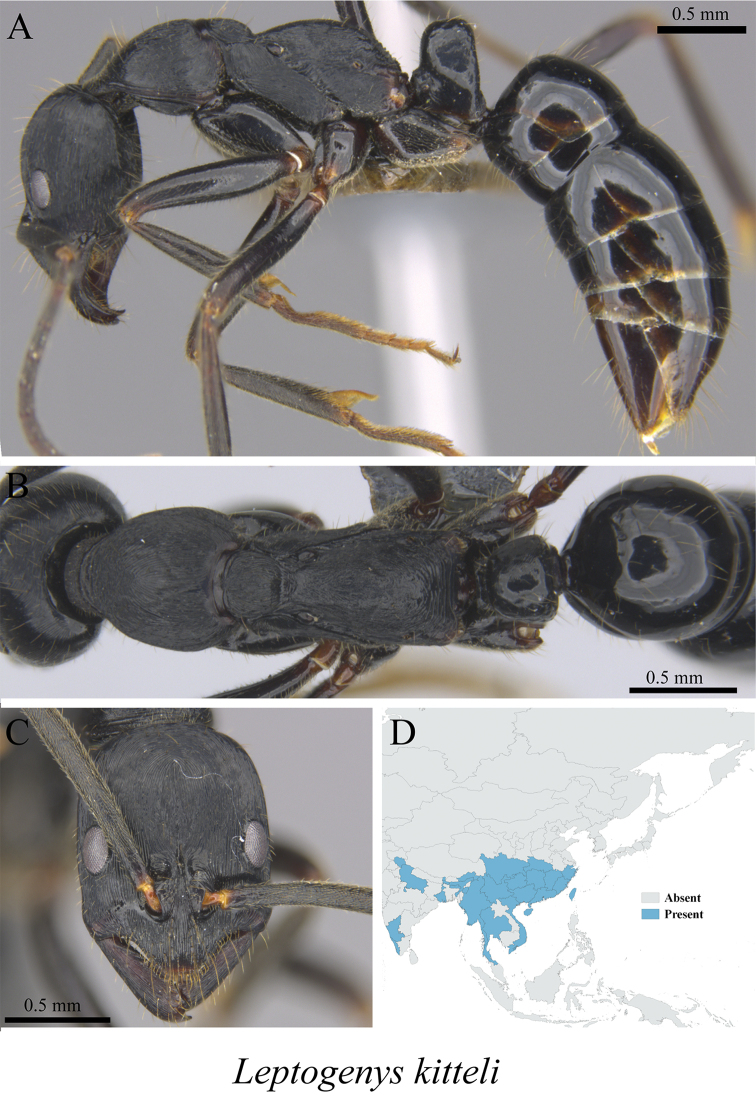
*Leptogenys kitteli* worker (MCZ-ENT00763321). **A** mesosoma in profile view **B** mesosoma in dorsal view **C** head in front view **D** global distribution map.

**Figure 124.**
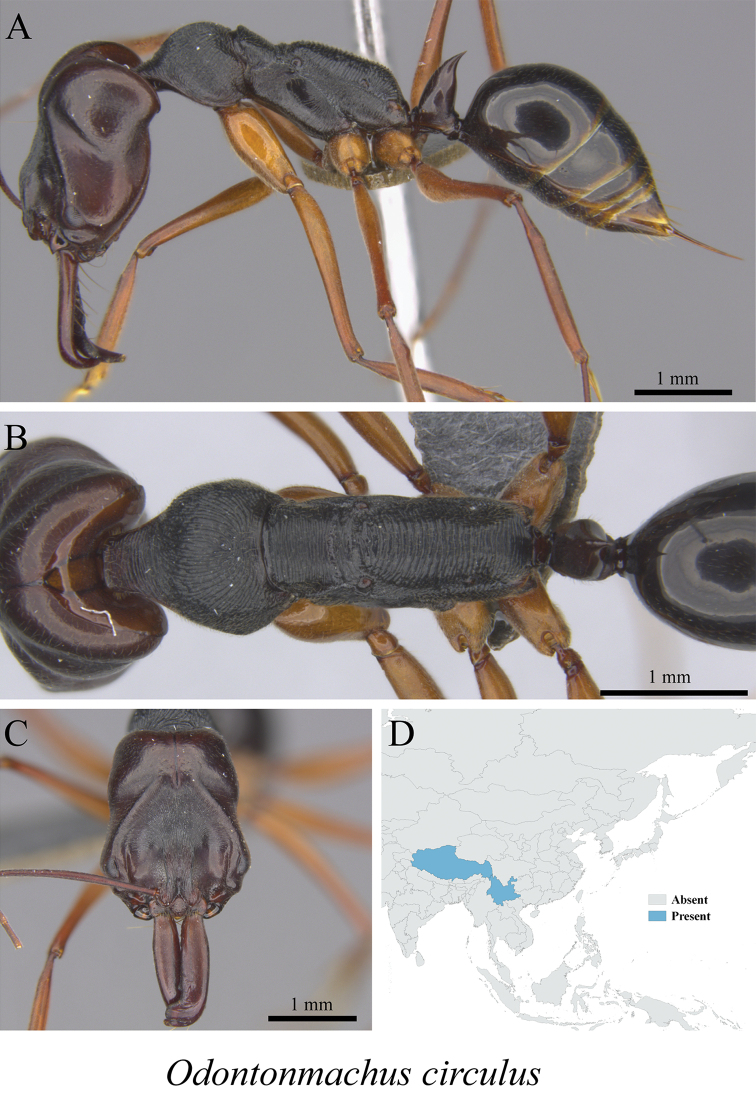
*Odontomachus circulus* worker (MCZ-ENT00762856). **A** mesosoma in profile view **B** mesosoma in dorsal view **C** head in front view **D** global distribution map.

**Figure 125.**
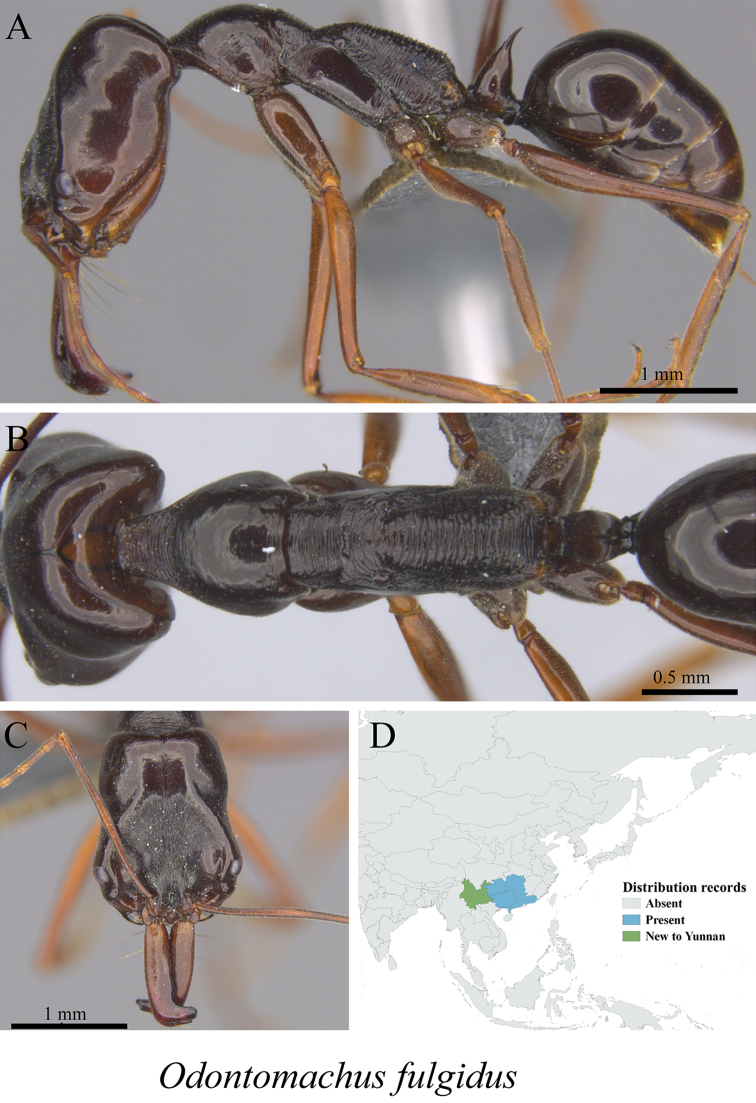
*Odontomachus fulgidus* worker (MCZ-ENT00760009, new to Yunnan) **A** mesosoma in profile view **B** mesosoma in dorsal view **C** head in front view **D** global distribution map.

**Figure 126.**
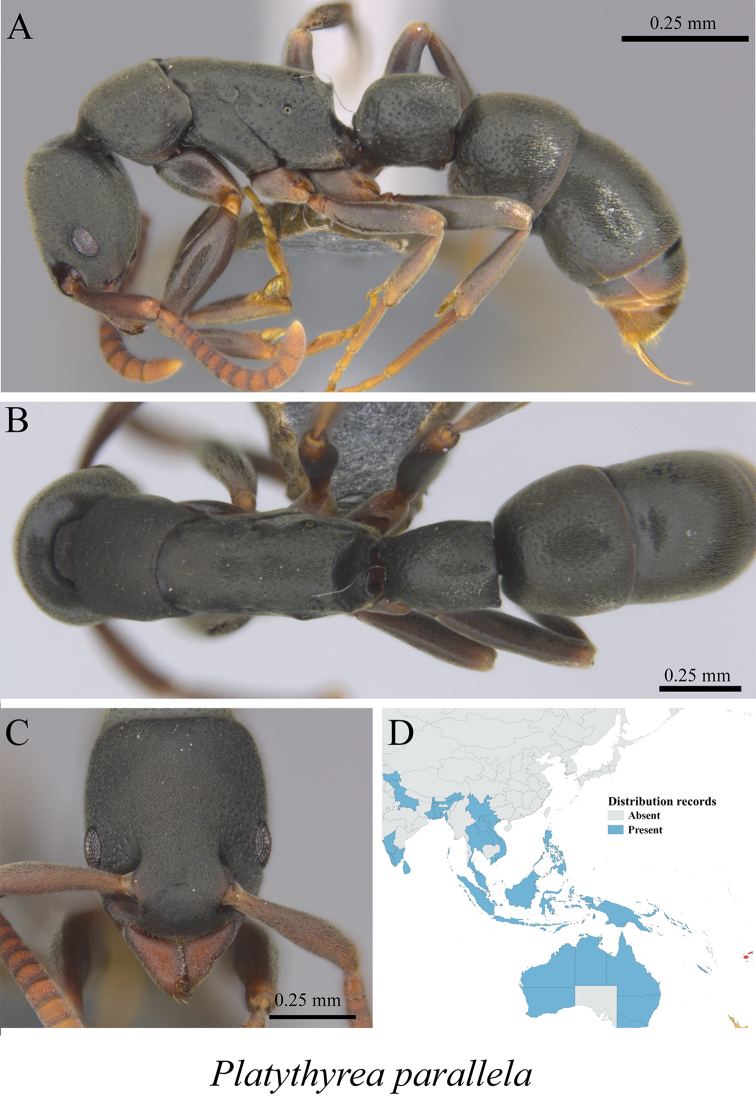
*Platythyrea parallela* worker (MCZ-ENT00763657) **A** mesosoma in profile view **B** mesosoma in dorsal view **C** head in front view **D** global distribution map.

**Figure 127.**
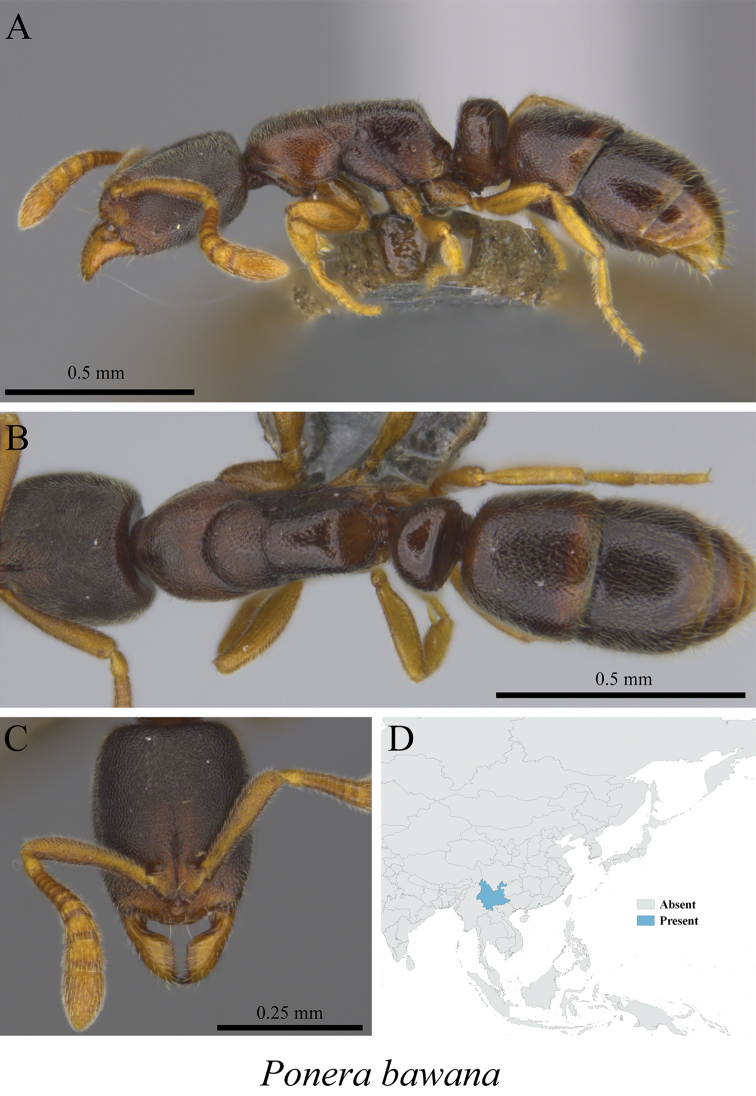
*Ponera bawana* worker (MCZ-ENT00759807) **A** mesosoma in profile view **B** mesosoma in dorsal view **C** head in front view **D** global distribution map.

**Figure 128.**
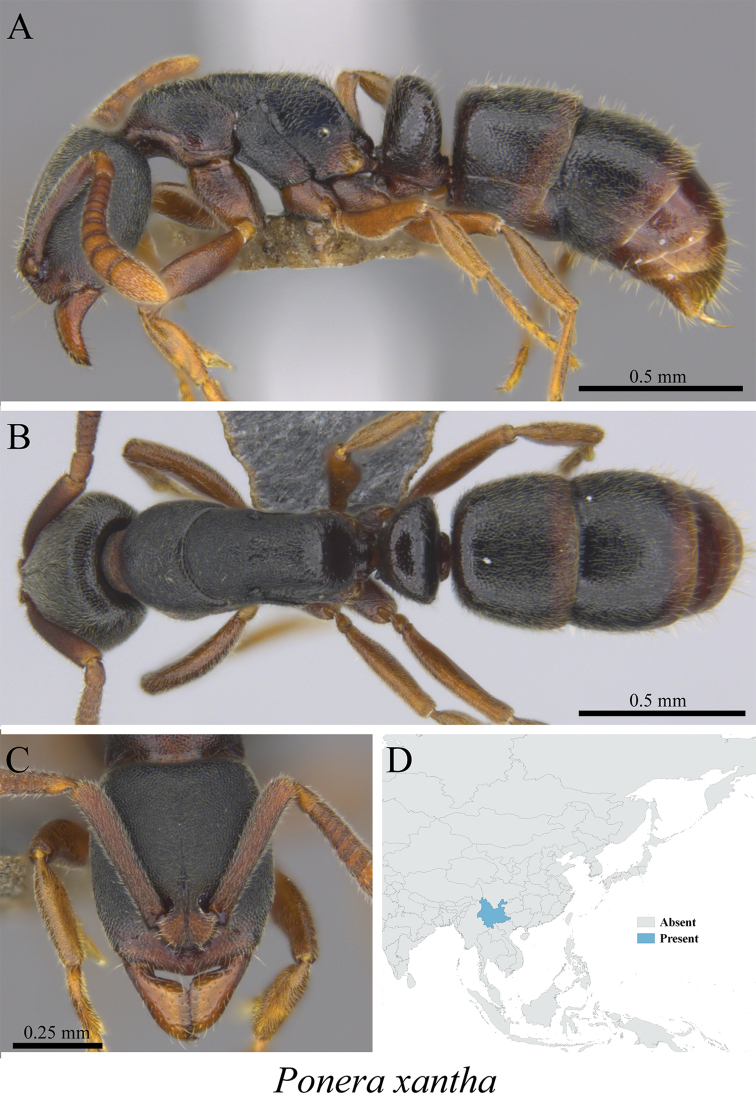
*Ponera xantha* worker (MCZ-ENT00759845) **A** mesosoma in profile view **B** mesosoma in dorsal view **C** head in front view **D** global distribution map.

**Figure 129.**
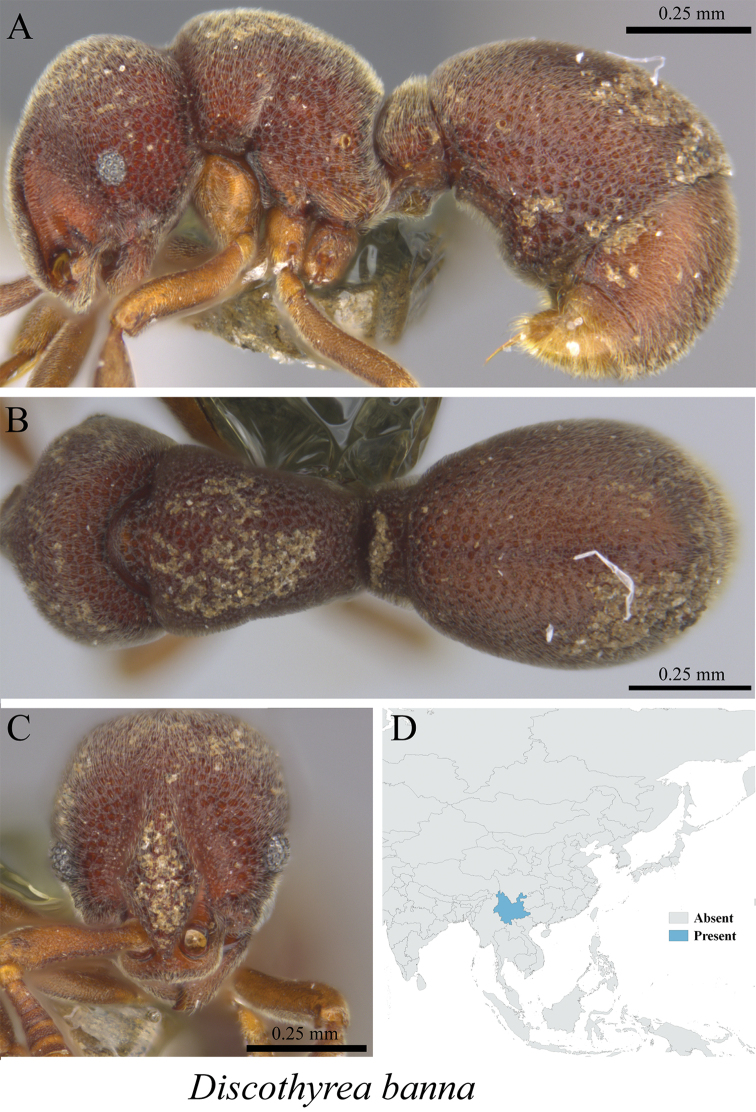
*Discothyrea banna* worker (MCZ-ENT00759809) **A** mesosoma in profile view **B** mesosoma in dorsal view **C** head in front view **D** global distribution map.

**Figure 130.**
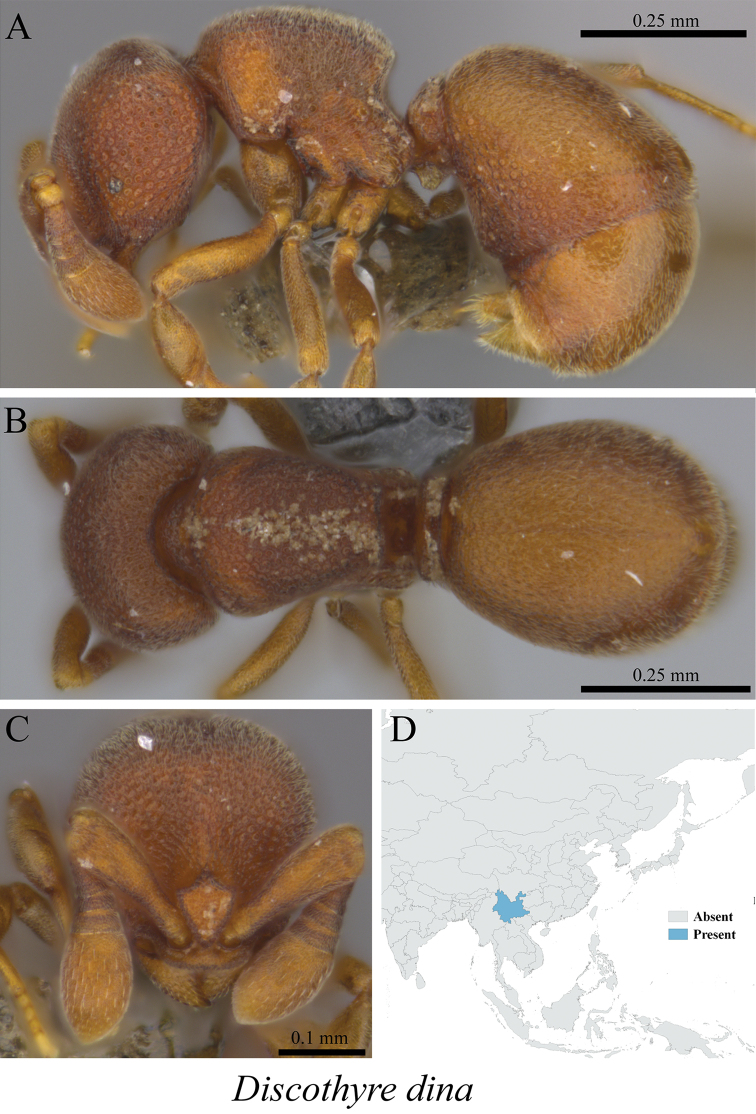
*Discothyrea diana* worker (MCZ-ENT00759806) **A** mesosoma in profile view **B** mesosoma in dorsal view **C** head in front view **D** global distribution map.

**Figure 131.**
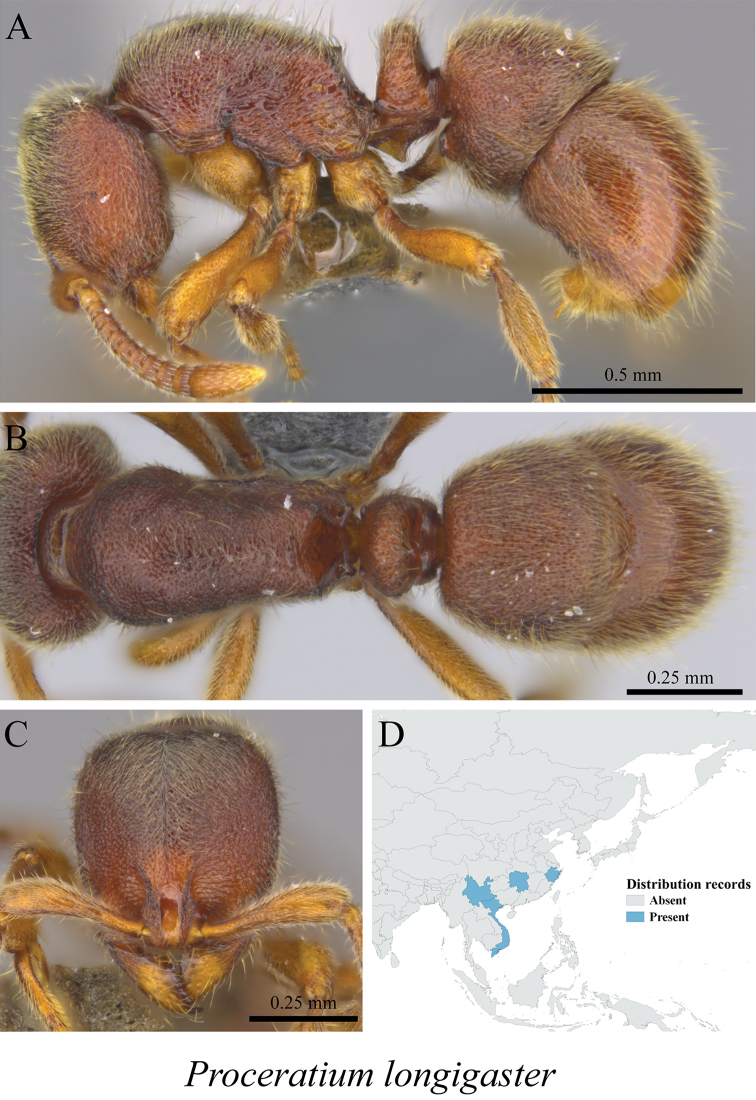
*Proceratium longigaster* worker (MCZ-ENT00759931) **A** mesosoma in profile view **B** mesosoma in dorsal view **C** head in front view **D** global distribution map.

**Figure 132.**
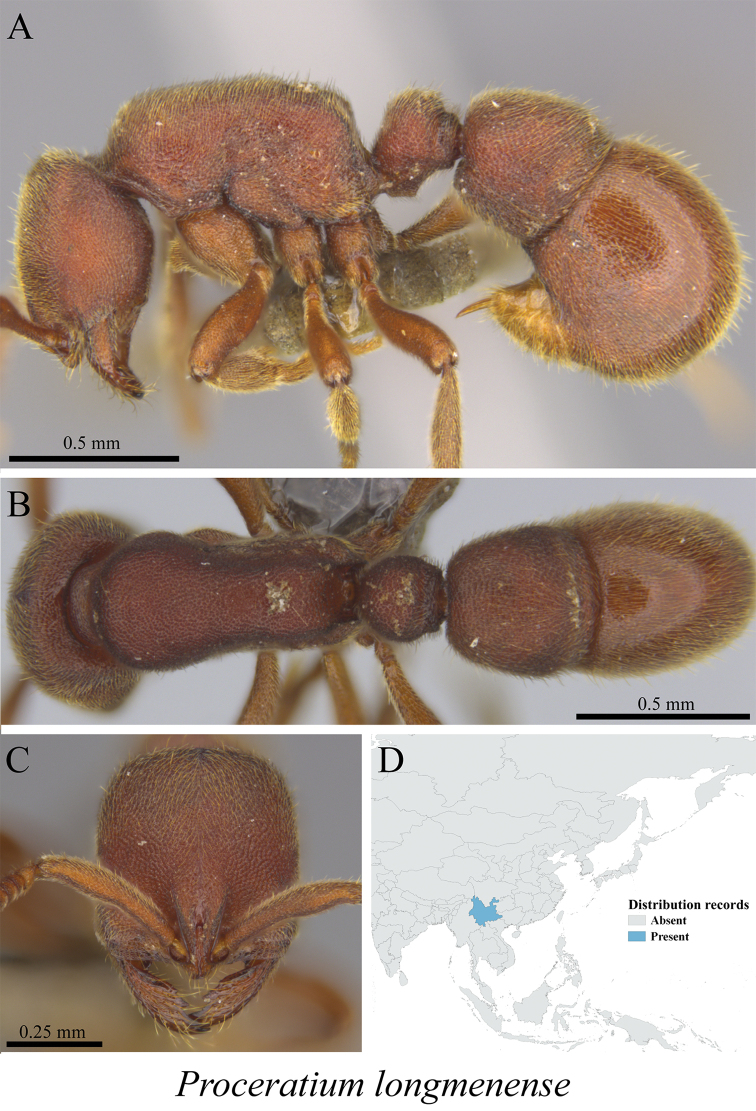
*Proceratium longmenense* worker (MCZ-ENT00763325) **A** mesosoma in profile view **B** mesosoma in dorsal view **C** head in front view **D** global distribution map.

**Figure 133.**
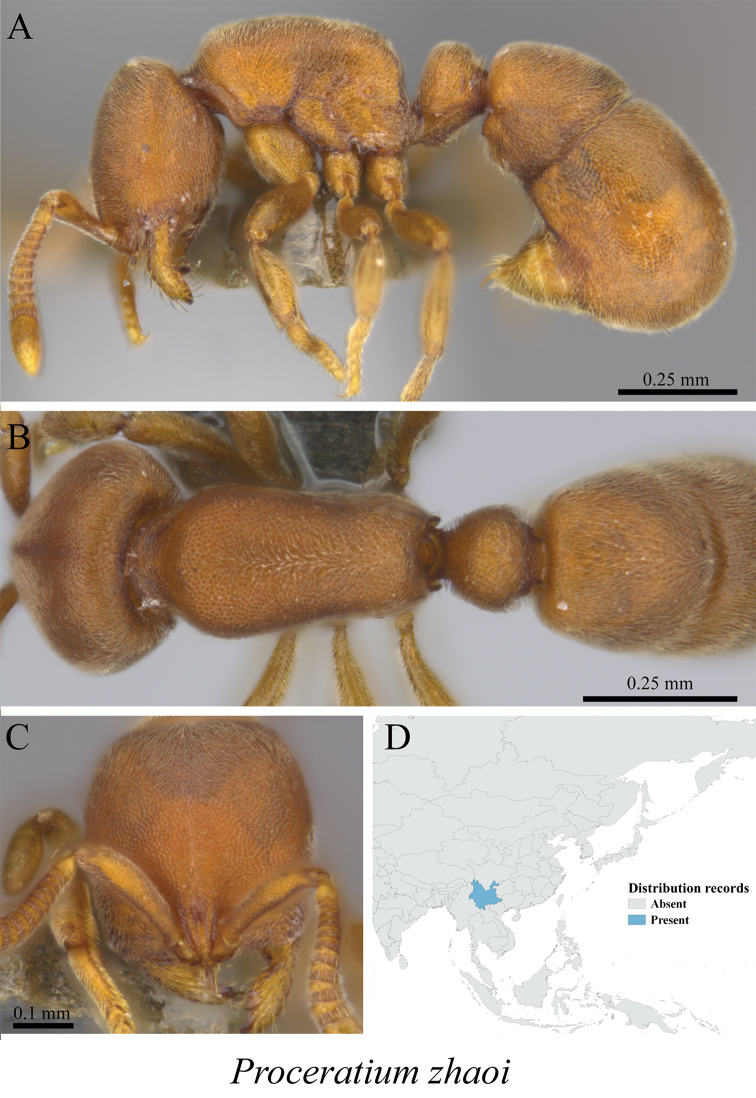
*Proceratium zhaoi* worker (MCZ-ENT00759857) **A** mesosoma in profile view **B** mesosoma in dorsal view **C** head in front view **D** global distribution map.

**Figure 134.**
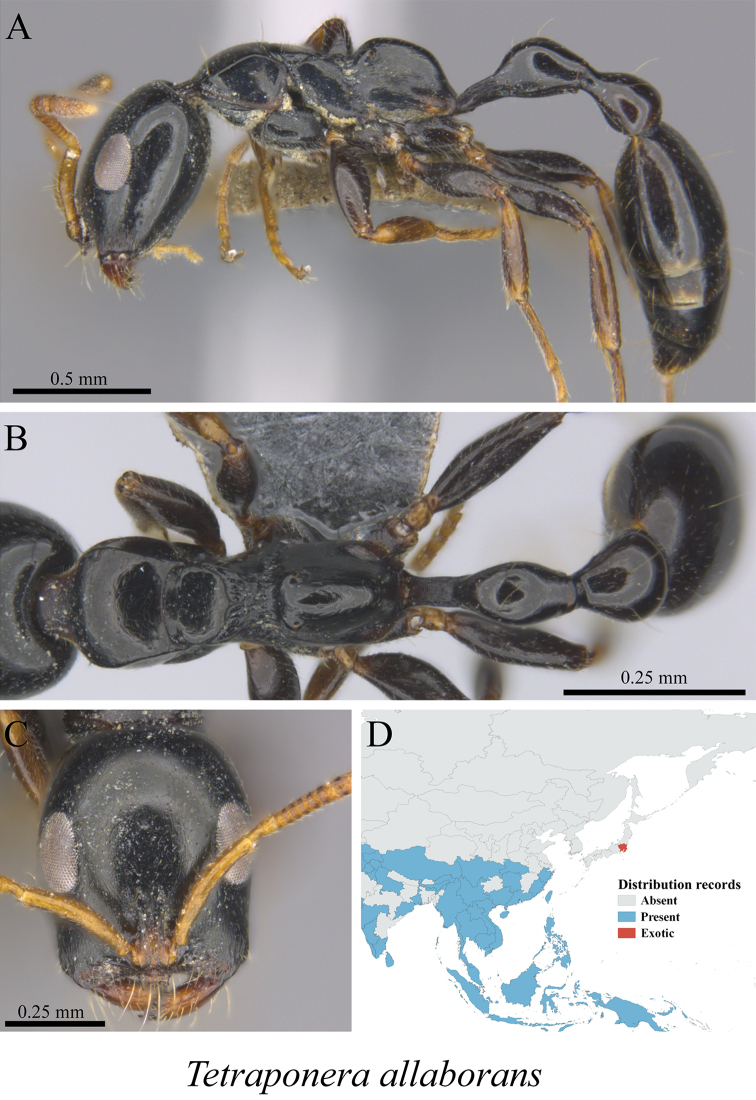
*Tetraponera allaborans* worker (MCZ-ENT00763523) **A** mesosoma in profile view **B** mesosoma in dorsal view **C** head in front view **D** global distribution map.

**Figure 135.**
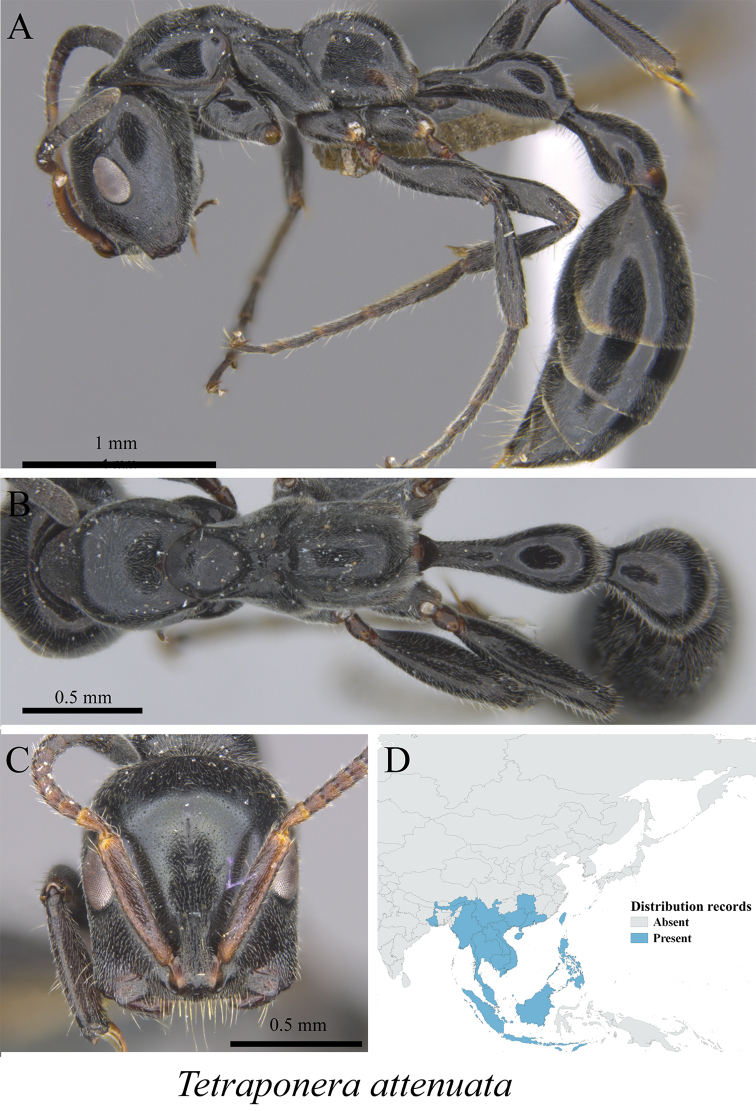
*Tetraponera attenuata* worker (MCZ-ENT00763165) **A** mesosoma in profile view **B** mesosoma in dorsal view **C** head in front view **D** global distribution map.

**Figure 136.**
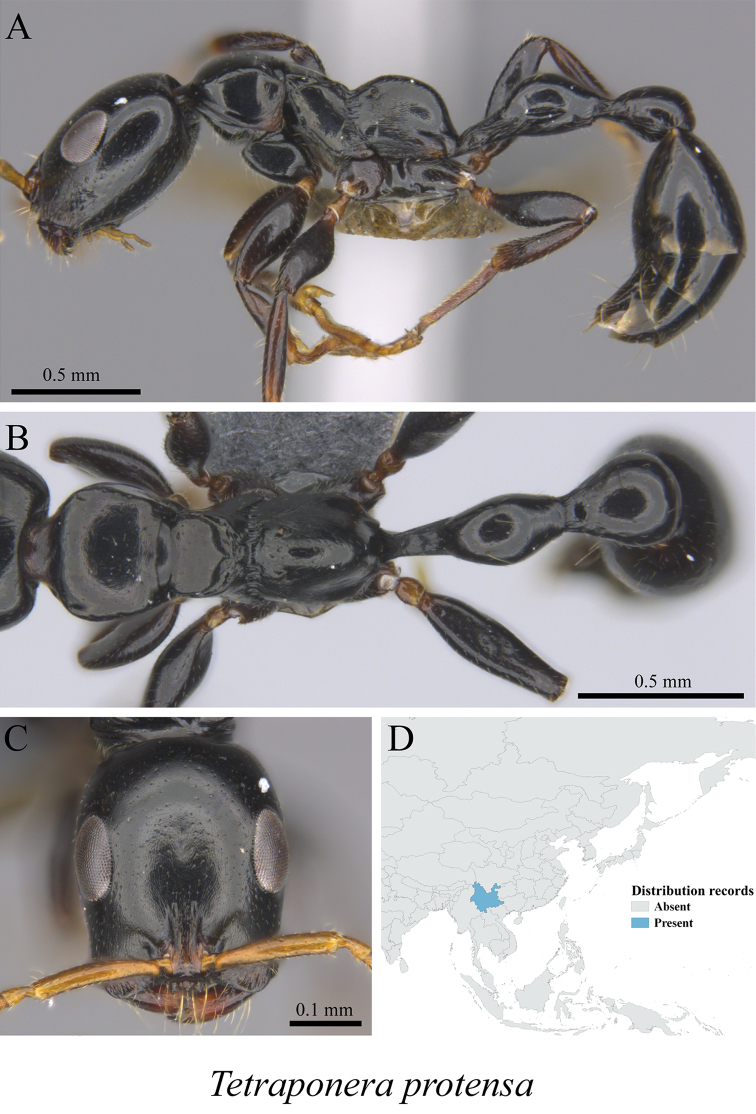
*Tetraponera protensa* worker (MCZ-ENT00763526) **A** mesosoma in profile view **B** mesosoma in dorsal view **C** head in front view **D** global distribution map.

#### Yunnan ant list

##### AMBLYOPONINAE

***Mystrium***: 1 species

*Mystrium camillae* Emery, 1989

***Prionopelta***: 1 species (undescribed)

*Prionopelta* sp.

***Stigmatomma***: 11 species

* *Stigmatomma amblyops* Karavaiev, 1935

* *Stigmatomma awa* (Xu, 2012)

*Stigmatomma crenatum* (Xu, 2001)

* *Stigmatomma kangba* (Xu, 2012)

* *Stigmatomma meilianum* (Xu, 2012)

* *Stigmatomma mulanae* (Xu, 2000)

*Stigmatomma octodentatum* (Xu, 2006)

*Stigmatomma rothneyi* (Forel, 1900)

* *Stigmatomma scrobiceps* (Guénard, 2013)

*Stigmatomma silvestrii* (Wheeler, 1928)

*Stigmatomma trilobum* (Xu, 2001)

##### DOLICHODERINAE

***Chronoxenus***: 3 species

*Chronoxenus myops* (Forel, 1895)

*Chronoxenus walshi* (Forel, 1895)

*Chronoxenus wroughtonii* (Forel, 1895)

***Dolichoderus***: 9 species

*Dolichoderus affinis* Emery, 1889

*Dolichoderus feae* Emery, 1889

*Dolichoderus incisus* Xu, 1995

* *Dolichoderus laotius* Santschi, 1920

*Dolichoderus moggridgei* Forel, 1886

*Dolichoderus sagmanotus* Xu, 2001

*Dolichoderus squamanodus* Xu, 2001

*Dolichoderus taprobanae* (Smith, 1858)

*Dolichoderus thoracicus* (Smith, 1860)

***Iridomyrmex***: 1 species

*Iridomyrmex anceps* (Roger, 1863)

***Liometopum***: 1 species

*Liometopum sinense* Wheeler, 1921

***Ochetellus***: 1 species

*Ochetellus glaber* (Mayr, 1862)

***Philidris***: 1 species

*Philidris laevigata* (Emery, 1895)

***Tapinoma***: 4 species

*Tapinoma geei* Wheeler, 1927

*Tapinoma indicum* Wheeler, 1895

*Tapinoma melanocephalum* (Fabricius, 1793)

*Tapinoma wroughtonii* Forel, 1904

***Technomyrmex***: 11 species

*Technomyrmex albipes* (Smith, 1861)

*Technomyrmex antennus* Zhou, 2001

*Technomyrmex bicolor* Emery, 1893

*Technomyrmex brunneus* Forel, 1895

*Technomyrmex elatior* Forel, 1902

*Technomyrmex horni* Forel, 1912

* *Technomyrmex kraepelini* Forel, 1905

*Technomyrmex obscurior* Wheeler, 1928

*Technomyrmex pratensis* (Smith, 1860)

* *Technomyrmex vitiensis* Mann, 1921

* *Technomyrmex yamanei* Bolton, 2007

##### DORYLINAE

***Aenictus***: 19 species

* *Aenictus artipus* Wilson, 1964

*Aenictus binghamii* Forel, 1900

* *Aenictus brevinodus* Jaitrong & Yamane, 2011

*Aenictus ceylonicus* (Mayr, 1866)

*Aenictus dentatus* Forel, 1911

*Aenictus feae* Emery, 1889

*Aenictus fergusoni* Forel, 1901

*Aenictus grandis* Bingham, 1903

*Aenictus hodgsoni* Forel, 1901

*Aenictus laeviceps* (Smith, 1857)

* *Aenictus maneerati* Jaitrong & Yamane, 2013

* *Aenictus paradentatus* Jaitrong & Yamane, 2012

*Aenictus piercei* Wheeler & Chapman, 1930

*Aenictus punensis* Forel, 1901

*Aenictus shuckardi* Forel, 1901

*Aenictus thailandianus* Terayama & Kubota, 1993

* *Aenictus watanasiti* Jaitrong & Yamane, 2013

*Aenictus westwoodi* Forel, 1901

* *Aenictus yangi* Liu, 2015

***Cerapachys***: 1 species

*Cerapachys sulcinodis* Emery, 1889

***Chrysapace***: 1 species

* *Chrysapace costatus* (Bharti & Wachkoo, 2013)

***Dorylus***: 3 species

*Dorylus laevigatus* (Smith, 1857)

*Dorylus orientalis* Westwood, 1835

*Dorylus vishnui* Wheeler, 1913

***Lioponera***: 1 species

*Lioponera longitarsus* (Mayr, 1879)

***Ooceraea***: 1 species

*Ooceraea biroi* (Forel, 1907)

***Parasyscia***: 1 species

*Parasyscia fossulata* (Forel, 1895)

***Simopone***: 1 species

* *Simopone yunnanensis* Chen, 2015

***Syscia***: 1 species

*Syscia typhla* Roger, 1861

***Yunodorylus***: 1 species

*Yunodorylus sexspinus* Xu, 2000

##### ECTATOMMINAE

***Gnamptogenys***: 6 species

*Gnamptogenys bicolor* (Emery, 1889)

*Gnamptogenys coccina* Zhou, 2001

* *Gnamptogenys coxalis* (Roger, 1860)

* *Gnamptogenys quadrutinodules* Chen, 2017

*Gnamptogenys sichuanensis* Lattke, 2004

* *Gnamptogenys sinensis* Wu & Xiao, 1987

* *Gnamptogenys treta* Lattke, 2004

##### FORMICINAE

***Acropyga***: 2 species

*Acropyga nipponensis* Terayama, 1985

*Acropyga yaeyamensis* Terayama & Hashimoto, 1996

***Anoplolepis***: 1 species

*Anoplolepis gracilipes* (Smith, 1857)

***Camponotus***: 28 species

*Camponotus albosparsus* Bingham, 1903

*Camponotus anningensis* Wu & Wang, 1989

*Camponotus auratiacus* Zhou, 2001

*Camponotus barbatus taylori* Forel, 1892

* *Camponotus bellus leucodiscus* Wheeler, 1919

* *Camponotus binghamii* Forel, 1894

*Camponotus chongqingensis* Wu & Wang, 1989

*Camponotus compressus* (Fabricius, 1787)

*Camponotus confucii* Forel, 1894

*Camponotus cornis* Wang & Wu, 1994

* *Camponotus crassisquamis* Forel, 1902

*Camponotus dolendus* Forel, 1892

*Camponotus exiguoguttatus* Forel, 1886

* *Camponotus fuscivillosus* Xiao & Wang, 1989

*Camponotus holosericeus* Emery, 1889

* *Camponotus invidus* Forel, 1892

* *Camponotus itoi* Forel, 1912

*Camponotus japonicus* Mayr, 1866

*Camponotus jianghuaensis* Xiao & Wang, 1989

*Camponotus lasiselene* Wang & Wu, 1994

*Camponotus minus* Wang & Wu, 1994

*Camponotus mitis* (Smith, 1858)

*Camponotus nicobarensis* Mayr, 1865

*Camponotus parius* Emery, 1889

*Camponotus pseudoirritans* Wu & Wang, 1989

*Camponotus pseudolendus* Wu & Wang, 1989

* *Camponotus radiatus* Forel, 1892

*Camponotus siemsseni* Forel, 1901

*Camponotus singularis* (Smith, 1858)

*Camponotus tonkinus* Santschi, 1925

*Camponotus vitiosus* (Smith, 1874)

***Colobopsis***: 7 species

*Colobopsis badia* (Smith, 1857)

* *Colobopsis ceylonica* (Emery, 1925)

*Colobopsis cotesii* (Forel, 1893)

*Colobopsis leonardi* (Emery, 1889)

*Colobopsis politae* (Wu & Wang, 1994)

*Colobopsis rothneyi* (Forel, 1893)

*Colobopsis vitrea* (Smithi, 1860)

***Echinopla***: 2 species

* *Echinopla cherapunjiensis* Bharti & Gul, 2012

* *Echinopla striata* Smith, 1857

***Formica***: 5 species

*Formica cunicularia* Latreille, 1798

* *Formica gagatoides* Ruzsky, 1904

*Formica glabridorsis* Santschi, 1925

* *Formica lemani* Bondroit, 1917

*Formica japonica* Motschoulsky, 1866

*Formica sanguinea* Latreille, 1798

*Formica sinensis* Wheeler, 1913

***Gesomyrmex***: 1 species

* *Gesomyrmex kalshoveni* Wheeler, 1929

***Lasius***: 6 species

*Lasius draco* Collingwood, 1982

*Lasius flavus* (Fabricius, 1782)

* *Lasius himalayanus* Bingham, 1903

*Lasius nipponensis* Forel, 1912

* *Lasius obscuratus* Stitz, 1930

*Lasius sichuense* Seifert, 2020

***Lepisiota***: 8 species

*Lepisiota acuta* Xu, 1994

*Lepisiota capensis* (Mayr, 1862)

*Lepisiota opaca* (Forel, 1892)

* *Lepisiota pulchella* (Forel, 1892)

*Lepisiota reticulata* Xu, 1994

*Lepisiota rothneyi* (Forel, 1894)

*Lepisiota rothneyi wroughtonii* (Forel, 1902)

*Lepisiota xichangensis* (Wu & Wang, 1995)

***Myrmoteras***: 2 species

*Myrmoteras binghamii* Forel, 1893

*Myrmoteras cuneonodus* Xu, 1998

***Nylanderia***: 10 species

*Nylanderia birmana* (Forel, 1902)

*Nylanderia bourbonica* (Forel, 1886)

* *Nylanderia emmae* (Forel, 1894)

* *Nylanderia flaviabdominis* (Wang, 1997)

*Nylanderia flavipes* (Smith, 1874)

*Nylanderia indica* (Forel, 1894)

*Nylanderia sharpii* (Forel, 1899)

*Nylanderia taylori* (Forel, 1894)

*Nylanderia vividula* (Nylander, 1846) (**Exotic**)

*Nylanderia yerburyi* (Forel, 1894)

***Oecophylla***: 1 species

*Oecophylla smaragdina* (Fabricius, 1775)

***Paraparatrechina***: 2 species

* *Paraparatrechina sakurae* (Ito, 1914)

*Paraparatrechina sauteri* (Forel, 1913)

***Paratrechina***: 1 species

*Paratrechina longicornis* (Latreille, 1802) (**Exotic**)

***Plagiolepis***: 4 species

*Plagiolepis alluaudi* Emery, 1894 (**Exotic**)

*Plagiolepis demangei* Santschi, 1920

*Plagiolepis exigua* Forel, 1894

* *Plagiolepis jerdonii* Forel, 1894

***Polyrhachis***: 32 species

*Polyrhachis armata* (Le Guillou, 1842)

*Polyrhachis bakana* Xu, 1998

*Polyrhachis bicolor* Smith, 1858

*Polyrhachis bihamata* (Drury, 1773)

*Polyrhachis brevicorpa* Xu, 2002

*Polyrhachis burmanensis* Donisthorpe, 1938

*Polyrhachis cornihumera* Xu, 2002

*Polyrhachis cornuhumera* Zhou & Huang, 2002

*Polyrhachis cyphonota* Xu, 1998

*Polyrhachis dentihumera* Xu, 2002

*Polyrhachis dives* Smith, 1857

* *Polyrhachis exercita* (Walker, 1859)

*Polyrhachis furcata* Smith, 1858

*Polyrhachis gibba* Emery, 1901

*Polyrhachis halidayi* Emery, 1889

* *Polyrhachis hippomanes* Emery, 1861

*Polyrhachis hippomanes ceylonensis* Emery, 1893

*Polyrhachis illaudata* Walker, 1859

*Polyrhachis jianghuaensis* Wang & Wu, 1991

*Polyrhachis laevigata* Smith, 1857

*Polyrhachis moesta* Emery, 1887

*Polyrhachis orbihumera* Xu, 2002

*Polyrhachis paracamponota* Wang & Wu, 1991

*Polyrhachis proxima* Roger, 1863

*Polyrhachis pubescens* Mayr, 1879

*Polyrhachis punctillata* Roger, 1863

*Polyrhachis rastellata* (Latreille, 1802)

*Polyrhachis rotoccipita* Xu, 2002

*Polyrhachis rufipes* Smith, 1858

*Polyrhachis thompsoni* Bingham, 1903

*Polyrhachis thrinax* Roger, 1863

*Polyrhachis tibialis* Smith, 1858

***Prenolepis***: 7 species

*Prenolepis angularis* Zhou, 2001

* *Prenolepis fustinoda* Williams & LaPolla, 2016

* *Prenolepis mediops* Williams & LaPolla, 2016

*Prenolepis melanogaster* Emery, 1893

*Prenolepis naoroji* Forel, 1902

* *Prenolepis shanialena* Williams & LaPolla, 2016

* *Prenolepis striata* Chen & Zhou, 2018

***Pseudolasius***: 6 species

*Pseudolasius bidenticlypeus* Xu, 1997

*Pseudolasius cibdelus* Wu & Wang, 1992

*Pseudolasius emeryi* Forel, 1911

*Pseudolasius familiaris* (Smith, 1860)

*Pseudolasius risii* Forel, 1894

*Pseudolasius silvestrii* Wheeler, 1927

##### LEPTANILLINAE

***Leptanilla***: 3 species

*Leptanilla hunanensis* Tang, Li & Chen, 1992

*Leptanilla kunmingensis* Xu & Zhang, 2002

*Leptanilla yunnanensis* Xu, 2002

***Protanilla***: 4 species

*Protanilla bicolor* Xu, 2002

*Protanilla concolor* Xu, 2002

* *Protanilla furcomandibula* Xu, 2002

*Protanilla gengma* Xu, 2012

##### MYRMICINAE

***Acanthomyrmex***: 2 species

*Acanthomyrmex glabfemoralis* Zhou & Zheng, 1997

*Acanthomyrmex luciolae* Emery, 1893

***Aphaenogaster***: 9 species

*Aphaenogaster beccarii* Emery, 1887

*Aphaenogaster exasperata* (Smith, 1921)

*Aphaenogaster famelica* (Smith, 1874)

*Aphaenogaster feae* Emery, 1889

* *Aphaenogaster geei* Wheeler, 1921

*Aphaenogaster japonica* Forel, 1911

*Aphaenogaster lepida* Wheeler, 1930

*Aphaenogaster rothneyi* (Forel, 1902)

*Aphaenogaster schurri* (Forel, 1902)

*Aphaenogaster smythiesii* (Forel, 1902)

***Cardiocondyla***: 3 species

* *Cardiocondyla itsukii* Seifert, Okita & Heinze, 2017 (**Exotic**)

*Cardiocondyla obscurior* Wheeler, 1929

*Cardiocondyla wroughtonii* (Forel, 1890)

***Carebara***: 18 species

*Carebara acutispina* (Xu, 2003)

*Carebara affinis* (Jerdon, 1951)

*Carebara altinoda* (Xu, 2003)

*Carebara asina* (Forel, 1902)

*Carebara bengalensis* (Forel, 1902)

*Carebara bihornata* (Xu, 2003)

*Carebara curvispina* (Xu, 2003)

* *Carebara diversa* (Jerdon, 1851)

* *Carebara jiangxiensis* Wu & Wang, 1995

*Carebara lignata* Westwood, 1840

* *Carebara melasolena* (Zhou & Zheng, 1997)

*Carebara obtusidenta* (Xu, 2003)

*Carebara polyphemus* (Wheeler, 1928)

*Carebara rectidorsa* (Xu, 2003)

*Carebara reticapita* (Xu, 2003)

*Carebara striata* (Forel, 2003)

*Carebara taiponica* (Wheeler, 1928)

*Carebara trechideros* (Zhou & Zheng, 1997)

*Carebara wheeleri* (Ettershank, 1966)

***Cataulacus***: 4 species

*Cataulacus granulatus* (Latreille,1802)

* *Cataulacus marginatus* Bolton, 1974

*Cataulacus simoni* Emery, 1893

*Cataulacus taprobanae* Smith, 1853

***Crematogaster***: 25 species

*Crematogaster anthracina* Smith, 1857

* *Crematogaster artifex* Mayr, 1879

*Crematogaster binghamii* Forel, 1904

*Crematogaster biroi* Mayr, 1897

* *Crematogaster contemta* Mayr, 1879

*Crematogaster dalyi* Forel, 1902

*Crematogaster dohrni* Mayr, 1879

*Crematogaster ebenina* Forel, 1902

*Crematogaster ferrarii* Emery, 1888

*Crematogaster hodgsoni* Forel, 1902

* *Crematogaster inflata* Smith, 1857

*Crematogaster macaoensis* Wu & Wang, 1995

*Crematogaster matsumurai* Forel, 1901

*Crematogaster nawai* Ito, 1914

*Crematogaster osakensis* Forel, 1900

*Crematogaster politula* Forel, 1902

* *Crematogaster quadriruga* Forel, 1911

*Crematogaster rogenhoferi* Mayr, 1879

*Crematogaster rothneyi* Mayr, 1879

*Crematogaster subnuda* Mayr, 1879

*Crematogaster travancorensis* Forel, 1902

*Crematogaster treubi* Emery, 1896

*Crematogaster walshi* Forel, 1902

*Crematogaster wroughtonii* Forel, 1902

*Crematogaster zoceensis* Santschi, 1925

***Dilobocondyla***: 3 species

* *Dilobocondyla eguchii* Bharti & Kumar, 2013

*Dilobocondyla fouqueti* Santschi, 1910

* *Dilobocondyla gasteroreticulata* Bharti & Kumar, 2013

***Erromyrma***: 1 species

*Erromyrma latinodis* (Mayr, 1872)

***Gaoligongidris***: 1 species

*Gaoligongidris planodorsa* Xu, 2012

**Gauromyrmex**: 1 species

*Gauromyrmex acanthinus* (Karavaiev, 1935)

***Kartidris***: 3 species

*Kartidris ashima* Xu & Zheng, 1995

*Kartidris nyos* Bolton, 1991

*Kartidris sparsipila* Xu, 1999

***Lasiomyrma***: 1 species (undescribed)

*Lasiomyrma* sp.

***Lophomyrmex***: 3 species

*Lophomyrmex bedoti* Emery, 1893

*Lophomyrmex birmanus* Emery, 1893

*Lophomyrmex quadrispinosus* (Jerdon, 1851)

***Lordomyrma***: 1 species (undescribed)

*Lordomyrma* sp.

***Meranoplus***: 2 speices

*Meranoplus bicolor* (Guérin-Méneville, 1844)

*Meranoplus laeviventris* Emery, 1889

***Messor***: 1 species

* *Messor aciculatus* (Smith, 1874)

***Monomorium***: 5 species

*Monomorium chinense* Santschi, 1925

*Monomorium floricola* (Jerdon, 1851)

* *Monomorium hainanense* Wu & Wang, 1995

*Monomorium orientale* Mayr, 1879

*Monomorium pharaonis* (Linnaeus, 1758)

***Myrmecina***: 5 species

* *Myrmecina asiatica* Okido, Ogata & Hosoishsi, 2020

* *Myrmecina asthena* Okido, Ogata & Hosoishsi, 2020

*Myrmecina curvispina* Zhou, Huang & Ma, 2008

*Myrmecina guangxiensis* Zhou, 2001

* *Myrmecina sinensis* Wheeler, 1921

*Myrmecina striata* Emery, 1889

*Myrmecina taiwana* Terayama, 1995

***Myrmica***: 11 species

*Myrmica curiosa* Radchenko, Zhou & Elmes, 2008

*Myrmica draco* Radchenko, Zhou & Elmes, 2008

* *Myrmica excelsa* Kupyanskaya, 1990

* *Myrmica heterorhytida* Radchenko & Elmes, 2008

*Myrmica margaritae* Emery, 1889

*Myrmica pleiorhytida* Radchenko & Elmes, 2009

*Myrmica polyglypta* Radchenko & Rigato, 2008

*Myrmica ritae* Emery, 1889

*Myrmica serica* Wheeler, 1928

*Myrmica sinensis* Radchenko, Zhou & Elmes, 2008

*Myrmica titanica* Mayr, 2001

*Myrmica yunnanensis* Radchenko & Elmes, 2008

***Myrmicaria***: 1 species

*Myrmicaria brunnea* Saunders, 1842

***Perissomyrmex***: 2 species

*Perissomyrmex bidentatus* Zhou & Huang, 2006

*Perissomyrmex fissus* Xu & Wang, 2004

***Pheidole***: 42 species

*Pheidole allani* Bingham, 1903

*Pheidole binghamii* Forel, 1902

*Pheidole capellinii* Emery, 1902

*Pheidole constanciae* Forel, 1902

*Pheidole elongicephala* Eguchi, 2008

*Pheidole exasperata* (Mayr, 1866)

*Pheidole fervens* Smith, 1858

*Pheidole fervida* Smith, 1874

* *Pheidole fortis* Eguchi, 2006

*Pheidole gatesi* (Wheeler, 1927)

* *Pheidole hongkongensis* Wheeler, 1928

*Pheidole indica* Mayr, 1879

* *Pheidole indosinensis* Wheeler, 1928

*Pheidole jucunda* Forel, 1885

* *Pheidole laevicolor* Eguchi, 2006

* *Pheidole magna* Eguchi, 2006

*Pheidole multidens* Forel, 1902

*Pheidole nietneri* Emery, 1901

* *Pheidole nodifera* Smith, 1858

*Pheidole nodus* Smith, 1874

* *Pheidole ochracea* Eguchi, 2008

* *Pheidole parva* Mayr, 1865

*Pheidole pieli* Santschi, 1925

* *Pheidole plagiaria* Smith, 1860

* *Pheidole planifrons* Santschi, 1920

* *Pheidole rabo* Forel, 1913

*Pheidole roberti* Forel, 1902

* *Pheidole rugithorax* Eguchi, 2008

*Pheidole sagei* Forel, 1902

* *Pheidole singaporensis* Özdikmen, 2010

*Pheidole sinica* (Wu & Wang, 1992)

* *Pheidole smythiesii* Forel, 1902

*Pheidole spathifera* Forel, 1902

*Pheidole sulcaticeps* Roger, 1863

* *Pheidole tandjongensis* Forel, 1913

* *Pheidole tjibodana* Forel, 1905

* *Pheidole tumida* Eguchi, 2008

* *Pheidole vieti* Eguchi, 2008

* *Pheidole vulgaris* Eguchi, 2006

*Pheidole watsoni* Forel, 1902

*Pheidole yeensis* Forel, 1902

* *Pheidole zoceana* Santschi, 1925

***Pristomyrmex***: 4 species

*Pristomyrmex brevispinosus* Emery, 1887

*Pristomyrmex hamatus* Xu & Zhang, 2002

*Pristomyrmex punctatus* (Smith, 1860)

*Pristomyrmex sulcatus* Emery, 1895

***Recurvidris***: 3 species

* *Recurvidris kemneri* (Wheeler, 1954)

*Recurvidris nuwa* Xu & Zheng, 1995

*Recurvidris recurvispinosa* (Forel, 1890)

***Rhopalomastix***: 2 species

* *Rhopalomastix rothneyi* Forel, 1900

*Rhopalomastix umbracapita* Xu, 1999

***Rotastruma***: 1 species

* *Rotastruma stenoceps* Bolton, 1991

***Solenopsis***: 3 species

*Solenopsis indagatrix* Wheeler, 1928

*Solenopsis invicta* Buren, 1972 (**Exotic**)

*Solenopsis jacoti* Wheeler, 1923

***Stenamma***: 4 species

*Stenamma ailaoense* Liu & Xiu, 2011

*Stenamma gurkhale* DuBois, 1998

* *Stenamma jeriorum* DuBois, 1998

*Stenamma wumengense* Liu & Xiu, 2011

***Strumigenys***: 24 species

*Strumigenys ailaoshana* Xu & Zhou, 2004

* *Strumigenys assamensis* De Andrade, 1994

*Strumigenys dayui* (Xu, 2000)

* *Strumigenys doriae* Emery, 1887

* *Strumigenys dyschima* (Bolton, 2000)

*Strumigenys exilirhina* Bolton, 2000

*Strumigenys feae* Emery, 1895

* *Strumigenys kichijo* (Terayama, 1996)

* *Strumigenys leptothrix* Wheeler, 1929

*Strumigenys lewisi* Cameron, 1886

* *Strumigenys lyroessa* (Roger, 1862)

* *Strumigenys membranifera* Emery, 1869 (**Exotic**)

* *Strumigenys mitis* (Brown, 2000)

*Strumigenys mutica* (Brown, 1949)

*Strumigenys nanzanensis* Lin & Wu, 1996

* *Strumigenys nepalensis* De Andrade, 1994

*Strumigenys nongba* (Xu & Zhou, 2004)

* *Strumigenys paraposta* Bolton, 2000

* *Strumigenys rallarhina* Bolton, 2000

* *Strumigenys sauteri* (Forel, 1912)

*Strumigenys strygax* Bolton, 2000

* *Strumigenys sydorata* Bolton, 2000

* *Strumigenys taphra* (Bolton, 2000)

* *Strumigenys tritomea* Bolton, 2000

*Strumigenys yangi* (Xu & Zhou, 2004)

***Temnothorax***: 7 species

*Temnothorax angulohumerus* Zhou, 2010

*Temnothorax congruus* (Smith, 1874)

*Temnothorax hengshanensis* (Huang, 2004)

*Temnothorax nassonovi* (Ruzsky, 1895)

*Temnothorax orchidus* Zhou, 2010

*Temnothorax striatus* Zhou, 2010

*Temnothorax wui* (Wheeler, 1929)

***Tetramorium***: 29 species

*Tetramorium aptum* Bolton, 1977

*Tetramorium bicarinatum* (Nylander, 1846)

*Tetramorium cardiocarenum* Xu & Zheng, 1994

*Tetramorium ciliatum* Bolton, 1977

*Tetramorium crepum* Wang & Wu, 1988

*Tetramorium cuneinode* Bolton, 1977

*Tetramorium cyclolobium* Xu & Zheng, 1994

* *Tetramorium difficile* Bolton, 1977

* *Tetramorium flavipes* Emery, 1893

*Tetramorium indosinense* Wheeler, 1927

*Tetramorium insolens* (Smith, 1861)

*Tetramorium kheperra* (Bolton, 1976)

*Tetramorium kraepelini* Forel, 1905

*Tetramorium lanuginosum* Mayr, 1870

*Tetramorium laparum* Bolton, 1977

*Tetramorium nipponense* Wheeler, 1928

*Tetramorium nursei* Bingham, 1903

*Tetramorium obtusidens* Viehmeyer, 1916

*Tetramorium pacificum* Mayr, 1870

* *Tetramorium parvispinum* (Emery, 1893)

* *Tetramorium polymorphum* Yamane & Jaitrong, 2011

*Tetramorium repletum* Wang & Xiao, 1988

*Tetramorium simillimum* (Smith, 1851) (**Exotic**)

*Tetramorium smithi* Mayr, 1879

* *Tetramorium tonganum* Mayr, 1870

*Tetramorium walshi* (Forel, 1890)

* *Tetramorium wroughtonii* (Forel, 1902)

*Tetramorium yerburyi* Forel, 1902

*Tetramorium yulongense* Xu & Zheng, 1994

***Trichomyrmex***: 2 species

*Trichomyrmex destructor* (Jerdon, 1851) (**Exotic**)

*Trichomyrmex mayri* (Forel, 1902) (**Exotic**)

***Vollenhovia***: 2 species

* *Vollenhovia lucimandibula* Wang, 2005

*Vollenhovia pyrrhoria* Wu & Xiao, 1989

***Vombisidris***: 1 species

* *Vombisidris tibeta* Xu & Yu, 2012

##### PONERINAE

***Anochetus***: 6 species

*Anochetus graeffei* Mayr, 1870

* *Anochetus madaraszi* Mayr, 1897

*Anochetus mixtus* Radchenko, 1993

* *Anochetus myops* Emery, 1893

*Anochetus risii* Forel, 1900

*Anochetus subcoecus* Forel, 1912

***Brachyponera***: 2 species

*Brachyponera brevidorsa* Xu, 1994

* *Brachyponera chinensis* (Emery, 1895)

*Brachyponera luteipes* (Mayr, 1862)

***Buniapone***: 1 species

*Buniapone amblyops* (Emery, 1887)

***Centromyrmex***: 1 species

*Centromyrmex feae* (Emery, 1889)

***Cryptopone***: 5 species

*Cryptopone gigas* Wu & Wang, 1995

*Cryptopone recticlypea* Xu, 1998

* *Cryptopone sauteri* (Wheeler, 1906)

*Cryptopone taivanae* (Forel, 1930)

*Cryptopone testacea* Emery, 1893

***Diacamma***: 1 species

* *Diacamma rugosum* (Le Guillou, 1842)

***Ectomomyrmex***: 8 species

*Ectomomyrmex annamitus* (André, 1892)

*Ectomomyrmex astutus* (Smith, 1858)

*Ectomomyrmex javanus* Mayr, 1867

*Ectomomyrmex leeuwenhoeki* (Forel, 1886)

*Ectomomyrmex lobocarenus* (Xu, 1995)

* *Ectomomyrmex obtusus* Emery, 1900

*Ectomomyrmex sauteri* (Forel, 1912)

*Ectomomyrmex zhengi* (Xu, 1995)

***Emeryopone***: 1 species

*Emeryopone melaina* Xu, 1998

***Euponera***: 1 species

*Euponera pilosior* (Wheeler, 1928)

***Harpegnathos***: 1 species

*Harpegnathos venator* (Smith, 1858)

***Hypoponera***: 7 species

*Hypoponera ceylonensis* (Mayr, 1897)

*Hypoponera confinis* (Roger, 1860)

* *Hypoponera ergatandria* (Forel, 1893) (**Exotic**)

*Hypoponera nippona* (Santschi, 1937)

*Hypoponera punctatissima* (Roger, 1859) (**Exotic**)

*Hypoponera sauteri* Onoyama, 1989

*Hypoponera truncata* (Smith, 1860)

***Leptogenys***: 17 species

*Leptogenys binghamii* Forel, 1900

*Leptogenys birmana* Forel, 1900

*Leptogenys chinensis* (Mayr, 1870)

*Leptogenys crassicornis* Emery, 1895

* *Leptogenys davydovi* Karavaiev, 1935

*Leptogenys diminuta* (Smith, 1857)

* *Leptogenys kitteli* (Mayr, 1870)

* *Leptogenys kraepelini* Forel, 1905

*Leptogenys laozii* Xu, 2000

*Leptogenys lucidula* Emery, 1895

*Leptogenys mengzii* Xu, 2000

*Leptogenys pangui* Xu, 2000

* *Leptogenys peuqueti* (André, 1887)

* *Leptogenys processionalis* (Jerdon, 1851)

* *Leptogenys rufida* Zhou, 2012

* *Leptogenys sunzii* Xu, 2015

*Leptogenys zhuangzii* Xu, 2000

***Mesoponera***: 1 species

*Mesoponera melanaria* (Emery, 1893)

***Myopias***: 3 species

*Myopias conicara* Xu, 1998

* *Myopias daia* Xu, 2014

* *Myopias hania* Xu, 2012

***Odontomachus***: 6 species

*Odontomachus circulus* Wang, 1993

* *Odontomachus fulgidus* Wang, 1993

*Odontomachus granatus* Wang, 1993

*Odontomachus monticola* Emery, 1892

*Odontomachus rixosus* Smith, 1857

*Odontomachus tensus* Wang, 1993

***Odontoponera***: 1 species

* *Odontoponera denticulata* (Smith, 1858)

***Platythyrea***: 2 species

*Platythyrea clypeata* Forel, 1911

* *Platythyrea parallela* (Smith, 1859)

***Ponera***: 14 species

*Ponera alisana* Terayama, 1986

*Ponera baka* Xu, 2001

*Ponera bawana* Xu, 2001

* *Ponera chiponensis* Terayama, 1986

*Ponera diodonta* Xu, 2001

*Ponera longlina* Xu, 2001

*Ponera menglana* Xu, 2001

*Ponera nangongshana* Xu, 2001

* *Ponera paedericera* Zhou, 2001

*Ponera pentodontos* Xu, 2001

*Ponera pianmana* Xu, 2001

*Ponera scabra* Wheeler, 1928

*Ponera sinensis* Wheeler,1928

*Ponera xantha* Xu, 2001

***Pseudoneoponera***: 2 species

*Pseudoneoponera bispinosa* (Smith, 1858)

*Pseudoneoponera rufipes* (Jerdon, 1851)

##### PROCERATIINAE

***Discothyrea***: 3 species

* *Discothyrea banna* Xu, 2014

* *Discothyrea diana* Xu, 2014

*Discothyrea sauteri* Forel, 1912

***Probolomyrmex***: 1 species

*Probolomyrmex longiscapus* Xu & Zeng, 2000

***Proceratium***: 4 species

*Proceratium longigaster* Karavaiev, 1935

*Proceratium longmenense* Xu, 2006

* *Proceratium shohei* Staab, 2018

*Proceratium zhaoi* Xu, 2000

##### PSEUDOMYRMECINAE

***Tetraponera***: 12 species

*Tetraponera allaborans* (Walker, 1859)

*Tetraponera amargina* Xu & Chai, 2004

*Tetraponera attenuata* Smith, 1877

*Tetraponera binghami* (Forel, 1902)

*Tetraponera concava* Xu & Chai, 2004

*Tetraponera convexa* Xu & Chai, 2004

*Tetraponera furcata* Xu & Chai, 2004

*Tetraponera microcarpa* Wu & Wang, 1990

*Tetraponera nitida* (Smith, 1860)

*Tetraponera notabilis* Ward, 2001

*Tetraponera protensa* Xu & Chai, 2004

*Tetraponera rufonigra* (Jerdon, 1851)

## Discussion

### Ants in the Hengduan Mountain region

Field inventories and data synthesis efforts are essential for our understanding of ant diversity in ‘hotspots’ that harbor most of Earth’s biodiversity. Our study represents new survey data from an understudied region. We produce the first ant species checklist from China’s Hengduan Mountains (130 species).

A majority of the ant species were only collected below 1500 m, consistent with the strong effect of elevation on ant diversity observed elsewhere (Suppl. material 1, Fig. S1). This also suggests that future sampling in low elevation areas may increase species detection. For example, the number of *Strumigenys* species recovered in this survey is relatively low compared to the overall richness of this genus. This could be because we have relatively few collection events at low elevations where many of these species are known to occur. Indeed, all six *Strumigenys* species were collected below 1000 m from only three independent Winkler sampling sites.

Many of the new records in our collection such as *Aenictus brevinodus*, *Camponotus bellus leucodiscus*, *Cataulacus marginatus*, *Crematogaster quadriruga*, *Dilobocondyla eguchii*, *Gnamptogenys quadrutinodules*, and *Strumigenys taphra* represent the northern-most records of their known distributional ranges. Species records such as *Aenictus brevinodus*, *Camponotus bellus leucodiscus*, *Camponotus keihitoi*, *Cataulacus marginatus*, *Gnamptogenys quadrutinodules*, and *Strumigenys taphra* show a disjunction from the rest of their known distributions. It is unclear whether those records represent true biogeographic disjunctions, or sampling / taxonomic artifacts. Another potential reason could be that they were collected in the past, but have not been reported due to the lack of taxonomic infrastructure and species check lists from this region (Guénard et al. 2017). Additional inventories of ant diversity and taxonomic treatments are needed to answer these questions.

Despite the comparatively small area of China’s Hengduan Mountains that we explored for this inventory of myrmecofauna, we were able to collect 130 species, which accounts for more than 24 % of the total number of ant species (N = 550) for Yunnan province. Among them, more than 10% of the ant species that were collected in this survey represent new records for Yunnan province. Moreover, there are still more than 41 morphospecies (32% of the total collected) that we believe are undescribed and new to science. To date, three *Myrmecina* species (Figs 82–84) and one *Gauromyrmex* species (Fig. 75) are undergoing taxonomic revision, and species descriptions are being prepared.

Our sampling of the full ant diversity of the Hengduan mountain region is still relatively limited. For example, we only had one sampling site per elevation, which is insufficient to cover the complex topology of the Hengduan Mountains. We also only used leaf litter extraction and hand collection, which is unlikely to recover complete ant assemblages. The incorporation of additional sampling techniques into our methodology, such as pitfall trapping, soil baiting, twig sampling, light trapping and canopy fogging, will cover more strata and lifestyles, and thus significantly increase our rate of species discovery. Overall, our results highlight how little was previously known about the ant fauna in this region and emphasize the need for further collecting in order to better understand the hidden ant biodiversity in China’s Hengduan Mountains, and Yunnan Province overall.

### Ants in Yunnan

If the total species richness of ants in Yunnan, with 550 species, is still an underestimate of the full species numbers, the exceptional diversity of genera encountered in this region needs to be highlighted. With 99 genera, Yunnan generic diversity is only matched globally by a few regions in South East Asia, and Queensland, Australia. A major difference with other Asian regions lies in the composition of the genera retrieved and their origin. For instance, genera found in Borneo, Sumatra, Java, Vietnam, Thailand, Peninsular Malaysia are almost exclusively derived from tropical, Oriental origins. In contrast, the geographic location and topography of Yunnan province has promoted an intermixing of taxa from several biogeographic regions: the Oriental realm from the south, the Palearctic realm from the northwest, and the Sino-Japanese realm from the northeast. As a result, the composition of the Yunnan ant fauna includes both tropical, subtropical, and temperate elements. Such intermixed communities are evident even at a small scale. For instance, during previous fieldwork conducted in the Gaoligongshan Mountains in 2015 by two of the authors (BG and CL), for which specimens were unfortunately lost, the coexistence of tropical (*Dorylus*, *Ectomomyrmex*), subtropical (*Temnothorax*) and temperate genera (*Formica*, *Lasius*) was observed on a hillside at an elevation of about 1900 m on an ~ 250 m² patch of grassland. Interestingly, while this area exhibited a transition where fauna from distinctly different origins coexisted along a thin band of altitude, at lower elevations, tropical genera were dominant and at higher elevations, temperate genera became dominant. Overall, this generated an unexpectedly diverse faunal composition, with such mixed communities contemplated by Wheeler (1915) to explain the generic composition and diversity of fossil ants observed in Baltic amber. Possibly, the ant composition of genera now retrieved within Yunnan might represent the remains of a once more widespread assemblage found within Asia and Europe during the Miocene (Guénard et al. 2015). This highlights the specific nature of the Yunnan ant fauna and its importance in studying ant biogeography within Asia. It also serves as an excellent example of the formation and stability of ant community assemblages over time. Other neighboring regions such as Myanmar, Bhutan, Nepal or northeast India are likely to exhibit similar features, but to this point, the myrmecological exploration of these regions has been largely fragmentary (Guénard et al. 2010, 2012).

The diversity of Yunnan ants is also remarkable for particular ant genera for which their global peak of diversity is encountered in the region. While it is important to note that the global diversity of specific genera as well as their overall taxonomic descriptions remain incomplete, seven genera present their highest currently known global diversity in Yunnan (*Cryptopone*: 5 species, *Ectomomyrmex*: 8 species, *Kartidris*: 3 species, *Perissomyrmex*: 2 species, *Ponera*: 14 species, *Prenolepis*: 7 species, *Stigmatomma*: 11 species), while six others are remarkable by the level of global diversity there, among the highest observed globally (*Aenictus*: 19 species, *Carebara*: 19 species, *Dilobocondyla*: 3 species, *Myrmecina*: 7 species, *Proceratium*: 4 species, *Recurvidris*: 3 species).

In conclusion, the important topographic variation, with mountain ranges aligned along a north-south axis combined with the presence of multiple climatic zones, including tropical rainforest in the lowland areas of the southern part of Yunnan create a diversity of microhabitats for supporting a diverse ant fauna. Moreover, the geographic position of Yunnan at the confluence of three biogeographic realms may promote ant diversity in the region. The collection of these 16 new ant records for Yunnan together with our previous discovery of 40 new ant records for Yunnan (Liu et al. 2015a) suggest that the true ant diversity in Yunnan is significantly higher. Moreover, the species diversity of some ant genera in nearby regions (based on data from GABI) also suggests that some ant genera sampled will ultimately be much more diverse in Yunnan (Guénard et al. 2017). For example, Hong Kong has recorded a similar diversity of *Strumigenys* species while having an area nearly 350 times smaller and a much less diverse topography compare to Yunnan (Tang et al. 2019), perhaps because leaf litter extraction has not been widely used for sampling ants in Yunnan. Thus, our survey to date indicates that further intensive sampling focused on different ecological strata (arboreal, leaf litter, subterranean) and combining various methods of extraction in both tropical and mountain habitats should yield many additional records and new species discovery in this region.

## Supplementary materials

Supplementary material 1 Figure S1. Ant species richness pattern along an elevational gradient in the Hengduan Mountains Data type Image https://binary.pensoft.net/file/466093This dataset is made available under the Open Database License (http://opendatacommons.org/licenses/odbl/1.0/). The Open Database License (ODbL) is a license agreement intended to allow users to freely share, modify, and use this Dataset while maintaining this same freedom for others, provided that the original source and author(s) are credited.Cong Liu, Georg Fischer, Francisco Hita Garcia, Seiki Yamane, Qing Liu, Yan Qiong Peng, Evan P. Economo, Benoit Guénard, Naomi E. Pierce
