# Supplementary figures and images for: Ants of the Hengduan Mountains: a new altitudinal survey and updated checklist for Yunnan Province highlight an understudied insect biodiversity hotspot
Source: Zookeys. 2020 Oct 26;978:1–171. doi: 10.3897/zookeys.978.55767 (PMC7606585; doi:10.3897/zookeys.978.55767)

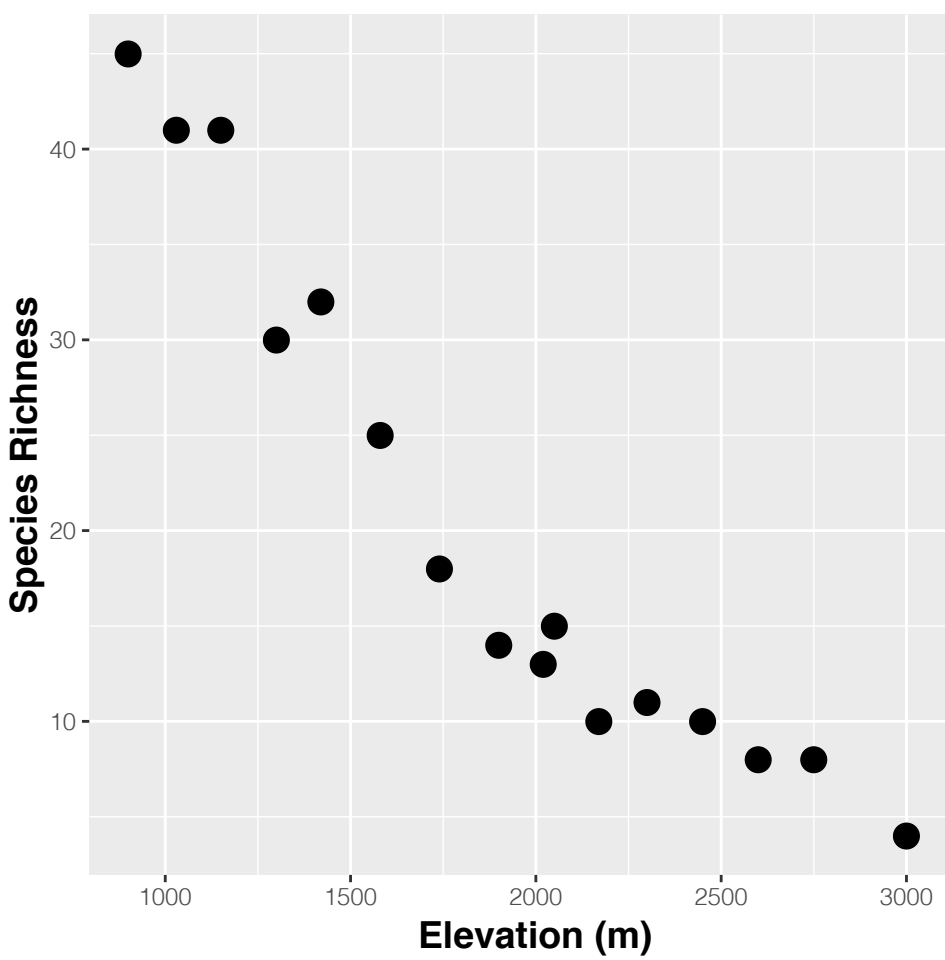

Supplement: Supplementary material 1 — Figure S1. Ant species richness pattern along an elevational gradient in the Hengduan Mountains [file zookeys-978-001-s001.pdf]
